# Burden of stroke in the United States of America, 1990–2021: a systematic analysis for the US burden of disease study 2021

**DOI:** 10.3389/fneur.2025.1609508

**Published:** 2025-08-14

**Authors:** Jung-Hyun Park, Yoonkyung Chang, Somin Park, Tae-Jin Song

**Affiliations:** ^1^Department of Medicine, Ewha Womans University College of Medicine, Seoul, Republic of Korea; ^2^Department of Neurology, Mokdong Hospital, Ewha Womans University College of Medicine, Seoul, Republic of Korea; ^3^Department of Neurology, Seoul Hospital, Ewha Womans University College of Medicine, Seoul, Republic of Korea

**Keywords:** stroke, global burden of disease, disability-adjusted life years, death, epidemiology

## Abstract

**Objectives:**

Accurate and updated stroke burden estimates are essential to inform public health interventions and resource allocation in the United States (US). We aimed to evaluate the burden of ischemic and hemorrhagic stroke in the US in 2021 and analyze trends from 1990 to 2021 by age, sex, and geographic location.

**Methods:**

This was a comprehensive analysis based on the 2021 Global Burden of Disease (GBD) study encompassing ischemic stroke, intracerebral hemorrhage (ICH), and subarachnoid hemorrhage (SAH). The stroke incidence, prevalence, mortality, and disability-adjusted life-years (DALYs), including absolute numbers and age-standardized rates per 100,000 population, were stratified by stroke subtype, sex, age, and geographic region.

**Results:**

In 2021, there were 0.41 million incident strokes (95% uncertainty interval (UI), 0.36–0.47 million), predominantly ischemic (0.31 million, 75.6%). The prevalence was 6.3 million, with ischemic stroke accounting for 78% (3.07 million, 48.7% men). Hemorrhagic strokes included 0.75 million ICH and 0.45 million SAH. Stroke deaths totaled 0.19 million, with DALYs of 3.91 million. From 1990 to 2021, the crude stroke prevalence markedly increased for ischemic stroke (65.7%), ICH (78.3%), and SAH (70.6%). Although age-standardized incidence and mortality rates generally decreased over this period, the incidence of SAH has increased recently, and hemorrhagic stroke mortality peaked around 2000. Younger populations (aged 15–49 years) experienced an increasing stroke burden, especially in Alaska and Arkansas, highlighting demographic and regional disparities.

**Conclusion:**

Despite improvements in age-standardized stroke incidence, mortality, and DALYs, the overall burden of stroke continues to increase owing to demographic shifts and the increasing prevalence of risk factors. There is a critical need for tailored and targeted interventions to address the evolving demographic and regional disparities and effectively reduce the US stroke burden.

## 1 Introduction

Stroke remains a significant global health concern, with approximately 15 million individuals experiencing it worldwide (1, 2). In the United States (US), stroke continues to be a leading cause of disability and death (3, 4). Accordingly, population-level data on the stroke incidence, prevalence, mortality, disability, and associated trends are essential for evidence-based health policy development and resource allocation. Such data can also facilitate the identification of populations that are most likely to benefit from targeted preventive and therapeutic interventions.

The Global Burden of Diseases, Injuries, and Risk Factors (GBD) study systematically quantifies global health losses attributable to diseases, injuries, and various risk factors, spanning 1990 through 2021 (5, 6). As a continuously updated, peer-reviewed, and comprehensive resource, the GBD provides detailed epidemiologic insights into stroke burden across demographic and geographic subgroups, including distinctions by age, sex, and US states. Recent evidence indicates an increasing stroke burden across the entire age spectrum, with a notable increasing incidence among younger individuals. Despite this, national-level trends specific to the US population have not been thoroughly explored (7, 8). Therefore, we investigated the burden of stroke, including ischemic and hemorrhagic strokes, in the US, stratified by age, sex, and state from the 2021 GBD data.

## 2 Methods

### 2.1 Overview and data source

The GBD study is a comprehensive and methodological analysis of global diseases. Using the estimates from this study, the present global, regional, and national disease burdens can be compared and assessed (9). The research adheres to the Guidelines for Accurate and Transparent Health Estimates Reporting (GATHER) (10). The research, conducted by the Institute for Health Metrics and Evaluation (IHME) at the University of Washington, utilizes anonymized data, which eliminates the requirement for informed consent. The stroke data analyzed in this study were sourced from GBD 2021, which offers the most recent epidemiological estimates of 371 diseases and injuries across 21 GBD regions and 204 countries and territories from 1990 to 2021. All data are freely available at the Global Health Data Exchange^1^ (11), with comprehensive information on the data, methodologies, and statistical modeling available in previous reports (5, 6). The relevant data were anonymous and publicly accessible. Our Institutional Review Board approved the waiver of informed consent for this study (approval number: EUMC 2025-01-05). This manuscript was produced as a part of the GBD Collaborator Network and is in accordance with the GBD Protocol.

### 2.2 Case definition

We assessed the overall stroke burden encompassing both total stroke events and specific stroke subtypes, including ischemic stroke, intracerebral hemorrhage (ICH), and subarachnoid hemorrhage (SAH). Stroke cases within the GBD framework were identified according to the World Health Organization (WHO) criteria as acute neurological deficits of vascular origin lasting >24 h or resulting in death, confirmed through clinical diagnosis and neuroimaging. Incident stroke was classified as the first clinically diagnosed stroke event, as determined by physician assessment and imaging, which is consistent with WHO standards. Ischemic stroke includes vascular occlusive events that cause cerebral infarction and excludes transient ischemic attacks. Hemorrhagic strokes comprise spontaneous (nontraumatic) events categorized as ICH, defined by intraparenchymal bleeding, and SAH, characterized by bleeding within the subarachnoid space, both verified through neuroimaging (4, 6). Case ascertainment for ischemic and hemorrhagic strokes utilized validated International Classification of Diseases (ICD-9 and ICD-10) diagnostic codes (Supplementary methods), with stroke burden estimates reflecting only the first-ever stroke occurrence in an individual’s lifetime.

### 2.3 Input data and estimates

For the GBD study, systematic reviews from various data sources were analyzed along with data from the National Health Interview Survey, the National Health and Nutrition Examination Survey in the US, health system administrative data such as the Healthcare Cost and Utilization Project (HCUP), and additional national claims data (12). Studies or datasets with small sample sizes (<150 participants), review articles, non-population-based studies, or those lacking clear subpopulation definitions were excluded by the IHME. Methods for gathering data on nonfatal outcomes and mortality have been detailed in previous studies (5, 11). Fatal estimates were obtained from vital registration and surveillance data. Detailed search terms for the systematic analysis are demonstrated in Supplementary methods (5, 11). To investigate the burden of stroke, prevalence, incidence, disability-adjusted life years (DALYs), years of life lost (YLLs), years lived with disability (YLDs), and mortality were estimated. DALYs are a measure of health loss in a population. One DALY represents the loss of 1 year of full health. DALYs were calculated by adding YLLs to YLDs. Detailed methods for determining the risk factors for GBD are demonstrated in Supplementary methods.

### 2.4 Statistical analysis

Bayesian meta-regression methods were implemented in the MR-BRT software (IHME) to systematically account for known methodological biases across studies. Adjustments were specifically applied to studies derived exclusively from administrative data sources, such as the HCUP, those combining first-ever and recurrent stroke events, studies reporting only aggregated data for stroke subtypes, and studies in which prehospital stroke deaths were omitted from stroke definitions or measurement protocols (13, 14). DisMod-MR, an epidemiologic state-transition modeling software, was used to estimate the stroke incidence and prevalence. Stroke mortality, including subtype-specific mortality, was derived from vital registration data classified using ICD coding standards. Although verbal autopsy data are generally incorporated into the GBD methodology for aggregated stroke mortality estimation, these data were excluded from subtype-specific estimates because of limited stroke-related medical history details. In the specific context of the US, verbal autopsy data are omitted entirely because of the availability of detailed vital registration records. Statistical modeling methods have been employed to improve comparability among diverse mortality data sources. Population-attributable fractions were independently computed for each risk factor based on exposure levels, relative risk values derived from meta-analyses, and theoretical minimum risk benchmarks tailored for each specific risk-outcome combination. To address comorbidities, simulations involving 40,000 hypothetical individuals per demographic subgroup (defined by age, sex, country, and year) were performed, assigning each individual a probability of developing conditions reflective of the observed prevalence rates. The reported 95% uncertainty intervals (UIs) for all estimates were derived from 1,000 posterior draws from the Bayesian models and expressed as intervals spanning the 2.5th to 97.5th percentiles of the resulting distributions (13, 14).

## 3 Results

### 3.1 Burden of stroke in the US in 2021

In 2021, the US experienced approximately 0.41 million incident strokes (95% UI, 0.36–0.47), of which ischemic strokes accounted for 0.31 million cases (75.6%; 95% UI, 0.26–0.37). The incidence of ICH and SAH were 0.07 million (95% UI, 0.06–0.08) and 0.03 million (95% UI, 0.03–0.03), respectively. The number of prevalent stroke cases was 6.3 million (95% UI, 5.89–6.76), with 3.07 million occurring among men (48.7%; 95% UI, 2.84–3.46) and 3.23 million among women (51.3%; 95% UI, 3.04–3.46). Prevalent cases of ICH and SAH totaled 0.75 million (95% UI, 0.68–0.84) and 0.45 million (95% UI, 0.41–0.50), respectively. Stroke accounted for approximately 0.19 million deaths (95% UI, 0.16–0.21), comprising 0.08 million deaths among men (95% UI, 0.07–0.09) and 0.11 million among women (95% UI, 0.09–0.12). DALYs lost owing to stroke amounted to 3.91 million (95% UI, 3.53–4.23), with 1.84 million DALYs among men (95% UI, 1.71–1.97) and 2.07 million among women (95% UI, 1.81–2.25) (Table 1).

**Table 1:** Absolute count of incidence, prevalence, mortality, DALYs, YLDs, and YLLs for all stroke in the US in 1990 and 2021, with percentage changes from 1990 to 2021.

Disease		All stroke			Ischemic stroke			Intracerebral hemorrhage			Subarachnoid hemorrhage		
Sex		Both	Women	Men	Both	Women	Men	Both	Women	Men	Both	Women	Men
Incidence	1990	357880.78 (303318.85 to 420067.62)	191785.43 (161799.72 to 224432.25)	166095.35 (139308.18 to 197467.05)	282791.3 (230222.1 to 345341.01)	151205.51 (123419.73 to 185024.23)	131585.78 (105832.44 to 161437.24)	55214.38 (45721.51 to 64379.8)	27726.38 (22837.4 to 32460.03)	27487.99 (22696.64 to 32132.14)	19875.11 (17051.66 to 23439.56)	12853.53 (10953.82 to 15231.59)	7021.58 (6054.48 to 8160.91)
	2021	412025.08 (358281.25 to 469611.65)	214738.83 (186708.7 to 245843.62)	197286.25 (170381.28 to 227750.92)	311586.81 (259142.17 to 370269.01)	163459.96 (136542.74 to 194583.57)	148126.85 (123426.56 to 177739.82)	70680.43 (60481.51 to 80920.63)	33828.48 (28701.67 to 38969.37)	36851.95 (31261.94 to 42109.92)	29757.84 (26203.88 to 34,291)	17450.38 (15245.1 to 20223.94)	12307.46 (10763.88 to 14120.81)
	change, %	15.13 (9.29 to 21.7)	11.97 (6.16 to 18.64)	18.78 (12.81 to 25.98)	10.18 (3.78 to 17.9)	8.1 (1.41 to 16.27)	12.57 (6.07 to 20.72)	28.01 (20.77 to 36.94)	22.01 (14.35 to 31.51)	34.07 (25.76 to 43.8)	49.72 (42.2 to 60.09)	35.76 (28.84 to 45.03)	75.28 (64.71 to 88.11)
Prevalence	1990	3767724.96 (3478373.61 to 4094434.4)	2025748.2 (1874703.51 to 2201876.32)	1741976.76 (1591942.03 to 1900771.75)	3100388.69 (2812947.11 to 3417383.47)	1644095.15 (1485199.78 to 1809484.55)	1456293.54 (1305430.97 to 1608008.69)	422557.29 (376060.02 to 472546.52)	214249.8 (190926.02 to 238980.4)	208307.48 (186276.63 to 233329.31)	266333.69 (236589.39 to 301773.08)	179595.76 (159445.45 to 203222.34)	86737.93 (76570.71 to 98947.34)
	2021	6299339.3 (5888661.22 to 6760944.55)	3232424.43 (3036402.7 to 3464277.63)	3066914.87 (2844033.84 to 3302190.86)	5136253.51 (4700312.21 to 5584576.89)	2593410.2 (2382400.64 to 2819656.69)	2542843.31 (2315511.3 to 2773023.71)	753476.61 (675190.65 to 838583.41)	362074.76 (324967.21 to 402909.09)	391401.85 (348210.83 to 436576.37)	454302.24 (409929.63 to 503031.48)	300368.07 (270652.77 to 332379.1)	153934.18 (139101.97 to 171093.99)
	change, %	67.19 (60.06 to 74.25)	59.57 (52.69 to 66.23)	76.06 (67.9 to 85.18)	65.66 (57.32 to 74.33)	57.74 (49.12 to 66.6)	74.61 (65.24 to 85.26)	78.31 (68.53 to 87.17)	69 (60.24 to 77.4)	87.9 (75.7 to 98.4)	70.58 (62.72 to 78.91)	67.25 (58.74 to 75.83)	77.47 (68.75 to 86.17)
Mortality	1990	146671.76 (130254.15 to 155463.74)	87685.26 (74813.42 to 94296.58)	58986.5 (55235.8 to 61156.73)	99528.86 (86391.96 to 106294.34)	61905.4 (51495.51 to 67250.79)	37623.45 (34599.45 to 39211.46)	35858.83 (33193.46 to 37340.04)	18409.03 (16442.37 to 19434.34)	17449.8 (16663.91 to 17985.49)	11284.07 (10654.72 to 11674.85)	7370.82 (6852.25 to 7691.24)	3913.25 (3787.68 to 4020.67)
	2021	191770.26 (162653.89 to 207055.2)	111047.36 (89916.82 to 122313.76)	80722.9 (72817.69 to 85544.55)	115555.16 (94590.2 to 126652.19)	72036.09 (56387.41 to 80401.59)	43519.07 (38151.49 to 46450.19)	58170.57 (52025.59 to 61737.07)	28437.58 (23972.96 to 30707.85)	29732.99 (27513.66 to 31255.91)	18044.53 (16291.84 to 19093.2)	10573.69 (9288.51 to 11332.28)	7470.84 (6947.25 to 7859.94)
	change, %	30.75 (24.05 to 35.41)	26.64 (19.14 to 31.62)	36.85 (29.08 to 42.44)	16.1 (9.02 to 20.34)	16.36 (9.13 to 21.1)	15.67 (8.24 to 20.51)	62.22 (53.51 to 68.64)	54.48 (45 to 61.74)	70.39 (61.25 to 77.54)	59.91 (50.98 to 66.93)	43.45 (34.45 to 50.21)	90.91 (79.38 to 99.85)
DALYs	1990	3055726.78 (2814110.94 to 3253875.95)	1688027.03 (1525022.47 to 1814885.96)	1367699.75 (1291669.65 to 1446572.87)	1830268.29 (1644784.36 to 1989853.88)	1046787.42 (920446.26 to 1149009.15)	783480.87 (723786.03 to 845225.08)	850497.77 (806832.08 to 881552.59)	406380.38 (378616.04 to 426288.97)	444117.39 (429253.72 to 458637.5)	374960.72 (360591.57 to 389551.15)	234859.24 (224595.77 to 245333.92)	140101.49 (135575.66 to 145005.11)
	2021	3911878.48 (3533574.54 to 4227869.76)	2070936.43 (1810643.56 to 2253918.14)	1840942.05 (1706822.85 to 1971872.5)	2178054.33 (1909480.33 to 2407924.58)	1228053.91 (1,044,360 to 1371619.6)	950000.42 (847017.65 to 1048119.21)	1252562.69 (1164767.98 to 1317343.2)	564971.11 (507838.85 to 600837.66)	687591.59 (651325.07 to 722407.04)	481261.46 (452792.89 to 508845.19)	277911.41 (257859.64 to 295747.98)	203350.05 (192684.85 to 214164.26)
	change, %	28.02 (23.24 to 31.74)	22.68 (17.94 to 26.21)	34.6 (29.3 to 39.29)	19 (14.4 to 22.72)	17.32 (12.78 to 21.17)	21.25 (15.5 to 25.62)	47.27 (40.63 to 52.74)	39.03 (32.31 to 44.59)	54.82 (47.6 to 61.31)	28.35 (22.63 to 33.13)	18.33 (12.36 to 22.9)	45.14 (37.75 to 51.93)
YLDs	1990	536,878 (381323.24 to 687481.73)	308487.72 (218932.01 to 396403.21)	228390.28 (164125.63 to 294058.97)	438406.79 (311557.63 to 565663.62)	248718.8 (175728.15 to 320417.02)	189687.99 (137113.96 to 243514.97)	60271.98 (42336.23 to 78615.83)	32999.98 (23417.93 to 43228.72)	27,272 (19056.68 to 35444.62)	38199.23 (27207.01 to 49585.44)	26768.95 (19297.66 to 34738.89)	11430.28 (8055.65 to 14983.44)
	2021	882003.9 (641272.79 to 1124842.09)	482002.92 (350182.52 to 617023.71)	400000.98 (288680.64 to 508285.39)	711180.89 (514467.36 to 902154.84)	382934.53 (277332.77 to 489435.7)	328246.36 (237618.42 to 417585.82)	106794.3 (77800.79 to 136938.6)	55282.9 (40183.83 to 71422.76)	51511.41 (37152.18 to 65784.72)	64028.71 (45348.6 to 81978.06)	43785.5 (31047.86 to 55954.73)	20243.21 (14418.02 to 25914.94)
	change, %	64.28 (56.65 to 72.11)	56.25 (48.92 to 63.65)	75.14 (65.73 to 85.5)	62.22 (53.36 to 71.18)	53.96 (45.13 to 62.42)	73.05 (62.4 to 84.55)	77.19 (66.82 to 87.76)	67.52 (57.55 to 77.28)	88.88 (76.15 to 101.8)	67.62 (57.86 to 78.42)	63.57 (52.8 to 75.29)	77.1 (66.77 to 87.97)
YLLs	1990	2518848.78 (2321268.35 to 2629038.1)	1379539.31 (1228446.22 to 1459027.72)	1139309.47 (1086553.69 to 1173409.02)	1391861.5 (1243724.84 to 1470862.61)	798068.62 (684949.05 to 856577.52)	593792.88 (556648.84 to 615,494)	790225.79 (751015.45 to 815001.12)	373380.4 (346489.34 to 389192.06)	416845.39 (403030.78 to 428530.24)	336761.49 (325310.28 to 345673.54)	208090.29 (199707.6 to 214633.5)	128671.2 (124846.58 to 132228.45)
	2021	3029874.58 (2714069.37 to 3213844.93)	1588933.5 (1355551.57 to 1711520.05)	1440941.08 (1336768.09 to 1514902.26)	1466873.44 (1246162.69 to 1581036.97)	845119.38 (685748.46 to 928927.6)	621754.06 (559756.85 to 657725.57)	1145768.39 (1059582.28 to 1203207.01)	509688.21 (453402.39 to 540891.55)	636080.18 (601896.35 to 667474.08)	417232.75 (391137.01 to 436538.44)	234125.91 (216618.51 to 247330.24)	183106.84 (173338.48 to 191783.64)
	change, %	20.29 (14.66 to 24.57)	15.18 (9 to 19.81)	26.47 (19.99 to 31.95)	5.39 (−0.26 to 9.17)	5.9 (−0.48 to 10.1)	4.71 (−1.2 to 9.11)	44.99 (38.11 to 50.72)	36.51 (29.16 to 42.62)	52.59 (44.72 to 59.34)	23.9 (17.45 to 29.06)	12.51 (6.33 to 17.37)	42.31 (34.15 to 49.75)

DALYs, disability-adjusted life years; YLLs, years of life lost; YLDs, years lived with disability.

### 3.2 Trends in the burden of stroke from 1990 to 2021

From 1990 to 2021, the burden of stroke in the US, as measured by the absolute number of incident cases, prevalent cases, DALYs, YLDs, and YLLs, showed an overall increasing trend (Table 1 and Supplementary Figure S1). However, the numbers of stroke incidents, deaths, DALYs, and YLLs exhibited a transient decline during the intermediate period within this interval. For nearly all evaluated measures, including total stroke, ischemic stroke, ICH, and SAH, the magnitude of the increase was consistently higher in men than in women, except for YLL, which was attributed to ischemic stroke. Specifically, between 1990 and 2021, the ischemic stroke incidence increased by 10.18% (95% UI, 3.78–17.9%), while the incidence of ICH and SAH increased by 28.01% (95% UI, 20.77–36.94%) and 49.72% (95% UI, 42.2–60.09%), respectively. The stroke prevalence exhibited substantial growth, with the prevalence of ischemic stroke increasing by 65.66% (95% UI, 57.32–74.33%), ICH by 78.31% (95% UI, 68.53–87.17%), and SAH by 70.58% (95% UI, 62.72–78.91%). Stroke-related mortality increased by 16.1% (95% UI, 9.02–20.34%) for ischemic stroke, while mortality due to ICH and SAH increased significantly by 62.22% (95% UI, 53.51–68.64%) and 59.91% (95% UI, 50.98–66.93%), respectively (Table 1).

### 3.3 Trends in the age-standardized burden of stroke from 1990 to 2021

**Figure 1:**
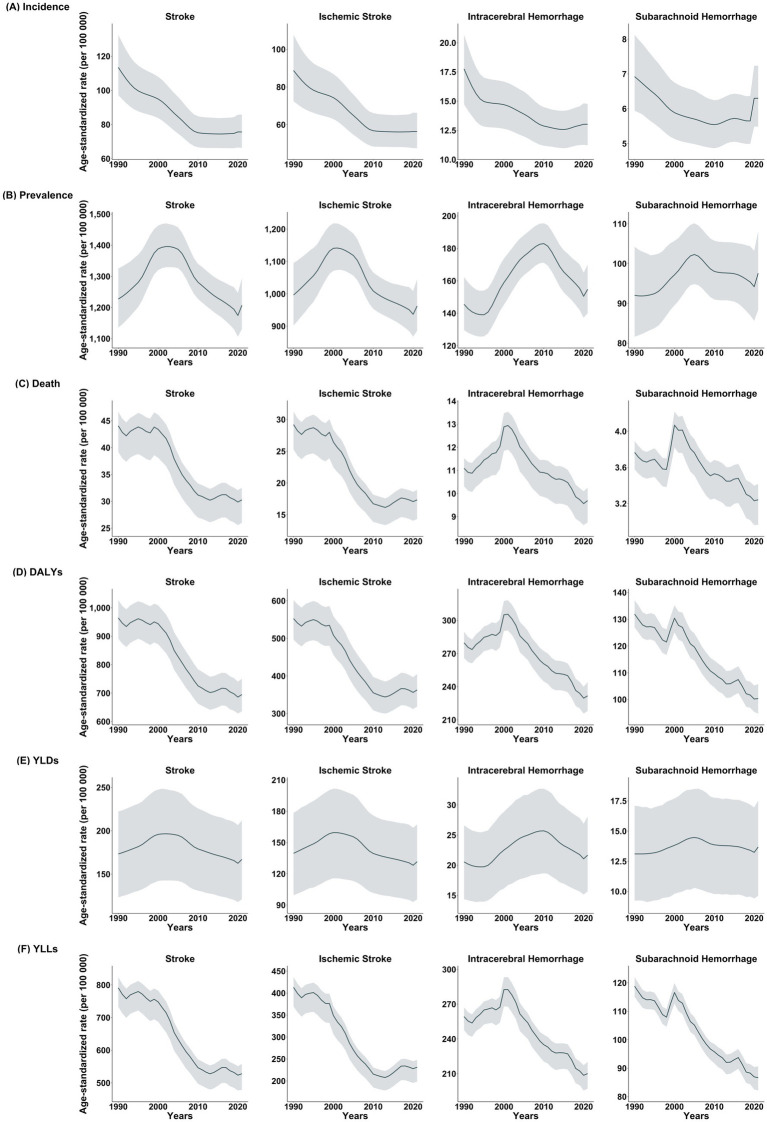
Trend of each stroke type in **(A)** incidence, **(B)** prevalence, **(C)** death, **(D)** DALYs, **(E)** YLDs, and **(F)** YLLs (1990–2021, US).

From 1990 to 2021, the age-standardized incidence, prevalence, mortality, DALYs, YLDs, and YLLs of stroke decreased or remained stable, with changes typically greater in men than in women (Table 2). Considerable declines were observed in overall stroke incidence, mortality, DALYs, and YLLs, whereas changes in the prevalence and YLDs were relatively minor. Notably, the prevalence exhibited varying trends across stroke subtypes during this period (Figure 1). Specifically, ischemic stroke prevalence declined by 3.44% (95% UI, −8.02 to 1.19%), whereas ICH and SAH prevalence increased by 6.34% (95% UI, 1.80–10.77%) and 6.03% (95% UI, 1.06–11.19%), respectively. Additionally, the reductions in the incidence and mortality were more pronounced for ischemic stroke than for hemorrhagic stroke (Table 2). While the incidence of ischemic stroke and ICH generally decreased from 1990 to 2021, the incidence of SAH demonstrated an increasing trend in recent years. Mortality, DALYs, and YLLs associated with hemorrhagic stroke peaked around 2000 (Figure 1 and Supplementary Figure S3).

**Table 2:** Age-standardized rate of incidence, prevalence, mortality, DALYs, YLDs, and YLLs for all stroke in the US in 1990 and 2021, with percentage changes from 1990 to 2021.

Disease		All stroke			Ischemic stroke			Intracerebral hemorrhage			Subarachnoid hemorrhage		
Sex		Both	Women	Men	Both	Women	Men	Both	Women	Men	Both	Women	Men
Incidence	1990	113.45 (97.15 to 132.48)	102.85 (88.58 to 119.4)	126.68 (107.62 to 149.56)	88.76 (72.46 to 107.66)	79.37 (65.48 to 96.09)	99.96 (80.76 to 121.99)	17.76 (14.72 to 20.67)	15.15 (12.52 to 17.76)	21.31 (17.62 to 24.93)	6.92 (5.94 to 8.12)	8.33 (7.09 to 9.81)	5.41 (4.66 to 6.29)
	2021	75.64 (66.28 to 85.84)	70.77 (62.58 to 80.14)	80.72 (70.82 to 92.08)	56.32 (47.48 to 66.31)	52.6 (44.33 to 61.7)	59.96 (50.84 to 70.95)	13.02 (11.22 to 14.76)	11.13 (9.57 to 12.68)	15.18 (13.05 to 17.26)	6.3 (5.49 to 7.24)	7.04 (6.12 to 8.17)	5.59 (4.88 to 6.39)
	change, %	−33.32 (−36.41 to −29.96)	−31.19 (−34.37 to −27.88)	−36.28 (−39.6 to −32.85)	−36.55 (−40 to −32.6)	−33.72 (−37.37 to −29.61)	−40.02 (−43.75 to −36.02)	−26.68 (−30.81 to −21.75)	−26.53 (−30.84 to −21.27)	−28.79 (−32.79 to −24.01)	−8.99 (−12.73 to −3.86)	−15.57 (−19.06 to −10.78)	3.36 (−1.02 to 9.03)
Prevalence	1990	1227.3 (1135.57 to 1325.17)	1166.38 (1079.42 to 1254.06)	1315.22 (1202.14 to 1431.9)	996.34 (901.9 to 1095.45)	921.82 (836.41 to 1011.41)	1098.18 (986.33 to 1207.19)	145.51 (129.59 to 162.29)	135.89 (120.38 to 151.56)	158.73 (141.96 to 177.13)	92.02 (81.63 to 104.22)	114.87 (102.43 to 129.64)	65.5 (57.94 to 74.53)
	2021	1206.97 (1130.76 to 1293.35)	1160.56 (1089.43 to 1242.41)	1264.3 (1177.91 to 1358.13)	962.1 (885.76 to 1044.72)	903.35 (833 to 982.44)	1031.5 (944.81 to 1122.74)	154.73 (140.07 to 169.92)	142.14 (127.91 to 156.48)	169.79 (152.86 to 187.16)	97.57 (88.38 to 108.09)	122.08 (110.43 to 134.75)	71.03 (64.31 to 79.1)
	change, %	−1.66 (−5.53 to 2.21)	−0.5 (−4.28 to 3.43)	−3.87 (−8.31 to 0.91)	−3.44 (−8.02 to 1.19)	−2 (−6.66 to 2.91)	−6.07 (−11.21 to −0.24)	6.34 (1.8 to 10.77)	4.6 (0.5 to 9)	6.97 (1.75 to 11.92)	6.03 (1.06 to 11.19)	6.27 (0.89 to 11.75)	8.44 (3.5 to 13.54)
Mortality	1990	44.05 (39.19 to 46.66)	41.13 (35.57 to 43.98)	47.83 (44.21 to 49.76)	29.2 (25.28 to 31.21)	27.3 (22.86 to 29.58)	31.6 (28.63 to 33.11)	11.09 (10.3 to 11.53)	9.47 (8.59 to 9.94)	13.24 (12.6 to 13.66)	3.77 (3.58 to 3.89)	4.36 (4.12 to 4.52)	3 (2.89 to 3.08)
	2021	30.27 (26.01 to 32.51)	28.94 (23.94 to 31.56)	31.21 (28.08 to 33.1)	17.33 (14.33 to 18.92)	17.35 (13.84 to 19.23)	16.77 (14.64 to 17.94)	9.69 (8.75 to 10.25)	8.19 (7.08 to 8.77)	11.37 (10.53 to 11.98)	3.24 (2.96 to 3.42)	3.4 (3.07 to 3.61)	3.06 (2.85 to 3.23)
	change, %	−31.3 (−34.25 to −29.08)	−29.62 (−33.03 to −26.96)	−34.76 (−37.96 to −32.3)	−40.63 (−43.59 to −38.66)	−36.42 (−39.75 to −33.98)	−46.93 (−49.99 to −44.88)	−12.61 (−16.88 to −9.23)	−13.43 (−18.37 to −9.62)	−14.08 (−18.5 to −10.54)	−13.94 (−18.27 to −10.31)	−22.18 (−26.65 to −18.81)	2.25 (−3.78 to 7.08)
DALYs	1990	964.29 (891.53 to 1026.45)	897.51 (817.82 to 963.39)	1051.28 (989.69 to 1111.5)	552.57 (496.57 to 601.36)	507.33 (447.99 to 558.05)	611.93 (562.8 to 658.78)	279.79 (265.86 to 289.67)	236.26 (222.52 to 247.08)	332.95 (321.2 to 344.04)	131.92 (126.95 to 137.11)	153.93 (147.44 to 160.49)	106.4 (102.91 to 110.11)
	2021	694.92 (635.82 to 750.45)	650.72 (584.93 to 709.52)	739.32 (685.42 to 793.19)	362.81 (318.36 to 404.82)	352.13 (304.94 to 395.04)	370.43 (328.77 to 409.21)	231.76 (218.11 to 244.14)	190.41 (175.07 to 201.74)	277.14 (262.68 to 291.49)	100.36 (94.75 to 105.76)	108.18 (101.61 to 115.02)	91.75 (87.03 to 96.63)
	change, %	−27.93 (−30.32 to −25.88)	−27.5 (−30.26 to −25.41)	−29.67 (−32.42 to −27.29)	−34.34 (−36.89 to −32.14)	−30.59 (−33.34 to −28.25)	−39.47 (−42.14 to −37.12)	−17.17 (−20.67 to −14.14)	−19.41 (−23.09 to −16.3)	−16.76 (−20.5 to −13.23)	−23.92 (−27.05 to −21.07)	−29.72 (−33 to −26.69)	−13.77 (−18.1 to −9.74)
YLDs	1990	173.29 (123.22 to 222.41)	174.38 (124.16 to 223.59)	174.15 (124.92 to 223.65)	139.64 (99.57 to 178.49)	136.89 (97.52 to 175.81)	144.61 (104.3 to 185.56)	20.55 (14.36 to 26.6)	20.56 (14.49 to 26.81)	20.9 (14.67 to 27.11)	13.11 (9.24 to 17.1)	16.93 (12.02 to 22.02)	8.65 (6.09 to 11.34)
	2021	166.93 (121.03 to 211.9)	169.93 (123.71 to 216.45)	164.5 (118.54 to 209.37)	131.59 (95.5 to 167.31)	130.9 (95.1 to 167.35)	132.93 (96.36 to 168.59)	21.66 (15.66 to 28.08)	21.33 (15.37 to 27.94)	22.24 (16.05 to 28.46)	13.68 (9.63 to 17.51)	17.7 (12.46 to 22.76)	9.32 (6.55 to 11.94)
	change, %	−3.67 (−7.74 to 0.55)	−2.55 (−6.78 to 1.63)	−5.54 (−10.36 to −0.42)	−5.77 (−10.65 to −0.79)	−4.38 (−9.41 to 0.68)	−8.07 (−13.6 to −2.3)	5.42 (0.53 to 10.52)	3.78 (−1.87 to 9.2)	6.45 (0.46 to 12.44)	4.38 (−1.88 to 11.06)	4.54 (−2.48 to 12.49)	7.76 (1.88 to 13.65)
YLLs	1990	791 (732.81 to 823.85)	723.13 (655.38 to 759.36)	877.13 (831.21 to 905.01)	412.93 (369.67 to 436.28)	370.44 (321.5 to 395.77)	467.33 (433.59 to 485.58)	259.25 (247.52 to 267.16)	215.7 (202.94 to 223.58)	312.06 (301.32 to 320.92)	118.81 (115.05 to 122)	136.99 (132.3 to 141.11)	97.75 (94.82 to 100.42)
	2021	528 (478.49 to 557.76)	480.79 (422.94 to 512.48)	574.82 (533.69 to 604.71)	231.22 (198.61 to 248.09)	221.23 (182.83 to 241.28)	237.5 (213.29 to 251.3)	210.1 (197.03 to 220.23)	169.08 (153.46 to 178.18)	254.89 (241.48 to 267.84)	86.68 (82.18 to 90.61)	90.48 (85.08 to 95.05)	82.42 (77.87 to 86.43)
	change, %	−33.25 (−35.93 to −30.88)	−33.51 (−36.51 to −31.07)	−34.47 (−37.46 to −31.7)	−44.01 (−46.63 to −42.1)	−40.28 (−43.44 to −37.9)	−49.18 (−51.8 to −47.14)	−18.96 (−22.59 to −15.8)	−21.62 (−25.54 to −18.14)	−18.32 (−22.33 to −14.6)	−27.05 (−30.43 to −23.94)	−33.95 (−37.33 to −30.97)	−15.68 (−20.51 to −11.27)

DALYs, Disability-adjusted life years; YLLs, years of life lost; YLDs, years lived with disability.

### 3.4 Burden of stroke by age group and geographic location

**Figure 2:**
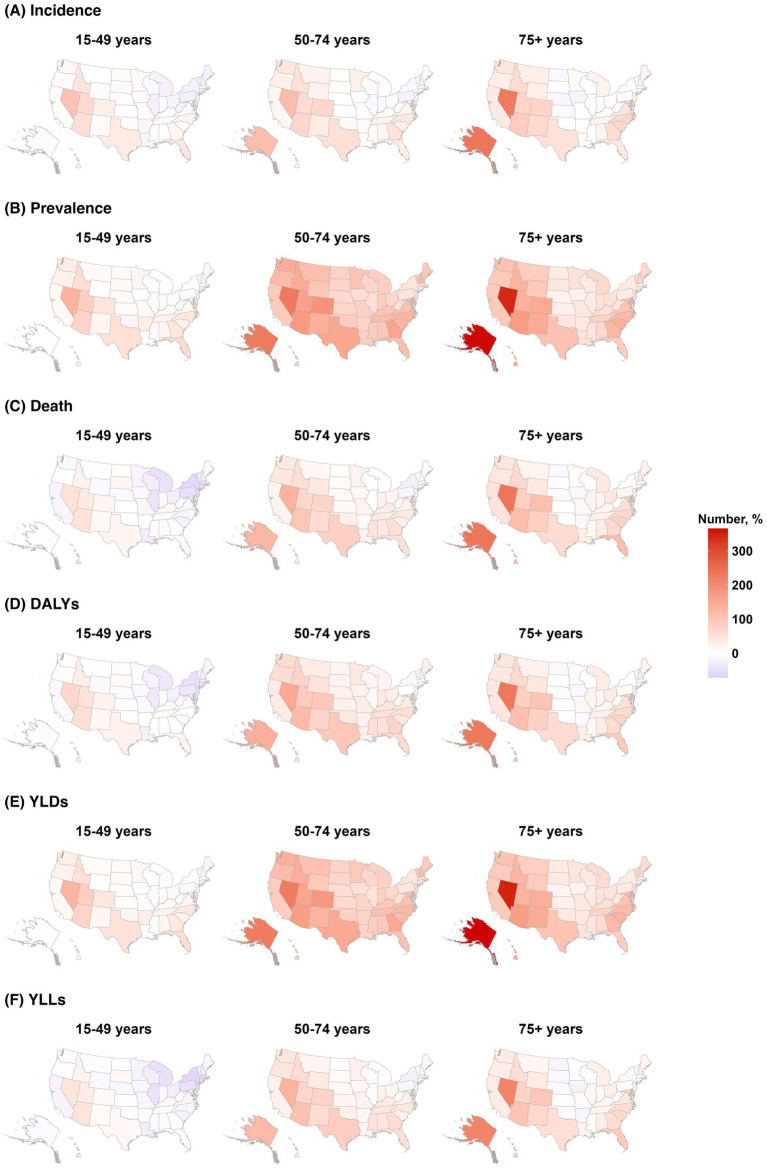
Change in burden of stroke by age groups of 15–49, 50–74, and ≥75 years in **(A)** incidence, **(B)** prevalence, **(C)** death, **(D)** DALYs, **(E)** YLDs, and **(F)** YLLs (1990–2021, US).

The percentage change in age-standardized DALYs due to stroke ranged from −11.72% (95% UI, −22.31 to 0.24%) in Mississippi to −48.86% (95% UI, −54.91 to 42.54%) in the District of Columbia (Supplementary Tables S1, S2). The stroke prevalence decreased most substantially in individuals aged ≥75 years (−2.76%; 95% UI, −9.06 to 4.79%), followed by the age group of 50–74 years (−0.29%; 95% UI, −5.60 to 5.51%), whereas the prevalence increased by 2.46% (95% UI, −1.58 to 6.43%) among those aged 15–49 years. Alaska and Nevada experienced the greatest increases across all stroke measures. The number of incident cases, deaths, and DALYs decreased in most eastern US states in the youngest age group (15–49 years). Arkansas showed the highest increase in age-standardized prevalence and YLD rates among those aged 15–49 years, whereas the District of Columbia experienced the largest reductions in death and DALYs for the same age group (Figure 2). The trends in each stroke subtype according to geographic location are described in Supplementary Tables S3, S4.

### 3.5 Temporal trends and risk factor impact on stroke subtypes (1990–2021)

Among the various risk factors, metabolic risks accounted for the largest proportion of the stroke burden, followed by behavioral and environmental/occupational risks (Supplementary Figures S4, S5). Deaths attributed to household air pollution from solid fuels showed the largest decrease, declining by 87.13% (95% UI, −99.99 to 90.15%), while high temperature demonstrated the greatest increase of 10.10% (95% UI, −146.43 to 148.37%) (Supplementary Table S6). The DALYs associated with high systolic blood pressure declined by 43.71% (95% UI, −49.17 to 39.30%) for ischemic stroke, 27.35% (95% UI, −33.89 to 21.36%) for ICH, and 29.14% (95% UI, −35.27 to 22.86%) for SAH (Table 3). The age-standardized rates and percentage changes in stroke are described in Supplementary Tables S7, S8.

**Table 3:** Age-standardized rate and percentage change of mortality and DALYs for stroke over time by risk factors, both sexes, US, 1990–2021.

Disease	Risk factor	Death			DALYs		
		1990	2021	Change, %	1990	2021	Change, %
Stroke	High systolic blood pressure	26.38 (19.81 to 32.08)	15.77 (11.12 to 20.21)	−40.22 (−46.4 to −35.03)	536.58 (402.07 to 650.51)	335.94 (241.27 to 424.52)	−37.39 (−42.77 to −32.86)
	High fasting plasma glucose	5.68 (4.45 to 7.04)	5.14 (4.02 to 6.34)	−9.49 (−16.94 to −0.83)	107.74 (83.88 to 132.1)	104.46 (82.29 to 126.35)	−3.05 (−10.13 to 4.97)
	High LDL cholesterol	8.89 (2.8 to 15.06)	4.11 (1.19 to 7.34)	−53.79 (−57.67 to −50.87)	189.56 (68.43 to 303.9)	105.08 (36.77 to 172.19)	−44.57 (−48.48 to −41.32)
	High body-mass index	2.4 (0.18 to 5.22)	2.35 (0.14 to 4.79)	−2.07 (−16.87 to 10.07)	72.65 (4.47 to 153.46)	77.6 (4.45 to 151.21)	6.8 (−8.02 to 23.02)
	Dietary risks	2.95 (0.06 to 6.86)	2.14 (−0.07 to 5.09)	−27.43 (−72.09 to 3.98)	69.88 (−8.02 to 159.67)	62.89 (−0.75 to 133.26)	−10 (−50.93 to 46)
	Low physical activity	0.89 (−0.67 to 2.59)	0.53 (−0.52 to 1.58)	−40.63 (−96.77 to 17.27)	20.69 (−0.14 to 45.31)	15.04 (0.79 to 32.82)	−27.29 (−54.79 to 7.16)
	Tobacco	6.35 (5.27 to 7.69)	3.15 (2.52 to 3.95)	−50.4 (−54.51 to −45.75)	194.01 (164.52 to 230.04)	103.93 (85.2 to 126.66)	−46.43 (−49.88 to −42.91)
	Alcohol use	1.97 (0.31 to 5.18)	1.99 (0.37 to 4.26)	0.97 (−28.94 to 71.5)	48.32 (9.33 to 117.88)	48.87 (9.61 to 101.19)	1.14 (−21.46 to 51.92)
	Air pollution	4.26 (1.67 to 7.55)	0.99 (0.49 to 1.62)	−76.83 (−90.05 to −47.84)	90.8 (36.15 to 156.06)	22.13 (10.95 to 35.66)	−75.63 (−89.64 to −45.64)
Ischemic stroke	High systolic blood pressure	17.85 (13.35 to 21.68)	9.28 (6.46 to 11.91)	−47.98 (−54.56 to −42.43)	322.59 (240.9 to 391.42)	181.59 (129.21 to 233.07)	−43.71 (−49.17 to −39.3)
	High fasting plasma glucose	4.97 (3.75 to 6.22)	4.36 (3.21 to 5.33)	−12.28 (−19.75 to −3.86)	91.24 (70.01 to 114.66)	86.66 (66.94 to 106.19)	−5.02 (−12.02 to 3.07)
	High LDL cholesterol	8.89 (2.8 to 15.06)	4.11 (1.19 to 7.34)	−53.79 (−57.67 to −50.87)	189.56 (68.43 to 303.9)	105.08 (36.77 to 172.19)	−44.57 (−48.48 to −41.32)
	High body-mass index	1.53 (0.22 to 3.14)	1.3 (0.19 to 2.72)	−14.82 (−25.58 to −5.6)	41.12 (6.04 to 81.01)	41.25 (6.24 to 76.92)	0.32 (−11.93 to 12.15)
	Dietary risks	2.82 (0.32 to 5.92)	1.77 (0.22 to 3.63)	−37.13 (−48.71 to −19.13)	71.83 (2.53 to 134.77)	56.63 (5.4 to 102.33)	−21.16 (−29.68 to 2.05)
	Low physical activity	0.89 (−0.67 to 2.59)	0.53 (−0.52 to 1.58)	−40.63 (−96.77 to 17.27)	20.69 (−0.14 to 45.31)	15.04 (0.79 to 32.82)	−27.29 (−54.79 to 7.16)
	Tobacco	3.17 (2.5 to 4.01)	1.31 (0.98 to 1.72)	−58.57 (−63.5 to −52.74)	85.73 (70.45 to 105.48)	43.49 (34.19 to 55.61)	−49.27 (−53.4 to −45.42)
	Alcohol use	1.26 (−0.11 to 3.99)	1.17 (−0.13 to 2.99)	−7.08 (−33.94 to 80.77)	27.91 (−3.28 to 84.63)	27.44 (−3.84 to 71.56)	−1.68 (−25.51 to 64.24)
	Air pollution	2.82 (1.1 to 5)	0.56 (0.28 to 0.94)	−80.03 (−91.43 to −54.8)	52.16 (20.51 to 90.74)	11.55 (5.61 to 18.86)	−77.85 (−90.65 to −50.68)
Intracerebral hemorrhage	High systolic blood pressure	6.53 (4.94 to 7.88)	4.92 (3.5 to 6.19)	−24.77 (−31.44 to −18.78)	152.22 (113.73 to 183.69)	110.59 (76.88 to 138.97)	−27.35 (−33.89 to −21.36)
	High fasting plasma glucose	0.72 (0.39 to 1.08)	0.79 (0.43 to 1.15)	9.89 (−1.14 to 21.52)	16.51 (8.81 to 24.76)	17.8 (9.85 to 25.94)	7.86 (−2.36 to 19.52)
	High body-mass index	0.59 (−0.01 to 1.36)	0.74 (−0.03 to 1.55)	26.95 (6.57 to 76.82)	19.61 (−0.56 to 44.6)	24.04 (−1.04 to 49.57)	22.56 (2.83 to 72.9)
	Dietary risks	0.14 (−1.92 to 1.86)	0.3 (−1.35 to 1.84)	119.86 (−361.81 to 200.53)	0.05 (−68.81 to 53.5)	5.42 (−47.95 to 49.15)	10438.53 (−201.91 to 290.89)
	Tobacco	2.23 (1.89 to 2.6)	1.33 (1.1 to 1.61)	−40.43 (−44.82 to −35.88)	70.03 (60.16 to 81.41)	40.93 (33.79 to 48.67)	−41.56 (−45.45 to −37.57)
	Alcohol use	0.72 (0.03 to 1.67)	0.82 (0.03 to 1.81)	15.1 (−6.79 to 66.82)	20.41 (0.68 to 44.98)	21.43 (0.65 to 46.09)	4.98 (−10.18 to 40.14)
	Air pollution	1.08 (0.43 to 1.9)	0.32 (0.16 to 0.52)	−70.43 (−87.28 to −33.61)	26.51 (10.69 to 45.94)	7.48 (3.72 to 12.06)	−71.78 (−87.92 to −36.58)
Subarachnoid hemorrhage	High systolic blood pressure	2 (1.51 to 2.43)	1.57 (1.11 to 1.98)	−21.41 (−27.87 to −15.35)	61.77 (45.42 to 76.83)	43.77 (30.1 to 55.14)	−29.14 (−35.27 to −22.86)
	High body-mass index	0.29 (−0.01 to 0.64)	0.31 (−0.01 to 0.63)	6.59 (−8.73 to 39.66)	11.92 (−0.36 to 26.71)	12.3 (−0.55 to 25.09)	3.23 (−12.05 to 41.41)
	Dietary risks	−0.01 (−0.99 to 0.75)	0.07 (−0.61 to 0.63)	−852.37 (−189.79 to 265.02)	−2.01 (−41.91 to 30.33)	0.83 (−26.47 to 22.45)	−141.44 (−168.51 to 123.24)
	Tobacco	0.96 (0.83 to 1.1)	0.51 (0.42 to 0.61)	−46.58 (−50.61 to −42.3)	38.25 (32.82 to 44.09)	19.51 (16.32 to 23.09)	−48.99 (−52.49 to −45.53)
	Air pollution	0.36 (0.14 to 0.63)	0.1 (0.05 to 0.17)	−70.86 (−87.49 to −35.11)	12.12 (4.86 to 21.01)	3.1 (1.53 to 4.98)	−74.45 (−89.09 to −43.26)

## 4 Discussion

This systematic analysis provides a comprehensive evaluation of stroke burden in the US, emphasizing trends from 1990 to 2021 based on data from the GBD 2021 study. Although the age-standardized stroke incidence, mortality, DALYs, YLDs, and YLLs showed an overall decline or remained stable during this period, the absolute stroke burden continued to increase, particularly among younger populations and specific geographic regions. These findings highlight the critical public health challenges that require targeted interventions tailored to demographic and geographic disparities.

Significant improvements observed in age-standardized stroke incidence, mortality, and DALYs likely reflect advancements in preventive strategies, including enhanced management of cardiovascular risk factors, such as hypertension and hyperlipidemia; improved public awareness campaigns; and broader access to acute stroke interventions, including thrombolytic therapy and endovascular thrombectomy (15). However, despite these advancements, recent years have witnessed a plateau in the decline of stroke mortality and DALYs, particularly those related to hemorrhagic stroke, indicating ongoing gaps in preventive care. The persistently high prevalence of risk factors such as hypertension, obesity, diabetes mellitus, and atrial fibrillation, particularly in younger populations, further complicates this trend (15).

Notably, the rising prevalence of stroke among individuals aged 15–49 years highlights a concerning shift in stroke epidemiology. This demographic change is likely attributable to the growing prevalence of cardiometabolic risk factors, unhealthy lifestyle choices, and environmental exposure, all of which increasingly affect younger adults. Previous studies have similarly documented rising stroke incidences among younger adults, emphasizing increased obesity, diabetes, and hypertension as critical contributors (8, 16, 17). The public health implications of increasing stroke occurrence in younger populations are substantial, as these individuals face prolonged disability and greater lifetime healthcare utilization, significantly impacting healthcare resources and economic productivity (8, 16–18).

An increase in the incidence of SAH is observed not only in the United States but also as a global trend (19.20). Potential contributing factors include an aging population, changes in lifestyle, advancements in diagnostic technologies, and the possible impact of the COVID-19 pandemic since 2020 (19–21). Despite the increasing incidence and declining mortality associated with SAH, there has been no corresponding improvement in YLD, suggesting a continued lack of effective therapeutic interventions that enhance long-term functional outcomes. This stagnation stands in contrast to recent advancements in the management of ischemic stroke and ICH, where well-designed clinical trials have substantially influenced clinical practice. The relative paucity of contemporary, large-scale randomized controlled trials specifically targeting SAH represents a significant gap in the current cerebrovascular research agenda. These findings highlight an urgent need for focused research initiatives and increased funding to facilitate the development and evaluation of interventions aimed at improving the prognosis and quality of life for patients with SAH.

The regional disparities identified in our analysis underscore the complex interplay between the demographic, environmental, and socioeconomic factors that shape stroke epidemiology. States such as Alaska, Nevada, and Arkansas experienced notably larger increases in stroke prevalence, incidence, and DALYs, suggesting potential differences in healthcare accessibility, effectiveness of regional public health initiatives, and prevalence of cardiovascular risk factors. The “Stroke Belt” regions, particularly in the South and Midwest, demonstrated sustained high stroke burden, which aligns with prior research indicating socioeconomic disparities, limited healthcare resources, and higher prevalence of cardiovascular risk factors in these regions (4, 22). These geographic variations highlight the need for targeted public health initiatives that account for local epidemiologic profiles and socioeconomic determinants (23).

Metabolic risk factors, particularly hypertension, diabetes, and obesity, continue to significantly influence the stroke burden, especially among younger populations. The current findings emphasize the critical need for aggressive and targeted management of these risk factors through comprehensive public health campaigns and personalized medical interventions aimed at younger demographics (8, 16, 17). Furthermore, the significant decline in stroke-related mortality attributed to a reduction in household air pollution demonstrates the success of targeted environmental policy interventions. However, the increasing stroke burden associated with rising ambient temperatures owing to climate change has introduced new public health challenges. Increased temperature exposure has been linked to a higher incidence of cardiovascular events, possibly due to heat-induced physiological stress, dehydration, increased blood viscosity, and disruptions in fluid and electrolyte balance (24). As such, future stroke prevention initiatives must incorporate adaptive strategies to mitigate climate-related risks, including promoting awareness of heat-related health risks, establishing early warning systems for heat waves, improving healthcare infrastructure resilience, and developing community-specific preparedness and response strategies (24). These adaptive strategies should complement traditional cardiovascular risk management approaches to provide comprehensive stroke prevention in the face of evolving environmental threats.

Despite these insights, this study had certain limitations. First, the inherent methodological constraints of the GBD study, including potential misclassification and underreporting of stroke events due to reliance on administrative databases and ICD coding, must be acknowledged. Second, although the use of verbal autopsy to classify causes of death is common in the GBD dataset, the exact number of cases assigned using this method is not disclosed. Consequently, it was not possible to determine the proportion of stroke-related deaths, including SAH, that were classified based on verbal autopsy. Third, our analysis did not distinguish between stroke severity and recurrence, which restricted our ability to evaluate long-term functional outcomes and recurrence rates. Fourth, detailed race and ethnicity data, which are crucial for addressing disparities in the stroke burden, were not systematically included, limiting a comprehensive understanding of stroke epidemiology across different populations. Lastly, the use of global age standardization rather than US-specific demographics could potentially influence the observed temporal trends, particularly for subgroups characterized by distinct demographic shifts.

In conclusion, although considerable progress has been made in reducing the age-standardized burden of stroke, the rising absolute number of stroke cases, particularly among younger adults and specific geographic regions, underscores the complex and evolving public health challenge. Future public health strategies must prioritize tailored age- and region-specific interventions to address modifiable risk factors, improve healthcare access, and enhance stroke awareness. Continuous surveillance, robust risk factor control, and targeted healthcare interventions are essential to mitigate existing disparities and effectively manage the growing stroke burden in the US.

## Data Availability

Publicly available datasets were analyzed in this study. This data can be found at the Global Health Data Exchange (https://ghdx.healthdata.org). The GBD study is an open-source dataset that is freely accessible and available for use by anyone. The data in this study are available from the corresponding author upon reasonable request.

## Footnotes

^1^ https://ghdx.healthdata.org
