# Supplementary material for: Burden of stroke in the United States of America, 1990–2021: a systematic analysis for the US burden of disease study 2021
Source: Front Neurol. 2025 Aug 14;16:1609508. doi: 10.3389/fneur.2025.1609508 (PMC12394880; doi:10.3389/fneur.2025.1609508)
Supplement: Supplementary file 1 [file Data_Sheet_1.pdf]

frontiers

Supplementary Material

1 Supplementary Methods

1.1 ICD Codes used in fatal and non-fatal analysis

Fatal analysis	ICD10	ICD9
**Cerebrovascular disease**	G45-G46.8, I60-I63.9, I65-I66.9, I67.0-I67.3, I67.5-I67.6, I68.1-I68.2, I69.0-I69.3	430-435.9, 437.0-437.2, 437.5, 437.8
**Ischemic stroke**	G45-G46.8, I63-I63.9, I65-I66.9, I67.2-I67.3, I67.5-I67.6, 437.8 I69.3	433-435.9, 437.0-437.1, 437.5
**Hemorrhagic stroke**	I60-I62.9, I67.0-I67.1, I68.1-I68.2, I69.0-I69.2	430-432.9, 437.2

Nonfatal analysis	ICD10	ICD9
**Cerebrovascular disease**	I60-I63.9, I65-I66.9, I67.0-I67.3, I67.5-I67.6, I68.1-I68.2, I69.0- I69.3	430-434.9, 437.0-437.2, 437.5- 437.8
**Ischemic stroke**	stroke I63-I63.9, I65-I66.9, I67.2-I67.3, I67.5-I67.6, I69.3	433-434.9, 437.0-437.1, 437.5- 437.8
**Hemorrhagic stroke**	I60-I62.9, I67.0-I67.1, I68.1-I68.2, I69.0-I69.2	430-432.9, 437.2

1.2 Detailed case definition and Search terms for investigating the burden of stroke

Case definition

Stroke was defined according to WHO criteria as rapidly developing clinical signs of focal (or less commonly global) disturbance of cerebral function lasting more than 24 hours or leading to death with no apparent cause other than that of vascular origin. Cases of transient ischaemic attack (TIA) were not included.

Acute stroke: Stroke cases are considered acute from the day of incidence of a first-ever stroke through day 28 following the event.

Chronic stroke: Stroke cases are considered chronic beginning 28 days following the occurrence of an event. Chronic stroke includes the late sequelae of an acute stroke and all recurrent stroke events. GBD

2015 adopted this broader definition of chronic stroke than what was used in prior iterations to model acute strokes using only first-ever incident events.

Ischaemic stroke: Ischaemic strokes are characterised by occlusion of blood flow to part of the brain due to hypoperfusion, most commonly due to a thrombus or embolism. It is defined as an episode of neurological dysfunction caused by focal cerebral, spinal, or retinal infarction.

Intracerebral haemorrhage: Intracerebral haemorrhage is characterised by the rupture of a blood vessel resulting in bleeding into the intracerebral part of the brain. It is defined as focal collection of blood within the brain parenchyma or ventricular system that is not caused by trauma and results in a clinical stroke.

Subarachnoid haemorrhage: Subarachnoid haemorrhage is characterised as bleeding into the subarachnoid space (the space between the arachnoid membrane and the pia mater of the brain or spinal cord) resulting in a clinical stroke.

In cases where the exact subtype of stroke could not be determined (ICD-10 code I64), the cases were redistributed into ischemic stroke, ICH, and SAH based on the proportion of each subtype within the respective data source.

The reference definitions for ischaemic stroke and intracerebral haemorrhage were first-ever, subtype-specific stroke, which included subjects who did not survive to hospital admission. For these two subtypes we included, after adjustment, sources which used the following alternate definitions: 1) sources which included first and recurrent strokes; 2) sources which reported only estimates for all subtypes combined; and 3) sources which included only stroke cases which survived to hospital admission. The reference definition for subarachnoid haemorrhage was first-ever, subtype-specific stroke, with aneurysmal and non-aneurysmal events combined, which included subjects who did not survive to hospital admission. For subarachnoid haemorrhage, we included, after adjustment for bias, sources which used the following alternate definitions: 1) sources which included first and recurrent strokes; 2) sources which reported only estimates for aneurysmal subarachnoid haemorrhage; and 3) sources which included only stroke cases which survived to hospital admission.

Search terms

PubMed: ("stroke"[TIAB] OR "ischemic stroke"[TIAB] OR "ischaemic stroke"[TIAB] OR "cerebral infarction"[TIAB] OR "intracerebral hemorrhage"[TIAB] OR "intracerebral haemorrhage"[TIAB] OR "subarachnoid hemorrhage"[TIAB] OR "subarachnoid haemorrhage"[TIAB]) AND (incidence[TIAB] OR prevalence[TIAB] OR "excess mortality"[TIAB] OR "case fatality"[TIAB] OR "mortality ratio"[TIAB]) AND ("2017/09/01"[PDAT] : "2020/02/25"[PDAT])

1.3 Definition of risk factors

Risk factor	Definition
Behavioral risks	
Dietary risks	Composite risk factors consisting of suboptimal exposure to dietary factors including fruits, vegetables, whole grains, nuts and seeds, fiber, omega-3 fatty acids, polyunsaturated fatty acids, calcium, milk, legumes, red meat, processed meat, sugar-sweetened beverages, trans fatty acids, and sodium.

Tobacco	Composite risk factors consist of current or former users of any smoked tobacco product on a daily or occasional basis and current exposure of nonsmokers to secondhand tobacco smoke at home, at work, or in other public places.
High alcohol use	Grams per day of pure alcohol consumed among current drinkers greater than the age-, sex-, and region-specific TMREL.
Low physical activity	Physical activity performed by adults >25 years of age, for at least 10 minutes at a time, across all domains of life (leisure/recreation, work/household, and transport) <3,600-4,400 metabolic equivalent-minutes per week.
Environmental risks	
Air pollution	Ambient particulate matter pollution defined as population-weighted annual average mass concentration of PM2.5 in a cubic meter of air >2.4-5.9 $\mu g/m^3$ and household air pollution from solid fuels defined as the proportion of individuals exposed to >2.4-5.9 $\mu g/m^3$ of PM2.5 due to the use of solid fuels for cooking, including coal, charcoal, wood, agricultural residue, and animal dung.
Non-optimal temperature	Defined as exposure to temperatures warmer or colder than the temperature associated with the lowest overall mortality attributable to the risk, in a given location and year.
Lead exposure	Micrograms of lead per gram of bone greater than the age-specific TMREL.
Metabolic risks	
High systolic blood pressure (SBP)	Brachial SBP >105-115 mmHg in adults >25 years of age.
High low-density lipoprotein (LDL) cholesterol	Blood concentration of LDL cholesterol >0.9-1.4 mmol/L in adults >25 years of age.
High body-mass index	Body mass index >20-22.5 kg/m^2 in adults >20 years of age.
High fasting plasma glucose	Serum fasting plasma glucose >4.9-5.3 mmol/L in adults >25 years of age.
Kidney dysfunction	Estimated glomerular filtration rate ≤60 mL/min/1.73 m^2 and albumin-to-creatinine ratio >30 mg/g.
PM2.5 = particulate matter <2.5 $\mu\mu$m in diameter; TMREL = theoretical minimum risk exposure level.	

2 Supplementary Tables

2.1 Table S1a: Age-standardized rate and percentage change of incidence, prevalence, and mortality for all stroke over time, both sexes, by regions in US, 1990-2021

**All Stroke**	**Incidence**			**Prevalence**			**Mortality**		
**Location**	**1990**	**2021**	**change, %**	**1990**	**2021**	**change, %**	**1990**	**2021**	**change, %**
**Alabama**	127.89 (109.38 to 148.31)	94.12 (83.58 to 105.65)	-26.41 (-30.63 to -22.09)	1487.45 (1349.54 to 1615.97)	1606.69 (1480.65 to 1747.19)	8.02 (1.32 to 14.68)	57.96 (51.52 to 61.95)	46.17 (39.07 to 53.35)	-20.34 (-30.75 to -9.17)
**Alaska**	123.26 (104.20 to 145.28)	80.92 (71.03 to 92.03)	-34.35 (-38.13 to -29.87)	1212.42 (1093.07 to 1334.95)	1121.85 (1043.63 to 1208.05)	-7.47 (-13.36 to -1.61)	47.65 (41.61 to 51.49)	29.72 (24.38 to 34.34)	-37.64 (-44.11 to -29.54)
**Arizona**	100.92 (85.47 to 118.72)	68.20 (60.63 to 77.12)	-32.42 (-37.05 to -27.95)	1091.74 (1000.22 to 1200.84)	1075.80 (995.94 to 1161.89)	-1.46 (-7.22 to 5.09)	36.48 (31.61 to 39.24)	25.75 (21.50 to 30.07)	-29.42 (-38.84 to -20.00)
**Arkansas**	127.74 (109.61 to 147.89)	94.73 (83.40 to 107.24)	-25.84 (-30.21 to -20.96)	1450.44 (1316.81 to 1576.04)	1540.08 (1418.56 to 1664.43)	6.18 (-1.21 to 13.65)	57.69 (50.79 to 61.34)	36.11 (30.12 to 41.48)	-37.4 (-45.88 to -28.22)
**California**	109.42 (92.77 to 128.75)	70.82 (62.28 to 80.92)	-35.28 (-39.62 to -30.77)	1150.20 (1050.3 to 1262.67)	1079.00 (1003.84 to 1159.75)	-6.19 (-11.71 to 1.80)	44.80 (39.31 to 48.04)	27.89 (22.73 to 32.22)	-37.74 (-45.27 to -29.93)

**Colorado**	99.95 (84.13 to 120.11)	65.21 (57.29 to 73.28)	-34.76 (-40.14 to -29.35)	991.29 (895.62 to 1085.15)	951.26 (886.42 to 1021.64)	-4.04 (-9.88 to 2.47)	38.14 (32.81 to 41.11)	27.95 (21.88 to 32.58)	-26.72 (-36.9 to -16.15)
**Connecticut**	109.56 (94.22 to 128.55)	65.75 (57.34 to 74.82)	-39.98 (-43.70 to -36.34)	1108.59 (1019.58 to 1217.60)	981.98 (913.48 to 1053.27)	-11.42 (-16.72 to - 5.13)	36.25 (31.20 to 39.06)	22.45 (18.19 to 26.56)	-38.08 (-46.82 to -28.78)
**Delaware**	116.42 (99.85 to 134.53)	74.03 (65.22 to 83.98)	-36.42 (-40.04 to -32.19)	1223.81 (1107.22 to 1341.12)	1187.79 (1100.79 to 1278.50)	-2.94 (-9.06 to 4.11)	41.51 (36.63 to 44.72)	33.16 (27.92 to 37.97)	-20.12 (-28.11 to -11.04)
**District of Columbia**	126.07 (109.92 to 145.11)	68.39 (60.25 to 77.54)	-45.75 (-48.89 to -42.84)	1386.70 (1266.75 to 1521.40)	1224.14 (1138.16 to 1330.41)	-11.72 (-17.33 to -5.15)	53.54 (48.46 to 57.02)	27.45 (22.24 to 32.39)	-48.73 (-56.26 to -40.57)
**Florida**	103.67 (88.47 to 122.61)	73.91 (64.54 to 84.89)	-28.70 (-32.91 to -24.31)	1212.73 (1105.62 to 1311.67)	1233.21 (1148.66 to 1333.61)	1.69 (-4.66 to 8.22)	35.39 (30.81 to 37.92)	30.60 (23.93 to 35.87)	-13.54 (-25.13 to -2.07)
**Georgia**	131.68 (112.33 to 152.55)	84.58 (74.96 to 95.58)	-35.77 (-39.14 to -31.69)	1399.12 (1274.31 to 1526.77)	1355.05 (1263.32 to 1463.63)	-3.15 (-9.79 to 4.56)	58.57 (51.89 to 62.37)	37.91 (31.41 to 43.98)	-35.27 (-43.85 to -26.37)
**Hawaii**	114.12 (98.01 to 132.00)	72.36 (63.45 to 82.45)	-36.60 (-40.29 to -33.07)	1181.90 (1078.33 to 1288.63)	1093.35 (1020.11 to 1175.57)	-7.49 (-13.40 to -1.44)	42.77 (37.07 to 45.99)	27.13 (21.54 to 31.94)	-36.57 (-45.17 to - 27.07)

**Idaho**	111.91 (94.87 to 130.13)	74.75 (65.85 to 84.61)	-33.2 (-37.15 to -29.09)	1148.58 (1054.81 to 1255.57)	1073.01 (996.59 to 1149.39)	-6.58 (-12.46 to -0.23)	44.24 (38.05 to 47.89)	28.75 (23.89 to 32.81)	-35.01 (-43.39 to -26.93)
**Illinois**	120.45 (104.21 to 140.77)	75.70 (66.47 to 85.91)	-37.15 (-40.6 to -33.28)	1253.25 (1141.52 to 1368.18)	1186.39 (1103.62 to 1280.59)	-5.34 (-11.29 to 1.39)	44.45 (39.56 to 47.47)	31.81 (26.24 to 36.97)	-28.44 (-37.49 to -18.44)
**Indiana**	120.22 (103.38 to 139.03)	81.49 (71.60 to 92.81)	-32.22 (-36.59 to -28.01)	1279.51 (1171.45 to 1393.89)	1317.84 (1220.23 to 1422.65)	3.00 (-4.35 to 9.10)	49.45 (43.90 to 52.74)	36.38 (30.75 to 41.66)	-26.43 (-35.39 to -17.12)
**Iowa**	100.82 (84.76 to 120.15)	68.65 (60.47 to 78.21)	-31.91 (-36.76 to -26.94)	1050.59 (960.03 to 1147.15)	1061.93 (987.70 to 1149.25)	1.08 (-5.23 to 7.56)	38.92 (34.08 to 42.06)	26.73 (21.88 to 30.99)	-31.31 (-40.25 to -21.32)
**Kansas**	111.79 (95.53 to 130.45)	78.21 (68.63 to 88.90)	-30.04 (-33.97 to -26.05)	1194.53 (1087.23 to 1299.10)	1224.75 (1145.71 to 1322.34)	2.53 (-2.60 to 9.41)	41.76 (36.37 to 45.13)	30.66 (25.32 to 35.80)	-26.58 (-36.93 to -16.04)
**Kentucky**	120.66 (103.95 to 139.62)	83.14 (73.67 to 94.51)	-31.09 (-34.9 to -26.65)	1355.25 (1233.29 to 1469.93)	1445.72 (1323.29 to 1569.75)	6.68 (-0.68 to 14.04)	50.22 (44.2 to 53.66)	38.48 (31.88 to 44.65)	-23.38 (-32.98 to -12.53)
**Louisiana**	126.31 (108.74 to 146.63)	86.18 (75.73 to 98.06)	-31.77 (-35.53 to -27.82)	1410.87 (1291.27 to 1549.63)	1463.42 (1358.68 to 1585.2)	3.72 (-2 to 10.01)	51.54 (45.89 to 54.77)	40.31 (34.09 to 46.47)	-21.77 (-30.82 to -10.46)

**Maine**	109.05 (93.5 to 128.09)	71.89 (62.99 to 81.63)	-34.08 (-38.53 to -29.93)	1097.73 (1002.96 to 1199.67)	1086.75 (1014.09 to 1168.45)	-1 (-7 to 6.11)	39.4 (34.61 to 42.81)	26.44 (22.03 to 30.29)	-32.89 (-41.19 to -23.66)
**Maryland**	115.42 (98.79 to 134.87)	81.27 (72.26 to 91.44)	-29.59 (-34.4 to -24.91)	1262.77 (1159.17 to 1379.98)	1260.24 (1174 to 1353.69)	-0.2 (-6.29 to 6.49)	44.51 (39.5 to 47.72)	31.78 (25.95 to 37.32)	-28.59 (-39.17 to -17.19)
**Massachusetts**	98.3 (83.29 to 116.65)	64.91 (56.11 to 74.64)	-33.96 (-38.08 to -29.32)	1084.41 (989.29 to 1179.24)	1027.68 (959.1 to 1102.29)	-5.23 (-10.63 to 0.58)	37.7 (32.71 to 40.74)	22.54 (17.98 to 26.19)	-40.22 (-49.02 to -31.68)
**Michigan**	112.88 (96.08 to 132.43)	78.17 (68.56 to 88.58)	-30.75 (-34.66 to -26.29)	1281.91 (1160.48 to 1409.06)	1333.16 (1238.11 to 1428.02)	4 (-2.49 to 11.23)	43.49 (38.52 to 46.51)	30.28 (24.96 to 34.78)	-30.38 (-39.15 to -20.96)
**Minnesota**	106.26 (90.59 to 124.02)	73.84 (65 to 84.01)	-30.51 (-35.22 to -25.69)	1075.72 (980.66 to 1189.65)	1042.89 (970.42 to 1113.52)	-3.05 (-9.33 to 4.25)	44.11 (38.65 to 47.6)	28.24 (22.99 to 32.49)	-35.96 (-44.86 to -27.2)
**Mississippi**	128.1 (110.53 to 148.75)	91.17 (81.02 to 103.01)	-28.83 (-32.6 to -24.8)	1463.27 (1335.18 to 1593.91)	1586 (1477.31 to 1724.93)	8.39 (1.65 to 15.35)	54.92 (49.38 to 58.53)	45.66 (39.17 to 52.67)	-16.86 (-27.42 to -4.23)
**Missouri**	116.13 (99.6 to 135.16)	79.54 (70.49 to 90)	-31.51 (-35.27 to -27.24)	1255.07 (1147.29 to 1375.31)	1299.56 (1210.6 to 1404.49)	3.54 (-3.36 to 10.12)	44.29 (39.09 to 47.29)	33.06 (28.17 to 38.2)	-25.36 (-35.99 to -15.02)
**Montana**	112.88 (96.56 to 130.73)	73.61 (64.14 to 83.25)	-34.79 (-38.74 to -30.78)	1178.41 (1076.32 to 1285.22)	1144.97 (1064.93 to 1227.59)	-2.84 (-8.66 to 3.47)	43.5 (37.64 to 47.27)	25.39 (20.97 to 29.42)	-41.64 (-48.72 to -33.18)

**Nebraska**	112.65 (96.25 to 131.56)	73.92 (64.66 to 83.78)	-34.38 (-38.66 to -30.41)	1140.62 (1039.73 to 1250.99)	1094.63 (1025.74 to 1169.19)	-4.03 (-9.52 to 2.5)	42.49 (37.05 to 46.05)	28.62 (23.71 to 33)	-32.63 (-41.41 to -24.3)
**Nevada**	137.38 (117.54 to 160.31)	93.22 (81.71 to 105.75)	-32.15 (-36.06 to -28.23)	1385.32 (1280.56 to 1495.51)	1318.1 (1224.64 to 1422.49)	-4.85 (-9.79 to 1.4)	46.64 (41.62 to 49.99)	29.63 (24.98 to 34.05)	-36.46 (-44.53 to -28.2)
**New Hampshire**	110.54 (94.34 to 128.73)	68.59 (59.8 to 78.42)	-37.95 (-41.52 to -34.1)	1176.34 (1067.04 to 1291.57)	1057.12 (983.92 to 1137.26)	-10.14 (-16.25 to -3.83)	41.11 (35.92 to 44.37)	25.17 (20.72 to 29.01)	-38.78 (-46.21 to -30.7)
**New Jersey**	105.88 (90.17 to 124.27)	68.69 (60.38 to 78.78)	-35.12 (-38.99 to -31.3)	1176.17 (1073.26 to 1292.01)	1065.81 (996.99 to 1140.09)	-9.38 (-15.3 to -3.13)	37.79 (33.65 to 40.52)	24.07 (19.5 to 28.05)	-36.29 (-45.98 to -27.4)
**New Mexico**	107.44 (92.24 to 124.5)	70.12 (61.38 to 80.17)	-34.73 (-38.31 to -31.22)	1107.62 (1008.96 to 1216.25)	1111.38 (1032.31 to 1188.4)	0.34 (-6.18 to 7.15)	37.65 (32.88 to 40.87)	25.08 (20.59 to 29.18)	-33.38 (-42.53 to -23.14)
**New York**	99.81 (85.13 to 116.96)	62.22 (55.02 to 70.83)	-37.67 (-41.55 to -33.49)	1116.63 (1021.41 to 1213.74)	1041.12 (964.84 to 1128.48)	-6.76 (-12.56 to -0.64)	34.16 (30.5 to 36.43)	17.87 (14.59 to 20.88)	-47.7 (-55.34 to -40.28)
**North Carolina**	126.74 (108.21 to 148.53)	89.33 (78.75 to 100.93)	-29.52 (-34.02 to -25.04)	1400.92 (1274 to 1526.57)	1413.53 (1313.05 to 1516.77)	0.9 (-5.65 to 6.97)	54.8 (48.58 to 58.93)	35.59 (29.24 to 41.04)	-35.06 (-43.57 to -26.46)
**North Dakota**	108.67 (93.74 to 126.22)	73.5 (65 to 84.23)	-32.36 (-36.21 to -28.18)	1054.89 (958.28 to 1142.77)	1078.38 (1007.63 to 1151.64)	2.23 (-3.34 to 8.49)	40.57 (35.3 to 44.22)	24.82 (21.06 to 28.68)	-38.82 (-46.29 to -30.05)

**Ohio**	116.42 (99.43 to 135.12)	79.92 (71.06 to 90.83)	-31.35 (-35.48 to -27.24)	1271.91 (1166.69 to 1390.22)	1315.96 (1222.92 to 1407.79)	3.46 (-2.61 to 10.46)	43.6 (38.83 to 46.51)	35.71 (29.83 to 40.83)	-18.08 (-27.78 to -7.48)
**Oklahoma**	119.16 (102.13 to 138.37)	84.52 (74.73 to 96.18)	-29.06 (-33.49 to -24.84)	1315.61 (1206.48 to 1429.37)	1392.5 (1294.79 to 1504.67)	5.84 (-0.27 to 13.67)	48.79 (42.83 to 52.2)	33.71 (28.34 to 39.3)	-30.92 (-39.58 to -21.11)
**Oregon**	119.25 (102.52 to 139.32)	76.21 (66.97 to 86.71)	-36.09 (-39.92 to -31.85)	1219.3 (1106.16 to 1331.31)	1178.46 (1091.36 to 1267.89)	-3.35 (-10.08 to 3.91)	47.31 (41.56 to 51.16)	30.75 (25.06 to 36.07)	-35 (-43.04 to -25.27)
**Pennsylvania**	117.74 (100.82 to 137.06)	73.92 (65 to 83.74)	-37.22 (-40.79 to -32.92)	1243.22 (1138.15 to 1359.17)	1215.97 (1133.53 to 1299.85)	-2.19 (-8.24 to 4.47)	41.73 (37.14 to 44.65)	28.68 (23.58 to 33.3)	-31.28 (-39.96 to -21.48)
**Rhode Island**	105.13 (90.42 to 123.63)	65.38 (57.51 to 74.71)	-37.8 (-41.59 to -33.89)	1100.32 (1004.01 to 1206.68)	1020.33 (945.42 to 1096.97)	-7.27 (-12.86 to -1.45)	35.46 (31.2 to 38.27)	21.99 (18.05 to 25.71)	-38 (-45.87 to -29.25)
**South Carolina**	136.94 (118.47 to 159.31)	87.81 (77.57 to 98.74)	-35.88 (-40.35 to -31.97)	1445.65 (1322.39 to 1585.15)	1425.6 (1326.36 to 1545.75)	-1.39 (-7.25 to 4.95)	62.91 (56.56 to 67.19)	40.05 (33.44 to 45.56)	-36.35 (-44.96 to -27.59)
**South Dakota**	106.26 (90.91 to 123.04)	72.9 (63.67 to 82.92)	-31.4 (-35.23 to -27.5)	1104.21 (1009.68 to 1201.46)	1091.34 (1018.54 to 1174.91)	-1.17 (-7 to 5.36)	38.89 (33.77 to 41.96)	24.87 (20.91 to 28.28)	-36.05 (-43.27 to -28.13)
**Tennessee**	126.45 (108.11 to 147.19)	85.91 (76.33 to 97.06)	-32.06 (-36.46 to -27.4)	1423.98 (1296.51 to 1546.43)	1453.66 (1341.5 to 1568.13)	2.08 (-4.91 to 8.38)	55.92 (49.9 to 59.76)	37.13 (31.55 to 42.43)	-33.59 (-41.44 to -24.85)

**Texas**	118.26 (101.87 to 136.53)	79.6 (70.1 to 90.24)	-32.69 (-36.63 to -28.3)	1308 (1197.17 to 1428.34)	1265.25 (1175.27 to 1354.94)	-3.27 (-9.02 to 3.77)	47.12 (41.61 to 50.66)	33.58 (28.42 to 38.27)	-28.73 (-36.86 to -19.54)
**Utah**	110.08 (94.68 to 127.87)	73.66 (64.58 to 83.24)	-33.09 (-37.59 to -29.13)	1147.16 (1052.87 to 1257.52)	1083.31 (1010.8 to 1162.7)	-5.57 (-10.98 to 0.42)	44.76 (39.05 to 48.24)	30.57 (25.72 to 34.95)	-31.7 (-39.5 to -23.6)
**Vermont**	109.32 (93.28 to 128.43)	66.74 (57.96 to 76.12)	-38.95 (-42.41 to -35.17)	1107.03 (1008.31 to 1211.04)	1026.36 (951.48 to 1097.62)	-7.29 (-13.29 to -1.14)	39.48 (34.83 to 42.67)	23.62 (19.6 to 26.99)	-40.18 (-46.25 to -32.71)
**Virginia**	124 (106.47 to 145.12)	77.35 (67.85 to 87.83)	-37.62 (-41.21 to -33.97)	1266.49 (1153.14 to 1393.06)	1213.93 (1129.02 to 1307.42)	-4.15 (-10.3 to 3.13)	52.21 (46.77 to 55.68)	32.68 (26.89 to 37.88)	-37.41 (-46.12 to -28.18)
**Washington**	107.5 (90.64 to 128.81)	69.77 (61.89 to 78.81)	-35.1 (-40.17 to -29.47)	1132.63 (1035.34 to 1235.14)	1087.66 (1012.68 to 1167.94)	-3.97 (-9.68 to 2.19)	44.77 (39.35 to 48.23)	26.88 (21.81 to 31.2)	-39.98 (-48.2 to -32.13)
**West Virginia**	115.76 (98.29 to 134.84)	82.44 (72.68 to 93.56)	-28.79 (-33.07 to -24.18)	1251.75 (1140.35 to 1359.46)	1351.13 (1255.06 to 1457.51)	7.94 (1.45 to 16.13)	44.28 (39.53 to 47.59)	34.74 (29.87 to 39.97)	-21.54 (-31.48 to -11.22)
**Wisconsin**	105.93 (89.63 to 124.82)	68.9 (61.02 to 77.67)	-34.96 (-39.76 to -30.06)	1031.35 (944.97 to 1126.23)	977.76 (912.21 to 1056.75)	-5.2 (-10.6 to 1.54)	43.45 (37.92 to 46.59)	27 (21.89 to 31.34)	-37.85 (-46.13 to -28.38)
**Wyoming**	110.69 (94.08 to 129.24)	70.47 (61.93 to 80.56)	-36.33 (-40.07 to -32.45)	1139 (1037.38 to 1243.01)	1059.55 (982.77 to 1144.58)	-6.98 (-12.58 to -0.82)	42.14 (36.9 to 45.81)	26.91 (22.45 to 30.69)	-36.14 (-43.14 to -29)

2.2 **Table S1b: Age-standardized rate and percentage change of DALYs, YLDs, and YLLs for all stroke over time, both sexes, by regions in US, 1990-2021**

All Stroke	DALYs			YLDs			YLLs		
**Location**	**1990**	**2021**	**change, %**	**1990**	**2021**	**change, %**	**1990**	**2021**	**change, %**
**Alabama**	1296.59 (1201.53 to 1387.56)	1090.24 (944.78 to 1236.29)	-15.91 (-25.62 to -5.4)	209.62 (150.55 to 267.93)	221.51 (160.13 to 282.82)	5.67 (-2.21 to 13.14)	1086.97 (1003.64 to 1149.49)	868.73 (742.6 to 1011.33)	-20.08 (-31.51 to -7.92)
**Alaska**	997.67 (917.6 to 1077.01)	683.99 (608.06 to 762)	-31.44 (-37.22 to -24.54)	171.59 (123.45 to 224.73)	156.85 (113.41 to 200.84)	-8.59 (-15.41 to -1.1)	826.09 (753.59 to 880.78)	527.15 (453.29 to 603.15)	-36.19 (-43.03 to -27.79)
**Arizona**	787.48 (722.23 to 851.34)	605.19 (524.8 to 684.62)	-23.15 (-31.85 to -14.33)	154.74 (110.05 to 200.04)	148.81 (108.29 to 188.52)	-3.83 (-10.51 to 4.66)	632.74 (574.96 to 672.45)	456.38 (389.84 to 530.45)	-27.87 (-38.61 to -16.98)
**Arkansas**	1262.44 (1173.99 to 1352.46)	914.67 (793.99 to 1032.19)	-27.55 (-35.5 to -18.97)	205.09 (146.27 to 270.06)	213.56 (154.44 to 268.27)	4.13 (-4.36 to 13.31)	1057.35 (974.91 to 1112.09)	701.11 (595.99 to 809.47)	-33.69 (-43.04 to -23.14)
**California**	946.97 (870.36 to 1020.34)	630.02 (550.01 to 706.72)	-33.47 (-40.61 to -26.94)	161.34 (115.45 to 206.34)	148.78 (107.39 to 190.62)	-7.79 (-14.66 to 0.66)	785.63 (721.48 to 830.43)	481.24 (407.55 to 552.83)	-38.74 (-46.66 to -30.16)
**Colorado**	777.25 (707.3 to 836.76)	566.26 (480.11 to 642.36)	-27.15 (-35.29 to -18.1)	141.52 (99.09 to 178.04)	132.84 (96.07 to 170.95)	-6.13 (-13.18 to 1.39)	635.73 (575.48 to 673.72)	433.42 (350.22 to 505.98)	-31.82 (-41.82 to -20.81)

**Connecticut**	785.31 (714.04 to 850.47)	497.18 (424.04 to 571.81)	-36.69 (-43.63 to -29)	158.26 (111.88 to 202.52)	137.97 (99.45 to 176.49)	-12.82 (-19.69 to -5.96)	627.04 (567.78 to 666.09)	359.21 (297.31 to 419.55)	-42.71 (-51.73 to -33.2)
**Delaware**	917.2 (844.92 to 981.5)	718.32 (640.59 to 803.33)	-21.68 (-28.18 to -14.07)	172.54 (121.56 to 221.1)	164.29 (117.97 to 208.67)	-4.78 (-11.06 to 3.11)	744.66 (685.15 to 791.13)	554.03 (484.52 to 627.31)	-25.6 (-33.69 to -16.38)
**District of Columbia**	1348.34 (1261.41 to 1434.47)	689.5 (593.37 to 792.58)	-48.86 (-54.91 to -42.54)	195.27 (141.86 to 255.31)	170.97 (124.46 to 219.21)	-12.45 (-19.06 to -4.83)	1153.07 (1077.5 to 1214.47)	518.53 (430.52 to 615.28)	-55.03 (-61.83 to -47.4)
**Florida**	842.74 (773.05 to 904.63)	697.3 (601.67 to 789.03)	-17.26 (-26.46 to -7.68)	170.57 (120.52 to 218.01)	169.64 (122.25 to 216.79)	-0.54 (-7.31 to 7.83)	672.17 (614.7 to 711.08)	527.66 (437.58 to 611.84)	-21.5 (-33.27 to -9.62)
**Georgia**	1284.78 (1188.09 to 1368.66)	868.62 (755.17 to 985.86)	-32.39 (-40.46 to -23.54)	197.5 (138.83 to 254.17)	187.78 (137.62 to 239.41)	-4.92 (-12.19 to 3.76)	1087.28 (1007.32 to 1147.27)	680.84 (577.69 to 787.59)	-37.38 (-46.47 to -27.81)
**Hawaii**	953.72 (867.74 to 1029.62)	646.24 (555.57 to 737.33)	-32.24 (-40.1 to -23.55)	167.53 (118.98 to 214.98)	153.01 (111.49 to 194.81)	-8.67 (-15.36 to -2.08)	786.18 (717.02 to 833.33)	493.23 (409.03 to 577.15)	-37.26 (-46.49 to -27.26)
**Idaho**	886.87 (793.94 to 957.82)	610.76 (535.82 to 689.23)	-31.13 (-38.43 to -23.8)	162.78 (114.48 to 210.58)	149.93 (107.53 to 191.83)	-7.9 (-14.86 to -0.21)	724.09 (649.77 to 774.37)	460.83 (390.32 to 526.68)	-36.36 (-45.3 to -26.89)
**Illinois**	987.13 (912.98 to 1054.58)	707.01 (621.54 to 809.68)	-28.38 (-36.33 to -20.16)	177.1 (125.64 to 228.19)	165.41 (118.83 to 211.81)	-6.6 (-13.67 to 1.25)	810.03 (747.93 to 855.72)	541.61 (460.81 to 628.46)	-33.14 (-42.3 to -22.85)

**Indiana**	1035.1 (951.63 to 1109.19)	810.93 (707.13 to 911.09)	-21.66 (-29.74 to -12.59)	179.39 (129.44 to 230.7)	180.18 (131.42 to 228.92)	0.44 (-7.4 to 8.73)	855.7 (789.98 to 902.25)	630.75 (536.9 to 719.93)	-26.29 (-35.8 to -15.32)
**Iowa**	797.84 (735.23 to 863.91)	606.26 (520.83 to 687.69)	-24.01 (-32.56 to -14.57)	149.28 (107.4 to 192.96)	148.1 (106.81 to 188)	-0.79 (-8.32 to 7.39)	648.56 (590.5 to 692.93)	458.16 (384.4 to 529.42)	-29.36 (-39.88 to -17.78)
**Kansas**	884.61 (805.62 to 951.82)	695.11 (608.02 to 786.75)	-21.42 (-30.6 to -11.87)	169.64 (121.9 to 216.62)	170.05 (122.42 to 214.72)	0.25 (-6.18 to 8.34)	714.97 (648.65 to 763.63)	525.06 (446.96 to 612.69)	-26.56 (-37.75 to -14.61)
**Kentucky**	1072.49 (985.86 to 1149.65)	894.1 (765.8 to 1016.79)	-16.63 (-25.79 to -6.16)	190.2 (134.43 to 246.47)	197.55 (143.64 to 249.22)	3.86 (-4.37 to 12.62)	882.29 (813.06 to 932.49)	696.55 (586.99 to 815.88)	-21.05 (-32.17 to -8.77)
**Louisiana**	1199.7 (1118.66 to 1285.31)	949.11 (831.97 to 1079.4)	-20.89 (-29.34 to -11)	198.12 (146 to 256.43)	200.63 (144.25 to 256.13)	1.27 (-5.48 to 8.49)	1001.58 (929.3 to 1053.59)	748.48 (644.69 to 868.57)	-25.27 (-34.79 to -13.45)
**Maine**	824 (747.03 to 890.3)	595.28 (516.35 to 671.88)	-27.76 (-35.49 to -19.69)	155.43 (111.15 to 199.36)	150.26 (110.56 to 191.86)	-3.33 (-10.19 to 4.74)	668.57 (608.91 to 713.98)	445.02 (376.69 to 513.02)	-33.44 (-42.25 to -22.92)
**Maryland**	965.11 (888.01 to 1032.7)	715.98 (622.28 to 817.5)	-25.81 (-34.46 to -16.14)	179.08 (130.58 to 230.45)	175.65 (126.53 to 225.47)	-1.91 (-9.14 to 5.84)	786.04 (724.97 to 832.04)	540.32 (451.32 to 629.96)	-31.26 (-42.22 to -19.86)
**Massachusetts**	799.8 (735.15 to 862.55)	492.34 (418.94 to 564.29)	-38.44 (-45.6 to -30.66)	152.81 (108.28 to 197.33)	142.14 (102.63 to 178.26)	-6.98 (-14.03 to 0.12)	646.99 (589.53 to 689.21)	350.2 (289.3 to 407.52)	-45.87 (-54.28 to -37.03)

**Michigan**	965.84 (894.03 to 1034.1)	693.8 (599.1 to 788.15)	-28.17 (-35.78 to -20.15)	181.09 (130.08 to 233.99)	183.72 (133.03 to 232.36)	1.45 (-5.84 to 9.39)	784.75 (725.53 to 826.44)	510.08 (437.01 to 582.28)	-35 (-43.86 to -25.17)
**Minnesota**	859.67 (789.69 to 928.01)	595.19 (508.26 to 673.97)	-30.77 (-39.09 to -22.87)	152.83 (107.99 to 194.52)	145.32 (103.74 to 186.02)	-4.91 (-12.35 to 4.04)	706.85 (640.13 to 751.48)	449.87 (375.42 to 519.76)	-36.36 (-45.59 to -27)
**Mississippi**	1275.64 (1187 to 1364.21)	1126.18 (969.66 to 1277.73)	-11.72 (-22.31 to 0.24)	206.6 (148.44 to 268.34)	219.43 (159.28 to 278.22)	6.21 (-1.79 to 14.48)	1069.04 (995.77 to 1128.57)	906.74 (781.46 to 1055.26)	-15.18 (-27.22 to -1)
**Missouri**	968.76 (890.85 to 1040.69)	767.4 (668.05 to 865.73)	-20.79 (-30.17 to -11.97)	177.63 (127.67 to 229.46)	179.65 (129.12 to 227.25)	1.14 (-6.57 to 9.02)	791.13 (726.85 to 835.12)	587.75 (507.02 to 680.82)	-25.71 (-36.66 to -14.24)
**Montana**	896.42 (822.92 to 968.7)	598.32 (526.2 to 674.84)	-33.26 (-39.78 to -24.93)	167.34 (119.77 to 218.12)	158.99 (114.47 to 201.87)	-4.99 (-11.51 to 2.38)	729.09 (660.8 to 779.22)	439.32 (373.72 to 505.77)	-39.74 (-47.52 to -30.2)
**Nebraska**	883.21 (806.71 to 948)	638 (552.64 to 714.64)	-27.76 (-35.81 to -20.19)	161.76 (115.67 to 206.09)	152.21 (109.08 to 194.67)	-5.9 (-12.74 to 1.23)	721.45 (658.06 to 771.09)	485.79 (411.3 to 556.13)	-32.66 (-41.95 to -23.42)
**Nevada**	1034.34 (949.91 to 1112.66)	715.35 (622.9 to 806.33)	-30.84 (-38.47 to -23.31)	196.39 (140.23 to 250.31)	183.94 (133.78 to 236.2)	-6.34 (-12.81 to 1.06)	837.95 (770.95 to 887.19)	531.42 (453.36 to 610.19)	-36.58 (-45.66 to -27.63)
**New Hampshire**	850.18 (778.3 to 914.3)	539.68 (467.9 to 604.07)	-36.52 (-42.51 to -29.45)	166.54 (119.6 to 212.9)	146.39 (106.15 to 185.88)	-12.1 (-18.78 to -4.53)	683.64 (623.24 to 728.75)	393.29 (332.6 to 452.38)	-42.47 (-50.15 to -34.04)

**New Jersey**	870.4 (806.67 to 934.2)	558.92 (479.89 to 634.29)	-35.79 (-43.06 to -28.1)	166.08 (118.08 to 214.4)	148.36 (106.76 to 191.26)	-10.67 (-17.53 to -4.17)	704.32 (651.43 to 743.96)	410.56 (341.33 to 484.58)	-41.71 (-50.49 to -32.14)
**New Mexico**	814.59 (747.94 to 878.15)	611.97 (524.52 to 698.18)	-24.87 (-33.36 to -15.54)	156.62 (113.45 to 203.04)	153.42 (112.27 to 196.68)	-2.05 (-9.18 to 5.74)	657.97 (596.17 to 701.34)	458.55 (387.98 to 530.47)	-30.31 (-40.45 to -19.01)
**New York**	824.16 (763.75 to 880.35)	471.24 (409.79 to 538.38)	-42.82 (-48.87 to -36.24)	157.48 (110.3 to 205.06)	143.58 (104.68 to 182.27)	-8.83 (-15.94 to -1.14)	666.68 (621.29 to 701.99)	327.66 (274.5 to 382.29)	-50.85 (-58.19 to -42.99)
**North Carolina**	1193.06 (1106.7 to 1275.7)	823.84 (724.01 to 920.78)	-30.95 (-38.48 to -23.23)	197.12 (140.71 to 254.68)	193.87 (140.46 to 245.11)	-1.65 (-8.59 to 5.49)	995.94 (920.53 to 1054.61)	629.98 (531.27 to 730.81)	-36.75 (-46.17 to -27.37)
**North Dakota**	831.46 (755.7 to 902.79)	587.25 (515.53 to 662.98)	-29.37 (-36.3 to -21)	149.49 (108.1 to 194.65)	149.77 (107.13 to 192.5)	0.19 (-6.52 to 7.74)	681.97 (622.29 to 730.38)	437.47 (376.24 to 501.91)	-35.85 (-44.59 to -25.87)
**Ohio**	948.28 (880.32 to 1015.53)	787.27 (689.25 to 885.88)	-16.98 (-25.47 to -7.61)	179.85 (126.82 to 231.55)	181.05 (129.54 to 231.61)	0.67 (-6.78 to 8.26)	768.43 (707.46 to 809.53)	606.22 (515.28 to 692.63)	-21.11 (-31.09 to -8.99)
**Oklahoma**	1055.16 (974.08 to 1127.82)	822.49 (726.27 to 937.27)	-22.05 (-30.14 to -12.83)	186.02 (133.18 to 239.43)	191.25 (137.45 to 244.4)	2.81 (-4.15 to 12.12)	869.14 (793.62 to 916.12)	631.24 (545.18 to 729.25)	-27.37 (-37.14 to -16.29)
**Oregon**	952.96 (874.45 to 1021.47)	668.64 (576.62 to 770.73)	-29.83 (-37.92 to -20.73)	173.29 (122.43 to 222.68)	164.74 (119.12 to 208.63)	-4.93 (-12 to 2.99)	779.67 (716.79 to 829.82)	503.9 (429.8 to 590.3)	-35.37 (-44.39 to -24.85)

**Pennsylvania**	921.44 (855.38 to 982.53)	646.35 (560.08 to 739.38)	-29.85 (-37.31 to -21.4)	175.49 (125.87 to 226.7)	167.5 (122.34 to 210.96)	-4.56 (-11.22 to 2.85)	745.95 (687.45 to 785.8)	478.86 (404.4 to 552.88)	-35.81 (-44.36 to -25.84)
**Rhode Island**	778.13 (707.13 to 838.85)	499.11 (425.53 to 570.4)	-35.86 (-43.2 to -28.78)	156.2 (110.83 to 200)	142.02 (102.4 to 180.62)	-9.08 (-15.53 to -1.52)	621.93 (565.59 to 662.41)	357.1 (298.06 to 421.1)	-42.58 (-50.5 to -33.78)
**South Carolina**	1410.1 (1315.23 to 1499.92)	924.5 (795.2 to 1055.14)	-34.44 (-42.2 to -25.7)	203.96 (145.78 to 264.12)	195.88 (141.45 to 248.29)	-3.96 (-10.67 to 4.64)	1206.14 (1126.08 to 1276.63)	728.62 (614.53 to 837.95)	-39.59 (-48.61 to -29.86)
**South Dakota**	829.85 (756.04 to 894.92)	599.48 (527.26 to 672.9)	-27.76 (-34.8 to -20.32)	156.56 (111.52 to 202.83)	151.89 (109.55 to 194.6)	-2.99 (-10.09 to 4.83)	673.28 (615.43 to 718.16)	447.59 (390.74 to 508.27)	-33.52 (-41.61 to -24.23)
**Tennessee**	1205.69 (1119.62 to 1291.03)	890.13 (780.69 to 1004.09)	-26.17 (-33.53 to -17.62)	201.27 (144.22 to 262.84)	199.82 (143.53 to 254.25)	-0.72 (-8.11 to 6.36)	1004.42 (934.89 to 1059.48)	690.31 (593.03 to 796.96)	-31.27 (-39.89 to -21.19)
**Texas**	1032.06 (956.71 to 1103.36)	774.75 (682.26 to 869.56)	-24.93 (-32.65 to -16.9)	185.61 (131.7 to 238.6)	176.23 (126.93 to 226.03)	-5.05 (-11.57 to 3.13)	846.45 (782.78 to 897.51)	598.52 (521.88 to 684.33)	-29.29 (-38.23 to -19.25)
**Utah**	891.49 (814.37 to 963.08)	631.41 (559.65 to 715.01)	-29.17 (-35.91 to -21.32)	163.33 (117.51 to 211.32)	151.26 (110.38 to 191.34)	-7.39 (-13.6 to -0.09)	728.16 (664.81 to 776.38)	480.15 (415.49 to 549.56)	-34.06 (-42.08 to -24.71)
**Vermont**	818.74 (744.88 to 883.67)	520.41 (457.1 to 582.08)	-36.44 (-41.86 to -29.51)	156.59 (111.81 to 205.02)	143.05 (103.69 to 182.98)	-8.64 (-15.44 to -0.83)	662.15 (602.67 to 707.84)	377.35 (325.27 to 429.19)	-43.01 (-49.39 to -34.98)

**Virginia**	1084.81 (1011.75 to 1158.39)	723.4 (623.95 to 820.93)	-33.32 (-41.37 to -25.11)	179.68 (127.15 to 230.72)	169.34 (122.87 to 217.6)	-5.76 (-12.86 to 1.51)	905.13 (841.02 to 953.91)	554.06 (468.6 to 641.94)	-38.79 (-48.07 to -28.95)
**Washington**	896.62 (816.17 to 967.78)	585.55 (500.1 to 666.85)	-34.69 (-42.39 to -27.65)	160.98 (114.52 to 207.71)	151.93 (109.81 to 192.86)	-5.62 (-12.05 to 2.46)	735.64 (670.88 to 782.04)	433.62 (361.44 to 501.29)	-41.05 (-49.81 to -32.6)
**West Virginia**	972.98 (900.37 to 1050.08)	844.11 (732.83 to 953.63)	-13.25 (-23.16 to -2.16)	177.25 (125.62 to 228.3)	185.09 (133.81 to 235.69)	4.42 (-3.36 to 13.57)	795.73 (734.59 to 844.4)	659.01 (569.73 to 761.05)	-17.18 (-29.2 to -4.34)
**Wisconsin**	872.68 (799.69 to 937.35)	578.52 (499.32 to 660.11)	-33.71 (-41.51 to -25.45)	146.03 (104.32 to 189.16)	136.31 (97.37 to 175.1)	-6.66 (-13.16 to 1.25)	726.64 (664.99 to 773.85)	442.21 (367.44 to 514.48)	-39.14 (-47.76 to -29.36)
**Wyoming**	872.8 (797.94 to 949.2)	613.17 (538.68 to 691.74)	-29.75 (-36.59 to -23.01)	161.62 (116.4 to 209.46)	147.69 (105.28 to 188.27)	-8.62 (-15.16 to -1.1)	711.18 (650.72 to 760.63)	465.47 (402.28 to 526.55)	-34.55 (-42.37 to -26.64)

2.3 Table S2a: Age-standardized rate and percentage change of incidence, prevalence, and mortality for ischemic stroke over time, both sexes, by regions in US, 1990-2021

Ischemic stroke	Incidence			Prevalence			Mortality		
**Location**	**1990**	**2021**	**change, %**	**1990**	**2021**	**change, %**	**1990**	**2021**	**change, %**
**Alabama**	99.01 (80.92 to 119.1)	68.68 (58.07 to 79.79)	-30.64 (-35.73 to -25.66)	1200.67 (1060.94 to 1326.87)	1268.65 (1147.45 to 1407.79)	5.66 (-2.42 to 13.81)	37.4 (32.33 to 40.42)	26.57 (21.84 to 30.61)	-28.94 (-37.34 to -19.87)
**Alaska**	98.64 (80.61 to 119.58)	61.19 (51.38 to 72.07)	-37.96 (-42.12 to -32.42)	1012.16 (897.48 to 1133.81)	906.83 (825.08 to 992.44)	-10.41 (-17.13 to -3.14)	33.23 (28.3 to 36.18)	17.43 (13.69 to 20.31)	-47.56 (-53.31 to -40.78)
**Arizona**	79.34 (64.09 to 97.38)	51.73 (43.87 to 60.31)	-34.8 (-40.03 to -29.37)	892.06 (800.25 to 1005.58)	868.28 (787.78 to 949.19)	-2.67 (-9.41 to 5.08)	24.5 (20.62 to 26.66)	15.23 (12.38 to 17.87)	-37.85 (-45.83 to -30)
**Arkansas**	101.4 (83.25 to 121.27)	71.27 (59.92 to 83.35)	-29.72 (-34.57 to -23.88)	1201.76 (1070.35 to 1324.19)	1250.36 (1132.03 to 1376.86)	4.04 (-4.89 to 12.92)	38.38 (33.01 to 41.15)	19.24 (15.73 to 22.12)	-49.86 (-56.42 to -42.77)
**California**	85.46 (69.61 to 104.8)	52.52 (44.18 to 62.07)	-38.55 (-43.37 to -33.03)	926.3 (830.83 to 1030.89)	850.35 (777.92 to 927.47)	-8.2 (-14.7 to 1.38)	30.16 (25.6 to 32.73)	16.13 (12.66 to 18.88)	-46.52 (-52.95 to -39.71)
**Colorado**	78.88 (63.08 to 98.32)	48.48 (40.97 to 56.15)	-38.54 (-44.26 to -32.24)	795.58 (699.51 to 889.22)	746.93 (688.73 to 813.87)	-6.11 (-13.26 to 2.24)	26.16 (21.91 to 28.51)	17.7 (13.38 to 20.67)	-32.37 (-41.71 to -22.93)

**Connecticut**	85.31 (69.63 to 103.77)	48.92 (41.05 to 58.2)	-42.66 (-46.93 to -38.15)	897.14 (801.37 to 1010.37)	777.46 (709.16 to 844.34)	-13.34 (-19.55 to -5.69)	24.13 (20.07 to 26.3)	13.06 (10.18 to 15.6)	-45.87 (-53.49 to -38.17)
**Delaware**	90.92 (74.87 to 108.7)	54.75 (46.1 to 64.51)	-39.78 (-43.78 to -34.85)	996.53 (878.09 to 1111.4)	945.46 (861.41 to 1034.36)	-5.12 (-12.41 to 3)	27.02 (23.4 to 29.31)	19.64 (15.9 to 22.51)	-27.33 (-35.09 to -19.3)
**District of Columbia**	95.58 (78.81 to 114.32)	49.5 (41.41 to 58.2)	-48.21 (-51.84 to -44.78)	1115.5 (997.03 to 1249.61)	972.21 (881.56 to 1075.8)	-12.85 (-19.56 to -4.82)	29.58 (25.82 to 31.91)	14.33 (11.22 to 17.07)	-51.56 (-58.79 to -44.05)
**Florida**	80.92 (65.98 to 99.75)	55.81 (46.98 to 66.33)	-31.03 (-36 to -25.49)	984.53 (876.26 to 1083.51)	988.19 (901 to 1084)	0.37 (-6.96 to 8.28)	22.44 (18.82 to 24.44)	19.22 (14.58 to 22.42)	-14.36 (-25.62 to -3.57)
**Georgia**	102.58 (84.56 to 123.78)	61.54 (52.23 to 72.37)	-40 (-43.77 to -35.37)	1135.99 (1012.69 to 1264.66)	1068.79 (977.88 to 1171.66)	-5.92 (-13.38 to 3.32)	38.42 (32.88 to 41.45)	21.58 (17.65 to 25.08)	-43.84 (-50.76 to -36.03)
**Hawaii**	86.23 (70.28 to 104.27)	52.02 (43.4 to 61.64)	-39.67 (-44.21 to -35.38)	933.56 (829.27 to 1042.27)	841.75 (770.86 to 920.9)	-9.84 (-16.89 to -2.08)	25.28 (21.09 to 27.55)	14.33 (10.7 to 17.18)	-43.32 (-51.12 to -35.26)
**Idaho**	88.95 (72.77 to 107.44)	56.31 (47.25 to 66.28)	-36.69 (-41.29 to -31.97)	943.19 (848.72 to 1048.7)	857.88 (782.58 to 934.98)	-9.04 (-15.95 to -1.41)	32.03 (26.9 to 35)	17.77 (14.39 to 20.35)	-44.51 (-51.22 to -37.89)
**Illinois**	93.85 (77.64 to 113.52)	56.3 (47.3 to 66.24)	-40.01 (-43.99 to -35.44)	1017.61 (900.69 to 1133.27)	946.02 (865.68 to 1035.94)	-7.03 (-13.73 to 1.63)	29.11 (25.2 to 31.29)	18.36 (14.8 to 21.45)	-36.95 (-45.08 to -28.74)

**Indiana**	94.63 (78.18 to 113.75)	60.26 (50.58 to 71.04)	-36.33 (-41.27 to -31.08)	1046.12 (933.81 to 1160.65)	1056.56 (959.28 to 1155.25)	1 (-8.18 to 8.62)	33.89 (29.21 to 36.47)	20.62 (17 to 23.66)	-39.15 (-46.5 to -31.88)
**Iowa**	80.13 (64.03 to 98.47)	51.96 (44 to 61.07)	-35.16 (-40.52 to -29.19)	861.75 (769.77 to 958.5)	858.88 (786.35 to 943.31)	-0.33 (-7.91 to 7.85)	27.45 (23.37 to 30)	14.9 (11.79 to 17.49)	-45.73 (-52.74 to -38.07)
**Kansas**	88.06 (71.56 to 106.53)	58.5 (49.17 to 69.31)	-33.57 (-37.79 to -28.98)	976.9 (869.77 to 1079.86)	982.57 (902.58 to 1075.53)	0.58 (-5.69 to 8.93)	28.68 (24.35 to 31.31)	17.44 (14.17 to 20.44)	-39.2 (-47.15 to -30.96)
**Kentucky**	95 (78.23 to 113.96)	61.16 (51.4 to 72.2)	-35.62 (-40.17 to -30.45)	1109.53 (985.05 to 1222.81)	1159.67 (1043.06 to 1273.25)	4.52 (-4.47 to 13.11)	34.03 (29.18 to 36.72)	21.04 (17.16 to 24.48)	-38.16 (-45.69 to -29.91)
**Louisiana**	98.11 (80.62 to 118.02)	63.03 (53.13 to 74.18)	-35.75 (-40.23 to -31)	1145.88 (1032.91 to 1281.12)	1161.58 (1057.86 to 1279.37)	1.37 (-5.52 to 8.75)	32.73 (28.2 to 35.19)	21.98 (18.24 to 25.27)	-32.84 (-40.42 to -23.51)
**Maine**	85.95 (70.26 to 104.74)	53.66 (44.91 to 63.41)	-37.57 (-42.5 to -32.49)	896.78 (798.59 to 995.18)	868.04 (795.24 to 944.29)	-3.21 (-10.35 to 5.26)	27.11 (23.28 to 29.56)	15.86 (12.81 to 18.17)	-41.49 (-48.25 to -34.77)
**Maryland**	90.84 (74.76 to 109.97)	62.61 (53.79 to 72.79)	-31.08 (-36.95 to -25.33)	1042.16 (937.45 to 1165.45)	1034.64 (949.49 to 1128.17)	-0.72 (-7.85 to 7.15)	29.09 (24.93 to 31.52)	18.76 (14.91 to 21.93)	-35.49 (-44.71 to -25.08)
**Massachusetts**	77.13 (62.83 to 95.03)	49.05 (40.62 to 58.72)	-36.41 (-41.38 to -30.84)	871.61 (782.15 to 970.35)	816.7 (746.17 to 894.87)	-6.3 (-12.9 to 0.93)	25.27 (21.26 to 27.5)	13.13 (10.34 to 15.4)	-48.04 (-55.24 to -40.98)

**Michigan**	89.38 (72.58 to 108.23)	59.46 (50.41 to 70)	-33.47 (-37.92 to -28.48)	1053.2 (930.4 to 1187.89)	1086.74 (989.8 to 1181.79)	3.18 (-4.32 to 12.11)	28.95 (24.88 to 31.31)	18.05 (14.64 to 20.8)	-37.63 (-45.54 to -29.65)
**Minnesota**	83.11 (67.55 to 100.56)	55.19 (46.32 to 65.08)	-33.59 (-38.93 to -27.97)	869.26 (775.26 to 981.13)	829.4 (754.73 to 898.59)	-4.59 (-12.21 to 4.27)	31.28 (26.79 to 34.03)	16.52 (13.05 to 19.1)	-47.17 (-54.27 to -40.48)
**Mississippi**	99.29 (81.61 to 119.37)	65.94 (55.97 to 77.26)	-33.59 (-37.51 to -28.85)	1181.67 (1058.07 to 1307.46)	1255.65 (1144.53 to 1390.95)	6.26 (-2.13 to 14.65)	34.95 (30.54 to 37.63)	23.69 (19.59 to 27.25)	-32.22 (-40.13 to -23.14)
**Missouri**	90.7 (74.43 to 109.13)	58.83 (49.52 to 69.1)	-35.14 (-39.35 to -30.37)	1025.68 (917.72 to 1147.76)	1042.85 (954.88 to 1145.65)	1.67 (-6.22 to 9.79)	29.16 (24.98 to 31.49)	18.06 (14.92 to 20.89)	-38.07 (-45.92 to -29.99)
**Montana**	89.44 (73.02 to 108.06)	55.38 (46.27 to 64.83)	-38.09 (-42.72 to -33.47)	966.95 (863.45 to 1072.69)	921.4 (838.74 to 997.07)	-4.71 (-11.48 to 2.85)	30.44 (25.73 to 33.43)	14.47 (11.6 to 16.87)	-52.45 (-58.32 to -45.98)
**Nebraska**	88.91 (72.47 to 107.64)	55.19 (46.48 to 64.88)	-37.92 (-42.72 to -33.28)	930.05 (834.34 to 1037.73)	873.06 (803.95 to 946.46)	-6.13 (-12.84 to 1.25)	29.64 (25.11 to 32.41)	16.34 (13.02 to 18.84)	-44.87 (-51.8 to -38.35)
**Nevada**	106.26 (87.41 to 128.39)	69.71 (59.05 to 81.67)	-34.4 (-39.01 to -29)	1111.43 (1008.56 to 1221.06)	1038.4 (942.16 to 1136.45)	-6.57 (-12.73 to 0.64)	30.18 (26.4 to 32.71)	16.69 (13.56 to 19.37)	-44.69 (-51.44 to -37.9)
**New Hampshire**	87.3 (70.98 to 105.83)	51.41 (42.99 to 61.39)	-41.11 (-45.22 to -36.58)	956.65 (844.86 to 1074.45)	838.14 (765.63 to 913.69)	-12.39 (-19.57 to -4.62)	28.33 (24.05 to 30.78)	15.08 (12.1 to 17.5)	-46.77 (-52.98 to -40.47)

**New Jersey**	82.1 (66.11 to 100.63)	51.13 (42.81 to 61.09)	-37.72 (-42.04 to -33.19)	947.66 (840.48 to 1061.48)	845.2 (777.96 to 917.46)	-10.81 (-18.15 to -3.55)	23.82 (20.52 to 25.84)	12.6 (9.9 to 14.72)	-47.13 (-54.83 to -40)
**New Mexico**	84.29 (69.53 to 100.73)	52.77 (43.97 to 62.46)	-37.4 (-41.6 to -33.21)	903.99 (804.74 to 1011.96)	894.4 (818.23 to 976)	-1.06 (-8.72 to 7.02)	25.36 (21.59 to 27.88)	14.49 (11.58 to 16.92)	-42.86 (-50.11 to -34.35)
**New York**	76.67 (61.68 to 93.59)	46.3 (38.91 to 54.65)	-39.62 (-44.53 to -34.32)	889.67 (794.34 to 986.57)	823.28 (751.76 to 905.61)	-7.46 (-14.48 to 0.14)	20.77 (17.96 to 22.46)	8.98 (7.1 to 10.66)	-56.74 (-63.1 to -50.43)
**North Carolina**	100.31 (82.38 to 121.27)	67.58 (56.98 to 79.52)	-32.63 (-37.59 to -27.39)	1152.37 (1028.82 to 1284.44)	1144.47 (1038.62 to 1246.56)	-0.69 (-8.45 to 6.62)	36.61 (31.37 to 39.81)	20.22 (16.23 to 23.42)	-44.79 (-51.84 to -37.75)
**North Dakota**	85.38 (70.59 to 102.4)	54.51 (46.15 to 64.77)	-36.16 (-40.66 to -31.37)	861.75 (770.27 to 949.95)	861.9 (792.44 to 934.73)	0.02 (-6.55 to 7.25)	27.96 (23.8 to 30.76)	13.72 (11.06 to 16.12)	-50.93 (-56.45 to -44.23)
**Ohio**	91.44 (74.72 to 110.71)	59.15 (50.26 to 69.85)	-35.31 (-39.81 to -30.56)	1036.21 (929.66 to 1155.15)	1053.19 (959.82 to 1150.21)	1.64 (-5.45 to 10.44)	29.26 (25.45 to 31.58)	20.82 (17.01 to 23.92)	-28.85 (-37.14 to -19.86)
**Oklahoma**	93.71 (77.22 to 112.48)	62.96 (52.91 to 75)	-32.81 (-37.64 to -27.84)	1082.93 (974.68 to 1191.06)	1124.12 (1027.3 to 1232.75)	3.8 (-3.49 to 13.53)	33.34 (28.71 to 36.04)	18.83 (15.3 to 21.78)	-43.54 (-50.19 to -36.59)
**Oregon**	94.77 (77.49 to 114.24)	57.21 (47.78 to 67.91)	-39.64 (-44.04 to -34.68)	1004.09 (890.64 to 1115.11)	951.73 (863.58 to 1042.81)	-5.21 (-13.17 to 3.4)	33.78 (29.07 to 36.8)	18.53 (14.73 to 21.83)	-45.16 (-51.64 to -37.16)

**Pennsylvania**	92.18 (75.51 to 110.96)	54.61 (45.84 to 64.25)	-40.76 (-45.1 to -35.69)	1008.32 (907.12 to 1124.03)	967.75 (887.62 to 1050.15)	-4.02 (-11.31 to 4.26)	27.42 (23.69 to 29.73)	16.15 (12.89 to 18.78)	-41.09 (-48.52 to -33.35)
**Rhode Island**	81.76 (67.21 to 99.21)	48.3 (40.29 to 57.39)	-40.93 (-45.18 to -36.29)	887.36 (791.91 to 990.46)	805.17 (729.72 to 881.37)	-9.26 (-15.92 to -1.96)	22.88 (19.69 to 25.03)	12.4 (9.99 to 14.56)	-45.79 (-52.52 to -38.39)
**South Carolina**	106.54 (87.97 to 128.39)	64.09 (53.84 to 74.58)	-39.85 (-44.82 to -35.25)	1173.05 (1043.98 to 1313.78)	1129.18 (1031.61 to 1240.07)	-3.74 (-10.74 to 4.34)	40.71 (35.74 to 44.06)	22.29 (18.39 to 25.54)	-45.26 (-52.41 to -38.65)
**South Dakota**	83.54 (67.21 to 100.5)	54.3 (45.71 to 64.01)	-35 (-39.45 to -30.35)	897.62 (803.56 to 996.87)	866.74 (792.24 to 953.01)	-3.44 (-10.42 to 4.68)	26.69 (22.53 to 29.21)	13.76 (11.07 to 15.82)	-48.46 (-54.26 to -42.2)
**Tennessee**	99.38 (80.98 to 119.17)	63.5 (54.15 to 74.26)	-36.1 (-41.17 to -30.85)	1168.41 (1044.49 to 1288.71)	1166.24 (1059.66 to 1278.21)	-0.19 (-8.24 to 7.48)	37.91 (32.98 to 40.87)	20.1 (16.74 to 23.2)	-46.97 (-52.94 to -40.47)
**Texas**	92.5 (75.94 to 110.85)	58.77 (49.53 to 69.04)	-36.46 (-41.14 to -31.23)	1059.07 (950.4 to 1178.51)	997.58 (908.87 to 1085.93)	-5.81 (-12.73 to 2.64)	31.45 (27.07 to 34.1)	19.24 (15.83 to 22.16)	-38.81 (-45.47 to -31.56)
**Utah**	86.91 (71.11 to 104.85)	55.47 (46.72 to 65.29)	-36.17 (-41.05 to -31.67)	936.21 (837 to 1042.3)	866.28 (792.72 to 945.26)	-7.47 (-13.72 to -0.35)	31.8 (27.1 to 34.48)	19.03 (15.75 to 21.75)	-40.15 (-46.62 to -33.29)
**Vermont**	86.68 (70.93 to 105.65)	50.82 (42.07 to 60.44)	-41.37 (-45.49 to -36.89)	907.85 (806.81 to 1012.26)	827.44 (754.72 to 900.4)	-8.86 (-16.11 to -1.17)	27.8 (23.89 to 30.22)	14.96 (12.05 to 17.14)	-46.2 (-51.71 to -39.93)

**Virginia**	97.28 (80.01 to 117.19)	57.16 (47.72 to 67.46)	-41.24 (-45.45 to -36.64)	1032.78 (920.67 to 1158)	965.07 (884.37 to 1054.2)	-6.56 (-13.82 to 2.04)	35.54 (31.14 to 38.28)	18.94 (15.41 to 22.05)	-46.71 (-53.49 to -38.64)
**Washington**	85.24 (68.68 to 105.97)	52.76 (44.81 to 61.5)	-38.1 (-43.7 to -31.39)	918.72 (819.26 to 1020.88)	870.65 (796.14 to 947.35)	-5.23 (-11.81 to 2.45)	31.51 (27.06 to 34.16)	15.93 (12.43 to 18.5)	-49.45 (-56.38 to -42.92)
**West Virginia**	91.22 (75.16 to 110.36)	60.88 (51.34 to 71.5)	-33.26 (-37.98 to -27.72)	1029.15 (919.5 to 1137.06)	1086.23 (985.44 to 1190.99)	5.55 (-2.43 to 15.43)	29.29 (25.58 to 31.7)	18.64 (15.63 to 21.5)	-36.34 (-44.05 to -27.39)
**Wisconsin**	83.83 (67.53 to 101.58)	51.98 (44.18 to 60.65)	-37.99 (-43.48 to -32.03)	843.23 (754.61 to 936.75)	782.83 (717.29 to 860.31)	-7.16 (-13.59 to 0.95)	30.22 (25.74 to 32.77)	15.84 (12.6 to 18.5)	-47.6 (-54.58 to -39.87)
**Wyoming**	87.65 (71.11 to 106.47)	52.96 (44.83 to 62.99)	-39.58 (-43.87 to -35.19)	934.6 (838.03 to 1038.7)	848.27 (773.97 to 932.28)	-9.24 (-16.26 to -2.06)	29.43 (25.02 to 32.42)	15.74 (12.81 to 18.13)	-46.54 (-52.29 to -40.43)

2.4 Table S2b: Age-standardized rate and percentage change of DALYs, YLDs, and YLLs for ischemic stroke over time, both sexes, by regions in US, 1990-2021

Ischemic stroke	DALYs			YLDs			YLLs		
**Location**	**1990**	**2021**	**change, %**	**1990**	**2021**	**change, %**	**1990**	**2021**	**change, %**
**Alabama**	711.58 (642.38 to 777.4)	552.94 (478.81 to 626.39)	-22.29 (-29.52 to -14.5)	167.97 (119.95 to 215.32)	173.12 (126.55 to 220.57)	3.07 (-6.09 to 12.27)	543.61 (485.7 to 582.37)	379.82 (320.38 to 439.24)	-30.13 (-39.57 to -20.09)
**Alaska**	610.09 (545.12 to 669.69)	357.93 (308.23 to 402.8)	-41.33 (-45.93 to -36.22)	142.14 (101.95 to 188.12)	125.62 (90.72 to 162.17)	-11.63 (-19.1 to -2.77)	467.95 (413.88 to 504.41)	232.32 (191.18 to 268.34)	-50.35 (-55.8 to -43.76)
**Arizona**	464.7 (413.79 to 513.83)	327.24 (280.5 to 374.24)	-29.58 (-36.57 to -22.71)	125.43 (88.95 to 160.64)	118.76 (86.52 to 151.45)	-5.31 (-13.22 to 4.51)	339.27 (297.78 to 365.94)	208.48 (174.71 to 242.79)	-38.55 (-47.19 to -29.41)
**Arkansas**	724.22 (652.62 to 794.35)	450.42 (379.2 to 509.97)	-37.81 (-44.35 to -31.59)	168.83 (120.24 to 222.68)	171.89 (124.05 to 217.12)	1.81 (-7.77 to 12.63)	555.39 (492.09 to 590.9)	278.53 (233.34 to 318.99)	-49.85 (-56.61 to -42.18)
**California**	548.01 (490.27 to 601.99)	327.09 (278.56 to 370.01)	-40.31 (-45.93 to -35.29)	129.03 (93.07 to 164.2)	115.99 (83.19 to 149.01)	-10.11 (-18.49 to -0.02)	418.98 (368.48 to 449.43)	211.1 (171.16 to 244.22)	-49.61 (-55.98 to -43.16)
**Colorado**	466.2 (411.18 to 509.8)	319.37 (269.17 to 363.21)	-31.5 (-38.09 to -24.48)	112.57 (79.21 to 143.21)	103.07 (74.62 to 132.46)	-8.44 (-16.88 to 0.52)	353.62 (306.53 to 379.76)	216.3 (168.05 to 252.91)	-38.83 (-47.67 to -29.77)

**Connecticut**	456.53 (405.63 to 506.53)	270.93 (228.68 to 312.57)	-40.65 (-46.43 to -34.43)	127.13 (89.66 to 162.21)	108 (78.17 to 138.41)	-15.05 (-22.63 to -6.69)	329.4 (283.06 to 355.27)	162.93 (130.91 to 193.85)	-50.54 (-57.7 to -42.92)
**Delaware**	525.24 (468.02 to 575.29)	379.79 (332.51 to 428.74)	-27.69 (-33.17 to -21.51)	139.43 (98.31 to 179.88)	129.32 (92.31 to 164.95)	-7.25 (-14.41 to 2.05)	385.81 (341.44 to 415.64)	250.47 (210.64 to 285.72)	-35.08 (-42.22 to -27.47)
**District of Columbia**	622.1 (563.34 to 682.53)	336.68 (288.09 to 391.04)	-45.88 (-51.4 to -40.02)	156.04 (112.98 to 203.74)	134.6 (98 to 173.47)	-13.74 (-21.39 to -5.05)	466.06 (424.31 to 496.01)	202.08 (162.6 to 238.79)	-56.64 (-63.19 to -49.21)
**Florida**	461.17 (407.91 to 508.79)	385.86 (324.77 to 442.39)	-16.33 (-24.25 to -8.45)	137.41 (98.56 to 174.85)	134.51 (97.48 to 172.37)	-2.11 (-10.28 to 7.59)	323.76 (284.35 to 347.44)	251.35 (196.71 to 293.45)	-22.37 (-33.18 to -11.74)
**Georgia**	720.68 (649.16 to 785.35)	442.06 (380.16 to 504.9)	-38.66 (-44.8 to -31.84)	159.22 (112.86 to 205.73)	146.6 (107.5 to 185.1)	-7.93 (-16.39 to 2.18)	561.46 (500.65 to 601.22)	295.46 (245.11 to 343.25)	-47.38 (-54.52 to -39.43)
**Hawaii**	485.14 (426.68 to 539)	306.19 (260.55 to 354.03)	-36.89 (-43.12 to -30.41)	131.35 (92.8 to 170.17)	116.77 (84.82 to 150.69)	-11.1 (-18.85 to -2.44)	353.79 (306.29 to 381.42)	189.43 (149.81 to 225.05)	-46.46 (-54.15 to -38.65)
**Idaho**	566.04 (495.37 to 621.53)	344.68 (297.58 to 393.37)	-39.11 (-44.6 to -33.38)	132.62 (93.38 to 174.24)	118.66 (84.68 to 152.06)	-10.52 (-18.4 to -1.22)	433.42 (376.34 to 470.35)	226.02 (188.08 to 259.77)	-47.85 (-54.73 to -41.06)
**Illinois**	560.76 (500.22 to 615.8)	374.07 (322.01 to 430.49)	-33.29 (-39.5 to -27.04)	142.88 (102.31 to 186.26)	130.48 (93.85 to 167.19)	-8.67 (-16.75 to 0.63)	417.88 (373.6 to 445.78)	243.58 (201 to 283.39)	-41.71 (-49.49 to -33.13)

**Indiana**	616.68 (551.81 to 674.46)	418.1 (361 to 470.83)	-32.2 (-38.21 to -25.69)	145.38 (105 to 186.63)	142.63 (103.81 to 182.42)	-1.89 (-11.28 to 7.63)	471.3 (420.23 to 502.17)	275.47 (234.06 to 315.7)	-41.55 (-48.84 to -33.46)
**Iowa**	495.61 (443.3 to 546.3)	314.11 (265.92 to 356.81)	-36.62 (-42.8 to -29.91)	121.45 (86.77 to 158.13)	118.51 (85.55 to 149.98)	-2.42 (-11.17 to 6.96)	374.16 (327.65 to 402.69)	195.6 (160.11 to 227.07)	-47.72 (-54.84 to -39.91)
**Kansas**	533.49 (472.5 to 589.15)	363.3 (314.33 to 414.17)	-31.9 (-38.62 to -25.11)	137.66 (99.26 to 174.05)	134.88 (97.7 to 169.88)	-2.02 (-9.39 to 7.53)	395.83 (346.51 to 428.88)	228.43 (189.8 to 268.07)	-42.29 (-50.31 to -33.98)
**Kentucky**	636.87 (569.15 to 695.87)	445.81 (383.31 to 511.57)	-30 (-36.45 to -22.66)	154.4 (109.12 to 202.01)	156.52 (114.09 to 198.68)	1.37 (-8.59 to 11.74)	482.47 (429.29 to 514.16)	289.29 (239.29 to 334.39)	-40.04 (-48.08 to -31.2)
**Louisiana**	647.57 (590.28 to 709.5)	463.55 (402.62 to 524.86)	-28.42 (-34.64 to -21.51)	159.84 (116.46 to 207.43)	157.52 (114.46 to 199.3)	-1.45 (-9.36 to 7.12)	487.74 (436.78 to 521.29)	306.02 (258.66 to 352.31)	-37.26 (-44.77 to -27.7)
**Maine**	502.99 (445.14 to 554.97)	322.04 (277.47 to 368.06)	-35.97 (-41.57 to -30.27)	125.94 (91.08 to 162.93)	118.64 (86.93 to 150.79)	-5.8 (-13.97 to 4.16)	377.05 (335.42 to 406.17)	203.4 (169.26 to 233.02)	-46.05 (-52.77 to -39.19)
**Maryland**	552.68 (492.31 to 607.19)	387.9 (335.05 to 444.39)	-29.82 (-36.49 to -22.66)	146.82 (106.78 to 187.96)	142.73 (102.89 to 182.77)	-2.78 (-11.11 to 6.86)	405.87 (359.61 to 435.58)	245.16 (200.29 to 286.81)	-39.59 (-48.33 to -29.69)
**Massachusetts**	469 (419.56 to 515.3)	273.8 (229.03 to 316.02)	-41.62 (-47.74 to -35.8)	121.76 (86.26 to 158.26)	111.5 (81.07 to 139.54)	-8.42 (-16.71 to -0.08)	347.24 (303.59 to 374.2)	162.3 (129.82 to 189.45)	-53.26 (-60.12 to -46.59)

**Michigan**	562.15 (507.62 to 615.96)	382.02 (327.31 to 435.82)	-32.04 (-38.39 to -25.82)	147.66 (105.08 to 190.93)	148.02 (108.09 to 187.59)	0.24 (-8.63 to 10.15)	414.49 (369.71 to 441.87)	234 (194.24 to 268.41)	-43.54 (-51.15 to -35.93)
**Minnesota**	535.86 (479.65 to 589.13)	321.86 (272.42 to 368.41)	-39.94 (-45.64 to -33.93)	122.43 (86.37 to 155.95)	114.14 (82 to 145.44)	-6.77 (-15.26 to 4)	413.43 (367.19 to 446.06)	207.72 (169.92 to 239.34)	-49.76 (-56.94 to -42.78)
**Mississippi**	689.53 (618.64 to 754.42)	519.16 (442.08 to 589.17)	-24.71 (-32.65 to -15.94)	165.76 (118.01 to 216.28)	172.01 (124.91 to 218.55)	3.77 (-5.93 to 13.76)	523.77 (472.55 to 559)	347.15 (296.34 to 402.16)	-33.72 (-42.28 to -23.9)
**Missouri**	554.18 (495.34 to 612.24)	386.91 (335.09 to 439.89)	-30.18 (-37.15 to -23.67)	144.14 (104.76 to 187.86)	142.47 (102.94 to 180.42)	-1.16 (-10.2 to 7.98)	410.04 (363.15 to 440.14)	244.44 (207.4 to 282.48)	-40.39 (-48.48 to -32.11)
**Montana**	552.18 (493.14 to 608.81)	316.32 (274.39 to 360)	-42.71 (-48.01 to -36.67)	136.15 (96.97 to 177.3)	126.48 (91.53 to 162.2)	-7.1 (-14.6 to 1.68)	416.03 (363.53 to 452.02)	189.85 (157.05 to 220.47)	-54.37 (-60.26 to -47.38)
**Nebraska**	535.27 (474.11 to 583.07)	335.85 (287.25 to 381.85)	-37.26 (-43.4 to -32.08)	130.87 (92.96 to 166.93)	119.96 (86.57 to 153.87)	-8.34 (-15.75 to 0.21)	404.39 (356.93 to 436.76)	215.88 (178.3 to 249.22)	-46.62 (-53.73 to -39.82)
**Nevada**	585.09 (525.32 to 644.43)	371.9 (316.72 to 418.65)	-36.44 (-41.92 to -30.92)	156.7 (112 to 201.01)	143.69 (104.79 to 184.2)	-8.3 (-15.63 to 0.7)	428.39 (385.64 to 458.45)	228.2 (191.57 to 262.6)	-46.73 (-53.43 to -39.58)
**New Hampshire**	520.75 (463.77 to 569.43)	300.49 (254.77 to 340.04)	-42.3 (-47.49 to -37.06)	134.42 (95.58 to 172.51)	114.66 (83.24 to 146.49)	-14.7 (-22.72 to -6.51)	386.33 (340.94 to 415.98)	185.83 (151.1 to 213.92)	-51.9 (-57.95 to -45.54)

**New Jersey**	474 (424.88 to 521.57)	279.84 (234.71 to 320.69)	-40.96 (-46.52 to -35.62)	132.81 (93.97 to 170.8)	116.24 (84.86 to 149.52)	-12.47 (-20.57 to -4.28)	341.2 (305.55 to 366.3)	163.6 (131.73 to 191.02)	-52.05 (-59.47 to -45)
**New Mexico**	478.44 (429.11 to 527.57)	319.57 (270.83 to 368.4)	-33.21 (-39.45 to -26.04)	126.76 (91.2 to 165.52)	122.14 (89.42 to 156.53)	-3.65 (-12.03 to 5.65)	351.67 (308.74 to 381.86)	197.42 (160.81 to 229.61)	-43.86 (-51.63 to -34.9)
**New York**	428.86 (383.52 to 474.34)	235.7 (197.13 to 272.96)	-45.04 (-50.51 to -39.85)	124.54 (87.54 to 161.73)	112.19 (81.24 to 143.98)	-9.92 (-17.95 to -1.05)	304.32 (272.21 to 324.95)	123.51 (100.64 to 145.22)	-59.41 (-65.77 to -53.57)
**North Carolina**	684.21 (613.39 to 744.38)	429.25 (370.61 to 484.77)	-37.26 (-42.57 to -31.46)	160.94 (114.27 to 206.64)	155.03 (113.22 to 197.8)	-3.67 (-11.95 to 4.89)	523.27 (465.45 to 560.82)	274.22 (225.34 to 315.28)	-47.6 (-54.59 to -40.4)
**North Dakota**	498.14 (440.4 to 549.5)	300.08 (258.24 to 342.06)	-39.76 (-45.1 to -34.15)	121.08 (87.31 to 158.54)	118.5 (85.61 to 153.36)	-2.13 (-9.8 to 6.79)	377.05 (331.78 to 409.97)	181.58 (152.74 to 210.91)	-51.84 (-57.63 to -44.73)
**Ohio**	557.93 (504.11 to 611.57)	416.05 (355.33 to 467.43)	-25.43 (-31.98 to -18.76)	145.42 (104.12 to 190.02)	143.13 (102.69 to 184.21)	-1.58 (-9.95 to 7.77)	412.51 (371.55 to 440.45)	272.92 (228.81 to 311.99)	-33.84 (-41.96 to -24.75)
**Oklahoma**	622.14 (559.43 to 677.14)	419.29 (362.72 to 482.94)	-32.61 (-38.91 to -26.62)	152.11 (109.41 to 196.69)	153.06 (111.16 to 195.12)	0.63 (-7.49 to 11.63)	470.03 (420.56 to 502.93)	266.23 (222.9 to 307.8)	-43.36 (-50.39 to -35.74)
**Oregon**	600.65 (535.65 to 657.21)	369.04 (316.19 to 429.44)	-38.56 (-44.21 to -31.88)	141.73 (99.58 to 182.79)	131.61 (94.69 to 168.85)	-7.14 (-15.49 to 2.08)	458.93 (408.65 to 494.71)	237.43 (192.85 to 279.54)	-48.26 (-54.72 to -40.43)

**Pennsylvania**	530.03 (478.87 to 580.76)	340.26 (287.72 to 391.53)	-35.8 (-41.91 to -29.64)	141.2 (102.57 to 181.02)	131.7 (96.35 to 166.91)	-6.73 (-14.65 to 2.75)	388.83 (346.89 to 415.07)	208.56 (172.44 to 241.58)	-46.36 (-53.22 to -39.31)
**Rhode Island**	442.99 (387.55 to 490.18)	268.44 (225.37 to 309.81)	-39.4 (-45.22 to -33.61)	125.07 (88.02 to 160.53)	110.78 (79.34 to 141.24)	-11.42 (-18.99 to -2.45)	317.92 (282.45 to 344.37)	157.66 (128.82 to 184.99)	-50.41 (-57.04 to -43.24)
**South Carolina**	772.85 (700.77 to 841.03)	457.01 (387.65 to 523.55)	-40.87 (-47.33 to -34.96)	164.31 (116.68 to 213.49)	153.53 (110.35 to 195.09)	-6.56 (-14.39 to 3.49)	608.54 (550.24 to 654.73)	303.48 (253.57 to 346.28)	-50.13 (-57.12 to -43.03)
**South Dakota**	495.19 (440 to 543.76)	302.8 (260.05 to 344.61)	-38.85 (-44.24 to -33.77)	126.31 (89.48 to 162.46)	119.44 (86.44 to 152.88)	-5.43 (-14.01 to 3.77)	368.88 (323.85 to 397.4)	183.36 (153.59 to 208.92)	-50.29 (-56.01 to -44.09)
**Tennessee**	705.97 (638.55 to 776.51)	440.98 (380.25 to 503.7)	-37.54 (-43 to -31.77)	163.94 (117.98 to 214.02)	158.54 (115.31 to 203.39)	-3.29 (-11.94 to 5.2)	542.03 (489.14 to 577.95)	282.44 (239 to 325.7)	-47.89 (-54.08 to -41.02)
**Texas**	593.65 (533.26 to 649.38)	402.33 (347.55 to 457.7)	-32.23 (-38.17 to -26.16)	149.19 (105.23 to 191.53)	137.47 (100.09 to 175.57)	-7.85 (-15.79 to 1.92)	444.46 (396.35 to 476.54)	264.86 (224.86 to 302.87)	-40.41 (-47.42 to -32.55)
**Utah**	556.83 (495.72 to 612.87)	357.79 (311.19 to 406.38)	-35.75 (-41.04 to -29.49)	132.33 (95.05 to 172.02)	119.58 (86.66 to 152.32)	-9.63 (-16.74 to -1.51)	424.51 (372.6 to 456.04)	238.21 (199.87 to 272.91)	-43.88 (-50.39 to -36.81)
**Vermont**	510.64 (454.65 to 560.98)	299.81 (258.71 to 340.17)	-41.29 (-45.91 to -36.14)	127.39 (90.66 to 166.32)	114.14 (83.17 to 145.72)	-10.4 (-18.33 to -1.63)	383.25 (338.27 to 412.12)	185.67 (153.77 to 212.05)	-51.55 (-56.75 to -45.22)

**Virginia**	645.88 (586.36 to 701.9)	380.52 (323.82 to 433.04)	-41.09 (-47.21 to -35.18)	145.54 (103.17 to 186.71)	133.27 (97.27 to 171.04)	-8.43 (-16.94 to -0.03)	500.34 (451.67 to 534.76)	247.25 (204.56 to 287.45)	-50.58 (-57.57 to -43.02)
**Washington**	550.77 (485.95 to 605)	321.59 (271.93 to 369.17)	-41.61 (-47.61 to -35.9)	129.62 (91.7 to 166.29)	120.22 (87.25 to 151.99)	-7.26 (-15 to 1.86)	421.14 (371.26 to 452.1)	201.37 (163.7 to 233.85)	-52.18 (-58.79 to -45.58)
**West Virginia**	564.75 (505.02 to 619.49)	407.84 (351.31 to 465.42)	-27.78 (-34.53 to -20.32)	144.62 (103.11 to 186.14)	147.17 (106.8 to 187.4)	1.76 (-6.49 to 13.01)	420.12 (378.55 to 450.9)	260.67 (225.07 to 299.46)	-37.95 (-46.05 to -29.13)
**Wisconsin**	527.32 (470.22 to 574.18)	310.07 (263.72 to 354.89)	-41.2 (-46.98 to -35.02)	118.4 (84.49 to 153.39)	107.84 (77.98 to 138.02)	-8.92 (-16.23 to 0.76)	408.92 (360.08 to 438.24)	202.23 (163.14 to 234.53)	-50.55 (-57.54 to -42.79)
**Wyoming**	529.9 (471.45 to 587.4)	325.49 (278.13 to 371.67)	-38.58 (-43.75 to -33.54)	131.5 (94.68 to 170.94)	116.93 (83.89 to 149.65)	-11.08 (-18.9 to -2.43)	398.4 (350.78 to 432.07)	208.56 (175.31 to 236.91)	-47.65 (-53.69 to -41.44)

2.5 Table S3a: Age-standardized rate and percentage change of incidence, prevalence, and mortality for intracerebral hemorrhage over time, both sexes, by regions in US, 1990-2021

Intracerebral hemorrhage	Incidence			Prevalence			Mortality		
**Location**	**1990**	**2021**	**change, %**	**1990**	**2021**	**change, %**	**1990**	**2021**	**change, %**
**Alabama**	21.29 (17.88 to 24.47)	17.4 (15.06 to 19.74)	-18.24 (-24.01 to -12.3)	187.73 (166.59 to 211.12)	223.96 (200.07 to 249.95)	19.3 (10.62 to 27.71)	15.42 (14.25 to 16.44)	14.3 (12.21 to 16.77)	-7.27 (-21.03 to 8.21)
**Alaska**	17.63 (14.72 to 20.55)	13.05 (11.12 to 14.9)	-25.97 (-31.02 to -19.98)	124.25 (110.67 to 140.55)	133.42 (119.49 to 148.39)	7.38 (1.29 to 13.57)	10.3 (9.39 to 11.1)	8.82 (7.53 to 10.25)	-14.41 (-24.95 to -1.64)
**Arizona**	15.42 (12.71 to 18.05)	10.9 (9.45 to 12.33)	-29.32 (-34.66 to -22.12)	123.41 (108.84 to 139.01)	126.99 (113.78 to 140.56)	2.9 (-4.23 to 9.82)	9.02 (8.25 to 9.59)	7.81 (6.65 to 9.03)	-13.4 (-26.16 to 0.3)
**Arkansas**	19.3 (15.87 to 22.4)	16.15 (13.93 to 18.44)	-16.32 (-22.69 to -9.11)	162.24 (144.45 to 182.14)	190.21 (170.11 to 209.44)	17.24 (10.36 to 25.02)	14.36 (13.26 to 15.27)	12.27 (10.35 to 14.32)	-14.56 (-27.44 to -0.88)
**California**	17.18 (14.15 to 20.19)	12.42 (10.58 to 14.21)	-27.72 (-33.18 to -21.39)	142.85 (126.8 to 159.61)	146.94 (131.15 to 162.63)	2.87 (-4.55 to 9.12)	10.93 (10.05 to 11.64)	8.96 (7.58 to 10.28)	-17.95 (-29.25 to -6.44)
**Colorado**	14.89 (12.16 to 17.5)	11.1 (9.59 to 12.64)	-25.45 (-32.03 to -17.63)	118.42 (104.18 to 132.93)	124.83 (111.69 to 138.49)	5.41 (-1.04 to 12.07)	8.9 (8.04 to 9.54)	7.53 (6.04 to 8.84)	-15.35 (-28.62 to -0.34)

**Connecticut**	17.38 (14.49 to 20.18)	11.07 (9.42 to 12.84)	-36.3 (-41.07 to -31.06)	130.62 (115.63 to 146.14)	124.69 (111.02 to 137.47)	-4.54 (-9.59 to 0.94)	9.15 (8.32 to 9.79)	7.22 (5.95 to 8.47)	-21.05 (-34.14 to -7.34)
**Delaware**	18.44 (15.31 to 21.42)	12.96 (11.11 to 14.79)	-29.73 (-34.14 to -24.26)	143.83 (127.68 to 161.7)	151.16 (135.32 to 166.15)	5.09 (-1.86 to 12.2)	10.57 (9.65 to 11.39)	10.27 (8.94 to 11.73)	-2.84 (-14.14 to 10.72)
**District of Columbia**	22.74 (18.87 to 26.45)	12.85 (11.11 to 14.65)	-43.5 (-47.14 to -39.64)	182.09 (160.78 to 203.62)	160.46 (143.33 to 178.92)	-11.88 (-17.79 to -5.96)	17.72 (16.3 to 18.81)	9.98 (8.27 to 11.76)	-43.71 (-52.83 to -33.74)
**Florida**	16.23 (13.44 to 19.05)	12.22 (10.52 to 13.8)	-24.74 (-30.78 to -18.09)	144.16 (127.63 to 161.73)	155.17 (139.04 to 173.41)	7.63 (0.83 to 14.37)	9.52 (8.7 to 10.09)	8.5 (7 to 9.94)	-10.69 (-25.28 to 3.43)
**Georgia**	21.34 (17.54 to 24.92)	15.58 (13.41 to 17.71)	-26.98 (-31.7 to -21.07)	170.61 (151.34 to 190.31)	183.87 (164.54 to 205.57)	7.77 (1.7 to 14.74)	15.16 (14.04 to 16.06)	12.03 (10.16 to 13.91)	-20.64 (-32.7 to -8.24)
**Hawaii**	20.27 (16.89 to 23.72)	13.54 (11.53 to 15.56)	-33.21 (-37.46 to -28.27)	159.09 (140.05 to 178.83)	158.6 (141.89 to 175.2)	-0.31 (-6.67 to 6.46)	13.29 (12 to 14.24)	9.58 (7.95 to 11.25)	-27.92 (-38.01 to -15.45)
**Idaho**	16.25 (13.4 to 19.06)	12.19 (10.41 to 13.86)	-25.02 (-30.38 to -18.59)	125.14 (110.01 to 140.39)	131.89 (118.12 to 146.24)	5.39 (-0.6 to 11.85)	9.1 (8.21 to 9.79)	8.13 (6.85 to 9.27)	-10.68 (-23.55 to 2.68)
**Illinois**	19.38 (16.17 to 22.7)	13.05 (11.12 to 15)	-32.64 (-36.87 to -27.62)	151.75 (133.51 to 169.02)	152.87 (136.83 to 169.42)	0.74 (-5.79 to 7.44)	11.59 (10.71 to 12.37)	10.18 (8.69 to 11.8)	-12.21 (-24.89 to 1.23)

**Indiana**	18.53 (15.4 to 21.6)	14.33 (12.37 to 16.29)	-22.68 (-27.49 to -16.14)	147.53 (131.4 to 165.09)	166.7 (149.21 to 185.7)	13 (6.45 to 20.19)	11.66 (10.79 to 12.42)	11.75 (9.96 to 13.45)	0.7 (-12.85 to 15.41)
**Iowa**	14.63 (11.95 to 17.34)	10.98 (9.31 to 12.54)	-24.91 (-31.61 to -16.89)	114.49 (101.73 to 128.6)	123.25 (110.28 to 135.94)	7.64 (1.29 to 14.25)	8.66 (7.92 to 9.36)	9.04 (7.56 to 10.49)	4.33 (-11.65 to 22.54)
**Kansas**	16.97 (14.17 to 19.78)	13.15 (11.27 to 15.06)	-22.53 (-27.45 to -16.55)	135.16 (118.99 to 151.7)	150.06 (134.77 to 166.44)	11.02 (3.98 to 18.64)	9.74 (8.83 to 10.44)	9.75 (8.18 to 11.41)	0.12 (-15.23 to 17.87)
**Kentucky**	18.68 (15.56 to 21.88)	14.99 (12.98 to 16.98)	-19.77 (-25.18 to -13.6)	156.71 (138.42 to 176.12)	181.85 (161.35 to 201.66)	16.04 (8.61 to 24.1)	12.18 (11.26 to 12.93)	12.89 (10.83 to 15.01)	5.84 (-9.07 to 23.61)
**Louisiana**	20.7 (17.07 to 24.05)	15.92 (13.75 to 18.26)	-23.12 (-28.01 to -16.81)	171.94 (151.71 to 192.03)	197.5 (176.18 to 221.2)	14.87 (7.03 to 23.27)	13.81 (12.93 to 14.59)	13.65 (11.71 to 15.79)	-1.12 (-13.75 to 14.91)
**Maine**	16.63 (13.83 to 19.41)	12.19 (10.38 to 13.99)	-26.73 (-32.22 to -20.46)	124.06 (110.03 to 140.04)	134.87 (120.23 to 148.32)	8.72 (2.19 to 14.8)	9.4 (8.57 to 10.12)	7.96 (6.66 to 9.2)	-15.39 (-27.37 to -1.86)
**Maryland**	17.99 (14.78 to 21.11)	12.91 (11.03 to 14.62)	-28.21 (-34.11 to -21.17)	143.75 (126.55 to 161.1)	149.27 (134.12 to 165.17)	3.84 (-3.32 to 10.29)	11.82 (10.9 to 12.58)	10.04 (8.33 to 11.68)	-15.06 (-29.02 to -0.48)
**Massachusetts**	14.65 (11.95 to 17.23)	10.26 (8.66 to 11.81)	-29.97 (-35.71 to -23.66)	124.45 (110.23 to 139.62)	123.52 (110.06 to 136.61)	-0.75 (-6.77 to 5.94)	9.31 (8.48 to 9.99)	7.13 (5.86 to 8.31)	-23.41 (-35.85 to -10.68)

**Michigan**	16.74 (13.82 to 19.53)	12.62 (10.88 to 14.42)	-24.64 (-30.2 to -16.84)	141.38 (125.74 to 158.09)	150.75 (135.52 to 167.8)	6.63 (0.29 to 14.13)	10.73 (9.84 to 11.47)	9.22 (7.84 to 10.63)	-14.1 (-26.01 to -0.16)
**Minnesota**	16.15 (13.28 to 18.92)	12.45 (10.63 to 14.25)	-22.88 (-28.42 to -16.76)	124.98 (111.88 to 140.17)	133.87 (120.38 to 148.14)	7.12 (1.11 to 13.34)	9.66 (8.77 to 10.33)	8.89 (7.39 to 10.32)	-7.92 (-21.8 to 6.76)
**Mississippi**	21.13 (17.65 to 24.51)	17.38 (14.94 to 19.81)	-17.75 (-22.65 to -12.08)	183.31 (163.57 to 205.72)	218.62 (193.82 to 245.89)	19.26 (10.79 to 28.45)	14.92 (13.89 to 15.85)	16.66 (14.28 to 19.44)	11.62 (-4.24 to 31.01)
**Missouri**	18.44 (15.51 to 21.4)	13.97 (12 to 16.03)	-24.23 (-28.81 to -18.45)	146.44 (129.19 to 164.09)	161.6 (144.42 to 179.73)	10.35 (3.51 to 18.32)	11.28 (10.39 to 11.97)	11.14 (9.52 to 12.92)	-1.23 (-16.31 to 14.86)
**Montana**	16.68 (13.89 to 19.55)	11.9 (10.17 to 13.63)	-28.66 (-33.65 to -21.83)	129.5 (114.98 to 145.05)	134.06 (120.49 to 148.31)	3.52 (-2.05 to 9.33)	9.65 (8.8 to 10.36)	7.98 (6.8 to 9.28)	-17.22 (-28.52 to -3.11)
**Nebraska**	16.87 (14 to 19.56)	12.33 (10.63 to 14.02)	-26.92 (-31.85 to -20.75)	129.5 (115.27 to 144.04)	135.65 (121.99 to 149.78)	4.75 (-0.98 to 11.1)	9.52 (8.65 to 10.23)	9.22 (7.73 to 10.64)	-3.15 (-18.16 to 11.02)
**Nevada**	22.06 (18.39 to 25.69)	16.01 (13.73 to 18.3)	-27.43 (-32.61 to -21.94)	171.92 (153.13 to 192.5)	178.91 (161.54 to 198.53)	4.07 (-1.68 to 9.93)	12.42 (11.39 to 13.24)	9.65 (8.16 to 11.1)	-22.36 (-33.76 to -10.17)
**New Hampshire**	16.67 (13.89 to 19.49)	11.46 (9.79 to 13.21)	-31.26 (-36.31 to -26.13)	135.11 (119 to 151.76)	135.73 (122.46 to 149.56)	0.45 (-5.16 to 6.95)	9.81 (8.95 to 10.57)	7.75 (6.48 to 9.05)	-20.97 (-32.54 to -8.8)

**New Jersey**	17.04 (13.9 to 19.9)	11.85 (10.07 to 13.69)	-30.43 (-35.68 to -24.41)	144.72 (128.96 to 161.49)	141.64 (126.68 to 156.21)	-2.13 (-8.56 to 3.38)	10.29 (9.48 to 11)	8.58 (7.12 to 10.12)	-16.59 (-29.75 to -2.43)
**New Mexico**	16.51 (13.67 to 19.24)	11.39 (9.78 to 13.07)	-31.02 (-35.86 to -25.2)	125.84 (111.33 to 140.77)	131.26 (116.97 to 145.06)	4.31 (-1.7 to 11.35)	9.15 (8.29 to 9.79)	7.8 (6.56 to 9.12)	-14.76 (-27.1 to -0.48)
**New York**	16.35 (13.25 to 19.35)	10.46 (9 to 11.96)	-36.01 (-41.21 to -28.81)	138.87 (122.65 to 155.03)	130.91 (116.96 to 145.11)	-5.73 (-11.31 to 0.49)	9.87 (9.16 to 10.45)	6.58 (5.4 to 7.64)	-33.33 (-43.39 to -22.13)
**North Carolina**	19.35 (15.92 to 22.72)	15.23 (13.18 to 17.37)	-21.26 (-26.98 to -14.47)	161.25 (143.96 to 180.95)	177.6 (158.21 to 195.4)	10.14 (3.68 to 16.05)	13.68 (12.58 to 14.56)	11.63 (9.82 to 13.55)	-14.98 (-27.54 to -1.76)
**North Dakota**	16.65 (13.91 to 19.44)	12.55 (10.82 to 14.31)	-24.62 (-29.83 to -18.83)	119.06 (105.29 to 132.94)	135.6 (121.34 to 149.4)	13.9 (6.32 to 21.22)	9.47 (8.6 to 10.23)	8.33 (7.09 to 9.6)	-12.09 (-24.22 to 2.29)
**Ohio**	17.95 (15.08 to 21.01)	14.02 (12.09 to 16.06)	-21.87 (-27.03 to -15.62)	146.69 (131.15 to 164.73)	165.3 (148.08 to 183.06)	12.69 (5.34 to 19.71)	10.59 (9.66 to 11.25)	11.21 (9.44 to 12.91)	5.84 (-8.65 to 20.89)
**Oklahoma**	18.22 (15.16 to 21.3)	14.38 (12.38 to 16.3)	-21.04 (-26.17 to -15.46)	145.68 (130.24 to 162.07)	168.88 (151.47 to 187.73)	15.92 (8.73 to 23.08)	11.25 (10.26 to 12.03)	10.71 (9.19 to 12.49)	-4.82 (-17.98 to 11.85)
**Oregon**	17.47 (14.52 to 20.45)	12.63 (10.83 to 14.5)	-27.73 (-32.72 to -21.54)	133.74 (120.12 to 150.32)	138.35 (124.11 to 152.21)	3.44 (-2.52 to 9.79)	10.11 (9.25 to 10.82)	9.26 (7.79 to 10.88)	-8.49 (-22 to 8.09)

**Pennsylvania**	18.54 (15.6 to 21.52)	12.98 (11.02 to 14.7)	-29.97 (-34.72 to -24.53)	147.51 (130.19 to 165.5)	155.06 (139.29 to 171.6)	5.12 (-0.97 to 11.19)	10.72 (9.81 to 11.4)	9.38 (7.86 to 10.89)	-12.5 (-24.88 to 0.98)
**Rhode Island**	16.69 (13.83 to 19.41)	11.33 (9.7 to 12.96)	-32.1 (-36.07 to -27.29)	130.41 (114.78 to 146.45)	130.34 (116.41 to 143.48)	-0.05 (-5.31 to 5.83)	9.6 (8.71 to 10.26)	7.44 (6.21 to 8.74)	-22.55 (-33.48 to -10.57)
**South Carolina**	22.55 (18.73 to 26.12)	16.34 (14.12 to 18.57)	-27.54 (-32.7 to -22.13)	180.55 (160.31 to 201.19)	194.15 (174.23 to 215.46)	7.53 (0.39 to 14.55)	16.69 (15.58 to 17.62)	13.1 (11.06 to 15.1)	-21.54 (-33.19 to -9.02)
**South Dakota**	16.05 (13.39 to 18.85)	12.2 (10.41 to 13.9)	-24.03 (-29.4 to -18.25)	126.38 (111.15 to 142.7)	138.93 (124.75 to 152.44)	9.93 (3.68 to 17.4)	9.02 (8.2 to 9.69)	8.26 (7.16 to 9.39)	-8.42 (-19.91 to 4.09)
**Tennessee**	19.77 (16.62 to 22.93)	15.31 (13.19 to 17.46)	-22.54 (-27.49 to -16.64)	164.24 (145.59 to 183.31)	186.67 (164.99 to 207.89)	13.66 (6.16 to 20.58)	13.47 (12.35 to 14.35)	12.48 (10.68 to 14.37)	-7.35 (-19.04 to 5.81)
**Texas**	18.59 (15.48 to 21.73)	14.07 (12.16 to 16.14)	-24.3 (-29.6 to -18.57)	156.77 (138.65 to 175.63)	171.89 (154.16 to 191.11)	9.65 (2.63 to 16.57)	11.73 (10.91 to 12.41)	10.7 (9.24 to 12.26)	-8.77 (-20.67 to 3.99)
**Utah**	16.4 (13.74 to 19.18)	12 (10.24 to 13.8)	-26.82 (-32.72 to -20.6)	126.95 (112.83 to 142.21)	130.93 (117.24 to 145)	3.14 (-2.74 to 9.27)	9.81 (8.96 to 10.53)	8.57 (7.23 to 9.89)	-12.67 (-23.59 to 0.62)
**Vermont**	15.99 (13.29 to 18.9)	10.22 (8.75 to 11.9)	-36.06 (-40.74 to -30.47)	119.49 (106.43 to 134.27)	117.59 (105.22 to 129.23)	-1.59 (-7.09 to 4.3)	8.64 (7.88 to 9.32)	6.42 (5.48 to 7.34)	-25.67 (-34.68 to -14.57)

**Virginia**	19.57 (16.3 to 22.9)	13.66 (11.73 to 15.57)	-30.21 (-35.29 to -24.71)	150.76 (134.31 to 170.08)	155.96 (140.74 to 171.87)	3.45 (-3.33 to 10.15)	12.85 (11.85 to 13.65)	10.46 (8.77 to 12.26)	-18.62 (-31.01 to -5.55)
**Washington**	15.74 (12.84 to 18.59)	11.35 (9.8 to 13.1)	-27.85 (-34.02 to -19.92)	129.9 (115.02 to 147.07)	132.15 (118.44 to 146.4)	1.73 (-4.25 to 8.16)	9.9 (9.02 to 10.56)	8.34 (6.9 to 9.69)	-15.74 (-28.47 to -2.16)
**West Virginia**	17.7 (14.72 to 20.61)	14.54 (12.48 to 16.55)	-17.84 (-23.77 to -11.69)	139.14 (123.1 to 155.98)	166.39 (149.23 to 184.79)	19.58 (11.9 to 28.1)	11.11 (10.26 to 11.88)	11.92 (10.25 to 13.78)	7.25 (-8.27 to 25.03)
**Wisconsin**	15.78 (12.9 to 18.67)	11.16 (9.62 to 12.8)	-29.25 (-35.64 to -21.38)	115.61 (102.21 to 129.03)	119.35 (106.8 to 132.02)	3.23 (-2.7 to 9.67)	9.96 (9.08 to 10.68)	8.47 (6.99 to 9.92)	-14.91 (-27.66 to -0.13)
**Wyoming**	16.36 (13.66 to 19.15)	11.36 (9.67 to 13.04)	-30.58 (-35.35 to -24.72)	124.56 (109.7 to 139.59)	128.53 (115.2 to 141.73)	3.19 (-2.89 to 9.28)	9.24 (8.4 to 9.92)	8.1 (6.95 to 9.18)	-12.28 (-23.62 to -0.66)

2.6 Table S3b: Age-standardized rate and percentage change of DALYs, YLDs, and YLLs for intracerebral hemorrhage over time, both sexes, by regions in US, 1990-2021

Intracerebral hemorrhage	DALYs			YLDs			YLLs		
**Location**	**1990**	**2021**	**change, %**	**1990**	**2021**	**change, %**	**1990**	**2021**	**change, %**
**Alabama**	403.97 (379.84 to 426.75)	372.55 (315.39 to 428.16)	-7.78 (-21.19 to 7.28)	26.28 (18.6 to 34.58)	30.81 (21.63 to 40.17)	17.23 (6.39 to 30.73)	377.69 (355.74 to 399.94)	341.74 (286.59 to 400.32)	-9.52 (-23.96 to 6.44)
**Alaska**	250.72 (235.03 to 266.14)	212.07 (187.41 to 240.18)	-15.42 (-24.99 to -3.85)	17.69 (12.41 to 23.3)	18.83 (13.43 to 24.73)	6.48 (-4.29 to 18.43)	233.03 (217.13 to 249.84)	193.23 (168.49 to 221.78)	-17.08 (-27.69 to -4.47)
**Arizona**	220.43 (207.41 to 232.1)	189.61 (163 to 218.75)	-13.98 (-25.92 to -1.12)	17.6 (12.44 to 23.17)	17.87 (12.8 to 23.5)	1.56 (-8.16 to 13.63)	202.83 (190.18 to 213.77)	171.74 (145.7 to 200.17)	-15.33 (-28.14 to -1.57)
**Arkansas**	364.95 (344.83 to 387.33)	315.57 (272.32 to 364.39)	-13.53 (-25.69 to 0.07)	22.8 (16.08 to 30.58)	26.29 (18.99 to 34.46)	15.33 (3.89 to 29.25)	342.15 (322.88 to 362.77)	289.27 (245.69 to 339.18)	-15.45 (-28.91 to -1.13)
**California**	270.7 (255.85 to 287.02)	216.4 (185.71 to 246.42)	-20.06 (-31.04 to -9.62)	19.96 (13.9 to 26.3)	20.43 (14.36 to 26.46)	2.34 (-7.58 to 15.1)	250.73 (236.6 to 265.48)	195.97 (166.91 to 225.24)	-21.84 (-33.12 to -10.64)
**Colorado**	208.79 (194.89 to 221.56)	166.3 (138.12 to 192.44)	-20.35 (-31.88 to -7.38)	17.13 (12.02 to 22.86)	17.8 (12.63 to 23.19)	3.91 (-5.38 to 14.89)	191.66 (178.26 to 203.13)	148.49 (120.35 to 173.8)	-22.52 (-35.1 to -8.87)

**Connecticut**	224.91 (210.52 to 239.48)	159.21 (133.6 to 186.06)	-29.21 (-39.95 to -16.89)	18.69 (13.44 to 24.35)	17.9 (12.63 to 23.12)	-4.27 (-13.67 to 5.15)	206.22 (193.25 to 220.31)	141.31 (115.73 to 166.3)	-31.47 (-43.08 to -18.59)
**Delaware**	261.21 (244.01 to 278.33)	237.52 (211.43 to 268.37)	-9.07 (-19.56 to 2.81)	20.28 (13.85 to 26.59)	21.17 (15.2 to 27.59)	4.4 (-5.9 to 15.47)	240.93 (223.9 to 258.69)	216.35 (189.84 to 248.03)	-10.2 (-21.43 to 2.41)
**District of Columbia**	501.62 (472.56 to 531.46)	251.72 (211.42 to 296.49)	-49.82 (-57.86 to -41.37)	25.33 (17.48 to 33.45)	22.43 (15.83 to 29.03)	-11.48 (-20.96 to -1.19)	476.28 (447.04 to 505.21)	229.3 (189.64 to 270.72)	-51.86 (-60.07 to -42.99)
**Florida**	254.89 (238.64 to 269.39)	215.39 (183.01 to 245.34)	-15.5 (-28.34 to -2.75)	20.3 (14.02 to 27.12)	21.59 (15.32 to 27.7)	6.37 (-3.48 to 17.62)	234.59 (220.48 to 247.34)	193.8 (161.04 to 224.41)	-17.39 (-31.89 to -3.69)
**Georgia**	391.48 (369.08 to 411.69)	296.44 (253.97 to 343.32)	-24.28 (-35.33 to -12.41)	23.93 (16.4 to 31.46)	25.55 (18.43 to 33.73)	6.77 (-3.82 to 18.57)	367.54 (345.36 to 386.79)	270.89 (229.07 to 315.36)	-26.3 (-38.05 to -13.89)
**Hawaii**	327.56 (306.91 to 345.83)	237.22 (203 to 276.88)	-27.58 (-37.9 to -15.3)	22.5 (15.65 to 29.99)	22.23 (16.13 to 28.91)	-1.21 (-10.53 to 10.92)	305.06 (284.32 to 322.15)	214.99 (180.88 to 253.43)	-29.52 (-40.61 to -16.43)
**Idaho**	216.63 (201.19 to 230.99)	181.29 (155.41 to 206.76)	-16.32 (-28.16 to -3.6)	17.88 (12.62 to 23.58)	18.65 (13.17 to 24.44)	4.29 (-5.57 to 16.53)	198.75 (183.78 to 212.38)	162.64 (136.3 to 187.73)	-18.17 (-30.88 to -4.27)
**Illinois**	293.56 (277.6 to 310)	235.78 (202.63 to 270.72)	-19.68 (-30.79 to -7.8)	21.35 (15.16 to 28.22)	21.58 (15.25 to 27.86)	1.08 (-10.02 to 13.95)	272.21 (257.73 to 287.64)	214.2 (184.07 to 248.96)	-21.31 (-33.22 to -8.96)

**Indiana**	284.52 (268.35 to 300.69)	273.69 (237.87 to 310.91)	-3.81 (-15.63 to 10.81)	20.84 (14.55 to 27.15)	23.21 (16.83 to 29.89)	11.38 (-0.08 to 24.03)	263.68 (248.41 to 278.38)	250.48 (213.88 to 288.71)	-5.01 (-17.83 to 10.41)
**Iowa**	207.86 (195.82 to 222.19)	205.71 (176.1 to 235.06)	-1.04 (-15.89 to 15.92)	16.46 (11.59 to 21.69)	17.52 (12.38 to 22.57)	6.46 (-4.22 to 18.95)	191.4 (178.66 to 204.03)	188.19 (157.31 to 218.73)	-1.68 (-17.58 to 16.7)
**Kansas**	238.2 (222.25 to 252.47)	226.14 (192 to 262.08)	-5.06 (-19.2 to 11.05)	19.29 (13.52 to 25.45)	21.19 (15.54 to 27.3)	9.87 (-2.14 to 23.21)	218.91 (203.57 to 232.56)	204.95 (170.88 to 242.05)	-6.38 (-21.33 to 11.16)
**Kentucky**	299.04 (282.22 to 314.57)	308.98 (262.45 to 354.68)	3.32 (-11.2 to 20.49)	22.05 (15.47 to 28.98)	25.21 (18.23 to 32.6)	14.33 (2.99 to 25.73)	276.99 (262.04 to 291.16)	283.77 (238.34 to 331.01)	2.45 (-12.71 to 20.83)
**Louisiana**	375.54 (354.28 to 396.99)	339.62 (293.89 to 394.72)	-9.57 (-21.27 to 4.78)	23.94 (16.65 to 31.83)	27.13 (19.62 to 35.88)	13.32 (1.37 to 26.97)	351.6 (333.02 to 369.78)	312.49 (267.74 to 366.43)	-11.12 (-23.95 to 4.08)
**Maine**	224.87 (210.38 to 238.16)	188.5 (161.5 to 216.64)	-16.17 (-27.77 to - 3.48)	17.73 (12.66 to 23.58)	19.01 (13.77 to 24.67)	7.22 (-2.82 to 18.54)	207.14 (192.61 to 220.23)	169.5 (144.54 to 197.34)	-18.17 (-30.4 to - 4.59)
**Maryland**	292.37 (275.85 to 309.6)	236.15 (203.42 to 274.35)	-19.23 (-31.56 to - 5.27)	20.33 (14.21 to 26.56)	21.02 (14.91 to 27.51)	3.38 (-7.55 to 14.68)	272.04 (256 to 287.62)	215.13 (180.44 to 252.92)	-20.92 (-34.05 to - 6.22)
**Massachusetts**	223.22 (209.83 to 237.64)	151.93 (128.44 to 178.14)	-31.94 (-41.67 to - 20.71)	17.73 (12.7 to 23.43)	17.57 (12.75 to 23.03)	-0.88 (-10.03 to 9.06)	205.5 (192.61 to 218.75)	134.36 (111.42 to 158.44)	-34.61 (-45.28 to - 22.57)

**Michigan**	268.79 (253.74 to 284.06)	216.28 (187.61 to 248.5)	-19.54 (-30.27 to -6.81)	20.06 (13.99 to 26.25)	21.19 (15.23 to 27.78)	5.63 (-4.84 to 17.74)	248.74 (234.13 to 263.41)	195.09 (168.05 to 225.73)	-21.57 (-32.99 to -7.82)
**Minnesota**	220.27 (206.04 to 233.69)	191.09 (160.33 to 220.75)	-13.25 (-26.46 to 0.36)	17.95 (12.82 to 24)	19.07 (13.75 to 24.81)	6.24 (-4.47 to 17.08)	202.33 (188.09 to 214.72)	172.03 (144.21 to 200.37)	-14.98 (-29.01 to -0.39)
**Mississippi**	406.57 (384.45 to 430.35)	433.1 (370.84 to 505.57)	6.52 (-8.81 to 24.63)	25.64 (18.01 to 34.12)	30.16 (21.49 to 38.98)	17.63 (5.05 to 32.19)	380.93 (359.53 to 403.86)	402.94 (345.35 to 474.08)	5.78 (-10.55 to 25.2)
**Missouri**	280.69 (262.99 to 298.31)	263.64 (226.12 to 307.03)	-6.07 (-19.94 to 8.64)	20.74 (14.51 to 27.1)	22.68 (16.37 to 29.74)	9.37 (-1.65 to 21.97)	259.95 (242.68 to 275.59)	240.96 (205.71 to 284.66)	-7.31 (-22.3 to 8.96)
**Montana**	229.74 (215.21 to 244.48)	187.54 (160.35 to 216.52)	-18.37 (-29.15 to -4.85)	18.57 (13.03 to 24.35)	19.04 (13.49 to 24.81)	2.51 (-8.19 to 12.39)	211.17 (197.55 to 225.29)	168.51 (142.11 to 196.94)	-20.2 (-31.69 to -5.62)
**Nebraska**	234.85 (219.47 to 249.25)	209.56 (179.88 to 238.72)	-10.77 (-23.85 to 2.03)	18.48 (13.26 to 24.44)	19.26 (13.7 to 25.05)	4.2 (-5.84 to 15.59)	216.36 (201.77 to 229.88)	190.3 (160.41 to 219.56)	-12.05 (-26.43 to 1.73)
**Nevada**	310.86 (292.45 to 330.52)	238.92 (202.29 to 273.93)	-23.14 (-34.83 to -11.84)	24.12 (17.48 to 32.07)	25 (17.77 to 32.86)	3.62 (-6.44 to 15.28)	286.74 (267.62 to 304.03)	213.92 (177.76 to 249.62)	-25.39 (-37.73 to -13.06)
**New Hampshire**	231.64 (216 to 247.24)	167.97 (143.88 to 193.1)	-27.49 (-37.07 to -16.71)	19.22 (13.83 to 25.57)	19.24 (13.87 to 25.17)	0.07 (-9.75 to 11.66)	212.42 (197.05 to 226.66)	148.74 (126.97 to 174.06)	-29.98 (-39.86 to -18.21)

**New Jersey**	264.37 (247.24 to 281.53)	195.53 (165.32 to 227.74)	-26.04 (-37.61 to -13.41)	20.42 (14.58 to 26.56)	20.04 (14.61 to 26.44)	-1.87 (-11.53 to 8.67)	243.95 (229.46 to 258.8)	175.49 (147.57 to 210.62)	-28.06 (-40.55 to -14.14)
**New Mexico**	225.21 (210.77 to 239.65)	197.01 (165.82 to 226.56)	-12.52 (-25 to 1.46)	17.97 (12.6 to 23.28)	18.46 (13.19 to 24.5)	2.73 (-7.28 to 13.88)	207.24 (191.95 to 220.39)	178.55 (149.54 to 207.94)	-13.84 (-27.43 to 1.36)
**New York**	262.71 (247.71 to 276.9)	160.36 (138.41 to 186.11)	-38.96 (-47.27 to -29.18)	19.61 (13.91 to 25.49)	18.43 (13.25 to 24.19)	-6.04 (-14.93 to 4.28)	243.1 (229.13 to 256.4)	141.93 (119.04 to 166.53)	-41.61 (-50.77 to -31.2)
**North Carolina**	352.9 (332.75 to 373.36)	280.07 (240.97 to 321.16)	-20.64 (-32.4 to -9.38)	22.62 (16.02 to 30.44)	24.71 (17.57 to 32.16)	9.2 (-1.34 to 21.1)	330.28 (310.08 to 349.93)	255.36 (215.8 to 298.06)	-22.68 (-35.15 to -10.53)
**North Dakota**	227.78 (212.17 to 242.71)	197.29 (170.35 to 225.25)	-13.38 (-25.15 to -0.04)	17.02 (11.8 to 22.36)	19.03 (13.45 to 24.76)	11.83 (1.07 to 24.35)	210.76 (196.52 to 225.34)	178.26 (152.28 to 204.99)	-15.42 (-28.02 to -0.64)
**Ohio**	258.72 (243.3 to 271.73)	259.21 (224.33 to 295.69)	0.19 (-12.95 to 15.43)	20.78 (14.34 to 27.12)	23.11 (16.39 to 30.16)	11.19 (-0.01 to 22.62)	237.94 (222.12 to 250.27)	236.11 (201.45 to 272.59)	-0.77 (-15.24 to 15.29)
**Oklahoma**	286.64 (269.01 to 303.12)	268.27 (233.14 to 310.02)	-6.41 (-18.9 to 9.99)	20.53 (14.3 to 26.81)	23.17 (16.78 to 30.34)	12.85 (1.55 to 26.23)	266.1 (249.73 to 282.73)	245.1 (209.8 to 287.07)	-7.89 (-21.22 to 9.43)
**Oregon**	239.37 (225.12 to 255.61)	209.81 (178.8 to 245.32)	-12.35 (-25.55 to 2.35)	19.01 (13.36 to 24.99)	19.69 (14.05 to 25.52)	3.57 (-6.51 to 15.27)	220.36 (206 to 234.76)	190.12 (161.26 to 224.92)	-13.72 (-27.52 to 2.31)

**Pennsylvania**	266.15 (250.51 to 280.84)	212.97 (182.09 to 243.76)	-19.98 (-30.64 to -8.09)	20.89 (14.73 to 27.05)	21.74 (15.64 to 28.23)	4.09 (-5.21 to 15.58)	245.26 (230.72 to 258.27)	191.23 (162.08 to 221.87)	-22.03 (-33.36 to -9.32)
**Rhode Island**	232.61 (216.86 to 246.83)	164.53 (139.98 to 193.45)	-29.27 (-39.77 to -18.61)	18.55 (12.69 to 24.45)	18.53 (13.24 to 24.08)	-0.13 (-9.3 to 9.46)	214.06 (197.88 to 227.42)	146.01 (122.29 to 173.36)	-31.79 (-42.62 to -20.26)
**South Carolina**	444.61 (419.8 to 467.91)	323.99 (275.05 to 373.98)	-27.13 (-37.82 to -15.25)	25.28 (17.97 to 33.04)	26.73 (18.99 to 34.94)	5.75 (-4.53 to 17.38)	419.33 (396.21 to 441.52)	297.26 (249.07 to 344.82)	-29.11 (-40.56 to -16.25)
**South Dakota**	224.72 (209.37 to 238.45)	201.05 (176.27 to 227.91)	-10.53 (-21.25 to 1.12)	17.99 (12.63 to 23.47)	19.56 (14.13 to 25.36)	8.73 (-1.2 to 19.58)	206.73 (192.57 to 220.57)	181.49 (158.01 to 206.84)	-12.21 (-23.62 to 0.71)
**Tennessee**	341.06 (321.72 to 360.8)	308.13 (265.69 to 353.64)	-9.65 (-21.58 to 3.9)	23.12 (16.36 to 30.66)	25.8 (18.35 to 33.44)	11.62 (1.06 to 24.19)	317.94 (299.38 to 336.61)	282.33 (241.99 to 326.61)	-11.2 (-23.71 to 3.4)
**Texas**	298.45 (281.79 to 313.93)	261.75 (229.33 to 297.48)	-12.29 (-24.05 to 0.82)	22.21 (15.53 to 29.33)	24.11 (17.11 to 31.51)	8.58 (-1.39 to 20.32)	276.24 (260.04 to 291.13)	237.64 (205.49 to 273.93)	-13.97 (-26.41 to 0.26)
**Utah**	229.36 (215.38 to 244.22)	188.51 (165.57 to 215.97)	-17.81 (-28.21 to -4.6)	18.19 (12.99 to 23.97)	18.66 (13.4 to 24.28)	2.59 (-8.01 to 13.62)	211.17 (197.69 to 225.22)	169.85 (145.61 to 198.12)	-19.57 (-30.74 to -5.39)
**Vermont**	207.19 (192.85 to 221.11)	148.62 (131.25 to 168.33)	-28.27 (-36.36 to -18.46)	17.05 (11.87 to 22.48)	16.68 (11.85 to 22.02)	-2.15 (-10.54 to 8.41)	190.14 (176.83 to 203.46)	131.94 (114.96 to 150.64)	-30.61 (-39.59 to -19.86)

**Virginia**	312.89 (295.37 to 329.67)	243.1 (206.88 to 281.91)	-22.3 (-34.44 to -10.4)	21.29 (15.03 to 27.88)	21.93 (15.35 to 28.3)	2.99 (-7.79 to 14.92)	291.6 (273.52 to 306.36)	221.17 (185.06 to 260.3)	-24.15 (-36.9 to -11.24)
**Washington**	232.39 (216.74 to 246.73)	184.47 (156.7 to 210.74)	-20.62 (-31.98 to -8.57)	18.52 (12.89 to 24.54)	18.87 (13.24 to 24.75)	1.9 (-7.69 to 14.25)	213.88 (199.55 to 226.33)	165.6 (138.32 to 191.91)	-22.57 (-34.78 to -9.28)
**West Virginia**	273.76 (257.71 to 291.27)	298.49 (257.41 to 341.27)	9.04 (-7.01 to 26.62)	19.79 (13.45 to 25.85)	23.04 (16.72 to 30.07)	16.44 (4.12 to 30.93)	253.96 (239.29 to 270.06)	275.45 (233.81 to 318.37)	8.46 (-8.58 to 27.58)
**Wisconsin**	236.94 (221.5 to 252.69)	187.58 (157.93 to 216.93)	-20.83 (-32.36 to -7.19)	16.54 (11.48 to 21.86)	17.04 (12.04 to 22.4)	3.01 (-7.6 to 15.2)	220.4 (206.12 to 234.49)	170.54 (141.34 to 201.41)	-22.62 (-35.22 to -8.09)
**Wyoming**	225.48 (209.93 to 242.01)	191.8 (168.46 to 216.43)	-14.94 (-25.53 to -3.6)	17.87 (12.18 to 23.36)	18.26 (12.96 to 23.98)	2.23 (-7.64 to 12.67)	207.62 (193.04 to 221.98)	173.54 (152.11 to 195.94)	-16.41 (-27.83 to -4.24)

2.7 Table S4a: Age-standardized rate and percentage change of incidence, prevalence, and mortality for subarachnoid hemorrhage over time, both sexes, by regions in US, 1990-2021

Subarachnoid hemorrhage	Incidence			Prevalence			Mortality		
**Location**	**1990**	**2021**	**change, %**	**1990**	**2021**	**change, %**	**1990**	**2021**	**change, %**
**Alabama**	7.59 (6.58 to 8.8)	8.03 (7.02 to 9.27)	5.83 (0.03 to 13.08)	108.57 (96.09 to 122.63)	127.1 (113.73 to 142.72)	17.06 (10.18 to 25.42)	5.14 (4.83 to 5.46)	5.3 (4.52 to 6.13)	2.98 (-12.81 to 20.83)
**Alaska**	6.99 (6.04 to 8.18)	6.67 (5.84 to 7.73)	-4.58 (-10.58 to 1.5)	82.22 (72.28 to 92.99)	88.08 (78.94 to 98.07)	7.13 (1.06 to 13.84)	4.12 (3.82 to 4.42)	3.47 (3.05 to 3.93)	-15.74 (-25.71 to -3.68)
**Arizona**	6.16 (5.23 to 7.31)	5.57 (4.84 to 6.51)	-9.56 (-14.93 to -3.32)	81.48 (71.84 to 92.06)	86.21 (77.41 to 95.77)	5.79 (0.76 to 12.26)	2.96 (2.75 to 3.16)	2.71 (2.31 to 3.17)	-8.54 (-22.64 to 7.11)
**Arkansas**	7.04 (6.03 to 8.38)	7.32 (6.34 to 8.44)	3.97 (-3.21 to 12.4)	94.72 (83.41 to 107.29)	110.18 (98.78 to 123.38)	16.33 (8.5 to 24.75)	4.96 (4.62 to 5.25)	4.6 (3.94 to 5.3)	-7.16 (-21.56 to 8.66)
**California**	6.78 (5.74 to 8.07)	5.88 (5.12 to 6.77)	-13.31 (-19.15 to -7.04)	87 (77.9 to 98.79)	87.9 (79.62 to 97.22)	1.03 (-4.51 to 6.53)	3.71 (3.46 to 3.98)	2.8 (2.38 to 3.21)	-24.64 (-36.06 to -13.56)
**Colorado**	6.18 (5.27 to 7.28)	5.63 (4.87 to 6.48)	-8.94 (-14.85 to -2.03)	82 (72.44 to 93.13)	84.55 (76.59 to 93.57)	3.11 (-2.44 to 8.8)	3.08 (2.85 to 3.29)	2.72 (2.25 to 3.19)	-11.57 (-25.65 to 3.14)

**Connecticut**	6.87 (5.93 to 8.05)	5.77 (5.08 to 6.61)	-16.08 (-20.75 to -11.05)	86.32 (76.59 to 97.34)	84.97 (77.06 to 93.76)	-1.56 (-7 to 4.48)	2.98 (2.76 to 3.18)	2.17 (1.77 to 2.57)	-27.25 (-39.31 to -14.2)
**Delaware**	7.06 (6.08 to 8.25)	6.32 (5.53 to 7.26)	-10.52 (-16.03 to -4.07)	89.9 (79.84 to 100.93)	98.58 (89.36 to 110.33)	9.65 (3.44 to 16.87)	3.92 (3.63 to 4.19)	3.25 (2.84 to 3.71)	-17.02 (-27.03 to -5.22)
**District of Columbia**	7.74 (6.69 to 9.12)	6.05 (5.35 to 6.91)	-21.92 (-26.84 to -16.36)	97.36 (85.29 to 110.92)	99 (87.98 to 111.78)	1.69 (-4.04 to 10.69)	6.24 (5.8 to 6.65)	3.14 (2.62 to 3.73)	-49.58 (-57.84 to -39.78)
**Florida**	6.51 (5.57 to 7.65)	5.88 (5.09 to 6.81)	-9.72 (-14.81 to -2.91)	90.4 (79.72 to 102.53)	97.08 (87.52 to 107.91)	7.39 (0.59 to 14.22)	3.43 (3.2 to 3.62)	2.88 (2.42 to 3.35)	-16.06 (-28.76 to -2.13)
**Georgia**	7.76 (6.65 to 9.1)	7.46 (6.49 to 8.63)	-3.87 (-9.57 to 2.85)	100.93 (89.83 to 113.99)	111.84 (100.1 to 124.97)	10.81 (4.49 to 18.52)	4.99 (4.7 to 5.27)	4.31 (3.59 to 4.99)	-13.61 (-26.15 to -0.29)
**Hawaii**	7.61 (6.55 to 8.99)	6.79 (5.93 to 7.85)	-10.78 (-15.89 to -4.4)	96.12 (85.22 to 108.68)	99.76 (89.69 to 110.74)	3.79 (-1.84 to 10.44)	4.21 (3.87 to 4.49)	3.23 (2.7 to 3.75)	-23.32 (-34.66 to -11.34)
**Idaho**	6.71 (5.76 to 7.75)	6.25 (5.44 to 7.25)	-6.76 (-11.88 to -1.24)	85.86 (75.99 to 97)	89.26 (80.43 to 99.37)	3.96 (-1.37 to 10.09)	3.11 (2.86 to 3.33)	2.85 (2.42 to 3.24)	-8.39 (-21.67 to 4.27)
**Illinois**	7.22 (6.16 to 8.46)	6.35 (5.5 to 7.27)	-12.08 (-16.81 to -5.79)	90.57 (80.4 to 102.6)	94.64 (85.6 to 104.88)	4.5 (-1.38 to 10.9)	3.75 (3.49 to 3.99)	3.28 (2.77 to 3.82)	-12.52 (-24.83 to 2.57)

**Indiana**	7.06 (6.06 to 8.26)	6.9 (6.06 to 7.93)	-2.19 (-7.45 to 4.71)	92.76 (81.91 to 104.2)	103.25 (92.82 to 115.05)	11.31 (4.52 to 17.7)	3.89 (3.63 to 4.14)	4.01 (3.42 to 4.64)	3.08 (-11.7 to 20.45)
**Iowa**	6.06 (5.16 to 7.16)	5.71 (4.97 to 6.58)	-5.81 (-12.01 to 1.1)	79.01 (69.8 to 89.2)	85.3 (76.64 to 94.52)	7.97 (2.02 to 13.89)	2.8 (2.59 to 3.02)	2.79 (2.31 to 3.23)	-0.23 (-15.16 to 17.02)
**Kansas**	6.76 (5.81 to 7.92)	6.57 (5.72 to 7.56)	-2.88 (-8.54 to 3.7)	88.54 (78.62 to 100.77)	99.65 (90.21 to 109.19)	12.54 (6.22 to 18.79)	3.33 (3.08 to 3.59)	3.47 (2.94 to 4.06)	4.12 (-11.58 to 21.83)
**Kentucky**	6.98 (6.04 to 8.1)	7 (6.15 to 7.95)	0.24 (-5.08 to 6.78)	96.62 (85.77 to 109.09)	114.42 (102.41 to 129.22)	18.43 (10.27 to 28.37)	4.01 (3.76 to 4.27)	4.55 (3.83 to 5.33)	13.43 (-4.02 to 32.47)
**Louisiana**	7.5 (6.43 to 8.86)	7.23 (6.33 to 8.31)	-3.56 (-9.85 to 3.46)	101.34 (89.77 to 114.55)	115.05 (102.33 to 129.74)	13.52 (6.63 to 22.08)	5 (4.69 to 5.31)	4.68 (4.03 to 5.47)	-6.4 (-19.39 to 9.49)
**Maine**	6.47 (5.53 to 7.6)	6.04 (5.25 to 6.89)	-6.6 (-11.58 to - 0.91)	82.04 (72.79 to 92.63)	89.83 (81.21 to 99.12)	9.49 (3.23 to 15.03)	2.88 (2.65 to 3.09)	2.62 (2.23 to 3.05)	-9.04 (-22.71 to 7.07)
**Maryland**	6.59 (5.57 to 7.75)	5.75 (4.98 to 6.59)	-12.78 (-18.44 to - 5.77)	83.4 (74 to 94.7)	83.64 (75.67 to 92.32)	0.29 (-5.75 to 6.15)	3.6 (3.35 to 3.8)	2.98 (2.5 to 3.48)	-17.25 (-31.12 to - 1.81)
**Massachusetts**	6.52 (5.54 to 7.69)	5.6 (4.84 to 6.46)	-13.99 (-18.93 to - 7.83)	93.76 (83.1 to 105.28)	92.91 (84.3 to 102.95)	-0.91 (-6.17 to 4.93)	3.12 (2.92 to 3.32)	2.28 (1.89 to 2.67)	-27.05 (-38.84 to - 13.95)

**Michigan**	6.76 (5.75 to 7.98)	6.09 (5.28 to 7.05)	-9.97 (-15.8 to -3.32)	94.14 (83.04 to 106.35)	103.78 (93.27 to 115.4)	10.23 (4.35 to 17.73)	3.81 (3.56 to 4.06)	3 (2.56 to 3.47)	-21.17 (-32.85 to -8.54)
**Minnesota**	7.01 (6.03 to 8.14)	6.2 (5.43 to 7.07)	-11.49 (-16.83 to -5.59)	86.85 (77.2 to 97.76)	85.26 (77.17 to 93.67)	-1.83 (-7.57 to 4.07)	3.17 (2.91 to 3.36)	2.83 (2.36 to 3.27)	-10.87 (-24.55 to 3.86)
**Mississippi**	7.68 (6.59 to 9.06)	7.85 (6.86 to 8.97)	2.15 (-4.72 to 9.77)	107.24 (94.74 to 120.88)	124.44 (111.19 to 138.29)	16.04 (9.19 to 24.73)	5.04 (4.73 to 5.36)	5.31 (4.5 to 6.16)	5.29 (-10.28 to 25.07)
**Missouri**	6.99 (6.01 to 8.08)	6.74 (5.88 to 7.72)	-3.57 (-9.46 to 2.8)	89.56 (79.6 to 101.46)	103.52 (92.77 to 115.79)	15.59 (8.7 to 24.01)	3.85 (3.57 to 4.1)	3.86 (3.29 to 4.45)	0.19 (-16.84 to 16.67)
**Montana**	6.76 (5.84 to 7.93)	6.33 (5.47 to 7.25)	-6.28 (-12.72 to -0.08)	87.96 (77.93 to 99.68)	96.09 (86.53 to 106.77)	9.24 (3.63 to 15.16)	3.41 (3.15 to 3.65)	2.93 (2.5 to 3.37)	-14.21 (-26.18 to -0.14)
**Nebraska**	6.88 (5.87 to 8.04)	6.4 (5.62 to 7.36)	-6.96 (-12.37 to -0.78)	86.65 (76.5 to 97.21)	92.01 (83.25 to 101.19)	6.19 (0.09 to 11.92)	3.32 (3.07 to 3.56)	3.06 (2.6 to 3.52)	-7.95 (-20.88 to 6.22)
**Nevada**	9.07 (7.81 to 10.63)	7.51 (6.51 to 8.61)	-17.22 (-21.65 to -11.94)	110.82 (98.33 to 124.99)	109.51 (98.76 to 122.16)	-1.18 (-6.33 to 4.7)	4.03 (3.74 to 4.31)	3.29 (2.78 to 3.78)	-18.32 (-29.61 to -4.63)
**New Hampshire**	6.57 (5.65 to 7.69)	5.71 (5.02 to 6.53)	-13.02 (-17.97 to -7.56)	90.77 (80.69 to 101.68)	89.07 (80.91 to 99.03)	-1.87 (-7.16 to 3.95)	2.96 (2.75 to 3.19)	2.33 (1.97 to 2.67)	-21.34 (-32.85 to -8.19)

**New Jersey**	6.74 (5.74 to 8)	5.71 (4.96 to 6.63)	-15.26 (-20.65 to -8)	89.93 (79.64 to 102.52)	84.83 (76.87 to 93.25)	-5.67 (-10.83 to -0.67)	3.67 (3.46 to 3.89)	2.9 (2.41 to 3.39)	-21.18 (-33.95 to -8.25)
**New Mexico**	6.64 (5.67 to 7.76)	5.97 (5.2 to 6.92)	-10.06 (-15.72 to -3.84)	83.14 (73.47 to 94.11)	91.78 (82.81 to 101.57)	10.4 (3.73 to 17.58)	3.13 (2.88 to 3.38)	2.79 (2.36 to 3.25)	-10.97 (-24.79 to 4.72)
**New York**	6.79 (5.75 to 8.01)	5.46 (4.71 to 6.29)	-19.62 (-24.71 to -14.07)	93.79 (82.82 to 106.03)	92.31 (83.29 to 102.17)	-1.58 (-7.78 to 4.75)	3.53 (3.31 to 3.75)	2.31 (1.94 to 2.67)	-34.69 (-44.75 to -23.7)
**North Carolina**	7.08 (6.07 to 8.34)	6.52 (5.65 to 7.53)	-7.98 (-13.49 to -1.05)	95.41 (84.9 to 107.64)	100.98 (90.76 to 112.1)	5.84 (-0.46 to 13.05)	4.51 (4.2 to 4.82)	3.74 (3.19 to 4.38)	-16.98 (-30.03 to -2.8)
**North Dakota**	6.63 (5.7 to 7.78)	6.44 (5.65 to 7.35)	-2.86 (-8.35 to 3.32)	78.82 (69.7 to 89.38)	86.78 (78.39 to 95.91)	10.09 (4.57 to 16.81)	3.14 (2.9 to 3.37)	2.77 (2.38 to 3.23)	-11.62 (-23.87 to 2.93)
**Ohio**	7.04 (6.06 to 8.18)	6.75 (5.87 to 7.73)	-4.05 (-9.69 to 2.16)	95.92 (85.47 to 108.83)	106.16 (95.36 to 118.01)	10.67 (4.6 to 17.53)	3.75 (3.51 to 3.98)	3.69 (3.12 to 4.27)	-1.66 (-15.49 to 14.06)
**Oklahoma**	7.23 (6.24 to 8.49)	7.18 (6.28 to 8.26)	-0.68 (-6.22 to 5.99)	93.98 (83.03 to 106.45)	108.73 (97.76 to 120.64)	15.7 (8.94 to 23.58)	4.2 (3.94 to 4.44)	4.17 (3.6 to 4.89)	-0.63 (-14.14 to 16.04)
**Oregon**	7.01 (6.03 to 8.23)	6.38 (5.58 to 7.37)	-9 (-14.6 to -2.45)	87.81 (77.68 to 99.43)	95.53 (86.05 to 105.37)	8.78 (2.75 to 15.56)	3.41 (3.18 to 3.64)	2.97 (2.52 to 3.56)	-13.02 (-25.71 to 2.12)

**Pennsylvania**	7.02 (6 to 8.18)	6.33 (5.56 to 7.26)	-9.85 (-15.39 to -3.99)	94.06 (83.35 to 106.81)	100.77 (90.6 to 111.58)	7.14 (0.12 to 13.69)	3.59 (3.33 to 3.82)	3.14 (2.64 to 3.67)	-12.38 (-25.23 to 2.78)
**Rhode Island**	6.67 (5.68 to 7.77)	5.75 (5.08 to 6.61)	-13.79 (-19.39 to -7.29)	87.91 (78.37 to 98.4)	90.13 (81.15 to 100)	2.52 (-2.65 to 8.71)	2.98 (2.73 to 3.19)	2.15 (1.79 to 2.5)	-27.98 (-38.32 to -16.4)
**South Carolina**	7.84 (6.77 to 9.22)	7.38 (6.45 to 8.37)	-5.96 (-12.18 to 1.17)	100.96 (88.87 to 113.94)	112.45 (100.87 to 124.58)	11.39 (4.42 to 19.43)	5.51 (5.21 to 5.86)	4.66 (3.96 to 5.35)	-15.28 (-28.19 to -2.07)
**South Dakota**	6.66 (5.75 to 7.78)	6.4 (5.58 to 7.33)	-3.93 (-9.96 to 2.04)	85.5 (76.3 to 96.15)	91.83 (82.85 to 101.76)	7.41 (2.01 to 13.07)	3.18 (2.94 to 3.42)	2.86 (2.49 to 3.26)	-10.23 (-21.8 to 3.69)
**Tennessee**	7.31 (6.31 to 8.56)	7.09 (6.18 to 8.14)	-3 (-8.74 to 4.52)	99.75 (87.71 to 112.65)	111.15 (99.87 to 123.59)	11.43 (5.52 to 18.63)	4.54 (4.26 to 4.81)	4.55 (3.91 to 5.26)	0.3 (-13.07 to 15.57)
**Texas**	7.18 (6.14 to 8.37)	6.76 (5.92 to 7.74)	-5.81 (-11.57 to 0.59)	99.67 (88.16 to 112.99)	104.24 (94.47 to 115.29)	4.59 (-1.71 to 11.22)	3.94 (3.68 to 4.18)	3.64 (3.14 to 4.16)	-7.75 (-21.54 to 6.74)
**Utah**	6.78 (5.78 to 7.95)	6.19 (5.39 to 7.11)	-8.71 (-13.88 to -2.11)	89.88 (79.23 to 101.79)	92.37 (83.25 to 101.66)	2.78 (-2.16 to 8.9)	3.16 (2.92 to 3.39)	2.97 (2.58 to 3.41)	-5.79 (-18.21 to 8.43)
**Vermont**	6.66 (5.73 to 7.77)	5.7 (4.95 to 6.52)	-14.35 (-19.21 to -8.84)	84.93 (75.6 to 94.82)	86.55 (78.83 to 95.82)	1.91 (-3.88 to 6.85)	3.04 (2.8 to 3.26)	2.24 (1.95 to 2.55)	-26.34 (-35.74 to -15.82)

**Virginia**	7.14 (6.15 to 8.37)	6.53 (5.7 to 7.51)	-8.54 (-13.53 to -2.81)	89.89 (79.67 to 101.92)	100.54 (90.88 to 112.11)	11.85 (5.42 to 19.57)	3.82 (3.58 to 4.03)	3.28 (2.78 to 3.84)	-14.07 (-27.54 to 1.18)
**Washington**	6.52 (5.5 to 7.73)	5.65 (4.89 to 6.56)	-13.34 (-18.4 to -7.74)	89.89 (79.33 to 101.27)	91.07 (82.63 to 101.48)	1.31 (-4.69 to 7.52)	3.37 (3.13 to 3.61)	2.61 (2.18 to 3.04)	-22.6 (-33.8 to -9.65)
**West Virginia**	6.84 (5.89 to 7.95)	7.02 (6.08 to 8.1)	2.54 (-3.69 to 9.32)	89.76 (78.97 to 102.28)	107.41 (95.86 to 120.13)	19.66 (12.68 to 28.06)	3.88 (3.62 to 4.15)	4.18 (3.59 to 4.93)	7.82 (-8.92 to 27.46)
**Wisconsin**	6.33 (5.39 to 7.45)	5.76 (4.96 to 6.59)	-9.05 (-14.36 to -1.62)	77.12 (67.92 to 87.35)	80.36 (72.09 to 88.64)	4.2 (-1.57 to 10.26)	3.26 (3.03 to 3.47)	2.69 (2.25 to 3.15)	-17.53 (-31.08 to -3.89)
**Wyoming**	6.67 (5.74 to 7.77)	6.16 (5.36 to 7.06)	-7.72 (-13.37 to -1.48)	85.41 (75.63 to 96.29)	88.54 (79.98 to 98.03)	3.66 (-1.72 to 9.18)	3.46 (3.18 to 3.72)	3.07 (2.65 to 3.47)	-11.37 (-22.13 to 0.04)

2.8 Table S4b: Age-standardized rate and percentage change of DALYs, YLDs, and YLLs for subarachnoid hemorrhage over time, both sexes, by regions in US, 1990-2021

Subarachnoid hemorrhage	DALYs			YLDs			YLLs		
**Location**	**1990**	**2021**	**change, %**	**1990**	**2021**	**change, %**	**1990**	**2021**	**change, %**
**Alabama**	181.04 (170.75 to 192.16)	164.75 (140.59 to 188.86)	-9 (-22.28 to 5.8)	15.36 (10.56 to 20.29)	17.58 (12.2 to 22.83)	14.4 (1.47 to 29.54)	165.67 (156.68 to 174.9)	147.17 (125 to 170.2)	-11.17 (-25.72 to 4.83)
**Alaska**	136.87 (127.74 to 145.76)	114 (101.15 to 128.3)	-16.71 (-26.39 to -5.64)	11.76 (8.33 to 15.43)	12.4 (8.68 to 16.12)	5.49 (-6.58 to 20.37)	125.11 (116.84 to 133.8)	101.6 (89.03 to 115.01)	-18.79 (-29.04 to -6.84)
**Arizona**	102.36 (96.44 to 109.2)	88.33 (76.08 to 101.55)	-13.7 (-25.3 to 0.11)	11.71 (8.11 to 15.3)	12.17 (8.44 to 15.97)	3.92 (-8.05 to 18.31)	90.65 (85.39 to 96.49)	76.16 (64.69 to 89)	-15.98 (-29.03 to -0.41)
**Arkansas**	173.27 (163.17 to 183.68)	148.68 (128.3 to 169.8)	-14.19 (-26.66 to -1.24)	13.46 (9.59 to 17.76)	15.38 (10.88 to 19.88)	14.24 (2.31 to 29.11)	159.81 (150.68 to 168.49)	133.31 (114.03 to 154.08)	-16.58 (-29.88 to -2.41)
**California**	128.26 (120.84 to 136.73)	86.54 (74.19 to 97.62)	-32.53 (-41.76 to -24.02)	12.35 (8.59 to 16.24)	12.36 (8.59 to 15.97)	0.11 (-11.74 to 12.56)	115.92 (109.26 to 123.46)	74.18 (63.49 to 84.99)	-36.01 (-45.84 to -26.11)
**Colorado**	102.26 (95.3 to 109.21)	80.6 (69.38 to 92.32)	-21.18 (-31.77 to -9.22)	11.81 (8.33 to 15.45)	11.97 (8.31 to 15.47)	1.36 (-9.57 to 13.87)	90.44 (84.43 to 96.43)	68.63 (57.39 to 80.12)	-24.12 (-35.86 to -10.52)

**Connecticut**	103.86 (97.86 to 110.22)	67.03 (56.82 to 78.26)	-35.46 (-44.78 to -24.66)	12.43 (8.55 to 16.42)	12.07 (8.53 to 15.64)	-2.92 (-13.77 to 8.5)	91.43 (86.53 to 97.28)	54.97 (44.77 to 64.95)	-39.88 (-50.85 to -28.3)
**Delaware**	130.75 (122.67 to 138.95)	101 (90.55 to 113.87)	-22.75 (-31.2 to -12.47)	12.83 (8.95 to 16.62)	13.8 (9.72 to 17.68)	7.57 (-4.73 to 21.21)	117.92 (110.6 to 125.54)	87.21 (76.92 to 99.43)	-26.05 (-35.63 to -15.13)
**District of Columbia**	224.63 (210.81 to 239.96)	101.09 (85.9 to 118.85)	-55 (-61.77 to -47.03)	13.9 (9.73 to 18.17)	13.94 (9.96 to 18.18)	0.31 (-12.49 to 15.69)	210.73 (197.48 to 225.35)	87.15 (72.5 to 104.35)	-58.64 (-65.6 to -50.33)
**Florida**	126.67 (119.57 to 133.59)	96.05 (82.31 to 110.04)	-24.17 (-34.31 to -13.07)	12.86 (8.86 to 16.77)	13.54 (9.47 to 17.73)	5.25 (-7.27 to 20.04)	113.81 (107.33 to 119.56)	82.52 (69.66 to 95.87)	-27.5 (-39.45 to -15.18)
**Georgia**	172.62 (163.57 to 181.94)	130.12 (110.82 to 149.2)	-24.62 (-34.58 to -13.59)	14.35 (9.96 to 18.87)	15.62 (11.06 to 19.87)	8.89 (-3.71 to 24)	158.27 (149.93 to 166.97)	114.5 (96.33 to 133.59)	-27.66 (-38.41 to -15.8)
**Hawaii**	141.02 (131.9 to 149.99)	102.83 (89.57 to 116.43)	-27.08 (-36.51 to -16.91)	13.68 (9.47 to 18.12)	14.01 (9.83 to 18.3)	2.4 (-8.99 to 17.38)	127.33 (118.91 to 135.24)	88.82 (76.14 to 102.86)	-30.25 (-40.87 to -19.18)
**Idaho**	104.2 (97.09 to 112)	84.79 (74.14 to 95.8)	-18.62 (-29.27 to -8.08)	12.27 (8.51 to 16.34)	12.61 (8.78 to 16.23)	2.76 (-8.53 to 16.19)	91.92 (86.29 to 98.08)	72.18 (61.63 to 83.06)	-21.48 (-33.08 to -9.52)
**Illinois**	132.82 (125.33 to 140.19)	97.17 (84.03 to 111.21)	-26.84 (-36.2 to -16.03)	12.88 (9.1 to 16.83)	13.34 (9.36 to 17.51)	3.61 (-8.51 to 17.42)	119.94 (112.89 to 126.5)	83.82 (71.79 to 97.74)	-30.11 (-40.99 to -17.7)

**Indiana**	133.9 (125.76 to 142.49)	119.14 (103.92 to 137.05)	-11.02 (-22.73 to 2.46)	13.18 (9.34 to 17.32)	14.34 (10.17 to 18.62)	8.82 (-3.1 to 22.56)	120.72 (113.42 to 128.21)	104.8 (89.72 to 121.61)	-13.19 (-25.84 to 1.75)
**Iowa**	94.37 (88.65 to 100.36)	86.44 (74.18 to 99.3)	-8.4 (-21.17 to 5.31)	11.37 (8.04 to 14.99)	12.07 (8.48 to 15.49)	6.11 (-6.09 to 20.41)	83 (77.91 to 88.29)	74.37 (61.66 to 86.45)	-10.39 (-24.14 to 5.02)
**Kansas**	112.92 (105.08 to 120.56)	105.67 (90.51 to 120.64)	-6.42 (-19.78 to 8.65)	12.69 (8.75 to 16.39)	13.99 (9.74 to 17.95)	10.2 (-1.58 to 24.67)	100.23 (94.15 to 107.57)	91.68 (76.61 to 108.2)	-8.53 (-23.04 to 8.36)
**Kentucky**	136.58 (128.95 to 145.15)	139.31 (118.62 to 161.89)	2 (-12.35 to 18.27)	13.75 (9.72 to 18.2)	15.82 (11.01 to 20.43)	15.06 (0.82 to 31.1)	122.83 (116.34 to 130.41)	123.49 (103.05 to 145.88)	0.54 (-14.98 to 19.03)
**Louisiana**	176.59 (165.76 to 187.45)	145.94 (125.75 to 169.27)	-17.36 (-28.97 to -3.73)	14.34 (9.82 to 18.74)	15.97 (11.42 to 20.62)	11.38 (-1.82 to 25.96)	162.25 (152.1 to 171.95)	129.97 (111.51 to 153.18)	-19.9 (-32.25 to -5.07)
**Maine**	96.14 (89.21 to 102.83)	84.74 (73.77 to 96.71)	-11.86 (-24.14 to 2.28)	11.76 (8.33 to 15.31)	12.61 (8.83 to 16.18)	7.22 (-4.39 to 20.59)	84.38 (78.71 to 89.92)	72.13 (61.45 to 84.16)	-14.52 (-28.03 to 0.88)
**Maryland**	120.06 (113.14 to 127)	91.93 (79.46 to 106.13)	-23.43 (-34.93 to -10.81)	11.93 (8.56 to 15.69)	11.91 (8.33 to 15.34)	-0.19 (-12.36 to 12.6)	108.13 (102.24 to 114.07)	80.03 (67.32 to 94.54)	-25.99 (-38.97 to -11.93)
**Massachusetts**	107.58 (100.72 to 114.26)	66.6 (56.77 to 77.46)	-38.09 (-46.75 to -28.51)	13.33 (9.32 to 17.58)	13.07 (9.31 to 16.97)	-1.92 (-12.91 to 11.78)	94.25 (89.23 to 99.61)	53.53 (44.68 to 63.46)	-43.2 (-52.75 to -32.8)

**Michigan**	134.89 (126.66 to 143.63)	95.5 (82.54 to 108.67)	-29.2 (-38.94 to -19.08)	13.37 (9.42 to 17.69)	14.52 (10.18 to 18.79)	8.54 (-4.22 to 22.1)	121.52 (114.58 to 128.96)	80.98 (68.96 to 93.84)	-33.35 (-43.56 to -21.88)
**Minnesota**	103.54 (96.83 to 109.75)	82.23 (70.05 to 94.11)	-20.58 (-31.55 to -8.48)	12.45 (8.92 to 16.42)	12.11 (8.43 to 15.56)	-2.72 (-13.62 to 9.37)	91.09 (85.44 to 96.32)	70.12 (58.97 to 82)	-23.02 (-35.4 to -9.86)
**Mississippi**	179.54 (168.29 to 190.27)	173.92 (149.21 to 201.67)	-3.13 (-17.24 to 13.06)	15.2 (10.8 to 20.18)	17.26 (12.11 to 22.75)	13.57 (-0.33 to 29.02)	164.34 (155.22 to 174.56)	156.66 (132.63 to 182.64)	-4.67 (-19.57 to 12.92)
**Missouri**	133.9 (125.49 to 141.97)	116.85 (100.23 to 134.07)	-12.73 (-25.73 to 0.85)	12.76 (8.96 to 16.61)	14.5 (10.41 to 18.89)	13.67 (0.67 to 28.43)	121.14 (113.62 to 128.26)	102.35 (86.65 to 119.03)	-15.51 (-29.67 to -0.07)
**Montana**	114.5 (107.34 to 121.99)	94.45 (83.02 to 106.91)	-17.51 (-27.6 to -5.38)	12.62 (8.91 to 16.54)	13.48 (9.66 to 17.41)	6.84 (-4.61 to 20.6)	101.89 (95.26 to 108.82)	80.97 (69.87 to 93.29)	-20.53 (-31.74 to -7.14)
**Nebraska**	113.09 (105.53 to 120.84)	92.59 (80.98 to 104.58)	-18.13 (-28.43 to -6.88)	12.4 (8.77 to 16.21)	12.98 (9.4 to 16.68)	4.71 (-7.92 to 18.47)	100.69 (94.16 to 108.2)	79.6 (68.53 to 91.27)	-20.94 (-32.33 to -8.47)
**Nevada**	138.39 (129.58 to 147.66)	104.54 (90.43 to 118.84)	-24.46 (-34.67 to -13.38)	15.57 (10.99 to 20.18)	15.25 (10.54 to 19.88)	-2.06 (-13.08 to 11.55)	122.82 (115.25 to 130.68)	89.29 (75.65 to 102.33)	-27.3 (-38.36 to -15.27)
**New Hampshire**	97.78 (91.44 to 105.32)	71.21 (61.92 to 80.31)	-27.17 (-36.49 to -16.75)	12.89 (9.01 to 16.77)	12.49 (8.84 to 16.08)	-3.12 (-13.65 to 7.4)	84.89 (79.6 to 91.13)	58.72 (49.95 to 67.47)	-30.82 (-41.12 to -18.97)

**New Jersey**	132.03 (124.45 to 139.15)	83.55 (72.28 to 96.11)	-36.72 (-45.56 to -27.04)	12.85 (8.85 to 16.66)	12.07 (8.44 to 15.82)	-6.02 (-15.16 to 5.26)	119.18 (112.96 to 125.68)	71.48 (59.71 to 84.09)	-40.03 (-49.83 to -29.38)
**New Mexico**	110.94 (103.64 to 119.07)	95.39 (82.68 to 109.3)	-14.02 (-25.72 to 0.97)	11.89 (8.54 to 15.77)	12.81 (9.12 to 16.74)	7.76 (-5.23 to 20.93)	99.05 (92.06 to 105.97)	82.58 (69.8 to 95.65)	-16.64 (-29.83 to -0.04)
**New York**	132.59 (125.18 to 141)	75.18 (65.41 to 86.47)	-43.3 (-50.46 to -34.45)	13.33 (9.27 to 17.56)	12.96 (8.86 to 16.73)	-2.75 (-13.45 to 9.99)	119.27 (112.7 to 126.17)	62.22 (53 to 72.59)	-47.83 (-55.86 to -38.57)
**North Carolina**	155.95 (147.45 to 165.49)	114.52 (98.75 to 132.07)	-26.56 (-36.88 to -15.54)	13.56 (9.58 to 17.73)	14.13 (9.92 to 18.17)	4.19 (-8.25 to 18.39)	142.39 (134.01 to 151.26)	100.4 (85.04 to 117.45)	-29.49 (-40.62 to -17.08)
**North Dakota**	105.55 (98.95 to 112.54)	89.87 (79.63 to 102.45)	-14.85 (-25.55 to -1.9)	11.39 (7.94 to 14.93)	12.24 (8.6 to 15.65)	7.49 (-5.47 to 21.2)	94.16 (88.44 to 100.23)	77.63 (67.23 to 89.65)	-17.55 (-29.51 to -3.23)
**Ohio**	131.63 (123.97 to 140.04)	112.01 (97.45 to 127.92)	-14.91 (-25.72 to -2.94)	13.65 (9.75 to 18.01)	14.82 (10.7 to 19.14)	8.55 (-4.81 to 22.72)	117.98 (111.25 to 124.27)	97.19 (82.64 to 111.78)	-17.62 (-29.32 to -3.86)
**Oklahoma**	146.39 (137.96 to 155.25)	134.93 (117.63 to 156.15)	-7.82 (-19.49 to 6.79)	13.38 (9.69 to 17.47)	15.02 (10.63 to 19.57)	12.21 (-0.97 to 28.65)	133 (125.45 to 140.71)	119.92 (102.75 to 141.84)	-9.84 (-22.48 to 6.3)
**Oregon**	112.93 (105.91 to 120.48)	89.8 (77.43 to 104.98)	-20.49 (-31.23 to -7.3)	12.55 (8.89 to 16.26)	13.44 (9.45 to 17.53)	7.06 (-4.41 to 21.53)	100.38 (94.54 to 106.72)	76.36 (65 to 91.4)	-23.93 (-35.92 to -9.81)

**Pennsylvania**	125.26 (117.81 to 133.44)	93.12 (80.61 to 106.6)	-25.66 (-35.5 to -14.5)	13.41 (9.46 to 17.82)	14.05 (9.95 to 18.27)	4.83 (-8.36 to 19.71)	111.86 (104.75 to 119.03)	79.07 (67.8 to 92.66)	-29.31 (-39.8 to -16.69)
**Rhode Island**	102.53 (95.6 to 109.11)	66.14 (56.8 to 76.45)	-35.49 (-44.14 to -26)	12.58 (8.68 to 16.31)	12.71 (8.87 to 16.52)	1.04 (-9 to 14.17)	89.95 (83.4 to 95.69)	53.43 (45.01 to 61.95)	-40.6 (-49.76 to -30.31)
**South Carolina**	192.63 (182.22 to 204.13)	143.5 (122.97 to 164.24)	-25.51 (-36.32 to -14.26)	14.37 (10.11 to 18.82)	15.62 (11.16 to 20.13)	8.67 (-3.55 to 22.71)	178.26 (168.99 to 188.65)	127.88 (107.52 to 147.73)	-28.26 (-39.74 to -15.95)
**South Dakota**	109.94 (102.58 to 118.25)	95.63 (84.83 to 108.09)	-13.02 (-23.33 to -1.19)	12.27 (8.4 to 15.82)	12.88 (9.01 to 16.63)	5.01 (-5.99 to 16.47)	97.67 (91.21 to 104.43)	82.75 (72.74 to 94.81)	-15.28 (-26.87 to -2.16)
**Tennessee**	158.66 (149.6 to 167.31)	141.02 (122.19 to 160.79)	-11.12 (-22.71 to 2.14)	14.21 (9.96 to 18.84)	15.48 (10.98 to 20.1)	8.88 (-3.77 to 24.12)	144.45 (136.36 to 152.5)	125.54 (107.72 to 145.34)	-13.09 (-25.15 to 1.1)
**Texas**	139.96 (131.65 to 148.07)	110.67 (96.98 to 125.96)	-20.93 (-31.97 to -10.3)	14.22 (10.03 to 18.51)	14.65 (10.26 to 19.02)	3.05 (-9.59 to 16.48)	125.74 (118.65 to 133.24)	96.02 (82.67 to 109.92)	-23.64 (-35.35 to -11.48)
**Utah**	105.29 (98.4 to 112.35)	85.11 (75.46 to 96.37)	-19.17 (-29.07 to -7.76)	12.81 (8.87 to 16.79)	13.03 (9.03 to 17.01)	1.67 (-9.86 to 14.26)	92.48 (86.32 to 98.73)	72.08 (62.77 to 83.11)	-22.06 (-32.52 to -9.37)
**Vermont**	100.9 (93.95 to 107.96)	71.97 (63.96 to 81.1)	-28.67 (-36.29 to -19.45)	12.15 (8.63 to 15.81)	12.23 (8.74 to 15.77)	0.71 (-9.83 to 13.49)	88.76 (83.08 to 94.46)	59.74 (52.31 to 67.66)	-32.69 (-41.42 to -23.06)

**Virginia**	126.04 (118.94 to 133.54)	99.78 (84.9 to 114.42)	-20.84 (-32.21 to -8.59)	12.85 (9.03 to 17.02)	14.14 (9.62 to 18.36)	10.03 (-2.98 to 24.66)	113.19 (107.48 to 118.51)	85.64 (72.19 to 99.86)	-24.34 (-36.46 to -10.16)
**Washington**	113.46 (105.83 to 120.73)	79.5 (67.68 to 91.18)	-29.94 (-39.07 to -19.22)	12.84 (9.04 to 16.88)	12.84 (9.11 to 16.57)	0.02 (-11.09 to 11.76)	100.62 (94.41 to 106.88)	66.66 (55.68 to 77.58)	-33.76 (-44.03 to -22.06)
**West Virginia**	134.48 (125.73 to 144.52)	137.78 (118.75 to 159.7)	2.45 (-12.48 to 20.05)	12.84 (8.87 to 16.87)	14.88 (10.69 to 19.27)	15.88 (1.35 to 32.63)	121.64 (114.21 to 129.84)	122.9 (104.6 to 145.65)	1.03 (-15.08 to 19.93)
**Wisconsin**	108.41 (101.69 to 114.83)	80.88 (68.83 to 93.41)	-25.4 (-36.65 to -13.85)	11.09 (7.75 to 14.51)	11.43 (7.82 to 15.01)	3.11 (-8.48 to 16.54)	97.33 (91.46 to 102.6)	69.44 (58.59 to 81.77)	-28.65 (-41.09 to -15.91)
**Wyoming**	117.41 (109.54 to 125.69)	95.87 (85.58 to 107.27)	-18.35 (-27.11 to -9.07)	12.26 (8.69 to 16.09)	12.49 (8.78 to 16.35)	1.94 (-8.65 to 15.01)	105.16 (97.96 to 112.18)	83.38 (73.97 to 93.81)	-20.71 (-30.25 to -10.65)

2.9 Table S5: Age-standardized rate and percentage change of mortality, DALYs, YLDs, and YLLs for stroke over time by risk factor, both sexes, US, 1990-2021

Disease	Risk Factor	Death			DALYs			YLDs			YLLs		
		1990	2021	change, %	1990	2021	change, %	1990	2021	change, %	1990	2021	change, %
Stroke	**Air pollution**	4.26 (1.67 to 7.55)	0.99 (0.49 to 1.62)	-76.83 (-90.05 to -47.84)	90.8 (36.15 to 156.06)	22.13 (10.95 to 35.66)	-75.63 (-89.64 to -45.64)	15.54 (5.51 to 29.16)	5.1 (2.34 to 8.84)	-67.2 (-86.28 to -27.85)	75.25 (29.79 to 132.32)	17.03 (8.45 to 27.63)	-77.37 (-90.31 to -49.15)
	**Ambient particulate matter pollution**	4.25 (1.67 to 7.55)	0.99 (0.49 to 1.62)	-76.82 (-90.05 to -47.83)	90.74 (36.13 to 156.06)	22.12 (10.95 to 35.67)	-75.62 (-89.64 to -45.63)	15.53 (5.5 to 29.17)	5.1 (2.34 to 8.85)	-67.19 (-86.29 to -27.85)	75.2 (29.78 to 132.32)	17.02 (8.46 to 27.64)	-77.36 (-90.31 to -49.14)
	**Household air pollution from solid fuels**	0 (0 to 0.02)	0 (0 to 0)	-87.13 (-99.99 to -90.15)	0.05 (0 to 0.44)	0.01 (0 to 0.04)	-86.2 (-99.98 to -89.37)	0.01 (0 to 0.07)	0 (0 to 0.01)	-81.71 (-99.98 to -85.8)	0.05 (0 to 0.36)	0.01 (0 to 0.03)	-87.11 (-99.99 to -90.18)
	**Other environmental risks**	1.71 (-0.22 to 3.9)	1 (-0.13 to 2.28)	-41.49 (-46.35 to -37.15)	37.1 (-4.86 to 86.3)	20.58 (-2.66 to 47.82)	-44.52 (-48.3 to -40.9)	6.32 (-0.85 to 14.57)	4.53 (-0.6 to 10.38)	-28.32 (-33.08 to -22.85)	30.78 (-3.96 to 70.89)	16.05 (-2.04 to 37.23)	-47.85 (-51.72 to -44.23)
	**Lead exposure**	1.71 (-0.22 to 3.9)	1 (-0.13 to 2.28)	-41.49 (-46.35 to -37.15)	37.1 (-4.86 to 86.3)	20.58 (-2.66 to 47.82)	-44.52 (-48.3 to -40.9)	6.32 (-0.85 to 14.57)	4.53 (-0.6 to 10.38)	-28.32 (-33.08 to -22.85)	30.78 (-3.96 to 70.89)	16.05 (-2.04 to 37.23)	-47.85 (-51.72 to -44.23)
	**Tobacco**	6.35 (5.27 to 7.69)	3.15 (2.52 to 3.95)	-50.4 (-54.51 to -45.75)	194.01 (164.52	103.93 (85.2 to 126.66)	-46.43 (-49.88 to -42.91)	36.31 (26.21	26.23 (18.88	-27.76 (-32.69 to -22.54)	157.71 (134.45	77.7 (64.6 to 93.3)	-50.73 (-54.18 to -47.11)

				to 230.04)			to 48.61)	to 35.46)		to 184.1)		
**Smoking**	5.68 (4.77 to 6.82)	2.82 (2.28 to 3.53)	-50.34 (-54.89 to -45.24)	175.09 (150.7 to 204.79)	93.42 (77.59 to 112.83)	-46.65 (-50.41 to -42.84)	32.68 (23.77 to 43.52)	23.5 (16.95 to 31.52)	-28.1 (-33.35 to -22.59)	142.41 (123.38 to 164.33)	69.92 (58.96 to 83.31)	-50.9 (-54.45 to - 47.1)
**Secondhand smoke**	0.84 (0.57 to 1.13)	0.39 (0.26 to 0.53)	-53.77 (-57.38 to -49.75)	25.76 (17.42 to 34.85)	13.11 (8.73 to 17.94)	-49.11 (-52.6 to -44.83)	4.99 (3.09 to 7.45)	3.42 (2.12 to 5.03)	-31.53 (-36.87 to -26.16)	20.77 (14.21 to 27.68)	9.69 (6.58 to 13.19)	-53.34 (-56.7 to - 49.32)
**Alcohol use**	1.97 (0.31 to 5.18)	1.99 (0.37 to 4.26)	0.97 (-28.94 to 71.5)	48.32 (9.33 to 117.88)	48.87 (9.61 to 101.19)	1.14 (-21.46 to 51.92)	8.83 (0.3 to 24.59)	12.19 (0.49 to 29.66)	38.08 (10.24 to 112.07)	39.49 (8.09 to 93.67)	36.68 (8.68 to 71.88)	-7.12 (-31.47 to 37.23)
**Metabolic risks**	32.71 (26.84 to 37.23)	20.82 (16.53 to 24.29)	-36.34 (-40.26 to -33)	673.41 (565.47 to 765.78)	456.85 (375.93 to 531.73)	-32.16 (-35.62 to -29.16)	124.62 (89.39 to 162.92)	115.58 (82.11 to 152.35)	-7.25 (-12.55 to -1.8)	548.79 (452.51 to 623.08)	341.26 (276.39 to 396.23)	-37.81 (-41.16 to - 34.63)
**High fasting plasma glucose**	5.68 (4.45 to 7.04)	5.14 (4.02 to 6.34)	-9.49 (-16.94 to -0.83)	107.74 (83.88 to 132.1)	104.46 (82.29 to 126.35)	-3.05 (-10.13 to 4.97)	21.67 (14.32 to 29.72)	30.21 (20.41 to 40.39)	39.39 (28.01 to 52.39)	86.07 (67.29 to 105.63)	74.25 (59.51 to 89.91)	-13.73 (-20.66 to - 6.24)
**High systolic blood pressure**	26.38 (19.81 to 32.08)	15.77 (11.12 to 20.21)	-40.22 (-46.4 to -35.03)	536.58 (402.07 to 650.51)	335.94 (241.27 to 424.52)	-37.39 (-42.77 to -32.86)	89.77 (61.05 to 123.71)	75.6 (49.34 to 105.66)	-15.78 (-24.31 to -7.93)	446.81 (334.49 to 539.08)	260.35 (186 to 328.68)	-41.73 (-46.62 to - 37.34)

**High body-mass index**	2.4 (0.18 to 5.22)	2.35 (0.14 to 4.79)	-2.07 (-16.87 to 10.07)	72.65 (4.47 to 153.46)	77.6 (4.45 to 151.21)	6.8 (-8.02 to 23.02)	16.51 (1.99 to 33.47)	23.13 (2.78 to 44.75)	40.1 (22.65 to 59.41)	56.14 (2.87 to 122.39)	54.46 (1.68 to 109.85)	-2.99 (-18.02 to 11.62)
**Dietary risks**	2.95 (0.06 to 6.86)	2.14 (-0.07 to 5.09)	-27.43 (-72.09 to 3.98)	69.88 (-8.02 to 159.67)	62.89 (-0.75 to 133.26)	-10 (-50.93 to 46)	23.14 (3.31 to 42.47)	26.43 (6.02 to 46.69)	14.25 (1.75 to 42.42)	46.74 (-33.99 to 125.09)	36.46 (-22.43 to 96.46)	-22 (-142.23 to 52.93)
**Diet low in fruits**	1.23 (0.08 to 2.3)	0.85 (0.03 to 1.59)	-31.5 (-45.23 to -23.24)	40.97 (3.28 to 72.34)	28.73 (2.11 to 50.41)	-29.87 (-35.43 to -24.85)	6.23 (2.04 to 10.77)	5.75 (1.8 to 9.87)	-7.61 (-13.84 to -0.1)	34.74 (1.09 to 63.25)	22.98 (0.18 to 41.96)	-33.86 (-41.05 to -27.91)
**Diet low in vegetables**	0.19 (-0.08 to 0.44)	0.18 (-0.04 to 0.4)	-4.26 (-83.71 to 98)	5.54 (1.73 to 9.33)	6.62 (2.78 to 10.85)	19.46 (-6.46 to 91.6)	1.99 (1.09 to 3.24)	2.81 (1.58 to 4.36)	41.01 (24.44 to 62.24)	3.55 (0.49 to 6.55)	3.82 (1.13 to 6.92)	7.39 (-40.71 to 101.84)
**Diet low in whole grains**	0.58 (-0.56 to 2.14)	0.33 (-0.34 to 1.25)	-41.99 (-52.67 to -21.36)	18.97 (-18.32 to 56.3)	14.77 (-14.87 to 42.51)	-22.17 (-27.72 to -3.69)	7.39 (-7.51 to 19.73)	7.86 (-8.26 to 21.07)	6.32 (-0.3 to 15.51)	11.58 (-11.06 to 35.89)	6.91 (-6.71 to 21.24)	-40.36 (-44.52 to -31.13)
**Diet high in red meat**	-1.44 (-6.15 to 2.12)	-1.12 (-4.79 to 1.66)	-22.24 (-62.51 to 13.22)	-48.61 (-215.25 to 70.36)	-35.57 (-159.37 to 52.15)	-26.84 (-55 to -11.87)	-0.57 (-10.72 to 6.63)	-0.86 (-11.91 to 6.08)	50.72 (-196.24 to 182.61)	-48.05 (-203.24 to 67)	-34.71 (-150.9 to 48.62)	-27.76 (-54.35 to -7.54)
**Diet high in processed meat**	0.76 (0.17 to 1.32)	0.44 (0.11 to 0.75)	-41.63 (-52.79 to -26.89)	16.14 (3.54 to 28.51)	12.48 (2.88 to 22.2)	-22.68 (-35.03 to -7.33)	4.3 (0.9 to 8.07)	5.4 (1.23 to 9.99)	25.65 (6.2 to 48.65)	11.84 (2.67 to 20.55)	7.08 (1.75 to 12.12)	-40.22 (-50.18 to -27.13)

**Diet high in sugar-sweetened beverages**	0.17 (0.08 to 0.26)	0.1 (0.05 to 0.17)	-37.25 (-54.95 to -14.54)	3.53 (1.6 to 5.6)	3.08 (1.47 to 4.87)	-12.77 (-31.46 to 8.96)	0.97 (0.42 to 1.71)	1.41 (0.65 to 2.35)	44.76 (15.87 to 81.5)	2.56 (1.18 to 4.02)	1.67 (0.83 to 2.62)	-34.69 (-50.12 to -16.63)
**Diet low in fiber**	0.97 (-0.18 to 2.26)	0.45 (-0.09 to 1.04)	-53.88 (-62.42 to -43.13)	32.25 (-6.86 to 66.82)	15.02 (-3.04 to 33.3)	-53.42 (-60.72 to -45.72)	6.25 (-0.81 to 12.97)	3.7 (-0.45 to 7.9)	-40.81 (-51.01 to -31.46)	25.99 (-5.97 to 55.05)	11.32 (-2.62 to 25.5)	-56.45 (-63.41 to -48.67)
**Diet low in polyunsaturated fatty acids**	0 (0 to 0.01)	0 (0 to 0)	-56.47 (-62.19 to -51.24)	0.06 (0.02 to 0.11)	0.03 (0.01 to 0.06)	-50.97 (-56.04 to -45.55)	0.02 (0 to 0.03)	0.01 (0 to 0.02)	-31.45 (-38.1 to -23.94)	0.04 (0.01 to 0.08)	0.02 (0 to 0.03)	-59.02 (-63.69 to -54.68)
**Diet high in sodium**	1.41 (0 to 5.31)	1.31 (0.03 to 4.33)	-6.8 (-24.87 to 926.74)	31.17 (0.07 to 114.04)	33.58 (0.92 to 103.8)	7.72 (-15.46 to 1296.44)	5.31 (0.01 to 20.44)	7.7 (0.2 to 24.3)	45.03 (13.33 to 1633.03)	25.86 (0.06 to 94.45)	25.88 (0.72 to 80.82)	0.07 (-21.42 to 1226.14)
**Low physical activity**	0.89 (-0.67 to 2.59)	0.53 (-0.52 to 1.58)	-40.63 (-96.77 to 17.27)	20.69 (-0.14 to 45.31)	15.04 (0.79 to 32.82)	-27.29 (-54.79 to 7.16)	6.16 (2.03 to 11.5)	6.58 (2.44 to 11.92)	6.89 (-15.88 to 45.42)	14.53 (-2.86 to 34.53)	8.46 (-2.71 to 20.65)	-41.78 (-69.95 to -19.94)
**All risk factors**	36.55 (31.56 to 40.36)	23.32 (19.55 to 26.49)	-36.19 (-39.5 to -33.15)	775.9 (692.82 to 847.01)	522.95 (453.65 to 586.41)	-32.6 (-35.65 to -29.66)	138.46 (99.93 to 176.54)	128 (93.41 to 165.69)	-7.56 (-12.69 to -2.69)	637.44 (567.24 to 696.5)	394.95 (338.33 to 445.74)	-38.04 (-41.13 to -34.99)
**Environmental/occupational risks**	8.8 (5.69 to 12.02)	4.1 (2.75 to 5.54)	-53.39 (-65.95 to -37.99)	177.98 (113.11 to 245.15)	79.57 (52.2 to 109.09)	-55.29 (-68.04 to -39.32)	21.24 (9.31 to 36.83)	9.48 (3.41 to 16.98)	-55.39 (-77.75 to -27.03)	156.74 (102.77 to 213.1)	70.09 (48.46 to 93.8)	-55.28 (-67.48 to -40.54)

**Behavioral risks**	11.04 (7.37 to 14.93)	7.05 (4.29 to 10.17)	-36.16 (-46.2 to -26.63)	296.29 (224.52 to 378.66)	201.47 (140.03 to 271.99)	-32 (-40.24 to -25.09)	62.06 (40.92 to 85.64)	59.01 (38.06 to 83.6)	-4.92 (-14.29 to 4.3)	234.23 (169.94 to 302.32)	142.47 (90.2 to 199.09)	-39.18 (-49.87 to -31.51)
**Non-optimal temperature**	3.46 (2.49 to 4.31)	2.3 (1.7 to 2.89)	-33.52 (-37.97 to -28.82)	61.82 (45.35 to 77.07)	40.03 (29.86 to 50.2)	-35.25 (-39.72 to -30.69)	N/A	N/A	N/A	61.82 (45.35 to 77.07)	40.03 (29.86 to 50.2)	-35.25 (-39.72 to -30.69)
**High temperature**	0.13 (-0.29 to 0.67)	0.15 (-0.19 to 0.59)	10.1 (-146.43 to 148.37)	2.49 (-5.14 to 12.57)	2.65 (-3.21 to 10.61)	6.44 (-150.76 to 137.17)	N/A	N/A	N/A	2.49 (-5.14 to 12.57)	2.65 (-3.21 to 10.61)	6.44 (-150.76 to 137.17)
**Low temperature**	3.33 (2.73 to 3.88)	2.16 (1.75 to 2.48)	-35.19 (-39.07 to -31)	59.48 (49.55 to 68.41)	37.53 (31.75 to 43.03)	-36.9 (-40.55 to -33.02)	N/A	N/A	N/A	59.48 (49.55 to 68.41)	37.53 (31.75 to 43.03)	-36.9 (-40.55 to -33.02)
**Kidney dysfunction**	4.2 (2.54 to 5.95)	3.01 (1.82 to 4.26)	-28.36 (-32.04 to -24.95)	80.28 (54.26 to 107.52)	60.83 (42.37 to 80.94)	-24.22 (-27.24 to -20.94)	14.23 (8.97 to 20.47)	14.54 (9.51 to 20.76)	2.18 (-3.46 to 8.84)	66.05 (43.55 to 88.19)	46.29 (31.19 to 61.48)	-29.91 (-33.32 to -26.57)
**High LDL cholesterol**	8.89 (2.8 to 15.06)	4.11 (1.19 to 7.34)	-53.79 (-57.67 to -50.87)	189.56 (68.43 to 303.9)	105.08 (36.77 to 172.19)	-44.57 (-48.48 to -41.32)	54.34 (21.31 to 87.72)	44.1 (16.56 to 71.59)	-18.85 (-25.18 to -13.43)	135.22 (46.17 to 220.41)	60.97 (20.13 to 103.73)	-54.91 (-58.03 to -52.45)
**Particulate matter pollution**	4.26 (1.67 to 7.55)	0.99 (0.49 to 1.62)	-76.83 (-90.05 to -47.84)	90.8 (36.15 to 156.06)	22.13 (10.95 to 35.66)	-75.63 (-89.64 to -45.64)	15.54 (5.51 to 29.16)	5.1 (2.34 to 8.84)	-67.2 (-86.28 to -27.85)	75.25 (29.79 to 132.32)	17.03 (8.45 to 27.63)	-77.37 (-90.31 to -49.15)

	**Air pollution**	2.82 (1.1 to 5)	0.56 (0.28 to 0.94)	-80.03 (-91.43 to -54.8)	52.16 (20.51 to 90.74)	11.55 (5.61 to 18.86)	-77.85 (-90.65 to -50.68)	12.64 (4.44 to 23.58)	4.05 (1.84 to 7.08)	-67.92 (-86.6 to -29.25)	39.53 (15.5 to 69.98)	7.5 (3.72 to 12.29)	-81.03 (-91.87 to -57.11)
	**Ambient particulate matter pollution**	2.82 (1.1 to 5)	0.56 (0.28 to 0.94)	-80.02 (-91.43 to -54.79)	52.13 (20.5 to 90.73)	11.55 (5.61 to 18.87)	-77.85 (-90.65 to -50.69)	12.63 (4.44 to 23.58)	4.05 (1.84 to 7.08)	-67.91 (-86.61 to -29.26)	39.5 (15.5 to 69.98)	7.5 (3.72 to 12.29)	-81.02 (-91.87 to -57.1)
	**Household air pollution from solid fuels**	0 (0 to 0.01)	0 (0 to 0)	-88.99 (-99.99 to -91.59)	0.03 (0 to 0.26)	0 (0 to 0.02)	-87.63 (-99.99 to -90.48)	0.01 (0 to 0.06)	0 (0 to 0.01)	-82.11 (-99.98 to -86.13)	0.02 (0 to 0.19)	0 (0 to 0.01)	-89.33 (-99.99 to -91.84)
**Ischemic stroke**	**Other environmental risks**	1.11 (-0.15 to 2.53)	0.59 (-0.08 to 1.34)	-47.09 (-52 to -42.6)	21.23 (-2.84 to 49.09)	11.37 (-1.48 to 26.22)	-46.42 (-50.39 to -42.72)	5.18 (-0.7 to 11.9)	3.69 (-0.49 to 8.47)	-28.68 (-34.45 to -22.42)	16.05 (-2.09 to 36.64)	7.68 (-0.98 to 17.52)	-52.15 (-56.25 to -48.38)
	**Lead exposure**	1.11 (-0.15 to 2.53)	0.59 (-0.08 to 1.34)	-47.09 (-52 to -42.6)	21.23 (-2.84 to 49.09)	11.37 (-1.48 to 26.22)	-46.42 (-50.39 to -42.72)	5.18 (-0.7 to 11.9)	3.69 (-0.49 to 8.47)	-28.68 (-34.45 to -22.42)	16.05 (-2.09 to 36.64)	7.68 (-0.98 to 17.52)	-52.15 (-56.25 to -48.38)
	**Tobacco**	3.17 (2.5 to 4.01)	1.31 (0.98 to 1.72)	-58.57 (-63.5 to -52.74)	85.73 (70.45 to 105.48)	43.49 (34.19 to 55.61)	-49.27 (-53.4 to -45.42)	28.08 (20 to 38.03)	20.14 (14.41 to 27.31)	-28.3 (-33.58 to -22.54)	57.64 (47.51 to 70.22)	23.36 (18.62 to 29.47)	-59.48 (-62.95 to -55.58)
	**Smoking**	2.8 (2.21 to 3.51)	1.16 (0.88 to 1.53)	-58.4 (-64.02 to -51.98)	76.97 (63.36 to 93.93)	38.99 (30.86 to 49.19)	-49.34 (-53.81 to -45.14)	25.29 (18.08 to 34.03)	18.06 (12.95 to 24.43)	-28.58 (-34.3 to -22.5)	51.68 (42.85 to 62.54)	20.93 (16.75 to 26.42)	-59.5 (-63.31 to -55.23)

**Secondhand smoke**	0.42 (0.28 to 0.59)	0.16 (0.11 to 0.23)	-61.37 (-64.93 to -57.01)	11.1 (7.4 to 15.44)	5.41 (3.57 to 7.6)	-51.28 (-54.97 to -47.27)	3.8 (2.34 to 5.68)	2.58 (1.59 to 3.81)	-31.94 (-37.67 to -26.26)	7.3 (4.95 to 9.91)	2.82 (1.9 to 3.93)	-61.34 (-64.68 to -57.8)
**Alcohol use**	1.26 (-0.11 to 3.99)	1.17 (-0.13 to 2.99)	-7.08 (-33.94 to 80.77)	27.91 (-3.28 to 84.63)	27.44 (-3.84 to 71.56)	-1.68 (-25.51 to 64.24)	7.61 (-1.07 to 22.98)	10.45 (-1.76 to 26.68)	37.39 (10.01 to 110.45)	20.3 (-2.19 to 61.41)	16.99 (-2.23 to 43.1)	-16.31 (-37.43 to 44.55)
**Metabolic risks**	23.36 (19.3 to 26.67)	13.32 (10.49 to 15.55)	-42.99 (-46.84 to -39.75)	437.12 (367.62 to 500.09)	276.15 (226.9 to 321.08)	-36.82 (-40.04 to -33.86)	107.57 (76.87 to 140.89)	98.21 (70.14 to 128.51)	-8.7 (-14.49 to -2.94)	329.55 (274.15 to 371.44)	177.94 (146.18 to 204.9)	-46 (-49.16 to -43.36)
**High fasting plasma glucose**	4.97 (3.75 to 6.22)	4.36 (3.21 to 5.33)	-12.28 (-19.75 to -3.86)	91.24 (70.01 to 114.66)	86.66 (66.94 to 106.19)	-5.02 (-12.02 to 3.07)	20.69 (13.59 to 28.32)	28.77 (19.41 to 38.76)	39.02 (27.45 to 52.15)	70.54 (54.28 to 87.5)	57.89 (44.34 to 70.43)	-17.94 (-24.45 to -10.54)
**High systolic blood pressure**	17.85 (13.35 to 21.68)	9.28 (6.46 to 11.91)	-47.98 (-54.56 to -42.43)	322.59 (240.9 to 391.42)	181.59 (129.21 to 233.07)	-43.71 (-49.17 to -39.3)	74.38 (50.58 to 103.19)	60.78 (39.99 to 84.18)	-18.28 (-27.09 to -9.73)	248.21 (187.32 to 299.78)	120.81 (85.58 to 154.56)	-51.33 (-56.17 to -47.08)
**High body-mass index**	1.53 (0.22 to 3.14)	1.3 (0.19 to 2.72)	-14.82 (-25.58 to -5.6)	41.12 (6.04 to 81.01)	41.25 (6.24 to 76.92)	0.32 (-11.93 to 12.15)	13.92 (2.05 to 27.59)	19.14 (2.96 to 36.02)	37.46 (21.32 to 55.35)	27.2 (4.06 to 54.36)	22.12 (3.4 to 43)	-18.68 (-28.57 to -9.91)
**Dietary risks**	2.82 (0.32 to 5.92)	1.77 (0.22 to 3.63)	-37.13 (-48.71 to -19.13)	71.83 (2.53 to 134.77)	56.63 (5.4 to 102.33)	-21.16 (-29.68 to 2.05)	23.5 (-1.65 to 44.52)	26.18 (0.45 to 48.38)	11.4 (-15.57 to 41.79)	48.33 (4.14 to 95.77)	30.46 (3.75 to 57.94)	-36.99 (-44.88 to -17.85)

**Diet low in fruits**	0.28 (-0.07 to 0.61)	0.14 (-0.05 to 0.32)	-50.31 (-65.06 to - 36.83)	9.21 (3.92 to 15.18)	6.11 (3.02 to 9.71)	-33.64 (-39.38 to - 18.88)	3.63 (2.03 to 5.63)	3.27 (1.79 to 5)	-9.81 (-16.73 to - 0.49)	5.59 (1.46 to 9.97)	2.84 (0.78 to 5.15)	-49.13 (-53.93 to - 43.1)
**Diet low in vegetables**	0.19 (-0.08 to 0.43)	0.14 (-0.07 to 0.32)	-26.2 (-48.37 to - 1.73)	5.48 (1.67 to 9.24)	5.31 (2.07 to 8.63)	-3.1 (-14.09 to 31.27)	1.98 (1.09 to 3.23)	2.67 (1.54 to 4.13)	34.67 (21.23 to 53.29)	3.49 (0.43 to 6.45)	2.63 (0.33 to 4.91)	-24.57 (-35.41 to - 11.71)
**Diet low in whole grains**	0.58 (-0.56 to 2.14)	0.33 (-0.34 to 1.25)	-41.99 (-52.67 to - 21.36)	18.97 (-18.32 to 56.3)	14.77 (-14.87 to 42.51)	-22.17 (-27.72 to - 3.69)	7.39 (-7.51 to 19.73)	7.86 (-8.26 to 21.07)	6.32 (-0.3 to 15.51)	11.58 (-11.06 to 35.89)	6.91 (-6.71 to 21.24)	-40.36 (-44.52 to - 31.13)
**Diet high in red meat**	0.12 (-0.07 to 0.46)	0.07 (-0.04 to 0.27)	-40.17 (-76.5 to 55.03)	8.34 (-5.08 to 22.86)	6.24 (-4.06 to 17.3)	-25.13 (-49.42 to 0.33)	4.23 (-2.68 to 11.01)	3.87 (-2.48 to 10.77)	-8.7 (-37.56 to 16.44)	4.1 (-2.38 to 11.83)	2.38 (-1.42 to 7.08)	-42.09 (-62.7 to - 15.27)
**Diet high in processed meat**	0.76 (0.17 to 1.32)	0.44 (0.11 to 0.75)	-41.63 (-52.79 to - 26.89)	16.14 (3.54 to 28.51)	12.48 (2.88 to 22.2)	-22.68 (-35.03 to - 7.33)	4.3 (0.9 to 8.07)	5.4 (1.23 to 9.99)	25.65 (6.2 to 48.65)	11.84 (2.67 to 20.55)	7.08 (1.75 to 12.12)	-40.22 (-50.18 to - 27.13)
**Diet high in sugar-sweetened beverages**	0.17 (0.08 to 0.26)	0.1 (0.05 to 0.17)	-37.25 (-54.95 to - 14.54)	3.53 (1.6 to 5.6)	3.08 (1.47 to 4.87)	-12.77 (-31.46 to 8.96)	0.97 (0.42 to 1.71)	1.41 (0.65 to 2.35)	44.76 (15.87 to 81.5)	2.56 (1.18 to 4.02)	1.67 (0.83 to 2.62)	-34.69 (-50.12 to - 16.63)
**Diet low in fiber**	0.33 (-0.15 to 0.99)	0.12 (-0.07 to 0.37)	-64.65 (-87.85 to - 44.83)	10.87 (-0.49 to 23.75)	4.78 (-0.15 to 10.67)	-56.02 (-62.87 to - 45.33)	4.43 (-0.22 to 9.03)	2.55 (-0.09 to 5.21)	-42.47 (-52.52 to - 32.67)	6.44 (-0.56 to 15.64)	2.23 (-0.17 to 5.54)	-65.36 (-79.92 to - 57.87)
**Diet low in polyunsaturated fatty acids**	0 (0 to 0.01)	0 (0 to 0)	-56.47 (-62.19 to - 51.24)	0.06 (0.02 to 0.11)	0.03 (0.01 to 0.06)	-50.97 (-56.04 to - 45.55)	0.02 (0 to 0.03)	0.01 (0 to 0.02)	-31.45 (-38.1 to - 23.94)	0.04 (0.01 to 0.08)	0.02 (0 to 0.03)	-59.02 (-63.69 to - 54.68)

**Diet high in sodium**	0.9 (0 to 3.48)	0.67 (0.01 to 2.32)	-26.01 (-40.05 to 594.81)	17.52 (0.04 to 65.1)	16.26 (0.36 to 52.18)	-7.19 (-26.33 to 1064.89)	4.33 (0.01 to 16.67)	6.16 (0.16 to 19.58)	42.13 (10.79 to 1651.61)	13.18 (0.02 to 49.73)	10.1 (0.19 to 33.17)	-23.4 (-39.15 to 805.85)
**Low physical activity**	0.89 (-0.67 to 2.59)	0.53 (-0.52 to 1.58)	-40.63 (-96.77 to 17.27)	20.69 (-0.14 to 45.31)	15.04 (0.79 to 32.82)	-27.29 (-54.79 to 7.16)	6.16 (2.03 to 11.5)	6.58 (2.44 to 11.92)	6.89 (-15.88 to 45.42)	14.53 (-2.86 to 34.53)	8.46 (-2.71 to 20.65)	-41.78 (-69.95 to - 19.94)
**All risk factors**	25.19 (21.25 to 28.02)	14.38 (11.53 to 16.45)	-42.93 (-46.4 to - 40.26)	474.08 (413.42 to 530.79)	300.92 (255.35 to 343.84)	-36.53 (-39.76 to - 33.97)	116.85 (84.35 to 150.64)	107.29 (78.11 to 138.66)	-8.18 (-13.81 to - 2.97)	357.23 (310.06 to 391.43)	193.63 (160.74 to 216.96)	-45.8 (-48.72 to - 43.62)
**Environmental/occupational risks**	5.82 (3.73 to 8.03)	2.36 (1.53 to 3.22)	-59.5 (-70.29 to - 46.1)	99.46 (61.55 to 139.57)	38.88 (23.61 to 55.92)	-60.91 (-72.4 to - 46.88)	17.31 (7.42 to 30.09)	7.62 (2.72 to 13.78)	-55.95 (-78.18 to - 28.67)	82.16 (53.13 to 112.01)	31.26 (20.91 to 42.39)	-61.96 (-72.05 to - 49.4)
**Behavioral risks**	7.19 (3.89 to 10.59)	4.22 (1.92 to 6.65)	-41.26 (-52.84 to - 30.1)	174.12 (113.72 to 239.1)	119.09 (68.91 to 169.9)	-31.6 (-40.79 to - 23.92)	53.17 (32.71 to 77.64)	51.31 (29.6 to 74.71)	-3.49 (-14.49 to 6.21)	120.94 (77.53 to 166.53)	67.77 (37.55 to 98.06)	-43.96 (-52.62 to - 36.52)
**Non-optimal temperature**	2.3 (1.66 to 2.89)	1.31 (0.94 to 1.67)	-42.96 (-47.04 to - 38.8)	32.41 (23.36 to 40.5)	17.47 (12.9 to 22.05)	-46.1 (-49.88 to - 42.13)	N/A	N/A	N/A	32.41 (23.36 to 40.5)	17.47 (12.9 to 22.05)	-46.1 (-49.88 to - 42.13)
**High temperature**	0.09 (-0.19 to 0.44)	0.09 (-0.11 to 0.35)	-2.43 (-150.39 to 111.31)	1.27 (-2.7 to 6.33)	1.17 (-1.4 to 4.64)	-7.88 (-145.61 to 105.3)	N/A	N/A	N/A	1.27 (-2.7 to 6.33)	1.17 (-1.4 to 4.64)	-7.88 (-145.61 to 105.3)

	**Low temperature**	2.22 (1.79 to 2.6)	1.23 (0.98 to 1.43)	-44.48 (-48.27 to - 40.84)	31.21 (25.55 to 36.34)	16.37 (13.31 to 18.83)	-47.57 (-50.89 to - 44.3)	N/A	N/A	N/A	31.21 (25.55 to 36.34)	16.37 (13.31 to 18.83)	-47.57 (-50.89 to - 44.3)
	**Kidney dysfunction**	3.1 (1.79 to 4.51)	1.97 (1.13 to 2.89)	-36.41 (-40.55 to - 33.05)	54.9 (35.56 to 75.49)	38.11 (25.57 to 51.87)	-30.58 (-33.97 to - 27.39)	12.62 (7.89 to 18.3)	12.67 (8.27 to 18.19)	0.38 (-5.77 to 7.25)	42.28 (26.08 to 59.11)	25.44 (15.69 to 35.71)	-39.83 (-43.48 to - 36.74)
	**High LDL cholesterol**	8.89 (2.8 to 15.06)	4.11 (1.19 to 7.34)	-53.79 (-57.67 to - 50.87)	189.56 (68.43 to 303.9)	105.08 (36.77 to 172.19)	-44.57 (-48.48 to - 41.32)	54.34 (21.31 to 87.72)	44.1 (16.56 to 71.59)	-18.85 (-25.18 to - 13.43)	135.22 (46.17 to 220.41)	60.97 (20.13 to 103.73)	-54.91 (-58.03 to - 52.45)
	**Particulate matter pollution**	2.82 (1.1 to 5)	0.56 (0.28 to 0.94)	-80.03 (-91.43 to - 54.8)	52.16 (20.51 to 90.74)	11.55 (5.61 to 18.86)	-77.85 (-90.65 to - 50.68)	12.64 (4.44 to 23.58)	4.05 (1.84 to 7.08)	-67.92 (-86.6 to - 29.25)	39.53 (15.5 to 69.98)	7.5 (3.72 to 12.29)	-81.03 (-91.87 to - 57.11)
**Intracerebral hemorrhage**	**Air pollution**	1.08 (0.43 to 1.9)	0.32 (0.16 to 0.52)	-70.43 (-87.28 to - 33.61)	26.51 (10.69 to 45.94)	7.48 (3.72 to 12.06)	-71.78 (-87.92 to - 36.58)	1.75 (0.65 to 3.31)	0.64 (0.29 to 1.12)	-63.53 (-84.39 to - 21.25)	24.76 (9.9 to 43.21)	6.84 (3.4 to 11.05)	-72.36 (-88.16 to - 37.9)
	**Ambient particulate matter pollution**	1.08 (0.43 to 1.9)	0.32 (0.16 to 0.52)	-70.42 (-87.28 to - 33.59)	26.49 (10.68 to 45.94)	7.48 (3.72 to 12.06)	-71.77 (-87.92 to - 36.56)	1.75 (0.65 to 3.3)	0.64 (0.29 to 1.12)	-63.52 (-84.39 to - 21.19)	24.74 (9.89 to 43.21)	6.84 (3.4 to 11.02)	-72.36 (-88.16 to - 37.88)
	**Household air pollution from solid fuels**	0 (0 to 0.01)	0 (0 to 0)	-83.37 (-99.98 to - 87.31)	0.02 (0 to 0.12)	0 (0 to 0.01)	-83.89 (-99.98 to - 87.67)	0 (0 to 0.01)	0 (0 to 0)	-79.74 (-99.98 to - 84.21)	0.01 (0 to 0.11)	0 (0 to 0.01)	-84.18 (-99.98 to - 87.98)
	**Other environmental risks**	0.45 (-0.06 to 1.04)	0.32 (-0.04 to 0.73)	-30.21 (-35.85 to - 25.15)	11.19 (-1.44 to 25.89)	6.79 (-0.85 to 15.89)	-39.38 (-43.64 to - 35.11)	0.7 (-0.09 to 1.65)	0.53 (-0.07 to 1.24)	-24.12 (-30.36 to - 17.46)	10.49 (-1.34	6.25 (-0.78 to 14.6)	-40.4 (-44.78 to - 35.87)

										to 24.38)		
**Lead exposure**	0.45 (-0.06 to 1.04)	0.32 (-0.04 to 0.73)	-30.21 (-35.85 to - 25.15)	11.19 (-1.44 to 25.89)	6.79 (-0.85 to 15.89)	-39.38 (-43.64 to - 35.11)	0.7 (-0.09 to 1.65)	0.53 (-0.07 to 1.24)	-24.12 (-30.36 to - 17.46)	10.49 (-1.34 to 24.38)	6.25 (-0.78 to 14.6)	-40.4 (-44.78 to - 35.87)
**Tobacco**	2.23 (1.89 to 2.6)	1.33 (1.1 to 1.61)	-40.43 (-44.82 to - 35.88)	70.03 (60.16 to 81.41)	40.93 (33.79 to 48.67)	-41.56 (-45.45 to - 37.57)	4.83 (3.45 to 6.5)	3.57 (2.51 to 4.86)	-26.13 (-31.7 to - 19.96)	65.2 (56.08 to 75.44)	37.36 (30.86 to 44.17)	-42.71 (-46.65 to - 38.68)
**Smoking**	2.02 (1.74 to 2.33)	1.2 (0.99 to 1.45)	-40.65 (-45.34 to - 35.81)	63.59 (55.27 to 73.34)	36.93 (31.07 to 43.71)	-41.92 (-46.01 to - 37.78)	4.34 (3.09 to 5.8)	3.19 (2.27 to 4.3)	-26.61 (-32.73 to - 20.17)	59.25 (51.64 to 67.71)	33.75 (28.34 to 39.64)	-43.04 (-47.2 to - 38.89)
**Secondhand smoke**	0.28 (0.19 to 0.38)	0.16 (0.11 to 0.21)	-43.77 (-48.31 to - 38.77)	9.17 (6.25 to 12.37)	5.09 (3.44 to 6.95)	-44.53 (-48.66 to - 39.47)	0.7 (0.43 to 1.04)	0.49 (0.3 to 0.72)	-30.66 (-36.77 to - 24.27)	8.47 (5.82 to 11.26)	4.6 (3.12 to 6.27)	-45.67 (-50.03 to - 40.68)
**Alcohol use**	0.72 (0.03 to 1.67)	0.82 (0.03 to 1.81)	15.1 (-6.79 to 66.82)	20.41 (0.68 to 44.98)	21.43 (0.65 to 46.09)	4.98 (-10.18 to 40.14)	1.22 (0.03 to 3.01)	1.74 (0.05 to 3.81)	42.36 (19.76 to 91.75)	19.19 (0.65 to 42.41)	19.69 (0.6 to 42.48)	2.6 (-12.46 to 36.86)
**Metabolic risks**	7.3 (5.92 to 8.49)	5.87 (4.57 to 6.98)	-19.58 (-25.16 to - 14.51)	171.78 (138.81 to 200.05)	133.84 (105.52 to 159.58)	-22.09 (-27.17 to - 17.03)	10.75 (7.36 to 14.52)	11.25 (7.69 to 15.29)	4.64 (-2.71 to 11.9)	161.03 (130.1 to 186.84)	122.59 (96.92 to 145.62)	-23.87 (-28.87 to - 18.89)

**High fasting plasma glucose**	0.72 (0.39 to 1.08)	0.79 (0.43 to 1.15)	9.89 (-1.14 to 21.52)	16.51 (8.81 to 24.76)	17.8 (9.85 to 25.94)	7.86 (-2.36 to 19.52)	0.98 (0.49 to 1.59)	1.44 (0.74 to 2.31)	47.23 (33.85 to 62.55)	15.53 (8.29 to 23.32)	16.36 (8.99 to 23.77)	5.38 (-4.69 to 16.77)
**High systolic blood pressure**	6.53 (4.94 to 7.88)	4.92 (3.5 to 6.19)	-24.77 (-31.44 to -18.78)	152.22 (113.73 to 183.69)	110.59 (76.88 to 138.97)	-27.35 (-33.89 to -21.36)	9.35 (6.19 to 13.05)	9.15 (5.83 to 13.01)	-2.14 (-10.42 to 6.64)	142.87 (107.44 to 172.13)	101.44 (70.79 to 127.69)	-29 (-35.47 to -23.22)
**High body-mass index**	0.59 (-0.01 to 1.36)	0.74 (-0.03 to 1.55)	26.95 (6.57 to 76.82)	19.61 (-0.56 to 44.6)	24.04 (-1.04 to 49.57)	22.56 (2.83 to 72.9)	1.5 (-0.04 to 3.48)	2.31 (-0.1 to 5.04)	54.2 (29.83 to 113)	18.12 (-0.52 to 41.09)	21.73 (-0.94 to 44.68)	19.95 (0.52 to 69.24)
**Dietary risks**	0.14 (-1.92 to 1.86)	0.3 (-1.35 to 1.84)	119.86 (-361.81 to 200.53)	0.05 (-68.81 to 53.5)	5.42 (-47.95 to 49.15)	10438.53 (-201.91 to 290.89)	-0.2 (-5.69 to 3.87)	0.24 (-4.96 to 4.35)	-219.57 (-326.66 to 191.44)	0.25 (-64.04 to 50.04)	5.19 (-43.06 to 44.94)	1975.66 (-215.97 to 261.31)
**Diet low in fruits**	0.67 (-0.04 to 1.29)	0.51 (-0.03 to 1)	-23.83 (-30.31 to -19.1)	20.23 (-1.47 to 37.78)	15.1 (-1.04 to 28.26)	-25.36 (-30.85 to -20.87)	1.53 (-0.12 to 3.11)	1.45 (-0.11 to 2.9)	-5.35 (-11.62 to 1.11)	18.7 (-1.35 to 35.28)	13.65 (-0.93 to 25.95)	-26.99 (-32.5 to -22.25)
**Diet low in vegetables**	0 (0 to 0.01)	0.03 (0 to 0.07)	1164.66 (732.93 to 1868.98)	0.05 (-0.01 to 0.1)	0.9 (-0.09 to 1.8)	1757.06 (1191.48 to 2581.89)	0 (0 to 0.01)	0.08 (-0.01 to 0.17)	2134.62 (1436.05 to 3200.43)	0.04 (0 to 0.1)	0.82 (-0.08 to 1.64)	1725.87 (1165.1 to 2565.07)
**Diet high in red meat**	-1.06 (-4.3 to 1.49)	-0.85 (-3.46 to 1.21)	-20.16 (-27.19 to 21450.31)	-35.28 (-143.91 to 47.36)	-27.44 (-113.2 to 36.88)	-22.21 (-28.26 to 25998.98)	-2.81 (-11.77 to 3.43)	-2.73 (-11.36 to 3.42)	-2.93 (-10.3 to 27542.57)	-32.47 (-131.69 to 43.91)	-24.72 (-103.71 to 33.59)	-23.88 (-30.41 to 25864.5)

**Diet low in fiber**	0.44 (-0.13 to 0.97)	0.24 (-0.07 to 0.54)	-46.54 (-54.71 to - 38.93)	13.4 (-3.96 to 28.28)	6.78 (-1.96 to 14.76)	-49.43 (-57.24 to - 42.58)	1.07 (-0.3 to 2.43)	0.67 (-0.17 to 1.53)	-37.63 (-47.21 to - 28.59)	12.33 (-3.64 to 26.07)	6.11 (-1.77 to 13.52)	-50.46 (-58.06 to - 43.49)
**Diet high in sodium**	0.38 (0 to 1.39)	0.49 (0.01 to 1.54)	29.67 (2.49 to 1612.77)	9.46 (0.03 to 34.07)	12.56 (0.41 to 36.96)	32.73 (1.23 to 1886.02)	0.58 (0 to 2.27)	0.97 (0.03 to 3.01)	66.85 (30.23 to 1950.35)	8.88 (0.02 to 32.1)	11.59 (0.38 to 34.33)	30.51 (-0.56 to 1878.82)
**All risk factors**	8.68 (7.65 to 9.63)	6.94 (5.84 to 7.96)	-20.09 (-24.87 to - 15.32)	213.09 (186.9 to 234.13)	162.79 (137.91 to 186.29)	-23.6 (-27.98 to - 19.16)	13.41 (9.48 to 17.66)	13.35 (9.35 to 17.66)	-0.47 (-7.47 to 6.88)	199.68 (176.03 to 220.5)	149.45 (127.37 to 170.58)	-25.16 (-29.52 to - 20.67)
**Environmental/occupational risks**	2.23 (1.46 to 3.05)	1.32 (0.9 to 1.77)	-41.09 (-57.37 to - 21.45)	54.08 (34.83 to 73.72)	29.02 (19.84 to 38.85)	-46.34 (-61.22 to - 27.71)	2.38 (1.05 to 4.12)	1.15 (0.41 to 2.1)	-51.64 (-75.56 to - 21.72)	51.69 (33.74 to 70.24)	27.87 (19.41 to 37.05)	-46.09 (-60.83 to - 27.76)
**Behavioral risks**	2.89 (1.21 to 4.36)	2.25 (0.7 to 3.73)	-21.91 (-43.89 to - 10.03)	84.99 (36.36 to 126.61)	62.12 (17.63 to 99.42)	-26.91 (-52 to - 16.95)	5.58 (1.91 to 8.97)	5.14 (0.94 to 9.35)	-7.9 (-51.28 to 7.25)	79.41 (34.39 to 117.48)	56.97 (16.62 to 91.84)	-28.25 (-52.29 to - 18.54)
**Non-optimal temperature**	0.86 (0.63 to 1.07)	0.74 (0.55 to 0.93)	-14.44 (-20.17 to - 8.2)	20.16 (14.82 to 24.85)	15.97 (12.01 to 20)	-20.78 (-26.31 to - 14.71)	N/A	N/A	N/A	20.16 (14.82 to 24.85)	15.97 (12.01 to 20)	-20.78 (-26.31 to - 14.71)
**High temperature**	0.03 (-0.07 to 0.18)	0.05 (-0.06 to 0.19)	33.76 (-153.11 to 190.89)	0.84 (-1.68 to 4.18)	1.05 (-1.28 to 4.19)	24.76 (-155.51 to 170.46)	N/A	N/A	N/A	0.84 (-1.68 to 4.18)	1.05 (-1.28 to 4.19)	24.76 (-155.51 to 170.46)

	**Low temperature**	0.83 (0.7 to 0.96)	0.7 (0.59 to 0.8)	-16.36 (-21.64 to - 10.87)	19.37 (16.28 to 22.28)	14.98 (12.9 to 17.06)	-22.65 (-27.19 to - 17.59)	N/A	N/A	N/A	19.37 (16.28 to 22.28)	14.98 (12.9 to 17.06)	-22.65 (-27.19 to - 17.59)
	**Kidney dysfunction**	1.1 (0.75 to 1.44)	1.04 (0.7 to 1.37)	-5.7 (-11.59 to - 1.1)	25.38 (18.91 to 32)	22.72 (16.79 to 28.61)	-10.47 (-15.19 to - 6.64)	1.61 (1.05 to 2.26)	1.87 (1.22 to 2.59)	16.3 (9.98 to 23.42)	23.77 (17.67 to 29.9)	20.85 (15.46 to 26.36)	-12.28 (-17 to - 8.23)
	**Particulate matter pollution**	1.08 (0.43 to 1.9)	0.32 (0.16 to 0.52)	-70.43 (-87.28 to - 33.61)	26.51 (10.69 to 45.94)	7.48 (3.72 to 12.06)	-71.78 (-87.92 to - 36.58)	1.75 (0.65 to 3.31)	0.64 (0.29 to 1.12)	-63.53 (-84.39 to - 21.25)	24.76 (9.9 to 43.21)	6.84 (3.4 to 11.05)	-72.36 (-88.16 to - 37.9)
**Subarachnoid hemorrhage**	**Air pollution**	0.36 (0.14 to 0.63)	0.1 (0.05 to 0.17)	-70.86 (-87.49 to - 35.11)	12.12 (4.86 to 21.01)	3.1 (1.53 to 4.98)	-74.45 (-89.09 to - 43.26)	1.16 (0.41 to 2.21)	0.41 (0.18 to 0.72)	-64.84 (-84.99 to - 21.73)	10.97 (4.39 to 19.09)	2.69 (1.35 to 4.37)	-75.47 (-89.45 to - 45.47)
	**Ambient particulate matter pollution**	0.36 (0.14 to 0.63)	0.1 (0.05 to 0.17)	-70.86 (-87.49 to - 35.09)	12.12 (4.86 to 21.01)	3.1 (1.53 to 4.98)	-74.45 (-89.08 to - 43.24)	1.16 (0.41 to 2.21)	0.41 (0.18 to 0.72)	-64.83 (-84.98 to - 21.65)	10.96 (4.39 to 19.09)	2.69 (1.35 to 4.36)	-75.46 (-89.44 to - 45.45)
	**Household air pollution from solid fuels**	0 (0 to 0)	0 (0 to 0)	-83.43 (-99.98 to - 87.4)	0.01 (0 to 0.06)	0 (0 to 0.01)	-84.96 (-99.98 to - 88.57)	0 (0 to 0.01)	0 (0 to 0)	-80.14 (-99.98 to - 84.69)	0.01 (0 to 0.05)	0 (0 to 0.01)	-85.46 (-99.98 to - 89.01)
	**Other environmental risks**	0.14 (-0.02 to 0.33)	0.09 (-0.01 to 0.22)	-33.53 (-38.88 to - 29.08)	4.68 (-0.58 to 10.96)	2.42 (-0.3 to 5.67)	-48.21 (-51.66 to - 44.2)	0.44 (-0.06 to 1.02)	0.3 (-0.04 to 0.72)	-30.8 (-36.4 to - 23.96)	4.24 (-0.52 to 9.99)	2.12 (-0.26 to 4.95)	-50.02 (-53.68 to - 45.82)
	**Lead exposure**	0.14 (-0.02 to 0.33)	0.09 (-0.01 to 0.22)	-33.53 (-38.88 to - 29.08)	4.68 (-0.58 to 10.96)	2.42 (-0.3 to 5.67)	-48.21 (-51.66 to - 44.2)	0.44 (-0.06 to 1.02)	0.3 (-0.04 to 0.72)	-30.8 (-36.4 to - 23.96)	4.24 (-0.52 to 9.99)	2.12 (-0.26 to 4.95)	-50.02 (-53.68 to - 45.82)

**Tobacco**	0.96 (0.83 to 1.1)	0.51 (0.42 to 0.61)	-46.58 (-50.61 to -42.3)	38.25 (32.82 to 44.09)	19.51 (16.32 to 23.09)	-48.99 (-52.49 to -45.53)	3.39 (2.37 to 4.62)	2.52 (1.73 to 3.42)	-25.61 (-31.46 to -18.82)	34.86 (30.06 to 40.08)	16.99 (14.16 to 20.01)	-51.26 (-54.71 to -47.49)
**Smoking**	0.86 (0.75 to 0.98)	0.46 (0.39 to 0.54)	-46.88 (-51.26 to -42.43)	34.53 (30.04 to 39.39)	17.49 (14.83 to 20.57)	-49.34 (-52.9 to -45.77)	3.05 (2.14 to 4.11)	2.25 (1.54 to 3.01)	-26.18 (-32.28 to -18.99)	31.49 (27.6 to 35.7)	15.25 (12.79 to 17.79)	-51.58 (-55.33 to -47.68)
**Secondhand smoke**	0.13 (0.09 to 0.18)	0.07 (0.04 to 0.09)	-50.62 (-54.41 to -46.54)	5.49 (3.69 to 7.37)	2.62 (1.77 to 3.61)	-52.38 (-55.98 to -48.37)	0.5 (0.3 to 0.74)	0.35 (0.21 to 0.53)	-29.61 (-36.19 to -22.2)	5 (3.39 to 6.72)	2.27 (1.51 to 3.11)	-54.64 (-58.23 to -50.82)
**Metabolic risks**	2.06 (1.55 to 2.5)	1.64 (1.16 to 2.05)	-20.31 (-26.59 to -14.45)	64.51 (47.72 to 79.24)	46.86 (32.31 to 58.61)	-27.37 (-33.56 to -21.32)	6.3 (4.09 to 8.99)	6.12 (3.82 to 8.67)	-2.86 (-13.72 to 8.48)	58.21 (43.26 to 71.66)	40.74 (28.15 to 50.82)	-30.02 (-35.95 to -23.97)
**High systolic blood pressure**	2 (1.51 to 2.43)	1.57 (1.11 to 1.98)	-21.41 (-27.87 to -15.35)	61.77 (45.42 to 76.83)	43.77 (30.1 to 55.14)	-29.14 (-35.27 to -22.86)	6.04 (3.95 to 8.68)	5.67 (3.58 to 8.1)	-6.16 (-16.61 to 5.5)	55.73 (40.72 to 68.75)	38.1 (26.37 to 47.85)	-31.63 (-37.61 to -25.58)
**High body-mass index**	0.29 (-0.01 to 0.64)	0.31 (-0.01 to 0.63)	6.59 (-8.73 to 39.66)	11.92 (-0.36 to 26.71)	12.3 (-0.55 to 25.09)	3.23 (-12.05 to 41.41)	1.09 (-0.03 to 2.5)	1.69 (-0.06 to 3.64)	54.49 (31.41 to 114.44)	10.83 (-0.33 to 24.06)	10.62 (-0.47 to 21.88)	-1.94 (-16.26 to 31.57)
**Dietary risks**	-0.01 (-0.99 to 0.75)	0.07 (-0.61 to 0.63)	-852.37 (-189.79 to 265.02)	-2.01 (-41.91 to 30.33)	0.83 (-26.47 to 22.45)	-141.44 (-168.51 to 123.24)	-0.17 (-3.98 to 2.74)	0.02 (-3.78 to 2.95)	-109.76 (-110.86 to 163.43)	-1.84 (-38.57 to 27.75)	0.82 (-22.87 to 19.32)	-144.29 (-168.43 to 120.78)

**Diet low in fruits**	0.29 (-0.02 to 0.54)	0.2 (-0.01 to 0.38)	-31.08 (-38.12 to -26.37)	11.53 (-0.91 to 21.02)	7.52 (-0.56 to 13.75)	-34.78 (-39.86 to -30.89)	1.07 (-0.09 to 2.21)	1.03 (-0.08 to 2.08)	-3.41 (-10.8 to 5.38)	10.46 (-0.82 to 19.11)	6.48 (-0.48 to 11.98)	-37.99 (-43.19 to -33.98)
**Diet low in vegetables**	0 (0 to 0)	0.01 (0 to 0.02)	1620.37 (1093.31 to 2416.97)	0.02 (0 to 0.04)	0.42 (-0.04 to 0.82)	2011.23 (1359.78 to 2981.93)	0 (0 to 0)	0.05 (-0.01 to 0.11)	2744.55 (1874.54 to 4043.14)	0.02 (0 to 0.04)	0.37 (-0.04 to 0.71)	1938.16 (1301.94 to 2912.95)
**Diet high in red meat**	-0.51 (-2.06 to 0.68)	-0.35 (-1.48 to 0.48)	-30.96 (-37.8 to 21040.19)	-21.67 (-89.14 to 27.9)	-14.36 (-59.97 to 18.8)	-33.72 (-39.74 to 26572.52)	-1.99 (-8.42 to 2.46)	-1.99 (-8.35 to 2.5)	0.11 (-10.84 to 43176.87)	-19.68 (-79.87 to 25.29)	-12.37 (-52.2 to 16.24)	-37.14 (-43.07 to 25111.4)
**Diet low in fiber**	0.19 (-0.06 to 0.41)	0.09 (-0.03 to 0.21)	-52.11 (-60.25 to -44.64)	7.97 (-2.43 to 16.25)	3.46 (-1.02 to 7.57)	-56.58 (-63.43 to -50.65)	0.75 (-0.22 to 1.69)	0.48 (-0.14 to 1.13)	-35.5 (-45.62 to -25.22)	7.23 (-2.21 to 14.8)	2.98 (-0.87 to 6.58)	-58.75 (-65.37 to -52.76)
**Diet high in sodium**	0.13 (0 to 0.45)	0.15 (0 to 0.48)	20.35 (-2.25 to 1215.05)	4.19 (0.01 to 15.13)	4.76 (0.15 to 14.72)	13.58 (-10.73 to 1211.92)	0.4 (0 to 1.47)	0.57 (0.01 to 1.85)	44.8 (16.13 to 1406.18)	3.8 (0.01 to 13.64)	4.19 (0.13 to 12.75)	10.33 (-13.12 to 1206.46)
**All risk factors**	2.67 (2.25 to 3.04)	2 (1.56 to 2.39)	-25.05 (-31.29 to -19.42)	88.73 (71.73 to 101.08)	59.24 (43.77 to 70.73)	-33.24 (-39.41 to -28.25)	8.21 (5.52 to 11.05)	7.36 (4.7 to 10.03)	-10.26 (-22.39 to -0.09)	80.53 (65.59 to 92.02)	51.88 (38.38 to 62.4)	-35.58 (-41.04 to -30.63)
**Environmental/occupational risks**	0.74 (0.49 to 1.01)	0.43 (0.3 to 0.57)	-42.39 (-58.25 to -23.36)	24.44 (15.77 to 33.4)	11.67 (8.16 to 15.44)	-52.25 (-65.67 to -35.36)	1.55 (0.7 to 2.68)	0.7 (0.26 to 1.26)	-54.87 (-76.88 to -24.7)	22.89 (15 to 30.96)	10.97 (7.87 to 14.34)	-52.07 (-64.87 to -35.83)
**Behavioral risks**	0.96 (0.29 to 1.51)	0.57 (0.02 to 1.06)	-40.81 (-82.9 to -27.98)	37.18 (10.08 to 58.39)	20.27 (-0.62 to 37.62)	-45.49 (-86.45 to -34.21)	3.31 (0.7 to 5.68)	2.55 (-0.32 to 5.02)	-22.94 (-91.54 to -3.8)	33.88 (9.31 to 53.14)	17.72 (-0.3 to 32.75)	-47.7 (-85.63 to -36.9)

**Non-optimal temperature**	0.29 (0.22 to 0.36)	0.25 (0.18 to 0.31)	-15.84 (-21.85 to -9.11)	9.26 (6.81 to 11.41)	6.59 (5.01 to 8.34)	-28.78 (-33.85 to -23.36)	N/A	N/A	N/A	9.26 (6.81 to 11.41)	6.59 (5.01 to 8.34)	-28.78 (-33.85 to -23.36)
**High temperature**	0.01 (-0.02 to 0.06)	0.02 (-0.02 to 0.06)	32.42 (-155.1 to 185.28)	0.39 (-0.76 to 1.92)	0.44 (-0.53 to 1.75)	13.59 (-151.19 to 141.19)	N/A	N/A	N/A	0.39 (-0.76 to 1.92)	0.44 (-0.53 to 1.75)	13.59 (-151.19 to 141.19)
**Low temperature**	0.28 (0.24 to 0.32)	0.23 (0.2 to 0.27)	-17.77 (-23.54 to -12.12)	8.89 (7.5 to 10.22)	6.18 (5.32 to 6.99)	-30.53 (-34.85 to -25.83)	N/A	N/A	N/A	8.89 (7.5 to 10.22)	6.18 (5.32 to 6.99)	-30.53 (-34.85 to -25.83)
**Particulate matter pollution**	0.36 (0.14 to 0.63)	0.1 (0.05 to 0.17)	-70.86 (-87.49 to -35.11)	12.12 (4.86 to 21.01)	3.1 (1.53 to 4.98)	-74.45 (-89.09 to -43.26)	1.16 (0.41 to 2.21)	0.41 (0.18 to 0.72)	-64.84 (-84.99 to -21.73)	10.97 (4.39 to 19.09)	2.69 (1.35 to 4.37)	-75.47 (-89.45 to -45.47)

2.10 Table S6: Rate and percentage change of mortality, DALYs, YLDs, and YLLs for stroke of all age over time by risk factor, both sexes, US, 1990-2021

Disease	Risk Factor	Death			DALYs			YLDs			YLLs		
		1990	2021	change, %	1990	2021	change, %	1990	2021	change, %	1990	2021	change,[illegible]
Stroke	**Air pollution**	5.59 (2.19 to 9.91)	1.88 (0.94 to 3.1)	-66.32 (-85.52 to -23.98)	114.51 (45.56 to 196.91)	38.09 (18.78 to 61.47)	-66.73 (-85.82 to -25.69)	19.38 (6.85 to 36.22)	8.38 (3.85 to 14.55)	-56.75 (-81.92 to -4.65)	95.13 (37.6 to 167.47)	29.71 (14.79 to 48.3)	-68.77 (-86.61 t[illegible] 29.67[illegible]
	**Ambient particulate matter pollution**	5.59 (2.19 to 9.91)	1.88 (0.94 to 3.1)	-66.31 (-85.52 to -23.96)	114.43 (45.53 to 196.9)	38.08 (18.78 to 61.46)	-66.73 (-85.82 to -25.67)	19.37 (6.84 to 36.23)	8.38 (3.85 to 14.56)	-56.74 (-81.93 to -4.65)	95.07 (37.58 to 167.47)	29.7 (14.76 to 48.31)	-68.76 (-86.61 t[illegible] 29.65[illegible]
	**Household air pollution from solid fuels**	0 (0 to 0.03)	0 (0 to 0)	-81.41 (-99.98 to -85.83)	0.07 (0 to 0.56)	0.01 (0 to 0.07)	-81.26 (-99.98 to -85.6)	0.01 (0 to 0.09)	0 (0 to 0.02)	-75.79 (-99.97 to -81.29)	0.06 (0 to 0.46)	0.01 (0 to 0.06)	-82.35 (-99.98 t[illegible] 86.55[illegible]
	**Other environmental risks**	2.24 (-0.29 to 5.1)	1.94 (-0.25 to 4.42)	-13.48 (-21.2 to -6.44)	46.68 (-6.12 to 108.5)	37.16 (-4.79 to 86.02)	-20.41 (-26.22 to -15.13)	7.87 (-1.06 to 18.04)	7.95 (-1.06 to 18.15)	1.06 (-6.3 to 9.19)	38.81 (-5 to 89.26)	29.2 (-3.7 to 67.36)	-24.76 (-30.8 t[illegible] 19.34[illegible]
	**Lead exposure**	2.24 (-0.29 to 5.1)	1.94 (-0.25 to 4.42)	-13.48 (-21.2 to -6.44)	46.68 (-6.12 to 108.5)	37.16 (-4.79 to 86.02)	-20.41 (-26.22 to -15.13)	7.87 (-1.06 to 18.04)	7.95 (-1.06 to 18.15)	1.06 (-6.3 to 9.19)	38.81 (-5 to 89.26)	29.2 (-3.7 to 67.36)	-24.76 (-30.8 t[illegible] 19.34[illegible]
	**Tobacco**	7.94 (6.57 to 9.68)	5.43 (4.28 to 6.89)	-31.63 (-37.83 to -24.48)	229.28 (194.56 to 272.39)	158.82 (128.95 to 194.98)	-30.73 (-35.61 to -25.68)	42.58 (30.64 to 57.12)	38.64 (27.52 to 52.24)	-9.26 (-16.05 to -2.48)	186.7 (159.06 to 218.6)	120.18 (99.54 to 145.57)	-35.63 (-40.3 t[illegible] 30.4)

**Smoking**	7.09 (5.9 to 8.59)	4.86 (3.85 to 6.18)	-31.56 (-38.51 to -23.65)	206.77 (177.05 to 243.13)	143 (117.63 to 174.99)	-30.84 (-36.04 to -25.34)	38.31 (27.74 to 51.04)	34.74 (24.77 to 46.65)	-9.32 (-16.67 to -2.31)	168.45 (145.59 to 196.02)	108.26 (89.82 to 130.35)	-35.73 (-40.84 t[illegible] 30.32[illegible]
**Secondhand smoke**	1.04 (0.7 to 1.41)	0.66 (0.44 to 0.92)	-36.55 (-41.78 to -30.51)	30.14 (20.33 to 40.93)	19.28 (12.93 to 26.45)	-36.02 (-40.6 to -31.14)	5.79 (3.57 to 8.59)	4.78 (2.97 to 6.98)	-17.44 (-24.01 to -10.45)	24.35 (16.58 to 32.64)	14.5 (9.82 to 19.68)	-40.4[illegible] (-45.02 t[illegible] 35.58[illegible]
**Alcohol use**	2.56 (0.37 to 6.84)	3.75 (0.64 to 8.22)	46.48 (3.7 to 155.41)	59.56 (10.65 to 148.78)	83.46 (15.55 to 175.58)	40.13 (8.65 to 115.26)	10.69 (0.28 to 30.36)	19.96 (0.63 to 48.84)	86.69 (47.87 to 196.9)	48.87 (9.39 to 119.9)	63.5 (14.71 to 127.04)	29.94 (-3.63 t[illegible] 97.61[illegible]
**Metabolic risks**	43.23 (35.58 to 49.13)	40.15 (31.96 to 47.01)	-7.12 (-13.89 to -1.66)	858.52 (719.69 to 975.78)	798.26 (655.63 to 926.6)	-7.02 (-11.84 to -2.86)	155.82 (112.09 to 204.69)	190.82 (136.95 to 249.21)	22.46 (15.15 to 29.92)	702.7 (579.43 to 796.45)	607.45 (491.62 to 704.33)	-13.5[illegible] (-18.31 t[illegible] 8.93)
**High fasting plasma glucose**	7.64 (5.99 to 9.46)	10.13 (7.83 to 12.51)	32.56 (20.78 to 45.49)	142 (110.49 to 173.88)	192.34 (151.7 to 233.49)	35.45 (25.58 to 46.71)	27.87 (18.41 to 38.21)	53.01 (35.94 to 70.6)	90.23 (73.45 to 108.6)	114.14 (89.03 to 139.81)	139.33 (112.49 to 169.49)	22.07 (11.88 [illegible] 32.83[illegible]
**High systolic blood pressure**	34.85 (26.23 to 42.31)	30.3 (21.19 to 38.88)	-13.07 (-22.51 to -4.88)	684.97 (513.48 to 828.48)	589.51 (427.43 to 746.57)	-13.94 (-21.03 to -7.33)	113.54 (77.4 to 155.8)	127.72 (84.4 to 175.47)	12.49 (1.51 to 23.5)	571.43 (429.4 to 689.19)	461.79 (329.1 to 586.49)	-19.1[illegible] (-26.05 t[illegible] 12.85[illegible]
**High body-mass index**	3.03 (0.24 to 6.53)	4.17 (0.28 to 8.57)	37.98 (17.42 to 55.48)	86.36 (5.67 to 181.51)	119.45 (7.7 to 234.66)	38.32 (19.52 to 57.98)	19.3 (2.33 to 39.15)	33.76 (4.11 to 66.09)	74.95 (53.01 to 98.03)	67.06 (3.81 to 145.12)	85.69 (3.61 to 173.07)	27.78 (9.14 t[illegible] 45.95[illegible]

**Dietary risks**	3.95 (0.5 to 8.99)	4.1 (0.25 to 9.46)	3.87 (-47.82 to 36.23)	89.44 (3.38 to 198.23)	105.89 (14.36 to 222.93)	18.4 (-7.6 to 97.67)	27.69 (4.15 to 51.16)	39.67 (9.24 to 71.86)	43.28 (24.56 to 82.03)	61.75 (-27.35 to 155.81)	66.22 (-22.28 to 165.27)	7.24 (-59.7 [illegible] 128.13[illegible]
**Diet low in fruits**	1.51 (0.1 to 2.84)	1.41 (0.06 to 2.7)	-6.35 (-24.31 to 4.12)	47.54 (4.08 to 84.24)	41.8 (3.29 to 75.18)	-12.07 (-20.5 to -5.97)	7.19 (2.36 to 12.52)	7.79 (2.38 to 13.5)	8.25 (-0.71 to 16.18)	40.35 (1.65 to 73.71)	34.02 (0.7 to 63.35)	-15.69[illegible] (-27.88 [illegible] 8.39)
**Diet low in vegetables**	0.25 (-0.11 to 0.58)	0.33 (-0.1 to 0.75)	32.51 (-44.55 to 126.21)	6.84 (1.7 to 11.8)	10.16 (3.59 to 17.48)	48.46 (16.12 to 118.31)	2.33 (1.25 to 3.85)	3.93 (2.01 to 6.32)	68.57 (47.64 to 92.25)	4.51 (0.43 to 8.49)	6.22 (1.28 to 11.99)	38.05[illegible] (-28.24 [illegible] 127.87[illegible]
**Diet low in whole grains**	0.75 (-0.73 to 2.82)	0.62 (-0.69 to 2.42)	-17.77 (-51.47 to 37.74)	23.13 (-22.23 to 70.22)	22.44 (-21.76 to 68.28)	-2.97 (-9.36 to 5.81)	8.58 (-8.65 to 23.11)	10.84 (-10.68 to 29.59)	26.39 (14.7 to 35.7)	14.55 (-13.84 to 46.01)	11.59 (-11.1 to 37.57)	-20.29[illegible] (-36.59 [illegible] 13.26[illegible]
**Diet high in red meat**	-1.69 (-7.24 to 2.5)	-1.76 (-7.43 to 2.7)	4.24 (-57.63 to 48.53)	-54.84 (-243.34 to 79.85)	-49.74 (-221.06 to 74.28)	-9.31 (-42.67 to 13.1)	-0.72 (-12.31 to 7.51)	-1.25 (-15.01 to 8.16)	72.87 (-255.41 to 242.26)	-54.12 (-229.56 to 75.96)	-48.48 (-210.47 to 69.57)	-10.41[illegible] (-43.94 [illegible] 13.94[illegible]
**Diet high in processed meat**	1.02 (0.23 to 1.79)	0.85 (0.21 to 1.45)	-16.96 (-33.27 to 4.83)	21.16 (4.66 to 37.19)	21.16 (4.89 to 37.27)	0 (-16.37 to 21.02)	5.36 (1.13 to 10.1)	8.33 (1.89 to 15.29)	55.31 (29.63 to 85.88)	15.8 (3.57 to 27.46)	12.83 (3.18 to 21.89)	-18.78[illegible] (-33.26 [illegible] 0.73)
**Diet high in sugar-sweetened beverages**	0.22 (0.1 to 0.36)	0.2 (0.09 to 0.33)	-10.23 (-36.63 to 25.12)	4.61 (2.09 to 7.36)	5.05 (2.44 to 7.87)	9.68 (-16.11 to 39.11)	1.21 (0.53 to 2.1)	2.06 (0.96 to 3.4)	70.75 (35.37 to 112.5)	3.4 (1.57 to 5.34)	2.99 (1.46 to 4.71)	-12.01[illegible] (-34.29 [illegible] 15.23[illegible]

**Diet low in fiber**	1.2 (-0.22 to 2.82)	0.77 (-0.15 to 1.8)	-35.87 (-48 to -22.56)	37.73 (-7.83 to 79.18)	21.9 (-4.36 to 48.99)	-41.94 (-51.63 to -32.87)	7.23 (-0.93 to 15.1)	4.95 (-0.59 to 10.68)	-31.54 (-44.64 to -19.79)	30.49 (-6.78 to 65.32)	16.95 (-3.8 to 38.38)	-44.4[illegible] (-54.41 [illegible] 35.22[illegible]
**Diet low in polyunsaturated fatty acids**	0 (0 to 0.01)	0 (0 to 0.01)	-34.65 (-43.32 to -26.65)	0.07 (0.02 to 0.15)	0.05 (0.01 to 0.1)	-33.68 (-40.49 to -26.89)	0.02 (0.01 to 0.04)	0.02 (0 to 0.04)	-12.87 (-21.96 to -3.73)	0.05 (0.01 to 0.11)	0.03 (0.01 to 0.07)	-41.6[illegible] (-48.39 [illegible] 35.46[illegible]
**Diet high in sodium**	1.84 (0 to 6.97)	2.43 (0.04 to 8.17)	32.13 (7.08 to 1254.83)	39.06 (0.09 to 143.02)	56.81 (1.35 to 179.51)	45.45 (15.08 to 1668.68)	6.58 (0.02 to 25.62)	12.66 (0.29 to 40.39)	92.44 (53.15 to 2099.71)	32.48 (0.07 to 119.7)	44.15 (1.05 to 140.67)	35.9[illegible] (8.28 [illegible] 1579.6[illegible]
**Low physical activity**	1.19 (-0.9 to 3.46)	1.03 (-1.13 to 3.18)	-13.39 (-117.93 to 91.76)	26.74 (-1.53 to 60.11)	26.04 (-2.49 to 59.63)	-2.61 (-54.82 to 28.77)	7.54 (2.29 to 14.55)	10.43 (3.61 to 20)	38.21 (8.89 to 87.46)	19.2 (-4.27 to 46.35)	15.62 (-6.97 to 39.51)	-18.6[illegible] (-80.73 [illegible] 22.91[illegible]
**All risk factors**	48.07 (41.48 to 53.1)	44.66 (36.94 to 50.89)	-7.1 (-12.89 to -2.18)	980.65 (873.8 to 1073.67)	903.42 (782.09 to 1014.76)	-7.88 (-11.91 to -4)	172.4 (124.02 to 219.52)	209.76 (153.17 to 268.39)	21.67 (14.95 to 28.76)	808.25 (714.86 to 883.72)	693.65 (588.94 to 783.45)	-14.1[illegible] (-18.45 [illegible] 9.89)
**Environmental/occupational risks**	11.54 (7.46 to 15.76)	7.85 (5.19 to 10.62)	-32 (-50.21 to -9.4)	223.9 (142.06 to 309.04)	138.68 (89.7 to 191.31)	-38.06 (-55.71 to -15.75)	26.48 (11.55 to 46.12)	16.07 (5.55 to 29.03)	-39.31 (-70.27 to -3.03)	197.42 (128.91 to 268.61)	122.61 (83.75 to 164.77)	-37.9[illegible] (-54.87 [illegible] 17.51[illegible]
**Behavioral risks**	14.21 (9.36 to 19.33)	12.95 (7.63 to 19.02)	-8.86 (-23.29 to 4.51)	361.06 (268.96 to 467.89)	328.52 (225.62 to 446.17)	-9.01 (-20.67 to 0.84)	74.28 (48.6 to 102.91)	90.87 (57.56 to 128.53)	22.33 (8.41 to 36.63)	286.78 (211.16 to 375.01)	237.65 (148.67 to 330.5)	-17.1[illegible] (-30.24 [illegible] 6.41)

	**Non-optimal temperature**	4.53 (3.26 to 5.65)	4.38 (3.2 to 5.52)	-3.34 (-10.15 to 3.63)	77.52 (56.91 to 96.7)	69.1 (51.37 to 86.46)	-10.86 (-16.98 to - 4.45)	N/A	N/A	N/A	77.52 (56.91 to 96.7)	69.1 (51.37 to 86.46)	-10.8[illegible] (-16.98 [illegible] 4.45)
	**High temperature**	0.18 (-0.38 to 0.87)	0.28 (-0.35 to 1.13)	59.11 (-165.86 to 257.7)	3.1 (-6.45 to 15.66)	4.53 (-5.56 to 18.24)	45.96 (-166.51 to 226.37)	N/A	N/A	N/A	3.1 (-6.45 to 15.66)	4.53 (-5.56 to 18.24)	45.96 (-166.51 [illegible] 226.37[illegible]
	**Low temperature**	4.37 (3.58 to 5.09)	4.12 (3.34 to 4.74)	-5.72 (-12.39 to 0.73)	74.6 (61.96 to 85.94)	64.82 (54.39 to 74.29)	-13.11 (-18.61 to - 7.46)	N/A	N/A	N/A	74.6 (61.96 to 85.94)	64.82 (54.39 to 74.29)	-13.1[illegible] (-18.61 [illegible] 7.46)
	**Kidney dysfunction**	5.57 (3.34 to 7.92)	5.96 (3.58 to 8.5)	6.91 (-0.05 to 13.25)	103.95 (69.31 to 140.17)	111.22 (75.37 to 148.67)	6.99 (2.29 to 11.52)	18.08 (11.26 to 26.13)	25.27 (16.42 to 35.97)	39.77 (31.74 to 48.92)	85.87 (55.77 to 115.7)	85.95 (56.93 to 116.21)	0.09 (-5.37 [illegible] 5.36)
	**High LDL cholesterol**	11.85 (3.67 to 20.17)	8.11 (2.28 to 14.59)	-31.61 (-37.93 to - 26.71)	243.02 (85.82 to 393.59)	180.19 (60.6 to 302.81)	-25.85 (-31.31 to - 21.81)	65.95 (25.11 to 107.68)	67.89 (24.08 to 112.62)	2.94 (-5.88 to 10.75)	177.07 (59.4 to 291)	112.3 (35.91 to 194.86)	-36.5[illegible] (-41.73 [illegible] 32.65[illegible]
	**Particulate matter pollution**	5.59 (2.19 to 9.91)	1.88 (0.94 to 3.1)	-66.32 (-85.52 to - 23.98)	114.51 (45.56 to 196.91)	38.09 (18.78 to 61.47)	-66.73 (-85.82 to - 25.69)	19.38 (6.85 to 36.22)	8.38 (3.85 to 14.55)	-56.75 (-81.92 to - 4.65)	95.13 (37.6 to 167.47)	29.71 (14.79 to 48.3)	-68.7[illegible] (-86.61 [illegible] 29.67[illegible]
**Ischemic stroke**	**Air pollution**	3.79 (1.47 to 6.71)	1.13 (0.57 to 1.88)	-70.21 (-87.2 to - 32.35)	68.7 (27.01 to 119.59)	21.14 (10.3 to 34.45)	-69.23 (-86.94 to - 31.18)	15.93 (5.61 to 29.75)	6.79 (3.1 to 11.85)	-57.39 (-82.21 to - 5.83)	52.78 (20.69 to 93.43)	14.36 (7.14 to 23.62)	-72.8[illegible] (-88.33 [illegible] 38.33[illegible]

**Ambient particulate matter pollution**	3.79 (1.47 to 6.71)	1.13 (0.57 to 1.88)	-70.2 (-87.2 to - 32.33)	68.66 (27 to 119.59)	21.13 (10.3 to 34.46)	-69.22 (-86.95 to - 31.18)	15.92 (5.61 to 29.77)	6.78 (3.1 to 11.86)	-57.38 (-82.2 to - 5.84)	52.74 (20.68 to 93.43)	14.35 (7.14 to 23.63)	-72.7[illegible] (-88.33 [illegible] 38.32[illegible]
**Household air pollution from solid fuels**	0 (0 to 0.02)	0 (0 to 0)	-83.65 (-99.98 to - 87.58)	0.04 (0 to 0.34)	0.01 (0 to 0.04)	-82.83 (-99.98 to - 86.84)	0.01 (0 to 0.07)	0 (0 to 0.01)	-76.11 (-99.97 to - 81.6)	0.03 (0 to 0.26)	0 (0 to 0.03)	-84.7[illegible] (-99.98 [illegible] 88.38[illegible]
**Other environmental risks**	1.5 (-0.2 to 3.41)	1.18 (-0.15 to 2.68)	-20.87 (-28.79 to - 14.32)	27.91 (-3.73 to 64.35)	21.44 (-2.78 to 49.25)	-23.18 (-29.21 to - 17.59)	6.52 (-0.88 to 15.1)	6.55 (-0.87 to 15.08)	0.5 (-8.12 to 9.71)	21.39 (-2.79 to 48.78)	14.88 (-1.89 to 33.93)	-30.4[illegible] (-36.88 [illegible] 24.64[illegible]
**Lead exposure**	1.5 (-0.2 to 3.41)	1.18 (-0.15 to 2.68)	-20.87 (-28.79 to - 14.32)	27.91 (-3.73 to 64.35)	21.44 (-2.78 to 49.25)	-23.18 (-29.21 to - 17.59)	6.52 (-0.88 to 15.1)	6.55 (-0.87 to 15.08)	0.5 (-8.12 to 9.71)	21.39 (-2.79 to 48.78)	14.88 (-1.89 to 33.93)	-30.4[illegible] (-36.88 [illegible] 24.64[illegible]
**Tobacco**	4.21 (3.3 to 5.34)	2.48 (1.82 to 3.3)	-40.94 (-48.34 to - 31.8)	107.43 (88.07 to 132.22)	71.49 (55.76 to 91.5)	-33.45 (-39.2 to - 27.79)	33.28 (23.53 to 44.94)	30.3 (21.4 to 41.44)	-8.95 (-16.19 to - 1.49)	74.14 (60.54 to 90.82)	41.19 (32.34 to 52.53)	-44.4[illegible] (-49.61 [illegible] 38.65[illegible]
**Smoking**	3.72 (2.92 to 4.68)	2.2 (1.63 to 2.93)	-40.8 (-49.1 to - 30.98)	96.38 (79.21 to 118.02)	64.15 (50.12 to 82.06)	-33.44 (-39.67 to - 27.38)	29.96 (21.39 to 40.38)	27.27 (19.25 to 37.15)	-8.97 (-16.69 to - 1.01)	66.42 (54.4 to 80.77)	36.88 (28.94 to 46.97)	-44.4[illegible] (-50.23 [illegible] 38.07[illegible]
**Secondhand smoke**	0.56 (0.37 to 0.78)	0.31 (0.2 to 0.44)	-44.38 (-49.77 to - 37.91)	13.78 (9.16 to 19.17)	8.63 (5.63 to 12.15)	-37.37 (-42.27 to - 31.85)	4.45 (2.72 to 6.64)	3.7 (2.29 to 5.43)	-16.75 (-24.14 to - 9.51)	9.33 (6.29 to 12.72)	4.92 (3.3 to 6.89)	-47.2[illegible] (-51.9 [illegible] 42)

**Alcohol use**	1.68 (-0.14 to 5.37)	2.3 (-0.24 to 5.95)	36.94 (-2.89 to 166.53)	35.9 (-4.04 to 110.31)	49.13 (-6.54 to 127.62)	36.87 (3.33 to 135.73)	9.29 (-1.24 to 28.14)	17.29 (-2.79 to 44.49)	86.15 (47.43 to 203.18)	26.61 (-2.75 to 81.22)	31.85 (-3.99 to 81.23)	19.67 (-11.85 [illegible] 111.92[illegible]
**Metabolic risks**	31.38 (25.94 to 35.8)	26.65 (20.83 to 31.24)	-15.06 (-21.45 to -9.8)	575.09 (482.95 to 658.05)	503.58 (414.89 to 584.08)	-12.44 (-17.1 to -8.34)	135.37 (96.16 to 178.01)	163.6 (117.33 to 213.09)	20.86 (13.2 to 28.83)	439.72 (365.79 to 495.3)	339.97 (276.73 to 393.08)	-22.6[illegible] (-27.59 [illegible] 18.58[illegible]
**High fasting plasma glucose**	6.72 (5.09 to 8.38)	8.69 (6.37 to 10.69)	29.46 (17.82 to 42.05)	121.66 (93.5 to 153.08)	162.16 (124.13 to 199.17)	33.28 (23.2 to 44.88)	26.67 (17.47 to 36.45)	50.64 (34.18 to 67.61)	89.89 (73.22 to 108.32)	94.99 (73.02 to 117.59)	111.51 (85.15 to 135.84)	17.39 (7.56 t[illegible] 28.08[illegible]
**High systolic blood pressure**	24.03 (17.98 to 29.16)	18.61 (12.81 to 23.9)	-22.55 (-32.57 to -13.84)	427.94 (320.13 to 518.09)	336.7 (239.52 to 432.5)	-21.32 (-29.35 to -14.52)	95.03 (64.76 to 130.56)	104.23 (69.34 to 144.03)	9.68 (-1.73 to 21.02)	332.91 (251.61 to 401.7)	232.46 (163.84 to 297.92)	-30.1[illegible] (-37.64 [illegible] 23.54[illegible]
**High body-mass index**	2.02 (0.29 to 4.16)	2.52 (0.36 to 5.32)	24.61 (7.33 to 38.31)	51.29 (7.5 to 102.06)	68.17 (10.15 to 129)	32.9 (16.32 to 47.06)	16.4 (2.4 to 32.92)	28.52 (4.34 to 54.7)	73.89 (52.43 to 96.59)	34.89 (5.17 to 70.42)	39.64 (5.98 to 78.87)	13.62 (-0.95 [illegible] 26.01[illegible]
**Dietary risks**	3.75 (0.44 to 7.91)	3.39 (0.42 to 7.09)	-9.53 (-30.34 to 16.54)	90.64 (4.15 to 172.39)	93.05 (10.81 to 170.76)	2.66 (-10.3 to 40.74)	28.04 (-1.67 to 52.78)	38.89 (2.7 to 72.13)	38.72 (-1.32 to 87.61)	62.6 (6.07 to 125.36)	54.16 (7.06 to 105.67)	-13.4[illegible] (-27.13 [illegible] 14.8)
**Diet low in fruits**	0.36 (-0.09 to 0.81)	0.26 (-0.12 to 0.63)	-29.44 (-76.92 to 14.76)	11.24 (4.16 to 19.05)	9.22 (3.4 to 15.57)	-17.99 (-25.33 to -9.18)	4.21 (2.32 to 6.62)	4.46 (2.42 to 7.02)	5.88 (-3.85 to 15.89)	7.03 (1.4 to 12.82)	4.76 (0.61 to 9.23)	-32.2[illegible] (-50.31 [illegible] 24.38[illegible]

**Diet low in vegetables**	0.24 (-0.11 to 0.57)	0.26 (-0.16 to 0.63)	5.11 (-57.91 to 59.53)	6.76 (1.64 to 11.67)	8.27 (1.88 to 14.15)	22.27 (8.74 to 40.93)	2.33 (1.25 to 3.84)	3.76 (1.98 to 6.03)	61.41 (43.56 to 81.16)	4.43 (0.36 to 8.36)	4.51 (-0.06 to 8.91)	1.73 (-27.24 [illegible] 22.97[illegible]
**Diet low in whole grains**	0.75 (-0.73 to 2.82)	0.62 (-0.69 to 2.42)	-17.77 (-51.47 to 37.74)	23.13 (-22.23 to 70.22)	22.44 (-21.76 to 68.28)	-2.97 (-9.36 to 5.81)	8.58 (-8.65 to 23.11)	10.84 (-10.68 to 29.59)	26.39 (14.7 to 35.7)	14.55 (-13.84 to 46.01)	11.59 (-11.1 to 37.57)	-20.2[illegible] (-36.59 [illegible] 13.26[illegible]
**Diet high in red meat**	0.15 (-0.08 to 0.58)	0.12 (-0.06 to 0.47)	-20.73 (-87.32 to 127.32)	9.47 (-5.78 to 26.28)	8.29 (-5.14 to 23.92)	-12.47 (-44.27 to 21.65)	4.73 (-3.01 to 12.59)	4.83 (-3.01 to 13.59)	2.03 (-31.06 to 31.46)	4.74 (-2.71 to 13.86)	3.47 (-1.99 to 10.61)	-26.9[illegible] (-61.03 [illegible] 14.75[illegible]
**Diet high in processed meat**	1.02 (0.23 to 1.79)	0.85 (0.21 to 1.45)	-16.96 (-33.27 to 4.83)	21.16 (4.66 to 37.19)	21.16 (4.89 to 37.27)	0 (-16.37 to 21.02)	5.36 (1.13 to 10.1)	8.33 (1.89 to 15.29)	55.31 (29.63 to 85.88)	15.8 (3.57 to 27.46)	12.83 (3.18 to 21.89)	-18.7[illegible] (-33.26 [illegible] 0.73)
**Diet high in sugar-sweetened beverages**	0.22 (0.1 to 0.36)	0.2 (0.09 to 0.33)	-10.23 (-36.63 to 25.12)	4.61 (2.09 to 7.36)	5.05 (2.44 to 7.87)	9.68 (-16.11 to 39.11)	1.21 (0.53 to 2.1)	2.06 (0.96 to 3.4)	70.75 (35.37 to 112.5)	3.4 (1.57 to 5.34)	2.99 (1.46 to 4.71)	-12.0[illegible] (-34.29 [illegible] 15.23[illegible]
**Diet low in fiber**	0.43 (-0.21 to 1.3)	0.22 (-0.15 to 0.73)	-48.58 (-105.24 to -4.27)	13.23 (-0.83 to 29.66)	7.18 (-0.39 to 16.9)	-45.71 (-61.28 to -35.98)	5.14 (-0.26 to 10.45)	3.42 (-0.12 to 7.24)	-33.54 (-46.82 to -21.43)	8.09 (-0.67 to 20.05)	3.76 (-0.57 to 9.83)	-53.4[illegible] (-84.78 [illegible] 42.59[illegible]
**Diet low in polyunsaturated fatty acids**	0 (0 to 0.01)	0 (0 to 0.01)	-34.65 (-43.32 to -26.65)	0.07 (0.02 to 0.15)	0.05 (0.01 to 0.1)	-33.68 (-40.49 to -26.89)	0.02 (0.01 to 0.04)	0.02 (0 to 0.04)	-12.87 (-21.96 to -3.73)	0.05 (0.01 to 0.11)	0.03 (0.01 to 0.07)	-41.6[illegible] (-48.39 [illegible] 35.46[illegible]

**Diet high in sodium**	1.21 (0 to 4.69)	1.31 (0.01 to 4.63)	7.99 (-12.49 to 864.33)	22.96 (0.04 to 85.85)	29.14 (0.57 to 95.08)	26.94 (2.3 to 1349.59)	5.43 (0.01 to 21.12)	10.28 (0.23 to 33.12)	89.34 (49.25 to 2139.84)	17.53 (0.03 to 66.46)	18.86 (0.32 to 62.97)	7.6 (-14.1 [illegible] 1069.5[illegible]
**Low physical activity**	1.19 (-0.9 to 3.46)	1.03 (-1.13 to 3.18)	-13.39 (-117.93 to 91.76)	26.74 (-1.53 to 60.11)	26.04 (-2.49 to 59.63)	-2.61 (-54.82 to 28.77)	7.54 (2.29 to 14.55)	10.43 (3.61 to 20)	38.21 (8.89 to 87.46)	19.2 (-4.27 to 46.35)	15.62 (-6.97 to 39.51)	-18.6[illegible] (-80.73[illegible] 22.91[illegible]
**All risk factors**	33.84 (28.61 to 37.6)	28.75 (22.85 to 33.04)	-15.02 (-20.62 to -10.76)	622.81 (544.02 to 697.79)	547.04 (462.76 to 625.44)	-12.17 (-16.59 to -8.72)	146.74 (105.61 to 189.9)	177.87 (129.26 to 227.82)	21.21 (13.89 to 28.76)	476.07 (412.73 to 522)	369.16 (305.14 to 416.23)	-22.4[illegible] (-27.08 [illegible] 18.98[illegible]
**Environmental/occupational risks**	7.82 (5.02 to 10.78)	4.73 (3.06 to 6.5)	-39.51 (-55.8 to -19.23)	131.19 (81.17 to 184.17)	73 (44.57 to 104.78)	-44.36 (-60.46 to -24.41)	21.81 (9.33 to 37.87)	13.12 (4.51 to 23.78)	-39.84 (-70.63 to -3.94)	109.38 (70.67 to 149.17)	59.88 (39.55 to 81.36)	-45.2[illegible] (-59.77 [illegible] 27.12[illegible]
**Behavioral risks**	9.61 (5.17 to 14.15)	8.19 (3.66 to 13.04)	-14.73 (-33.7 to 0.62)	221.95 (143.31 to 306.17)	203.15 (115.65 to 291.61)	-8.47 (-21.13 to 2.67)	64.14 (39.2 to 94.53)	79.71 (46.62 to 116.08)	24.27 (8.98 to 38.42)	157.81 (99.99 to 218.55)	123.44 (65.49 to 182.26)	-21.7[illegible] (-35.19 [illegible] 10.8)
**Non-optimal temperature**	3.08 (2.23 to 3.87)	2.63 (1.89 to 3.36)	-14.75 (-21.45 to -8.65)	42.99 (30.95 to 53.73)	33.33 (24.54 to 42.17)	-22.48 (-28.24 to -16.82)	N/A	N/A	N/A	42.99 (30.95 to 53.73)	33.33 (24.54 to 42.17)	-22.4[illegible] (-28.24 [illegible] 16.82[illegible]
**High temperature**	0.12 (-0.26 to 0.59)	0.17 (-0.21 to 0.69)	44.67 (-173.01 to 208.79)	1.68 (-3.58 to 8.37)	2.21 (-2.68 to 8.82)	31.8 (-164.24 to 193.79)	N/A	N/A	N/A	1.68 (-3.58 to 8.37)	2.21 (-2.68 to 8.82)	31.8 (-164.24 [illegible] 193.79[illegible]

	**Low temperature**	2.97 (2.4 to 3.49)	2.47 (1.95 to 2.88)	-16.98 (-23.45 to -11.27)	41.41 (33.91 to 48.23)	31.24 (25.35 to 36.03)	-24.57 (-29.98 to -19.59)	N/A	N/A	N/A	41.41 (33.91 to 48.23)	31.24 (25.35 to 36.03)	-24.5[illegible] (-29.98 [illegible] 19.59[illegible]
	**Kidney dysfunction**	4.15 (2.4 to 6.04)	4.03 (2.3 to 5.91)	-3.03 (-10.45 to 3.76)	72.75 (46.61 to 100.71)	72.03 (47.04 to 98.56)	-1 (-6.22 to 3.8)	16.13 (9.96 to 23.52)	22.16 (14.36 to 31.75)	37.42 (28.44 to 47.53)	56.63 (34.74 to 79.44)	49.87 (30.52 to 70.5)	-11.94 (-18.3 t[illegible] 6.34)
	**High LDL cholesterol**	11.85 (3.67 to 20.17)	8.11 (2.28 to 14.59)	-31.61 (-37.93 to -26.71)	243.02 (85.82 to 393.59)	180.19 (60.6 to 302.81)	-25.85 (-31.31 to -21.81)	65.95 (25.11 to 107.68)	67.89 (24.08 to 112.62)	2.94 (-5.88 to 10.75)	177.07 (59.4 to 291)	112.3 (35.91 to 194.86)	-36.58 (-41.73 [illegible] 32.65[illegible]
	**Particulate matter pollution**	3.79 (1.47 to 6.71)	1.13 (0.57 to 1.88)	-70.21 (-87.2 to -32.35)	68.7 (27.01 to 119.59)	21.14 (10.3 to 34.45)	-69.23 (-86.94 to -31.18)	15.93 (5.61 to 29.75)	6.79 (3.1 to 11.85)	-57.39 (-82.21 to -5.83)	52.78 (20.69 to 93.43)	14.36 (7.14 to 23.62)	-72.8[illegible] (-88.33 [illegible] 38.33[illegible]
	**Air pollution**	1.37 (0.54 to 2.43)	0.58 (0.29 to 0.94)	-58.06 (-81.94 to -5.8)	32.03 (12.89 to 55.58)	12.33 (6.1 to 19.83)	-61.52 (-83.49 to -13.56)	2.09 (0.78 to 3.95)	1 (0.46 to 1.75)	-52.33 (-79.52 to 3.49)	29.94 (11.94 to 52.36)	11.33 (5.6 to 18.3)	-62.16 (-83.76 [illegible] 14.96[illegible]
**[illegible]tracerebral [illegible]emorrhage**	**Ambient particulate matter pollution**	1.37 (0.54 to 2.43)	0.58 (0.29 to 0.94)	-58.05 (-81.94 to -5.77)	32.01 (12.88 to 55.57)	12.32 (6.1 to 19.84)	-61.51 (-83.49 to -13.54)	2.09 (0.78 to 3.95)	1 (0.46 to 1.75)	-52.32 (-79.52 to 3.56)	29.92 (11.94 to 52.36)	11.32 (5.6 to 18.26)	-62.15 (-83.76 [illegible] 14.93[illegible]
	**Household air pollution from solid fuels**	0 (0 to 0.01)	0 (0 to 0)	-76.53 (-99.97 to -82.09)	0.02 (0 to 0.15)	0 (0 to 0.02)	-78.16 (-99.98 to -83.24)	0 (0 to 0.01)	0 (0 to 0)	-73.75 (-99.97 to -79.37)	0.02 (0 to 0.14)	0 (0 to 0.02)	-78.46 (-99.98 [illegible] 83.6)

**Other environmental risks**	0.57 (-0.07 to 1.31)	0.58 (-0.07 to 1.36)	1.8 (-6.81 to 9.42)	13.49 (-1.73 to 31.04)	11.79 (-1.49 to 27.48)	-12.58 (-19.09 to -6)	0.84 (-0.11 to 1.96)	0.91 (-0.12 to 2.13)	8.69 (-0.55 to 18.97)	12.65 (-1.62 to 29.2)	10.88 (-1.37 to 25.47)	-13.99 (-20.52 t[illegible] 7.11)
**Lead exposure**	0.57 (-0.07 to 1.31)	0.58 (-0.07 to 1.36)	1.8 (-6.81 to 9.42)	13.49 (-1.73 to 31.04)	11.79 (-1.49 to 27.48)	-12.58 (-19.09 to -6)	0.84 (-0.11 to 1.96)	0.91 (-0.12 to 2.13)	8.69 (-0.55 to 18.97)	12.65 (-1.62 to 29.2)	10.88 (-1.37 to 25.47)	-13.99 (-20.52 t[illegible] 7.11)
**Tobacco**	2.66 (2.26 to 3.12)	2.18 (1.78 to 2.68)	-18.09 (-24.45 to -11.4)	79.84 (68.51 to 93.14)	61.02 (50.57 to 73.08)	-23.57 (-29.01 to -18.03)	5.5 (3.93 to 7.42)	4.98 (3.49 to 6.77)	-9.46 (-17.65 to -1.11)	74.35 (63.72 to 86.03)	56.04 (46.53 to 66.81)	-24.6[illegible] (-30.08 t[illegible] 19.08[illegible]
**Smoking**	2.41 (2.07 to 2.81)	1.97 (1.61 to 2.42)	-18.32 (-25.34 to -11.03)	72.47 (63.07 to 83.63)	55.18 (46.1 to 66.04)	-23.85 (-29.75 to -17.95)	4.94 (3.53 to 6.61)	4.46 (3.12 to 6.1)	-9.59 (-17.95 to -0.71)	67.53 (58.75 to 77.1)	50.72 (42.42 to 60.28)	-24.8[illegible] (-30.84 t[illegible] 19.01[illegible]
**Secondhand smoke**	0.33 (0.23 to 0.45)	0.25 (0.17 to 0.35)	-23.64 (-30.24 to -16.93)	10.35 (7.06 to 14)	7.28 (4.96 to 9.91)	-29.61 (-34.83 to -23.45)	0.79 (0.48 to 1.17)	0.64 (0.4 to 0.94)	-19.27 (-27.3 to -11.27)	9.56 (6.58 to 12.7)	6.64 (4.53 to 9.02)	-30.4[illegible] (-35.85 t[illegible] 24.43[illegible]
**Alcohol use**	0.88 (0.04 to 2.07)	1.44 (0.05 to 3.24)	64.81 (31.37 to 147.36)	23.66 (0.83 to 52.85)	34.32 (1.05 to 74.59)	45.08 (22.66 to 100.86)	1.41 (0.04 to 3.47)	2.68 (0.08 to 5.82)	90.27 (57.33 to 172.76)	22.25 (0.8 to 50.1)	31.65 (0.97 to 68.89)	42.22 (19.63 [illegible] 96.29[illegible]
**Metabolic risks**	9.38 (7.63 to 10.9)	10.69 (8.36 to 12.73)	13.93 (5.34 to 21.25)	209.71 (169.38 to 243.59)	223.52 (177.41 to 265.7)	6.58 (-0.39 to 12.91)	12.98 (8.83 to 17.43)	17.99 (12.42 to 24.42)	38.63 (29.11 to 48.54)	196.73 (159.26 to 228.44)	205.53 (162.86 to 243.79)	4.47 (-2.4 t[illegible] 11.09[illegible]

**High fasting plasma glucose**	0.93 (0.51 to 1.39)	1.44 (0.78 to 2.09)	55.07 (39.04 to 71.63)	20.34 (10.89 to 30.5)	30.18 (16.6 to 44.04)	48.39 (34.09 to 63.97)	1.2 (0.6 to 1.93)	2.37 (1.22 to 3.79)	97.92 (79.03 to 120.6)	19.15 (10.25 to 28.79)	27.82 (15.18 to 40.45)	45.3 (30.7 t 61.04
**High systolic blood pressure**	8.41 (6.37 to 10.14)	8.97 (6.39 to 11.39)	6.58 (-3.33 to 15.4)	186.32 (139.19 to 224.78)	185.46 (130.41 to 233.57)	-0.46 (-9.14 to 7.43)	11.33 (7.58 to 15.9)	14.81 (9.54 to 21.03)	30.7 (19.81 to 42.12)	174.99 (131.43 to 210.43)	170.65 (119.67 to 215.78)	-2.48 (-11.14 5.51)
**High body-mass index**	0.69 (-0.02 to 1.61)	1.21 (-0.05 to 2.58)	76.03 (47.74 to 145.26)	22.04 (-0.6 to 50.36)	35.11 (-1.43 to 72.89)	59.34 (33.16 to 119.23)	1.68 (-0.04 to 3.92)	3.07 (-0.12 to 6.55)	82.78 (52.47 to 151.57)	20.36 (-0.56 to 46.73)	32.04 (-1.31 to 65.98)	57.4 (31.43 117.45
**Dietary risks**	0.2 (-2.28 to 2.33)	0.58 (-2.14 to 3.25)	185.38 (-620.08 to 326.78)	0.89 (-78.27 to 62.78)	10.64 (-64.96 to 76.98)	1096.94 (-338.07 to 335.92)	-0.18 (-6.45 to 4.51)	0.61 (-6.04 to 6.48)	-434.67 (-247.26 to 336.24)	1.07 (-72.87 to 58.88)	10.03 (-59.36 to 70.91)	835.9 (-337.72 337.21
**Diet low in fruits**	0.82 (-0.05 to 1.58)	0.86 (-0.05 to 1.69)	4.85 (-5.15 to 11.81)	23.46 (-1.68 to 44.18)	22.56 (-1.49 to 43.09)	-3.81 (-12.41 to 2.28)	1.76 (-0.14 to 3.61)	1.98 (-0.15 to 4.08)	12.22 (1.26 to 21.13)	21.7 (-1.54 to 41.34)	20.59 (-1.35 to 39.65)	-5.11 (-13.76 1.08)
**Diet low in vegetables**	0 (0 to 0.01)	0.05 (-0.01 to 0.12)	1602.99 (1000.22 to 2590.98)	0.06 (-0.01 to 0.12)	1.34 (-0.12 to 2.81)	2191.63 (1475.19 to 3260.82)	0 (0 to 0.01)	0.11 (-0.01 to 0.23)	2423.21 (1643.36 to 3542.78)	0.05 (-0.01 to 0.12)	1.23 (-0.11 to 2.6)	2172.5 (1443.64 3266.3
**Diet high in red meat**	-1.27 (-5.13 to 1.8)	-1.37 (-5.59 to 2.02)	8.29 (-3.19 to 32998.77)	-40.28 (-163.94 to 54.49)	-39.52 (-163.67 to 54.19)	-1.89 (-10.6 to 39829.8)	-3.2 (-13.45 to 3.92)	-3.56 (-14.84 to 4.53)	11.25 (1.08 to 42148.26)	-37.08 (-150.54 to 50.52)	-35.95 (-150.1 to 49.83)	-3.03 (-11.9 39627.6

**Diet low in fiber**	0.54 (-0.16 to 1.19)	0.4 (-0.12 to 0.94)	-25.65 (-37.53 to - 14.38)	15.58 (-4.59 to 33.06)	10.12 (-2.89 to 22.86)	-35.01 (-45.49 to - 25.46)	1.24 (-0.34 to 2.83)	0.91 (-0.23 to 2.09)	-26.54 (-38.93 to - 14.68)	14.33 (-4.22 to 30.43)	9.21 (-2.63 to 20.71)	-35.74 (-46.36 [illegible] 26.13[illegible]
**Diet high in sodium**	0.48 (0 to 1.78)	0.87 (0.02 to 2.76)	81.11 (43.25 to 2283.84)	11.38 (0.03 to 41.33)	20.53 (0.6 to 61.84)	80.42 (39.95 to 2389.71)	0.69 (0 to 2.7)	1.52 (0.04 to 4.77)	121.32 (74.58 to 2334.17)	10.69 (0.03 to 38.97)	19.01 (0.56 to 57.62)	77.79 (37.83 [illegible] 2397.9[illegible]
**All risk factors**	11.07 (9.71 to 12.28)	12.53 (10.5 to 14.44)	13.2 (6.15 to 20.42)	257.33 (225.46 to 282.54)	268.28 (227.96 to 306.38)	4.26 (-1.86 to 9.89)	16.03 (11.35 to 21.04)	21.01 (14.77 to 27.71)	31.02 (21.99 to 40.49)	241.29 (212.23 to 266.83)	247.28 (210.42 to 282.24)	2.48 (-3.59 [illegible] 8.44)
**Environmental/occupational risks**	2.84 (1.85 to 3.89)	2.39 (1.63 to 3.22)	-15.95 (-39.11 to 11.65)	65.15 (41.91 to 88.9)	48.27 (32.76 to 64.75)	-25.92 (-46.55 to - 0.62)	2.85 (1.26 to 4.9)	1.88 (0.64 to 3.41)	-34.11 (-67.57 to 6.55)	62.3 (40.61 to 84.74)	46.39 (31.98 to 61.96)	-25.54 (-45.99 [illegible] 0.78)
**Behavioral risks**	3.51 (1.46 to 5.38)	3.87 (1.2 to 6.44)	10.26 (-20.04 to 28.24)	98.15 (41.27 to 146.83)	97.15 (29.09 to 157.69)	-1.02 (-30.24 to 13.84)	6.41 (2.12 to 10.33)	7.64 (1.57 to 13.5)	19.16 (-20.92 to 43.74)	91.74 (39.38 to 136.52)	89.51 (27.51 to 145.6)	-2.43 (-30.77 [illegible] 12.21[illegible]
**Non-optimal temperature**	1.1 (0.8 to 1.37)	1.34 (1 to 1.67)	21.41 (13.18 to 30.55)	24.2 (17.74 to 29.87)	26.22 (19.67 to 32.79)	8.36 (0.96 to 16.28)	N/A	N/A	N/A	24.2 (17.74 to 29.87)	26.22 (19.67 to 32.79)	8.36 (0.96 [illegible] 16.28[illegible]
**High temperature**	0.04 (-0.09 to 0.22)	0.08 (-0.11 to 0.34)	88.74 (-171.9 to 310.9)	1 (-2.01 to 5)	1.7 (-2.11 to 6.83)	69.6 (-170.76 to 265.73)	N/A	N/A	N/A	1 (-2.01 to 5)	1.7 (-2.11 to 6.83)	69.6 (-170.76 [illegible] 265.73[illegible]

	**Low temperature**	1.06 (0.88 to 1.22)	1.26 (1.05 to 1.44)	18.75 (10.39 to 26.81)	23.26 (19.57 to 26.72)	24.62 (21.13 to 28.11)	5.86 (-0.78 to 12.73)	N/A	N/A	N/A	23.26 (19.57 to 26.72)	24.62 (21.13 to 28.11)	5.86 (-0.78 [illegible] 12.73[illegible]
	**Kidney dysfunction**	1.42 (0.96 to 1.88)	1.93 (1.29 to 2.6)	35.97 (26.56 to 43.45)	31.19 (22.87 to 39.58)	39.19 (28.46 to 50.13)	25.63 (18.57 to 31.22)	1.95 (1.26 to 2.76)	3.11 (2.01 to 4.31)	59.21 (48.72 to 70.48)	29.24 (21.44 to 37.07)	36.08 (26.35 to 46.33)	23.39 (16.12 [illegible] 29.26[illegible]
	**Particulate matter pollution**	1.37 (0.54 to 2.43)	0.58 (0.29 to 0.94)	-58.06 (-81.94 to -5.8)	32.03 (12.89 to 55.58)	12.33 (6.1 to 19.83)	-61.52 (-83.49 to -13.56)	2.09 (0.78 to 3.95)	1 (0.46 to 1.75)	-52.33 (-79.52 to 3.49)	29.94 (11.94 to 52.36)	11.33 (5.6 to 18.3)	-62.16 (-83.76 [illegible] 14.96[illegible]
**barachnoid emorrhage**	**Air pollution**	0.43 (0.17 to 0.75)	0.18 (0.09 to 0.29)	-58.4 (-82.09 to -7.15)	13.77 (5.52 to 23.87)	4.62 (2.29 to 7.45)	-66.43 (-85.68 to -25.37)	1.36 (0.49 to 2.61)	0.6 (0.27 to 1.05)	-56.02 (-81.29 to -2.15)	12.42 (4.96 to 21.61)	4.03 (2.02 to 6.52)	-67.57 (-86.08 [illegible] 27.85[illegible]
	**Ambient particulate matter pollution**	0.43 (0.17 to 0.75)	0.18 (0.09 to 0.29)	-58.39 (-82.09 to -7.12)	13.76 (5.52 to 23.87)	4.62 (2.29 to 7.45)	-66.43 (-85.67 to -25.35)	1.36 (0.49 to 2.61)	0.6 (0.27 to 1.05)	-56.01 (-81.28 to -2.04)	12.41 (4.96 to 21.61)	4.02 (2.02 to 6.52)	-67.56 (-86.06 [illegible] 27.83[illegible]
	**Household air pollution from solid fuels**	0 (0 to 0)	0 (0 to 0)	-76.67 (-99.97 to -82.25)	0.01 (0 to 0.07)	0 (0 to 0.01)	-80.45 (-99.98 to -85.1)	0 (0 to 0.01)	0 (0 to 0)	-75.13 (-99.97 to -80.8)	0.01 (0 to 0.06)	0 (0 to 0.01)	-81.02 (-99.98 [illegible] 85.61[illegible]
	**Other environmental risks**	0.17 (-0.02 to 0.38)	0.17 (-0.02 to 0.39)	0.11 (-8.44 to 7.15)	5.29 (-0.66 to 12.39)	3.93 (-0.49 to 9.14)	-25.73 (-31.08 to -19.6)	0.52 (-0.07 to 1.2)	0.49 (-0.07 to 1.16)	-4.29 (-12.36 to 5.23)	4.78 (-0.59 to 11.21)	3.44 (-0.43 to 8)	-28.04 (-33.79 [illegible] 21.51[illegible]

**Lead exposure**	0.17 (-0.02 to 0.38)	0.17 (-0.02 to 0.39)	0.11 (-8.44 to 7.15)	5.29 (-0.66 to 12.39)	3.93 (-0.49 to 9.14)	-25.73 (-31.08 to - 19.6)	0.52 (-0.07 to 1.2)	0.49 (-0.07 to 1.16)	-4.29 (-12.36 to 5.23)	4.78 (-0.59 to 11.21)	3.44 (-0.43 to 8)	-28.0[illegible] (-33.79 [illegible] 21.51[illegible]
**Tobacco**	1.07 (0.92 to 1.24)	0.76 (0.64 to 0.92)	-28.76 (-34.94 to - 22.22)	42.01 (36.09 to 48.47)	26.31 (21.8 to 31.28)	-37.38 (-42.02 to - 32.67)	3.8 (2.66 to 5.17)	3.36 (2.32 to 4.51)	-11.71 (-19.07 to - 2.89)	38.21 (32.92 to 43.83)	22.95 (18.91 to 27.04)	-39.9[illegible] (-44.44 [illegible] 35.17[illegible]
**Smoking**	0.97 (0.84 to 1.11)	0.69 (0.57 to 0.82)	-29.02 (-35.78 to - 22.19)	37.92 (32.97 to 43.28)	23.67 (19.84 to 27.78)	-37.59 (-42.65 to - 32.6)	3.42 (2.4 to 4.61)	3.01 (2.07 to 4.03)	-11.95 (-19.6 to - 2.62)	34.5 (30.19 to 39.13)	20.66 (17.28 to 24.15)	-40.1[illegible] (-45.05 [illegible] 35.08[illegible]
**Secondhand smoke**	0.15 (0.1 to 0.2)	0.09 (0.06 to 0.13)	-35.93 (-41.15 to - 30.62)	6.02 (4.04 to 8.08)	3.37 (2.3 to 4.64)	-43.96 (-48.11 to - 39.12)	0.55 (0.33 to 0.83)	0.44 (0.27 to 0.65)	-20.35 (-28.05 to - 11.49)	5.46 (3.7 to 7.35)	2.93 (1.98 to 3.98)	-46.3[illegible] (-50.38 [illegible] 41.53[illegible]
**Metabolic risks**	2.47 (1.86 to 3)	2.81 (1.99 to 3.52)	13.8 (4.46 to 23.1)	73.71 (54.89 to 90.17)	71.17 (50.32 to 88.81)	-3.45 (-11.21 to 4.04)	7.47 (4.86 to 10.62)	9.22 (5.88 to 13.05)	23.48 (10.95 to 37.03)	66.25 (49.2 to 81.52)	61.95 (43.5 to 77.04)	-6.49 (-14.18 [illegible] 0.89)
**High systolic blood pressure**	2.41 (1.82 to 2.92)	2.72 (1.93 to 3.43)	12.9 (3.39 to 22.82)	70.71 (52.33 to 87.8)	67.36 (46.65 to 84.63)	-4.74 (-13.05 to 2.9)	7.18 (4.72 to 10.27)	8.68 (5.54 to 12.32)	20.96 (8.18 to 35.34)	63.54 (46.69 to 78.24)	58.68 (40.57 to 73.25)	-7.64 (-15.67 [illegible] 0.1)
**High body-mass index**	0.32 (-0.01 to 0.72)	0.45 (-0.02 to 0.92)	40.68 (18.96 to 83.74)	13.03 (-0.39 to 29.26)	16.17 (-0.71 to 33.21)	24.12 (4.26 to 65.74)	1.22 (-0.03 to 2.8)	2.17 (-0.07 to 4.7)	78.44 (50.82 to 146.89)	11.81 (-0.36 to 26.29)	14 (-0.63 to 29.09)	18.5[illegible] (-0.93 [illegible] 57.62[illegible]

**Dietary risks**	0 (-1.11 to 0.86)	0.13 (-0.87 to 1)	249823.96 (-285.85 to 443.16)	-2.09 (-46.33 to 33.59)	2.2 (-33.77 to 30.47)	-205 (-251.44 to 180.4)	-0.16 (-4.42 to 3.13)	0.17 (-4.79 to 4.11)	-203.41 (-291.88 to 237.74)	-1.93 (-42.59 to 30.68)	2.03 (-29.25 to 26.56)	-205.1[illegible] (-246.17[illegible] 186.78[illegible]
**Diet low in fruits**	0.33 (-0.02 to 0.61)	0.3 (-0.02 to 0.58)	-8.53 (-20.55 to -1.15)	12.85 (-1.01 to 23.46)	10.02 (-0.71 to 18.55)	-21.97 (-30.23 to -16.98)	1.22 (-0.1 to 2.51)	1.35 (-0.1 to 2.73)	10.7 (-0.34 to 20.78)	11.63 (-0.91 to 21.25)	8.67 (-0.61 to 16.43)	-25.4[illegible] (-33.68 t[illegible] 20.19[illegible]
**Diet low in vegetables**	0 (0 to 0)	0.02 (0 to 0.04)	2060.68 (1383.73 to 3079.4)	0.02 (0 to 0.05)	0.55 (-0.06 to 1.07)	2283.5 (1578.14 to 3341.16)	0 (0 to 0)	0.07 (-0.01 to 0.13)	2969.89 (2086.8 to 4305.31)	0.02 (0 to 0.04)	0.48 (-0.05 to 0.94)	2213.3[illegible] (1524.56[illegible] 3256.4[illegible]
**Diet high in red meat**	-0.57 (-2.33 to 0.77)	-0.51 (-2.1 to 0.72)	-11.16 (-21.94 to 32490.57)	-24.04 (-99.26 to 30.95)	-18.51 (-76.42 to 24.98)	-22.99 (-29.62 to 40771.71)	-2.25 (-9.5 to 2.78)	-2.51 (-10.46 to 3.22)	11.69 (0.58 to 65244.89)	-21.79 (-88.52 to 27.99)	-16 (-67.57 to 21.63)	-26.5[illegible] (-33.42[illegible] 38618.4[illegible]
**Diet low in fiber**	0.22 (-0.07 to 0.47)	0.14 (-0.04 to 0.32)	-35.91 (-47.91 to -25.32)	8.92 (-2.73 to 18.19)	4.6 (-1.36 to 10.27)	-48.46 (-57.16 to -40.73)	0.85 (-0.26 to 1.93)	0.62 (-0.18 to 1.44)	-26.79 (-39.06 to -15.58)	8.07 (-2.46 to 16.56)	3.98 (-1.17 to 8.8)	-50.7[illegible] (-59.23 t[illegible] 43.24[illegible]
**Diet high in sodium**	0.15 (0 to 0.54)	0.26 (0.01 to 0.82)	69.63 (39.98 to 1570.79)	4.73 (0.02 to 17.08)	7.14 (0.2 to 21.89)	51.13 (21.01 to 1511.99)	0.46 (0 to 1.71)	0.86 (0.02 to 2.74)	85.73 (51.29 to 1665.92)	4.26 (0.01 to 15.25)	6.29 (0.18 to 18.96)	47.39[illegible] (17.6 t[illegible] 1503.5[illegible]
**All risk factors**	3.17 (2.66 to 3.61)	3.38 (2.65 to 4.03)	6.62 (-1.91 to 14.84)	100.51 (81.31 to 114.68)	88.1 (66.8 to 104.52)	-12.35 (-19.59 to -5.63)	9.62 (6.55 to 12.8)	10.88 (7.15 to 14.71)	13.09 (0.98 to 25.07)	90.89 (74.05 to 103.97)	77.21 (58.92 to 92.6)	-15.05[illegible] (-22.03 t[illegible] 8.84)

**Environmental/occupational risks**	0.88 (0.58 to 1.19)	0.73 (0.5 to 0.97)	-17.2 (-39.92 to 10.19)	27.56 (17.72 to 37.72)	17.41 (11.93 to 23.26)	-36.83 (-54.61 to - 14.73)	1.82 (0.81 to 3.16)	1.07 (0.4 to 1.93)	-41.07 (-70.29 to - 1.98)	25.74 (16.84 to 34.85)	16.34 (11.57 to 21.55)	-36.53 (-53.88 t[illegible] 15.31[illegible]
**Behavioral risks**	1.09 (0.32 to 1.72)	0.88 (0.04 to 1.66)	-18.73 (-72.63 to 0.47)	40.96 (10.78 to 64.45)	28.23 (0.37 to 51.95)	-31.08 (-75.84 to - 15.89)	3.73 (0.77 to 6.43)	3.52 (-0.31 to 6.92)	-5.67 (-74.21 to 18.31)	37.23 (9.92 to 58.78)	24.71 (0.68 to 45.15)	-33.63 (-74.56 t[illegible] 18.53[illegible]
**Non-optimal temperature**	0.35 (0.25 to 0.43)	0.41 (0.31 to 0.52)	19.56 (10.39 to 29.28)	10.33 (7.6 to 12.73)	9.55 (7.2 to 12.01)	-7.53 (-13.89 to - 0.42)	N/A	N/A	N/A	10.33 (7.6 to 12.73)	9.55 (7.2 to 12.01)	-7.53 (-13.89 t[illegible] 0.42)
**High temperature**	0.01 (-0.03 to 0.07)	0.03 (-0.03 to 0.11)	86.39 (-172.58 to 302.56)	0.43 (-0.85 to 2.13)	0.63 (-0.77 to 2.5)	46.15 (-161.89 to 211.54)	N/A	N/A	N/A	0.43 (-0.85 to 2.13)	0.63 (-0.77 to 2.5)	46.15 (-161.89[illegible] 211.54[illegible]
**Low temperature**	0.33 (0.28 to 0.38)	0.39 (0.33 to 0.45)	16.89 (8.15 to 25.01)	9.93 (8.36 to 11.4)	8.96 (7.71 to 10.19)	-9.72 (-15.37 to - 3.72)	N/A	N/A	N/A	9.93 (8.36 to 11.4)	8.96 (7.71 to 10.19)	-9.72 (-15.37 t[illegible] 3.72)
**Particulate matter pollution**	0.43 (0.17 to 0.75)	0.18 (0.09 to 0.29)	-58.4 (-82.09 to - 7.15)	13.77 (5.52 to 23.87)	4.62 (2.29 to 7.45)	-66.43 (-85.68 to - 25.37)	1.36 (0.49 to 2.61)	0.6 (0.27 to 1.05)	-56.02 (-81.29 to - 2.15)	12.42 (4.96 to 21.61)	4.03 (2.02 to 6.52)	-67.57 (-86.08 t[illegible] 27.85[illegible]

2.11 Table S7a: Age-standardized rate and percentage change of incidence, prevalence, and mortality for stroke over time by age groups, both sexes, US, 1990-2021

Disease	Age	Incidence			Prevalence			Mortality		
		1990	2021	change, %	1990	2021	change, %	1990	2021	change, %
All Stroke	All ages	140.85 (119.37 to 165.32)	123.86 (107.71 to 141.18)	-12.06 (-16.52 to -7.03)	1482.8 (1368.93 to 1611.38)	1893.72 (1770.26 to 2032.49)	27.71 (22.27 to 33.1)	57.72 (51.26 to 61.18)	57.65 (48.9 to 62.25)	-0.13 (-5.24 to 3.44)
	15-49 years	31.73 (26.13 to 39.14)	28.22 (23.13 to 34.82)	-11.06 (-14.33 to -7.96)	597.64 (525.09 to 667.26)	612.34 (550.54 to 671.86)	2.46 (-1.58 to 6.43)	4.57 (4.45 to 4.7)	3.29 (3.08 to 3.47)	-28.06 (-33.19 to -23.57)
	50-74 years	307.77 (242.55 to 396.33)	183.35 (149.93 to 233.07)	-40.43 (-44 to -36.52)	3347.23 (2952.2 to 3703.97)	3337.68 (3024.63 to 3646.68)	-0.29 (-5.6 to 5.51)	79.63 (76.52 to 81.88)	50.71 (48.19 to 52.69)	-36.32 (-38.74 to -33.76)
	75+ years	1144.17 (912.2 to 1422.19)	793.58 (666.51 to 949.48)	-30.64 (-36.74 to -23.33)	9036.35 (7897.58 to 10159.89)	8787.33 (7950.91 to 9638.25)	-2.76 (-9.06 to 4.79)	759.79 (645.24 to 819.9)	595.34 (479.72 to 655.82)	-21.64 (-26.06 to -18.92)
Ischemic stroke	All ages	111.29 (90.6 to 135.91)	93.67 (77.9 to 111.31)	-15.84 (-20.73 to -9.94)	1220.17 (1107.05 to 1344.93)	1544.07 (1413.02 to 1678.85)	26.55 (20.17 to 33.16)	39.17 (34 to 41.83)	34.74 (28.44 to 38.07)	-11.31 (-16.72 to -8.08)

	**15-49 years**	19.66 (14.26 to 26.84)	18.63 (13.63 to 25.58)	-5.22 (-9.72 to -1.55)	413.73 (346.76 to 481.26)	428.92 (369.48 to 484.72)	3.67 (-1.85 to 9.84)	0.5 (0.49 to 0.52)	0.4 (0.38 to 0.43)	-19.42 (-25.13 to -14.09)
	**50-74 years**	247.27 (184.54 to 332.76)	139.58 (106.87 to 184.22)	-43.55 (-47.35 to -39.13)	2785.2 (2390.15 to 3156.35)	2720.12 (2417.06 to 3028.71)	-2.34 (-8.59 to 4.27)	36.57 (34.89 to 37.69)	17.25 (16.35 to 18.01)	-52.82 (-54.87 to -50.7)
	**75+ years**	947.88 (702.28 to 1228.47)	616.21 (483.36 to 767.65)	-34.99 (-41.65 to -27.33)	8167.21 (7025.38 to 9304.33)	7671.36 (6868.36 to 8551.95)	-6.07 (-12.91 to 2.22)	610.47 (514.98 to 659.28)	426.3 (338.85 to 472.96)	-30.17 (-34.49 to -27.64)
**Intracerebral hemorrhage**	**All ages**	21.73 (17.99 to 25.34)	21.25 (18.18 to 24.33)	-2.22 (-7.75 to 4.6)	166.3 (148 to 185.97)	226.51 (202.98 to 252.1)	36.21 (28.73 to 42.97)	14.11 (13.06 to 14.7)	17.49 (15.64 to 18.56)	23.91 (17.26 to 28.82)
	**15-49 years**	6.45 (4.79 to 8.27)	4.86 (3.72 to 6.13)	-24.56 (-30.1 to -18.06)	110.97 (93.29 to 131.11)	105.62 (90.48 to 122.94)	-4.82 (-8.67 to 0.09)	2.1 (2.04 to 2.17)	1.68 (1.57 to 1.78)	-20.19 (-26.02 to -14.96)
	**50-74 years**	44.44 (32.9 to 57.74)	29.98 (23.38 to 37.78)	-32.54 (-38.04 to -24.9)	341.49 (290.79 to 397.56)	380.14 (326.68 to 439.36)	11.32 (5.11 to 18.37)	32.26 (31.1 to 33.2)	25.19 (24 to 26.22)	-21.93 (-25.21 to -18.69)
	**75+ years**	170.42 (129.96 to 218.61)	141.34 (114.28 to 171.25)	-17.06 (-24.55 to -7.05)	648.28 (526.67 to 781.78)	893.01 (736.92 to 1070.44)	37.75 (29.52 to 46.72)	125.97 (109.91 to 134.41)	134.16 (110.74 to 145.92)	6.5 (0.23 to 11)

**Subarachnoid hemorrhage**	**All ages**	7.82 (6.71 to 9.22)	8.95 (7.88 to 10.31)	14.37 (8.62 to 22.29)	104.82 (93.11 to 118.76)	136.57 (123.23 to 151.22)	30.3 (24.3 to 36.67)	4.44 (4.19 to 4.59)	5.42 (4.9 to 5.74)	22.15 (15.33 to 27.51)
	**15-49 years**	5.62 (4.17 to 7.12)	4.72 (3.64 to 5.89)	-15.99 (-21.08 to -10.09)	73.97 (63.27 to 86.76)	78.94 (69.32 to 90.1)	6.72 (1.91 to 12.91)	1.97 (1.91 to 2.03)	1.21 (1.13 to 1.27)	-38.67 (-42.86 to -34.85)
	**50-74 years**	16.06 (12.59 to 20.97)	13.8 (10.89 to 17.68)	-14.09 (-18.76 to -7.76)	239.13 (204.75 to 279.33)	257.8 (228.01 to 290.74)	7.81 (0.9 to 14.63)	10.81 (10.44 to 11.14)	8.27 (7.86 to 8.6)	-23.44 (-26.84 to -20.3)
	**75+ years**	25.86 (19.62 to 33.71)	36.03 (28.65 to 44.2)	39.32 (24.97 to 57.07)	302.35 (241.98 to 363.08)	322.74 (277.07 to 368.41)	6.74 (-2.58 to 16.38)	23.35 (20 to 25.04)	34.88 (28.73 to 38.13)	49.43 (41.76 to 55.47)

2.12 Table S7b: Age-standardized rate and percentage change of DALYs, YLDs, and YLLs for stroke over time by age groups, both sexes, US, 1990-2021

2.13

Disease	Age	DALYs			YLDs			YLLs		
		1990	2021	change, %	1990	2021	change, %	1990	2021	change, %
All Stroke	All ages	1202.59 (1107.51 to 1280.58)	1176 (1062.27 to 1270.99)	-2.21 (-5.86 to 0.63)	211.29 (150.07 to 270.56)	265.15 (192.78 to 338.15)	25.49 (19.66 to 31.47)	991.3 (913.55 to 1034.67)	910.85 (815.91 to 966.15)	-8.12 (-12.42 to -4.85)
	15-49 years	308.65 (286.18 to 332.38)	242.11 (219.65 to 267.01)	-21.56 (-25.66 to -17.96)	80.45 (56.75 to 104.98)	81.22 (57.66 to 104.52)	0.96 (-3.53 to 5.77)	228.2 (222.11 to 234.41)	160.89 (151.06 to 169.84)	-29.5 (-34.52 to -25.07)
	50-74 years	2444.11 (2317.6 to 2592.95)	1730.58 (1610.83 to 1852.21)	-29.19 (-32.03 to -26.97)	438.92 (314.44 to 571.82)	428.09 (311.83 to 551.37)	-2.47 (-8.26 to 3.59)	2005.19 (1930.85 to 2061.55)	1302.49 (1243.5 to 1354.07)	-35.04 (-37.54 to -32.35)
	75+ years	10361.42 (9085.85 to 11185.61)	7927.59 (6690.89 to 8698.05)	-23.49 (-26.61 to -21.27)	1485.92 (1049.45 to 1924.9)	1428.31 (1021.7 to 1835.9)	-3.88 (-10.23 to 3.81)	8875.51 (7658.66 to 9506.48)	6499.28 (5308.22 to 7108.16)	-26.77 (-30.73 to -24.13)
Ischemic stroke	All ages	720.31 (647.31 to 783.12)	654.77 (574.03 to 723.88)	-9.1 (-12.61 to -6.26)	172.54 (122.61 to 222.62)	213.8 (154.66 to 271.21)	23.91 (17.14 to 30.76)	547.77 (489.47 to 578.86)	440.97 (374.62 to 475.29)	-19.5 (-23.81 to -16.61)

	**15-49 years**	79.45 (63.17 to 96.94)	75.41 (60.01 to 92.12)	-5.09 (-9.52 to -0.48)	54.66 (38.21 to 72.28)	55.83 (39.87 to 72.74)	2.14 (-4.2 to 9.14)	24.79 (24.09 to 25.53)	19.58 (18.39 to 20.71)	-21.03 (-26.58 to -15.82)
	**50-74 years**	1211.03 (1108.6 to 1329.51)	754.02 (659.01 to 854.74)	-37.74 (-41.31 to -34.59)	360.82 (255.96 to 473.46)	344.12 (248.52 to 444.85)	-4.63 (-11.28 to 2.49)	850.21 (812.29 to 876.6)	409.9 (389.41 to 427.49)	-51.79 (-53.91 to -49.71)
	**75+ years**	8293.89 (7249.91 to 9000.52)	5716.52 (4791.13 to 6326.78)	-31.08 (-33.9 to -28.99)	1324.2 (920.17 to 1725.46)	1224.98 (880.59 to 1565.36)	-7.49 (-14.4 to 0.78)	6969.69 (5970.87 to 7479.97)	4491.53 (3625.66 to 4946.35)	-35.56 (-39.43 to -33.22)
**Intracerebral hemorrhage**	**All ages**	334.72 (317.53 to 346.94)	376.55 (350.16 to 396.02)	12.5 (7.42 to 16.67)	23.72 (16.66 to 30.94)	32.1 (23.39 to 41.17)	35.35 (27.43 to 43.42)	311 (295.57 to 320.75)	344.44 (318.53 to 361.71)	10.75 (5.49 to 15.13)
	**15-49 years**	118.95 (113.58 to 125.53)	95.53 (89.24 to 102.43)	-19.7 (-24.71 to -14.86)	15.3 (10.56 to 20.9)	14.4 (9.99 to 19.5)	-5.92 (-12.8 to 0.59)	103.65 (100.61 to 106.68)	81.13 (75.91 to 85.92)	-21.73 (-27.46 to -16.65)
	**50-74 years**	892.39 (861.95 to 920.41)	710.77 (676.61 to 739.45)	-20.35 (-23.7 to -17.4)	45.44 (32.05 to 59.89)	49.51 (36.39 to 64.71)	8.95 (0.99 to 17.51)	846.95 (818.1 to 871.77)	661.26 (631.75 to 688.36)	-21.93 (-25.28 to -18.63)
	**75+ years**	1721.13 (1524.46 to 1836.61)	1753.1 (1491.78 to 1899.01)	1.86 (-3.21 to 5.54)	110.76 (76.81 to 147.32)	149.1 (103.53 to 196.1)	34.61 (25.04 to 44.69)	1610.36 (1424.11 to 1707.98)	1604.01 (1345.79 to 1730.65)	-0.39 (-5.85 to 3.75)

**Subarachnoid hemorrhage**	**All ages**	147.57 (141.91 to 153.31)	144.68 (136.12 to 152.97)	-1.96 (-6.33 to 1.69)	15.03 (10.71 to 19.51)	19.25 (13.63 to 24.64)	28.04 (20.58 to 36.29)	132.53 (128.03 to 136.04)	125.43 (117.58 to 131.23)	-5.36 (-10.28 to -1.42)
	**15-49 years**	110.24 (106.06 to 115.04)	71.17 (66.47 to 76.05)	-35.44 (-39.25 to -31.87)	10.48 (7.14 to 13.67)	10.99 (7.48 to 14.37)	4.88 (-2.64 to 13.24)	99.76 (97.18 to 102.72)	60.18 (56.39 to 63.54)	-39.67 (-43.87 to -35.9)
	**50-74 years**	340.68 (327 to 354.81)	265.8 (252.52 to 280.08)	-21.98 (-25.1 to -19.03)	32.66 (23.37 to 43.49)	34.46 (24.57 to 44.84)	5.51 (-3 to 14.43)	308.03 (297.68 to 317.68)	231.34 (220.16 to 240.48)	-24.9 (-28.15 to -21.84)
	**75+ years**	346.4 (306.98 to 375.32)	457.97 (387.76 to 500.2)	32.21 (25.31 to 37.63)	50.95 (35.94 to 68.98)	54.23 (40 to 70.8)	6.45 (-4 to 18.45)	295.46 (256.49 to 315.66)	403.74 (336.55 to 438.16)	36.65 (29.72 to 42.04)

3 Supplementary Figures

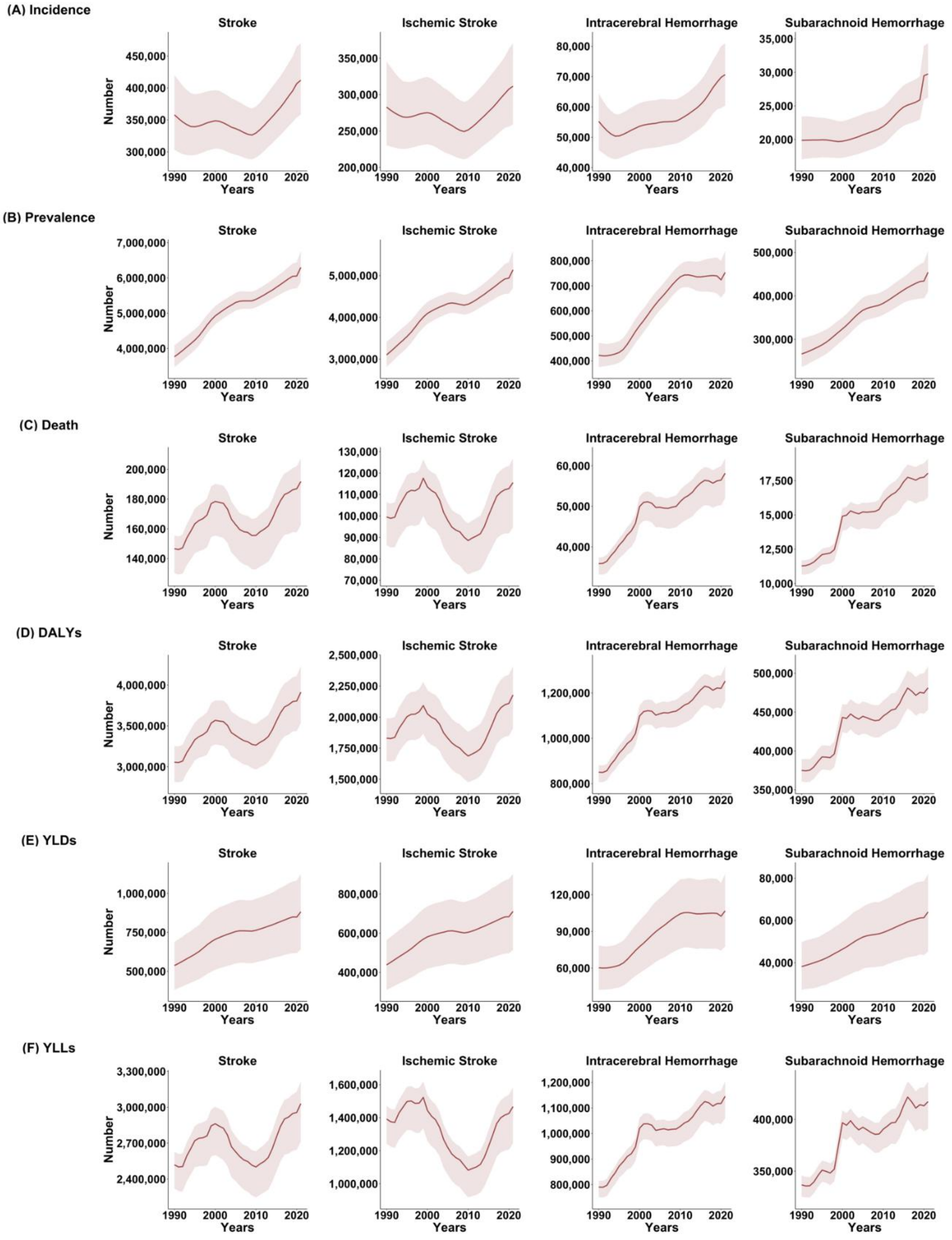

3.1 Figure S1: Annual count change in (A) incidence, (B) prevalence, (C) death, (D) DALYs, (E) YLDs, and (F) YLLs for stroke, 1990-2021, US

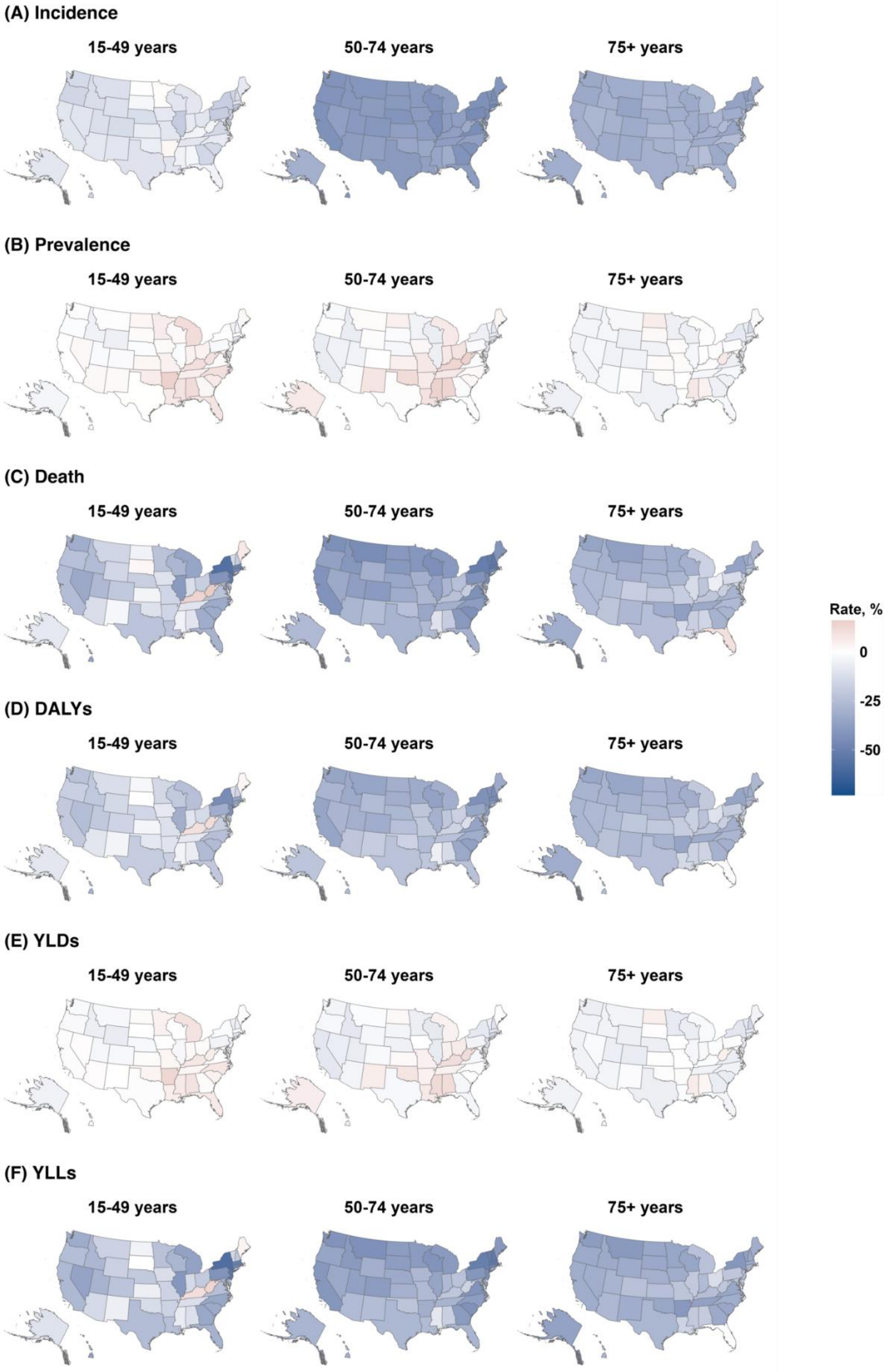

3.2 **Figure S2: Percentage change by age groups of 15-49 years, 50-74 years, and 75+ years in (A) incidence, (B) prevalence, (C) death, (D) DALYs, (E) YLDs, and (F) YLLs rate for stroke, 1990-2021, US**

(A) Incidence

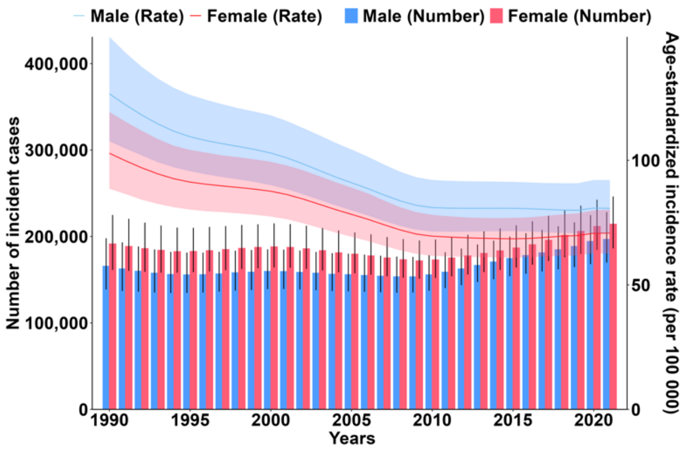

(B) Prevalence

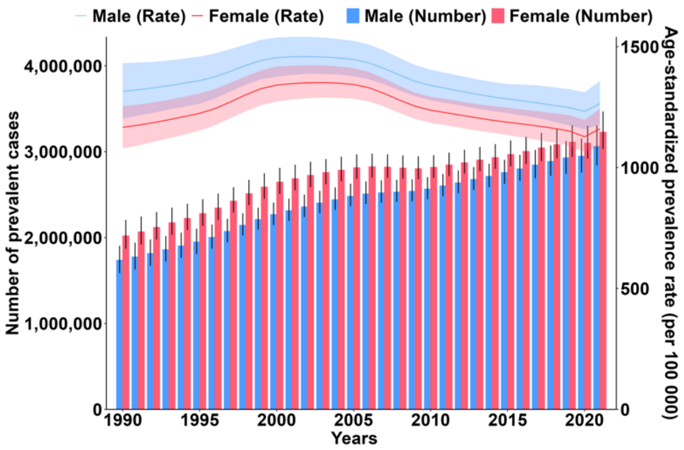

(C) Death

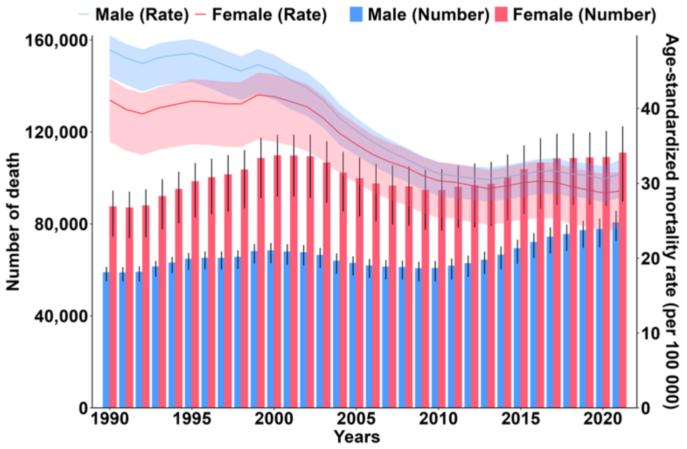

(D) DALYs

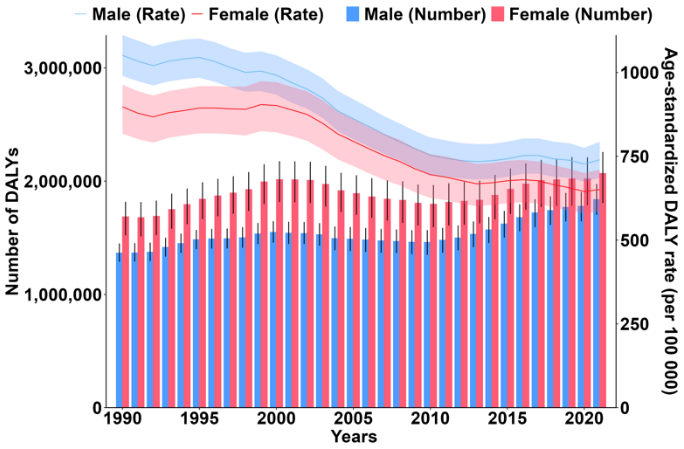

(E) YLDs

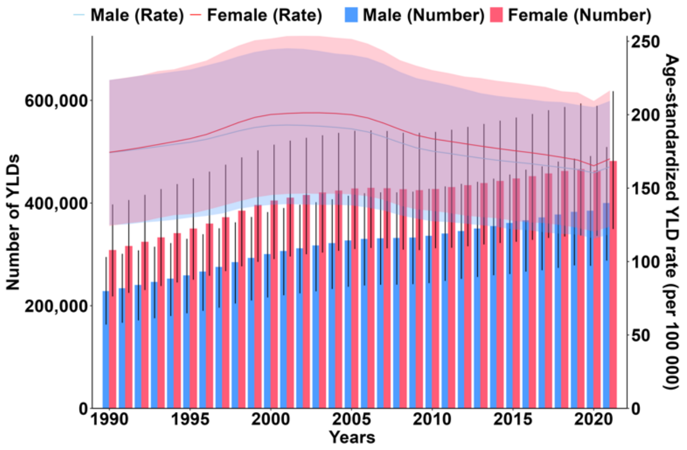

(F) YLLs

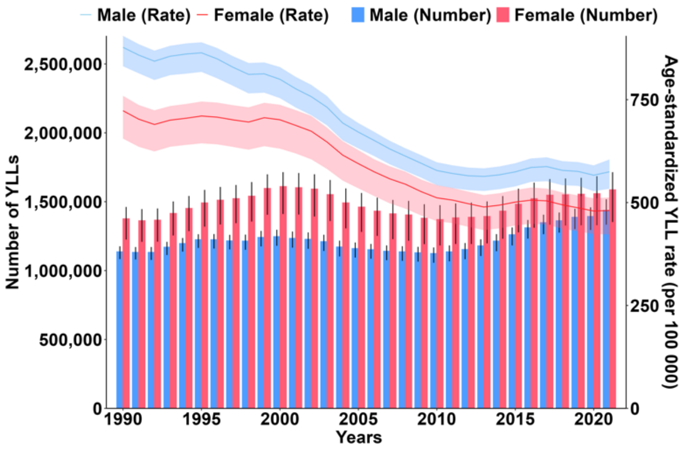

3.3 Figure S3a: Annual age standardized rate and all age number change in (A) incidence, (B) prevalence, (C) death, (D) DALYs, (E) YLDs, and (F) YLLs for all stroke, by sex, 1990-2021, US

(A) Incidence

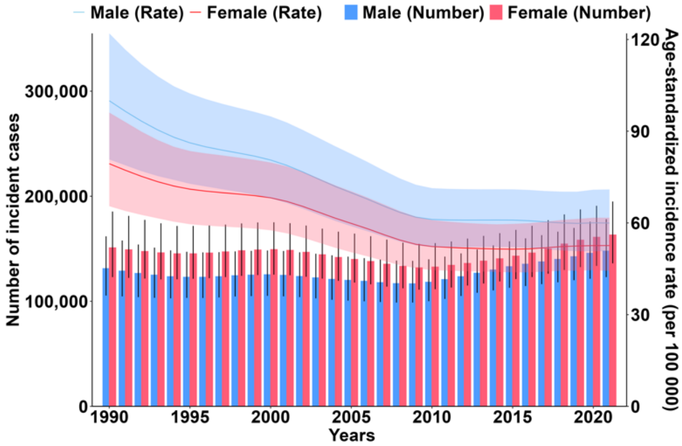

(B) Prevalence

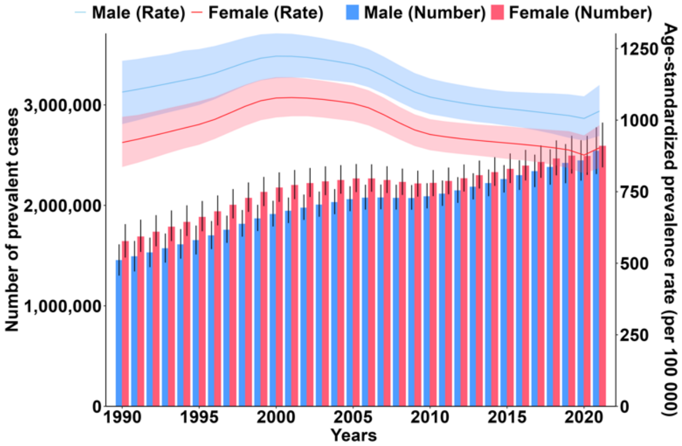

(C) Death

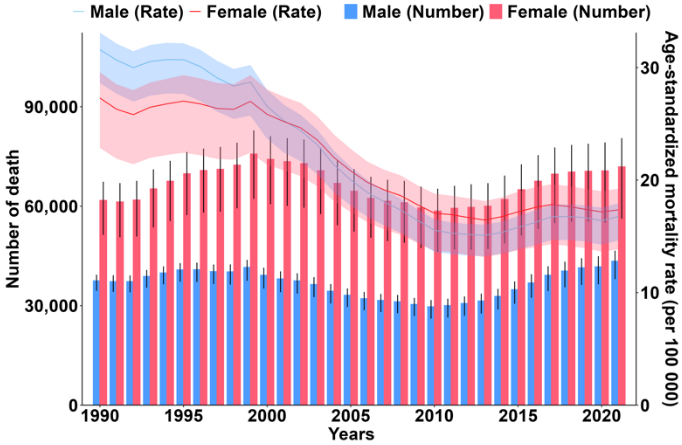

(D) DALYs

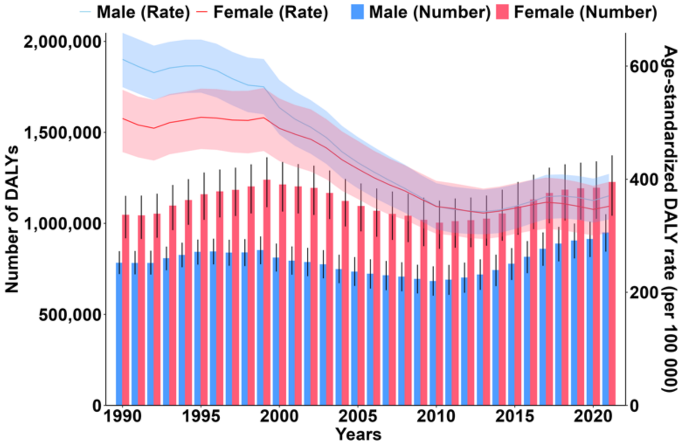

(E) YLDs

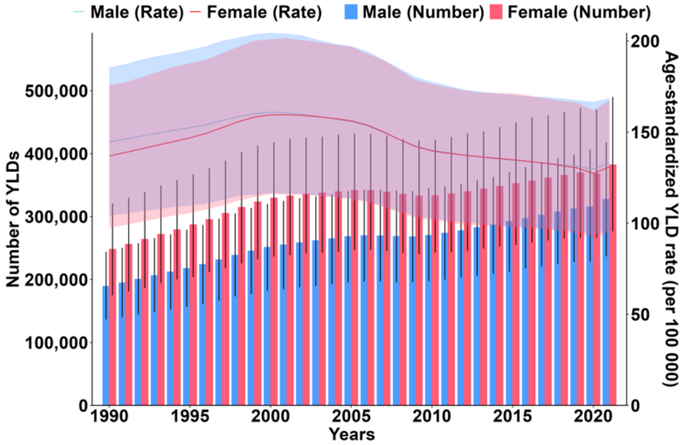

(F) YLLs

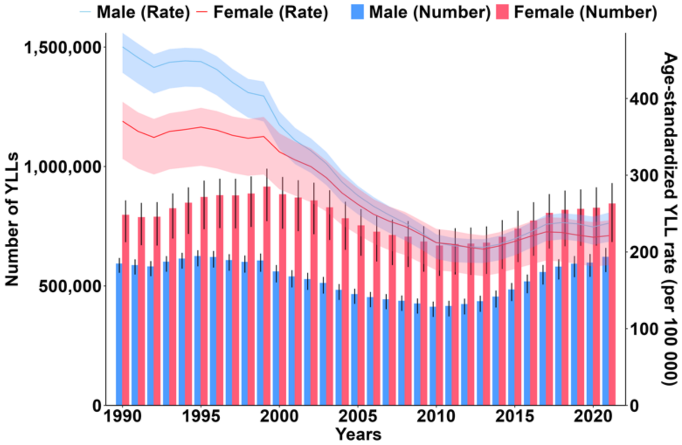

3.4 Figure S3b: Annual age standardized rate and all age number change in (A) incidence, (B) prevalence, (C) death, (D) DALYs, (E) YLDs, and (F) YLLs for ischemic stroke, by sex, 1990-2021, US

(A) Incidence

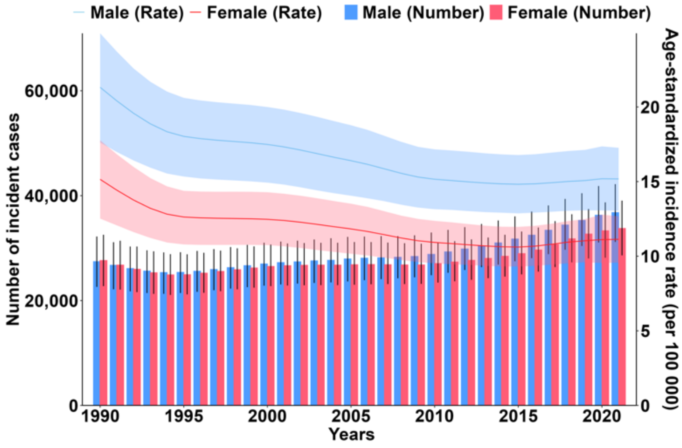

(B) Prevalence

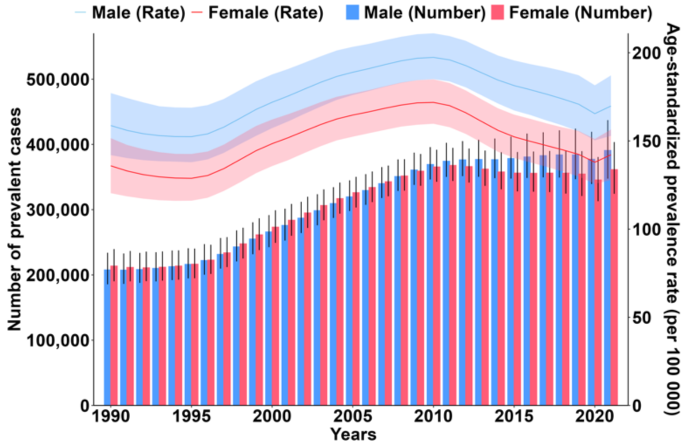

(C) Death

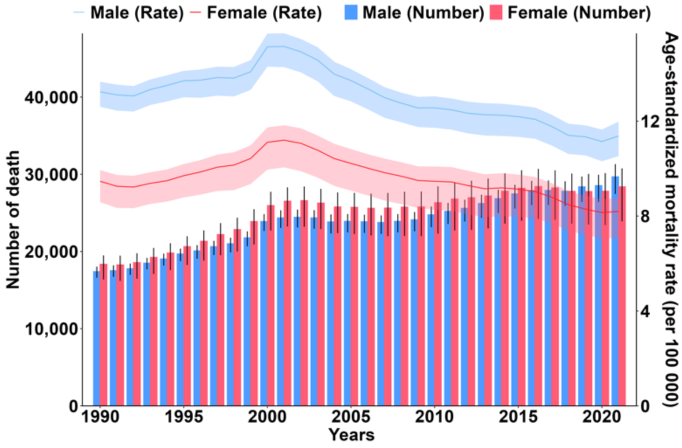

(D) DALYs

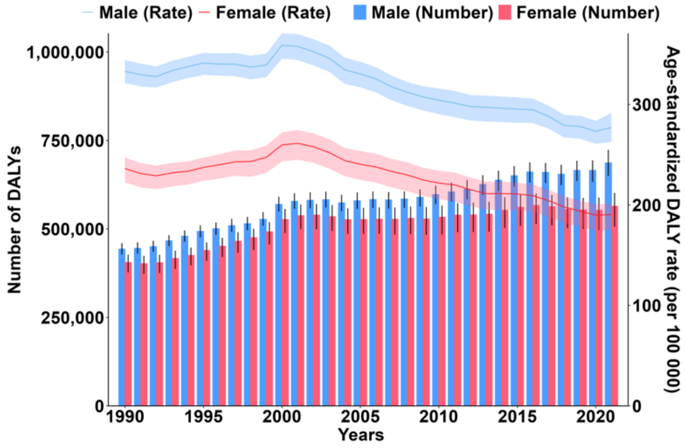

(E) YLDs

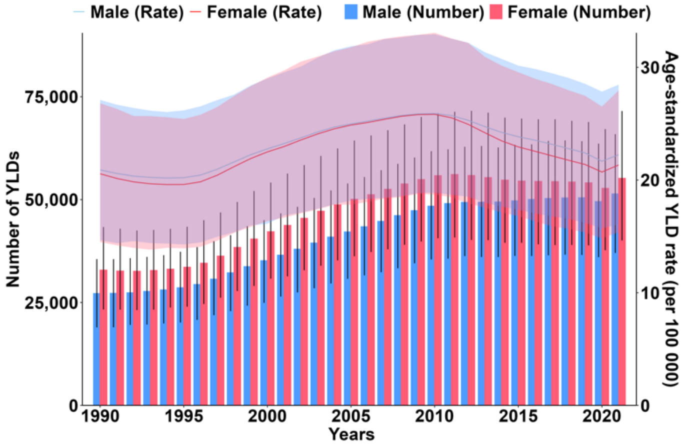

(F) YLLs

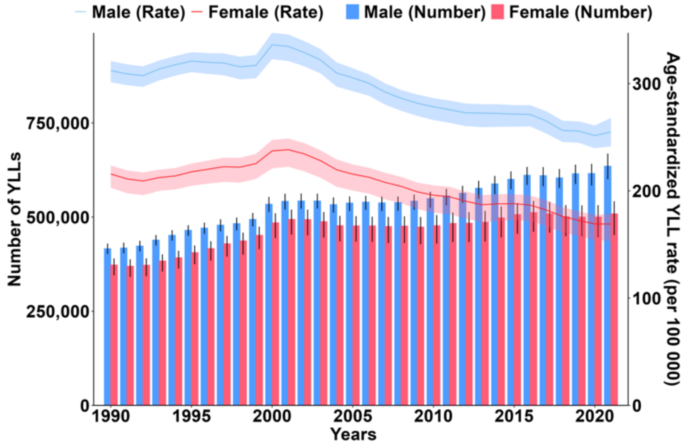

3.5 Figure S3c: Annual age standardized rate and all age number change in (A) incidence, (B) prevalence, (C) death, (D) DALYs, (E) YLDs, and (F) YLLs for intracerebral hemorrhage, by sex, 1990-2021, US

(A) Incidence

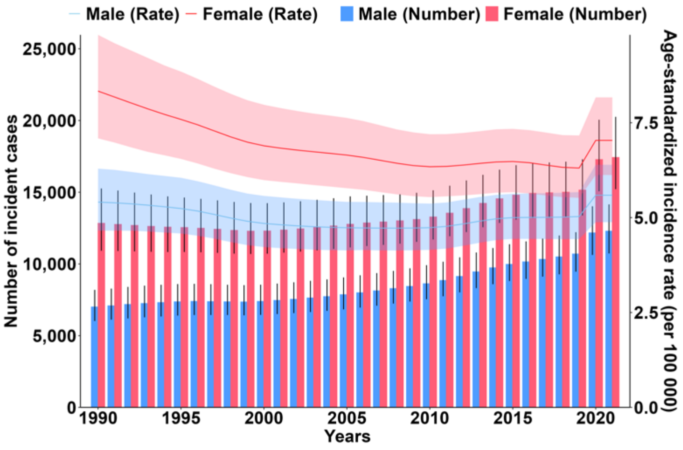

(B) Prevalence

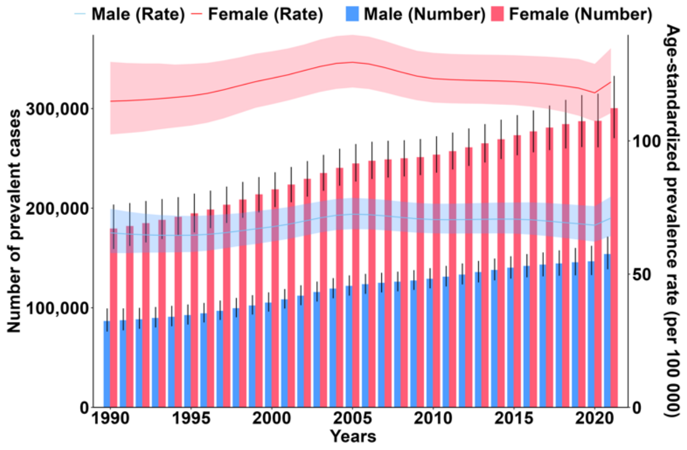

(C) Death

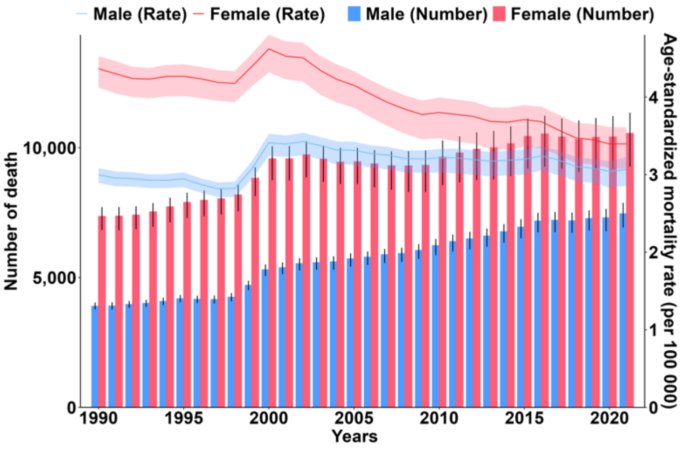

(D) DALYs

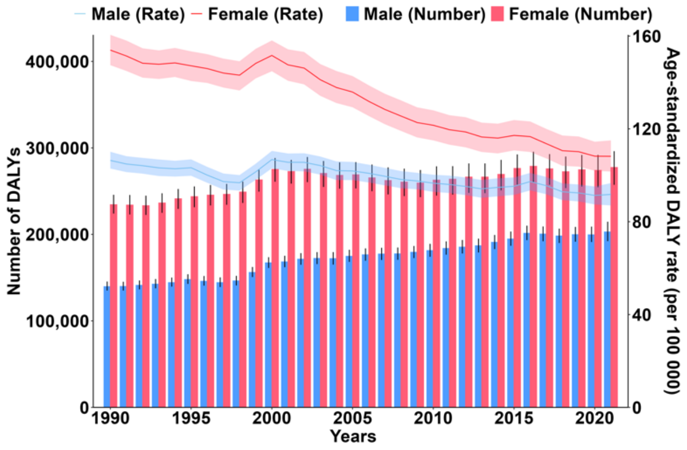

(E) YLDs

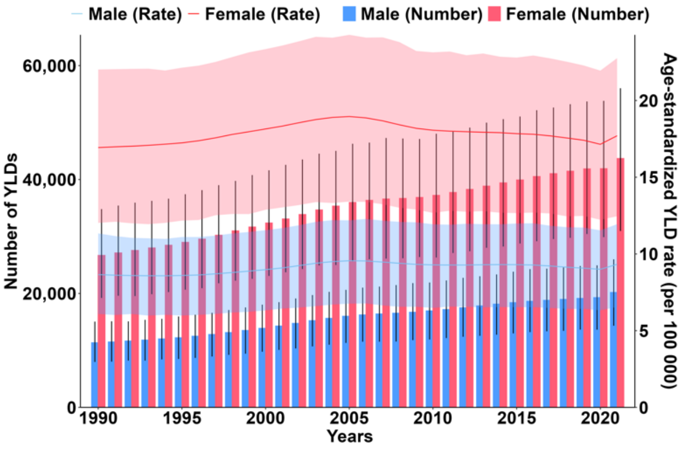

(F) YLLs

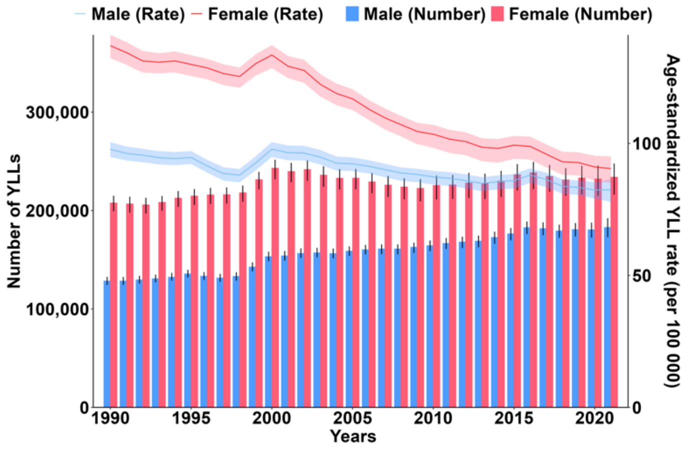

3.6 **Figure S3d: Annual age standardized rate and all age number change in (A) incidence, (B) prevalence, (C) death, (D) DALYs, (E) YLDs, and (F) YLLs for subarachnoid hemorrhage, by sex, 1990-2021, US**

(A) Death

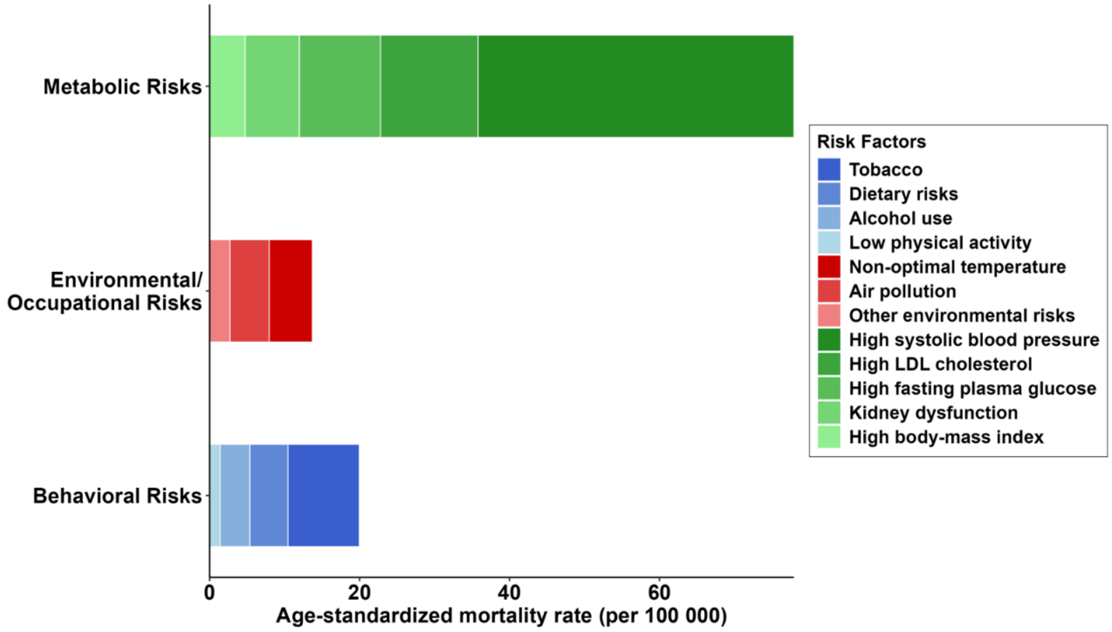

(B) DALYs

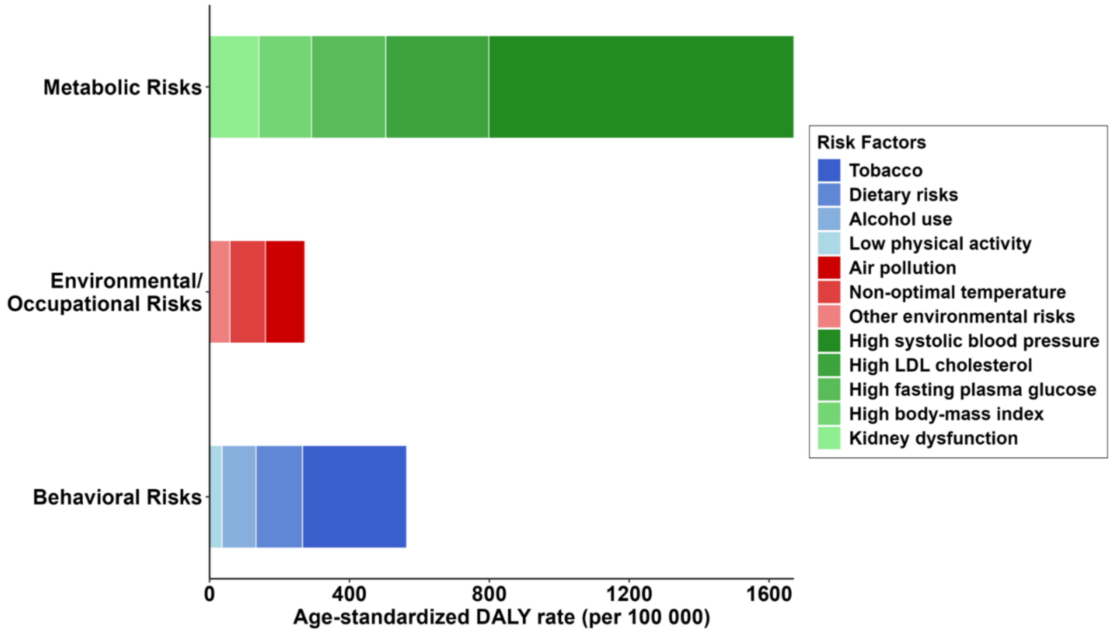

(C) YLDs

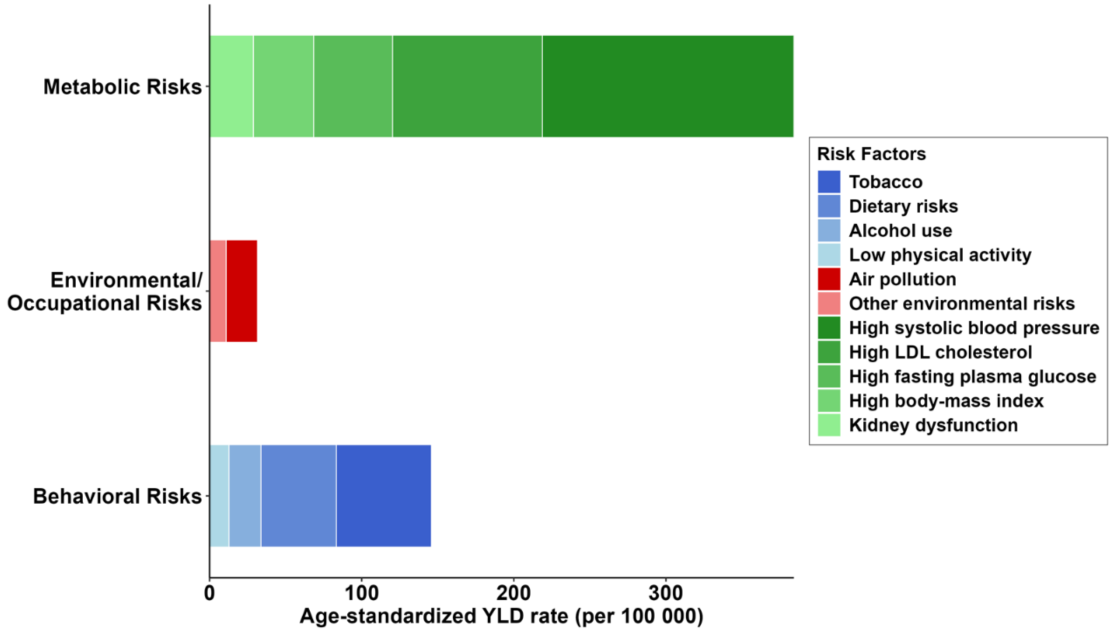

(D) YLLs

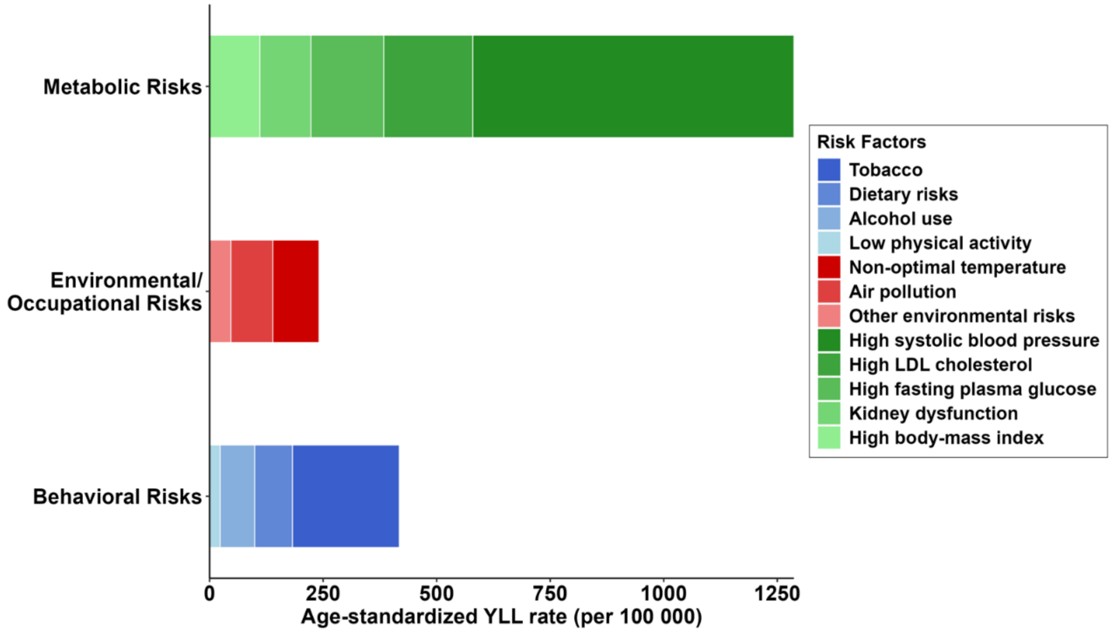

3.7 **Figure S4a: Age-standardized (A) mortality, (B) DALY, (C) YLD, and (D) YLL rate for stroke by types of risk factors, 2021, US**

(A) Death

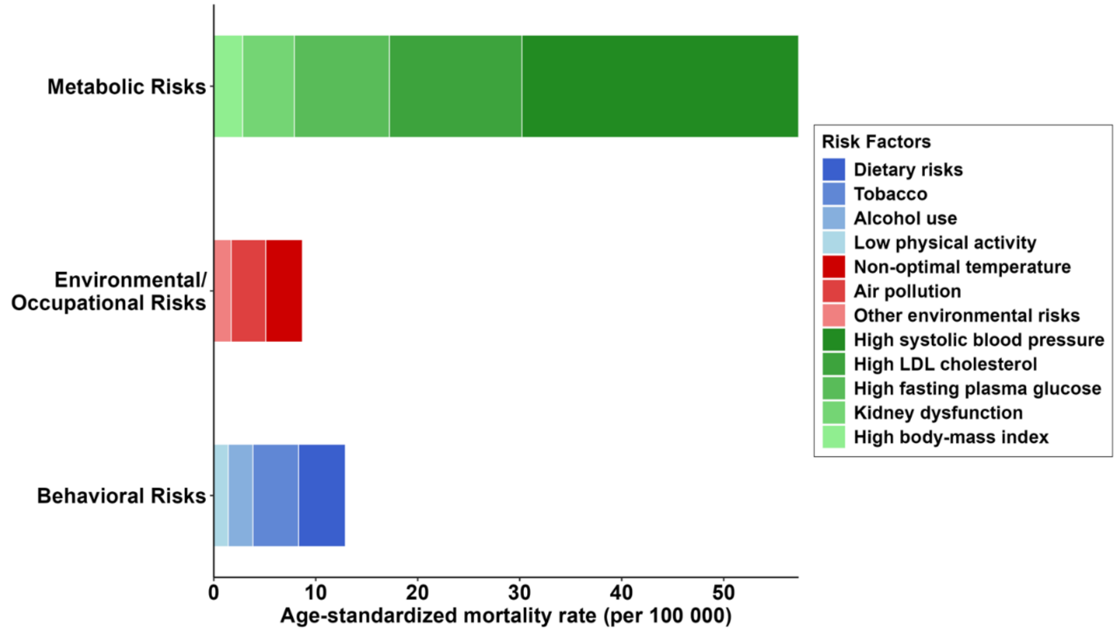

(B) DALYs

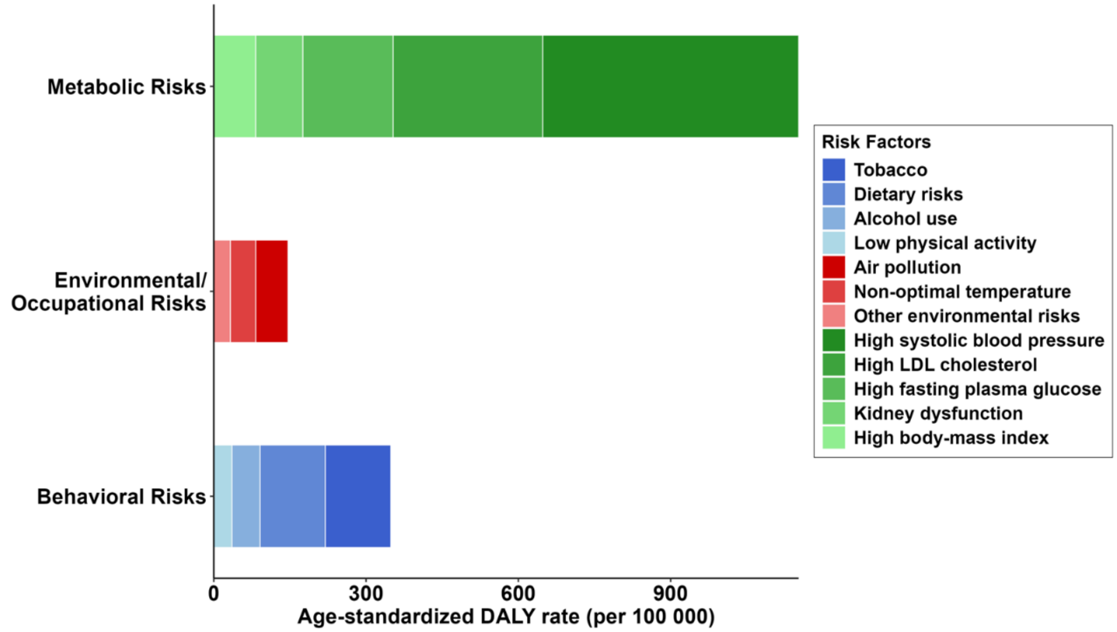

(C) YLDs

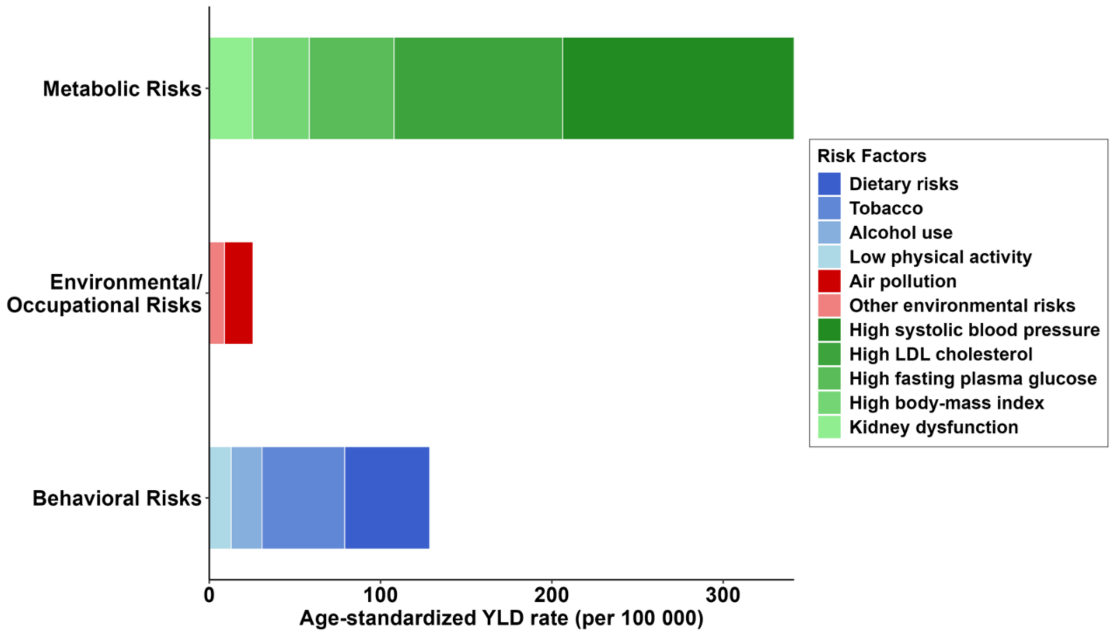

(D) YLLs

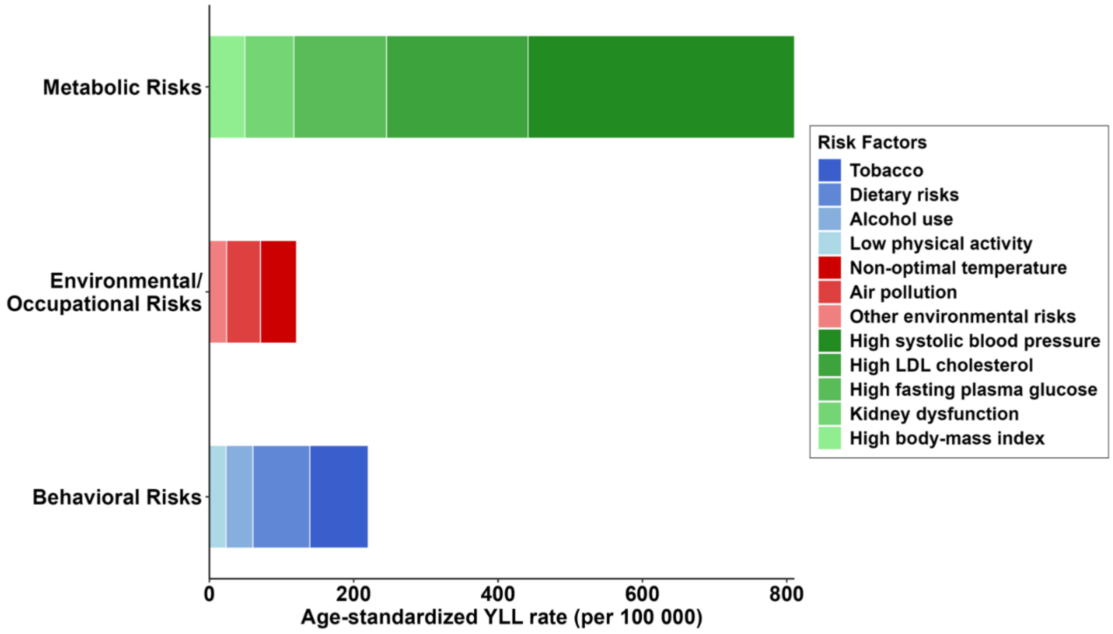

3.8 **Figure S4b: Age-standardized (A) mortality, (B) DALY, (C) YLD, and (D) YLL rate for ischemic stroke by types of risk factors, 2021, US**

(A) Death

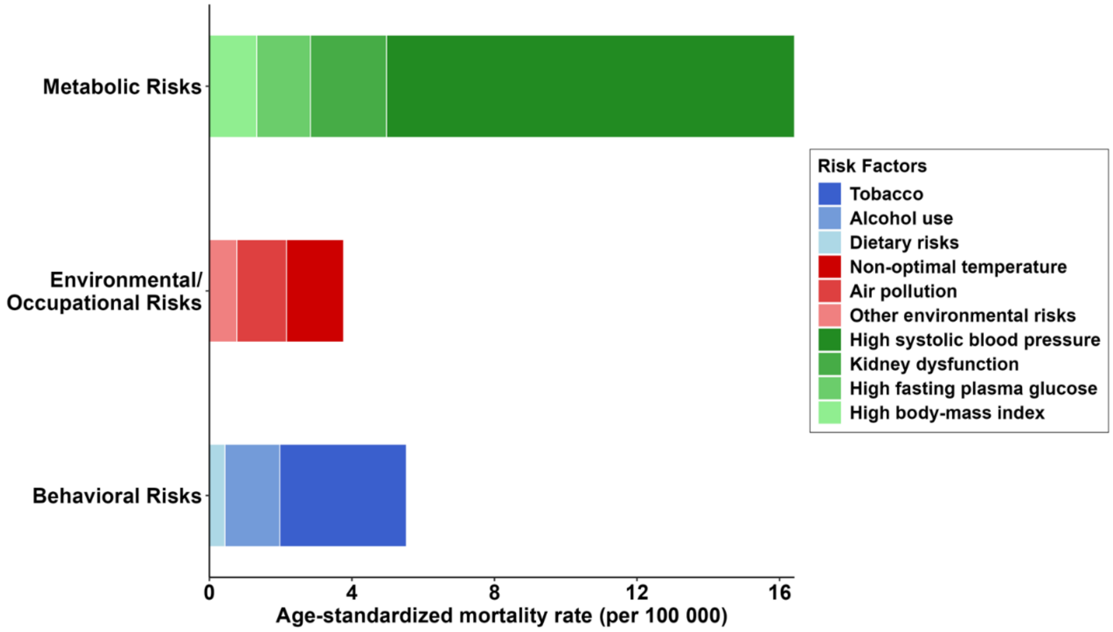

(B) DALYs

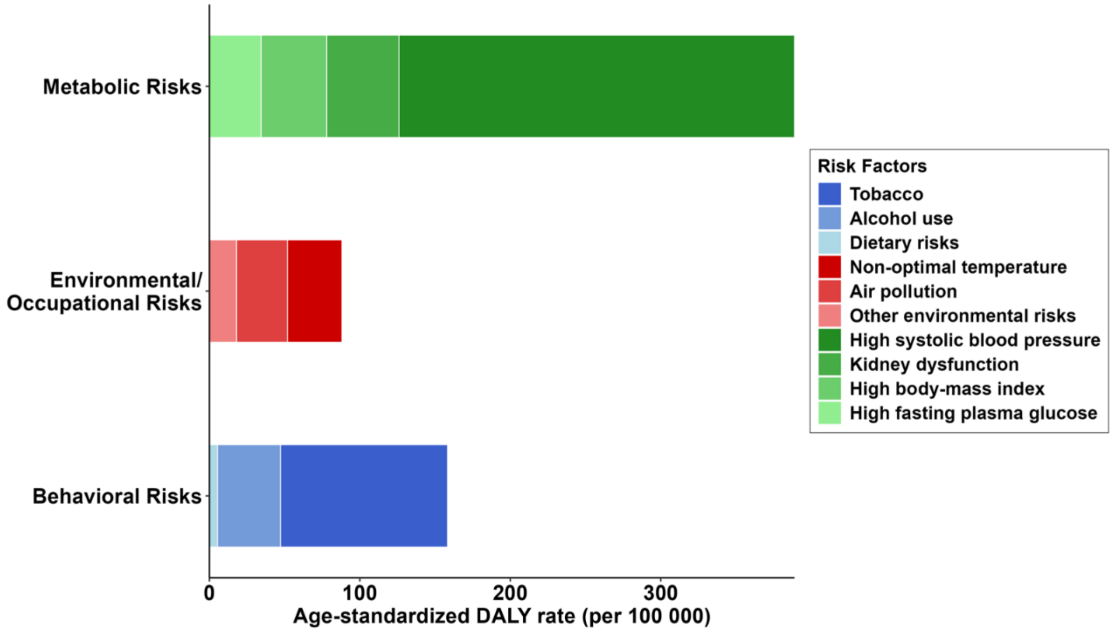

(C) YLDs

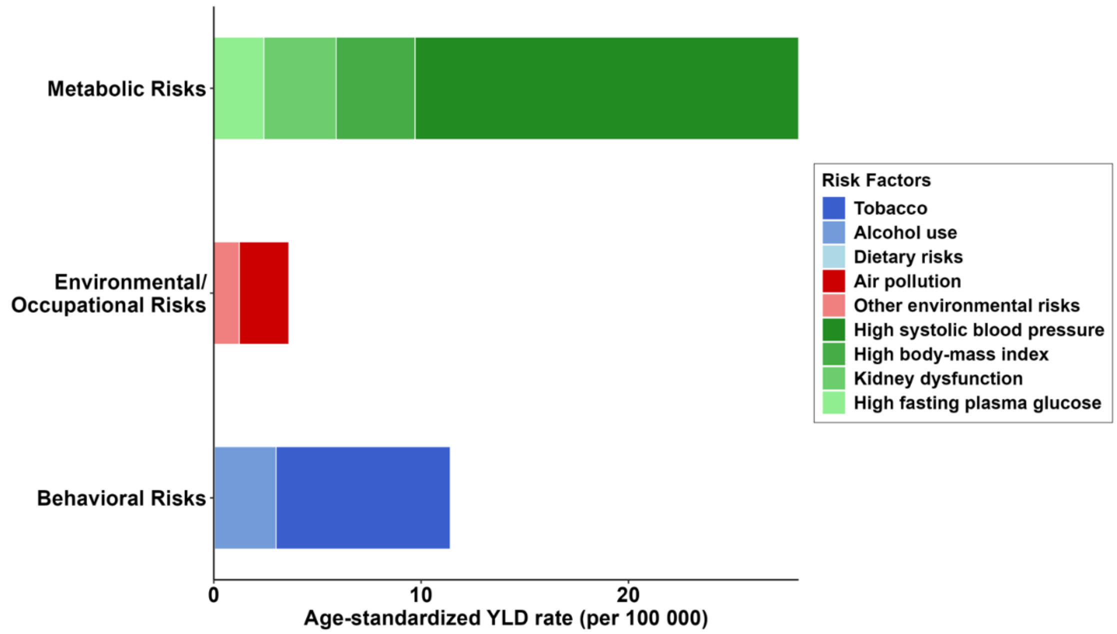

(D) YLLs

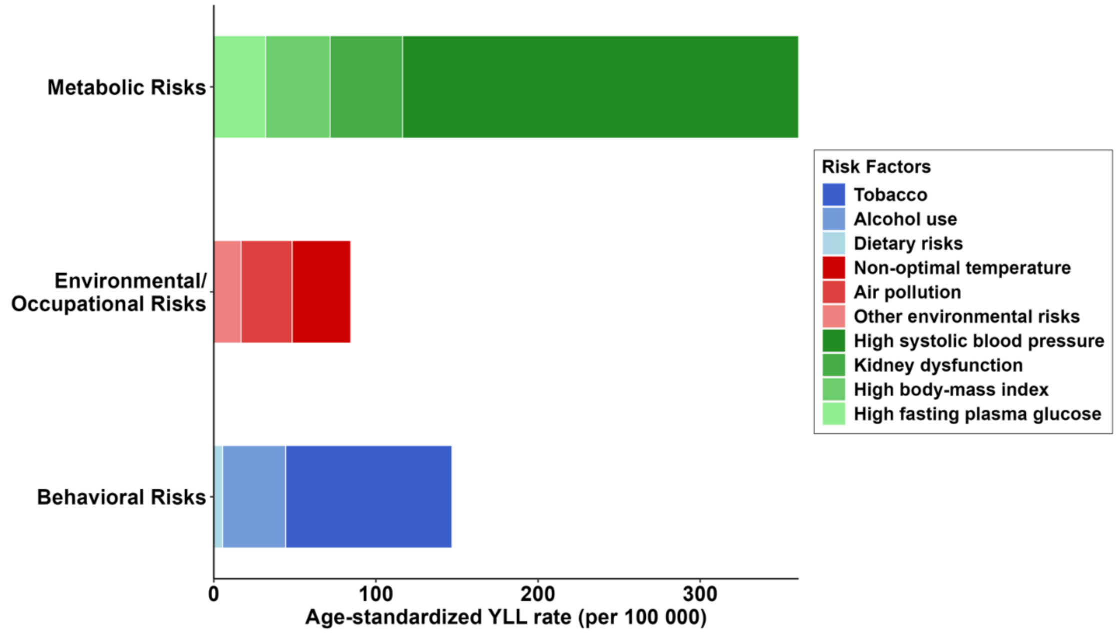

3.9 **Figure S4c: Age-standardized (A) mortality, (B) DALY, (C) YLD, and (D) YLL rate for intracerebral hemorrhage by types of risk factors, 2021, US**

(A) Death

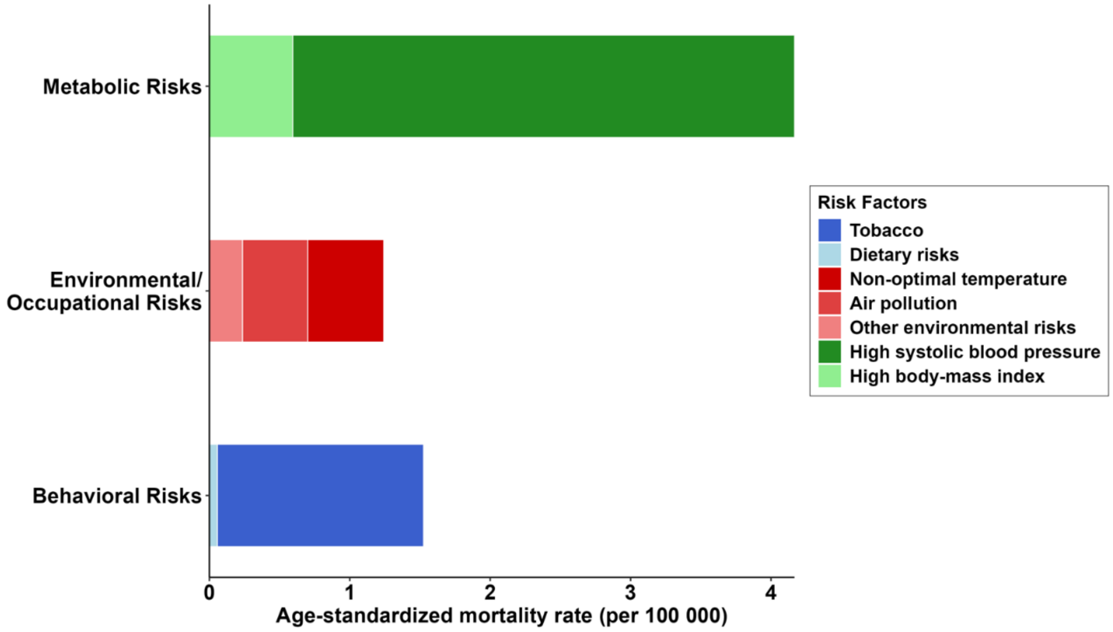

(B) DALYs

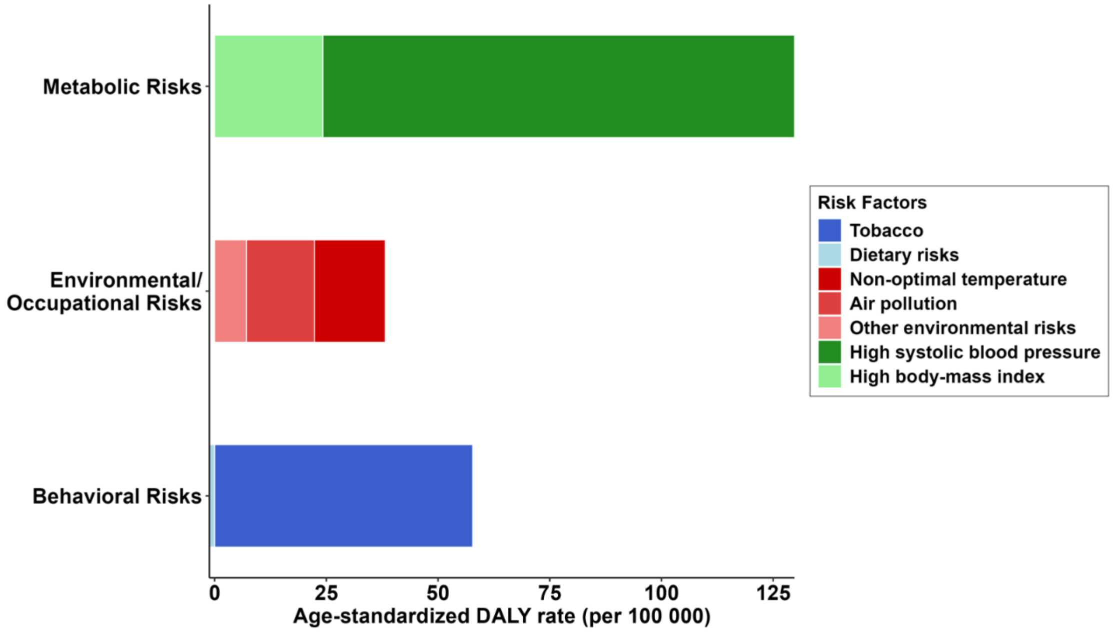

(C) YLDs

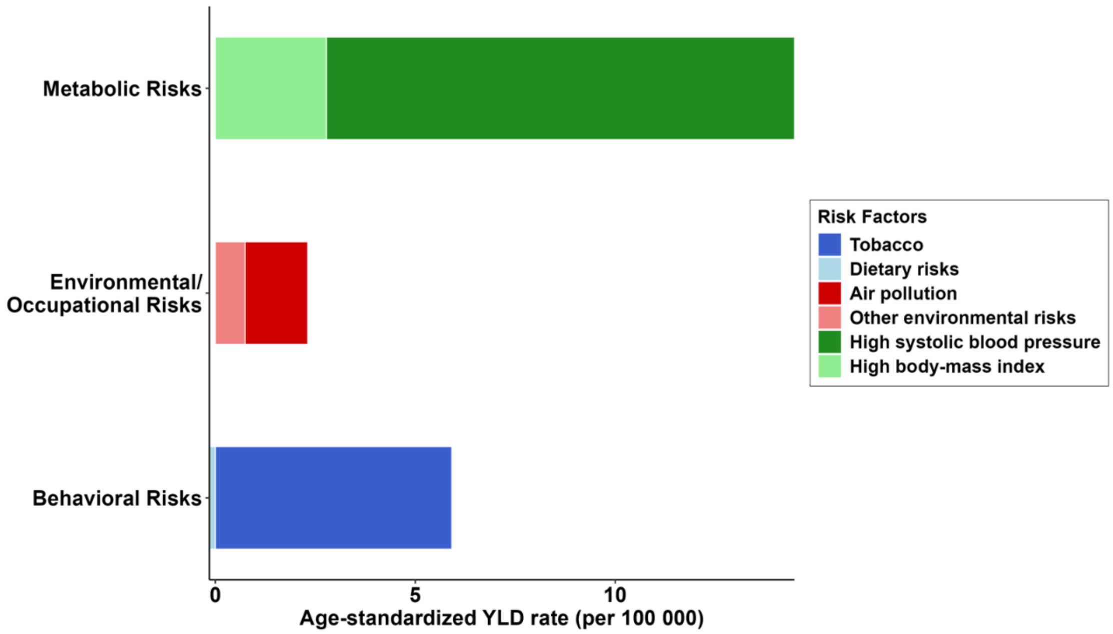

(D) YLLs

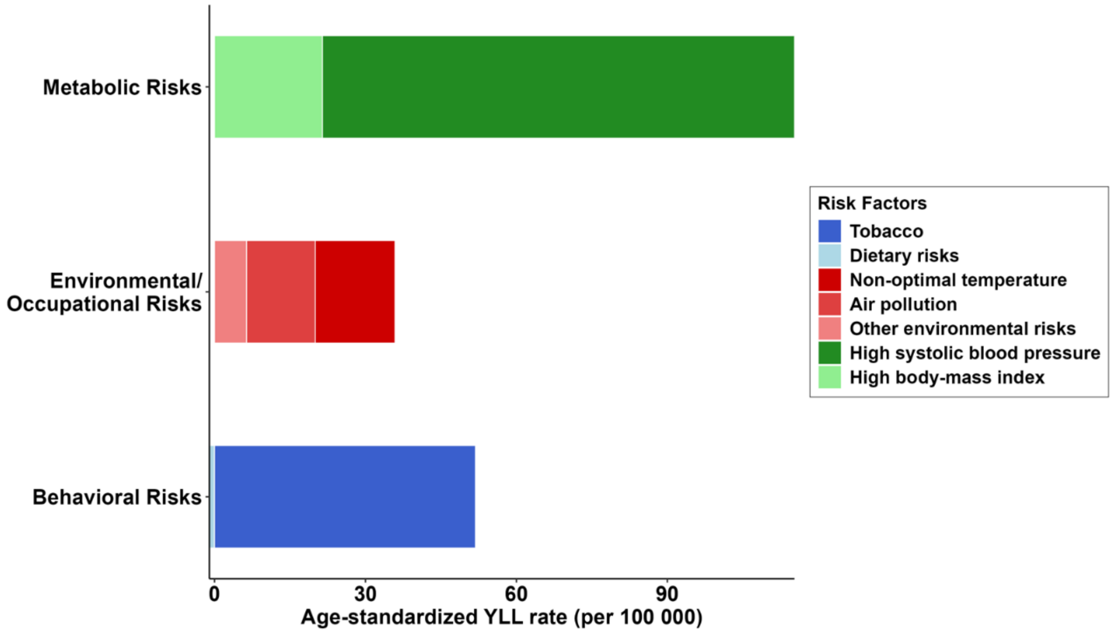

3.10 **Figure S4d: Age-standardized (A) mortality, (B) DALY, (C) YLD, and (D) YLL rate for subarachnoid hemorrhage by types of risk factors, 2021, US**

(A) Death

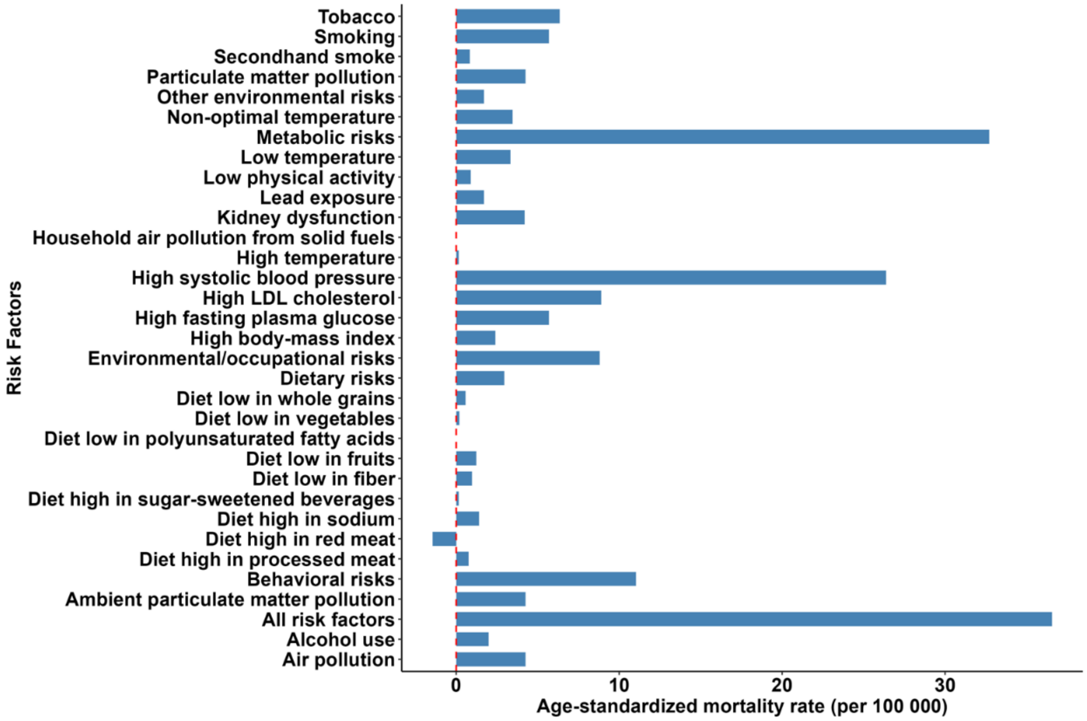

(B) DALYs

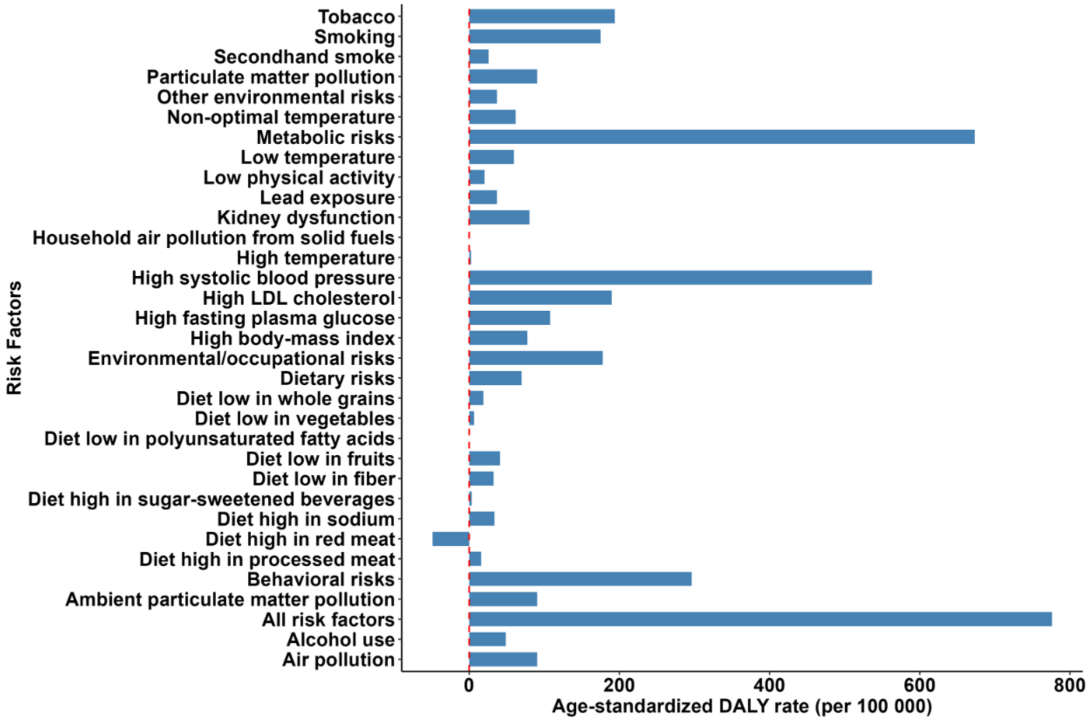

(C) YLDs

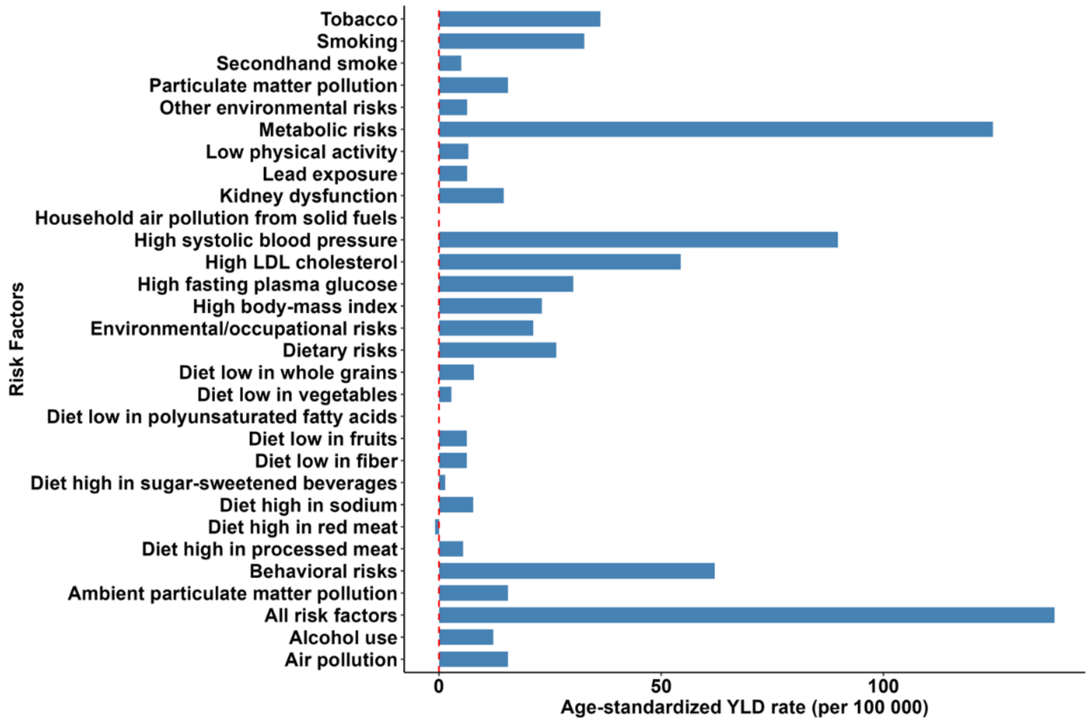

(D) YLLs

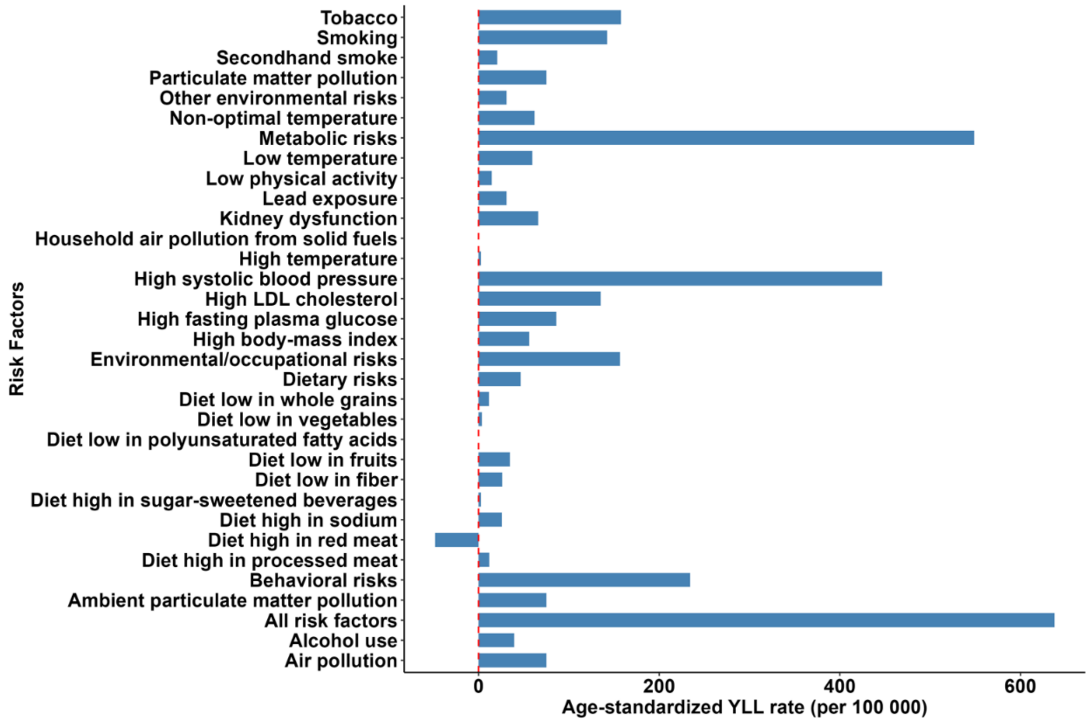

3.11 Figure S5a: Age-standardized (A) mortality, (B) DALY, (C) YLD, and (D) YLL rate for stroke by risk factors, 2021, US

(A) Death

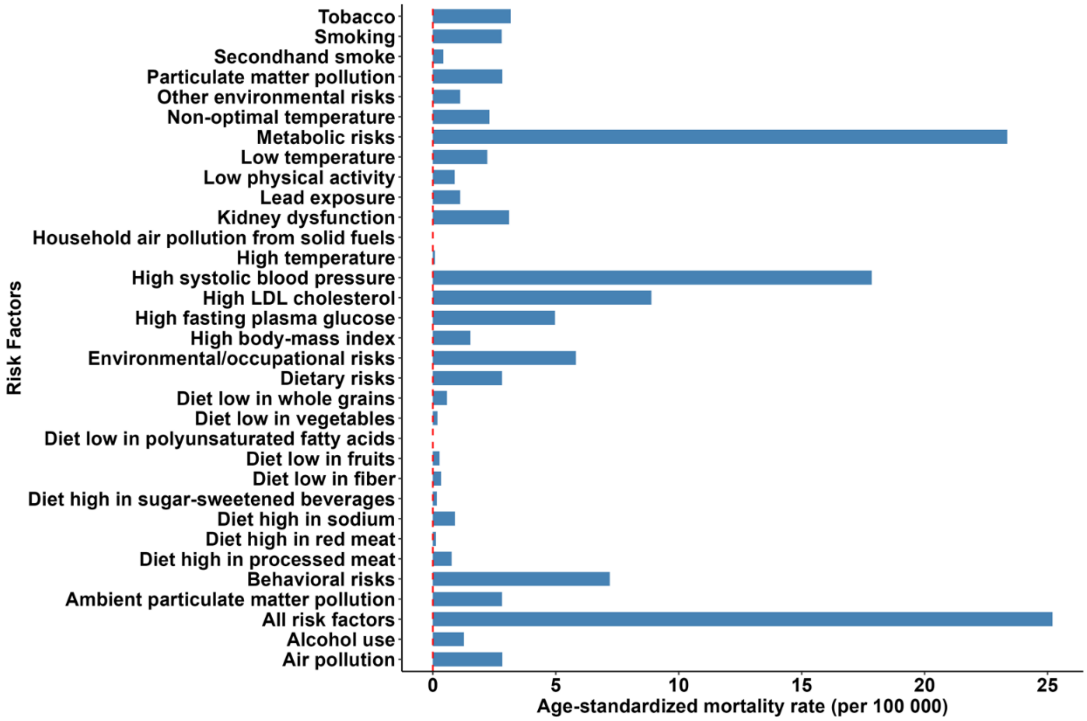

(B) DALYs

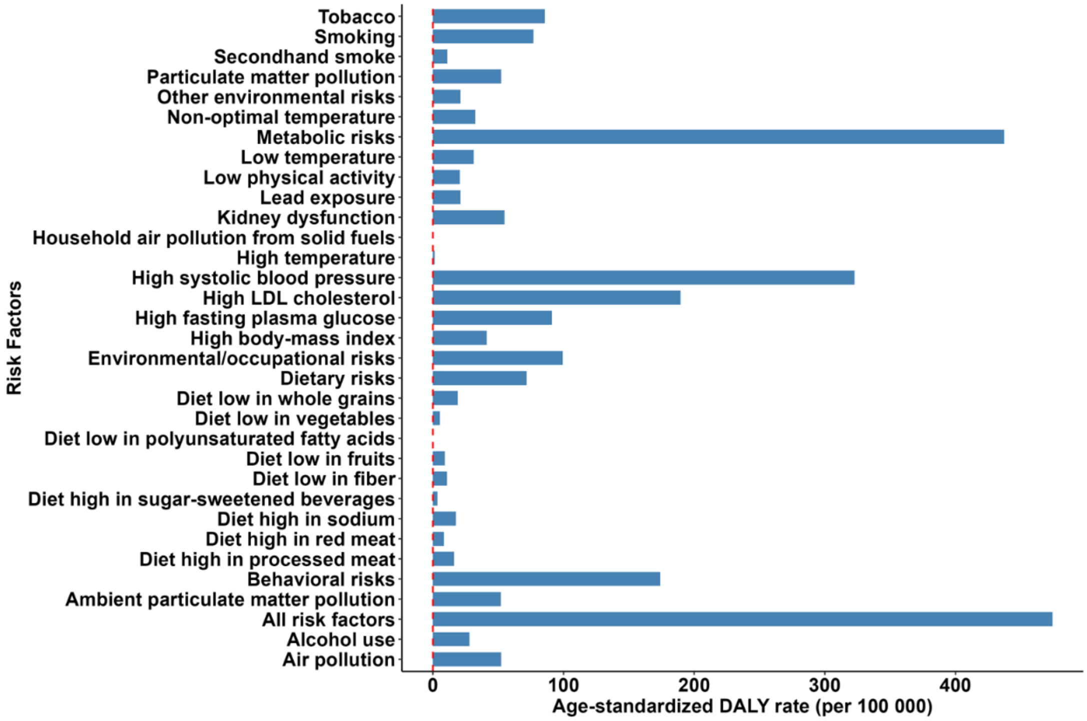

(C) YLDs

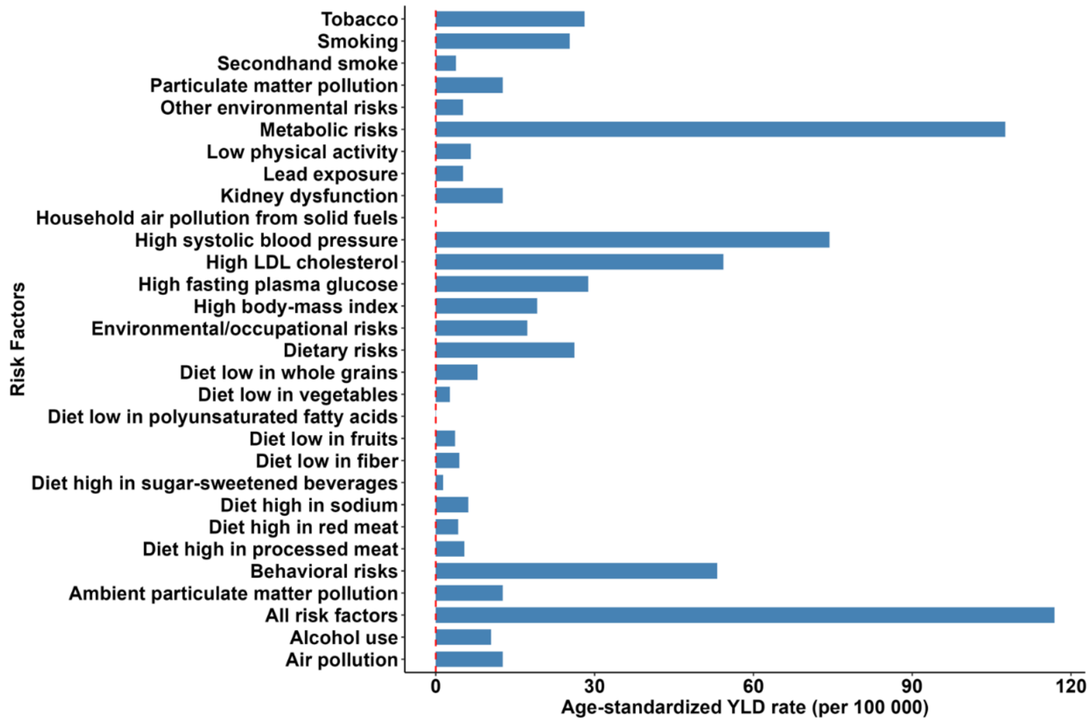

(D) YLLs

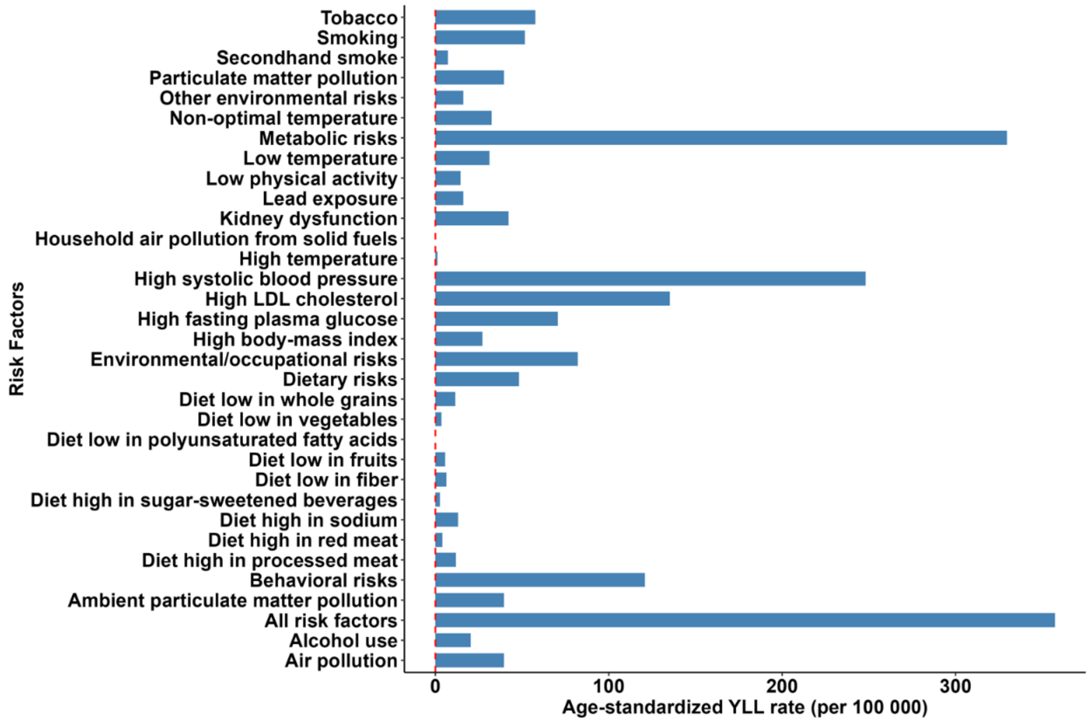

3.12 Figure S5b: Age-standardized (A) mortality, (B) DALY, (C) YLD, and (D) YLL rate for ischemic stroke by risk factors, 2021, US

(A) Death

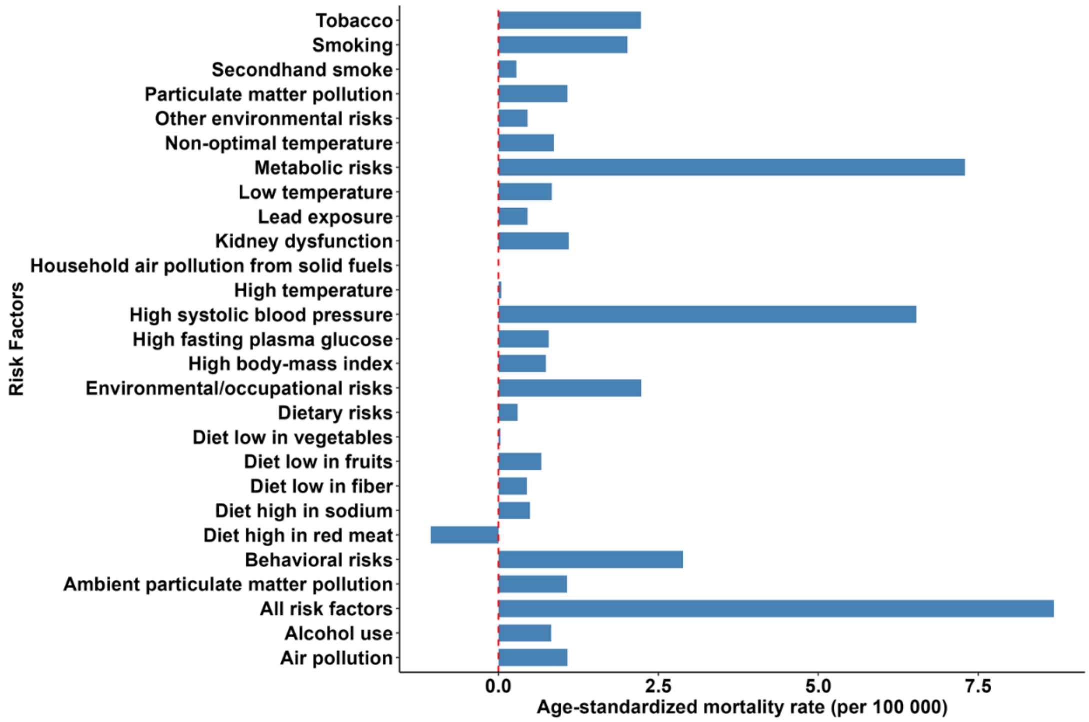

(B) DALYs

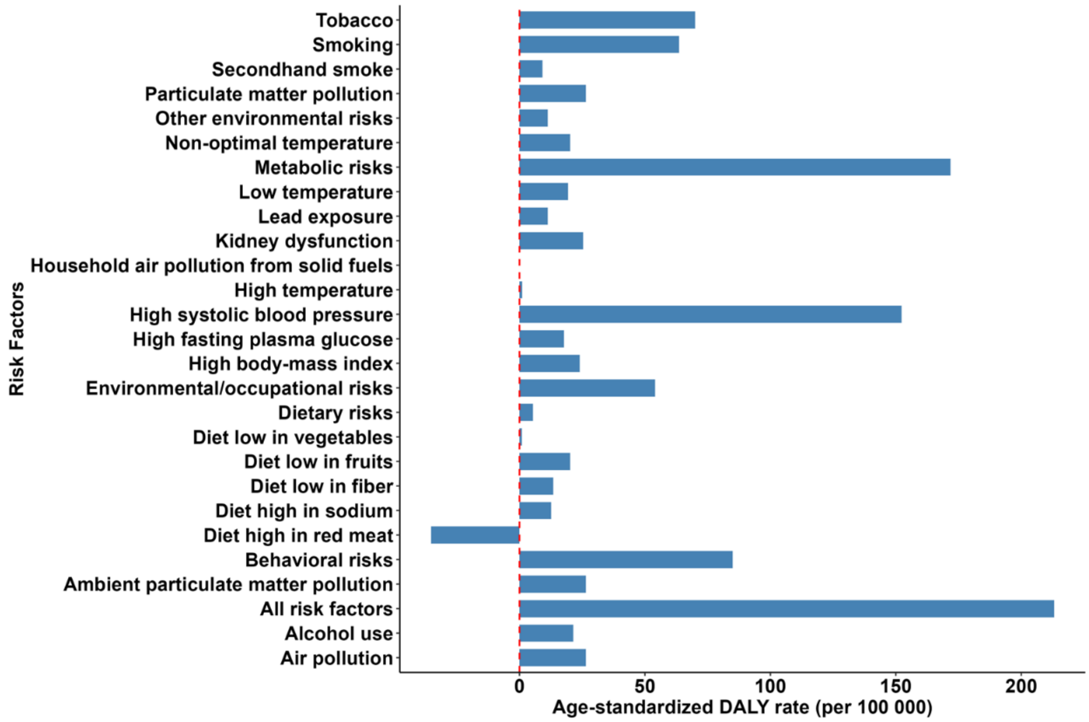

(C) YLDs

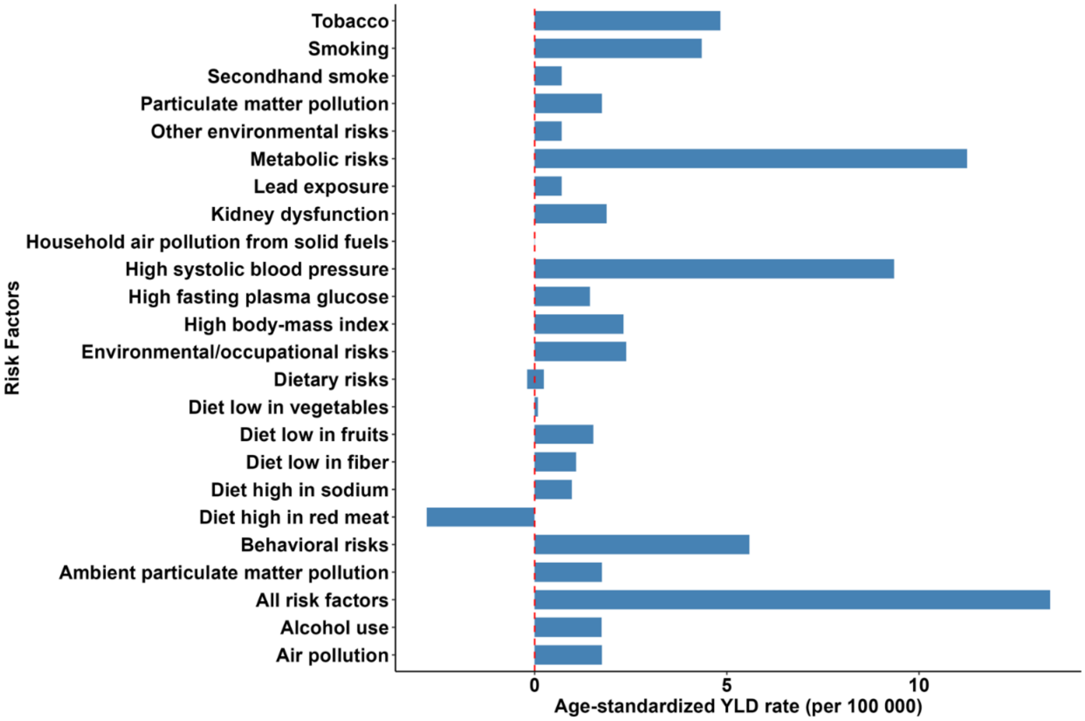

(D) YLLs

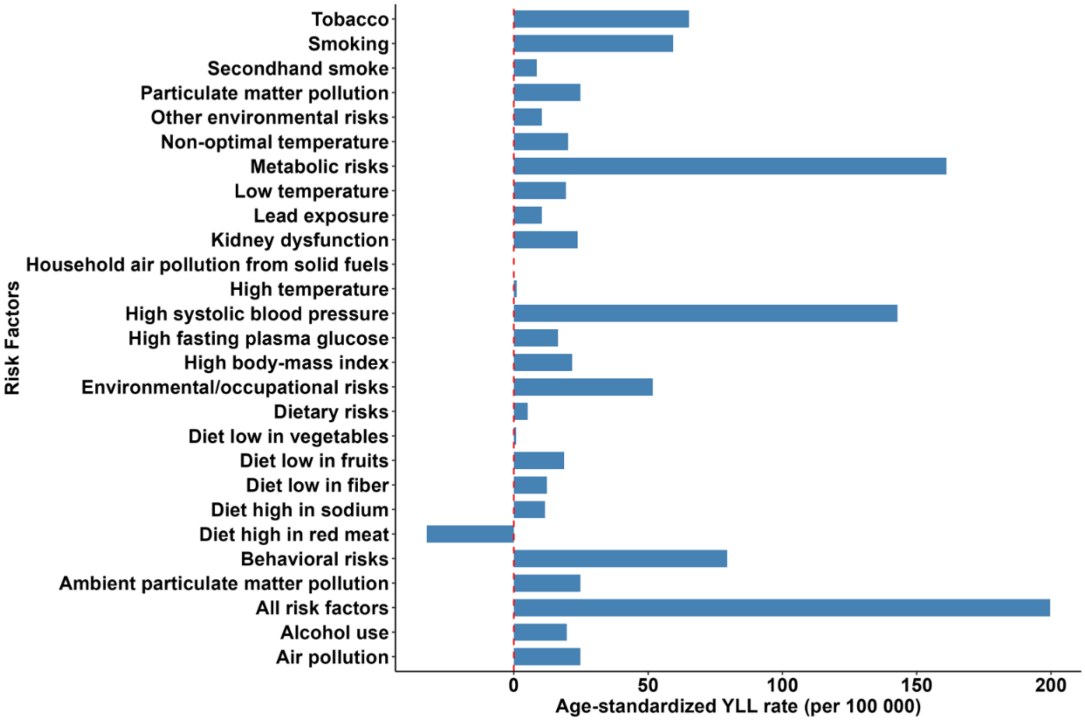

3.13 Figure S5c: Age-standardized (A) mortality, (B) DALY, (C) YLD, and (D) YLL rate for intracerebral hemorrhage by risk factors, 2021, US

(A) Death

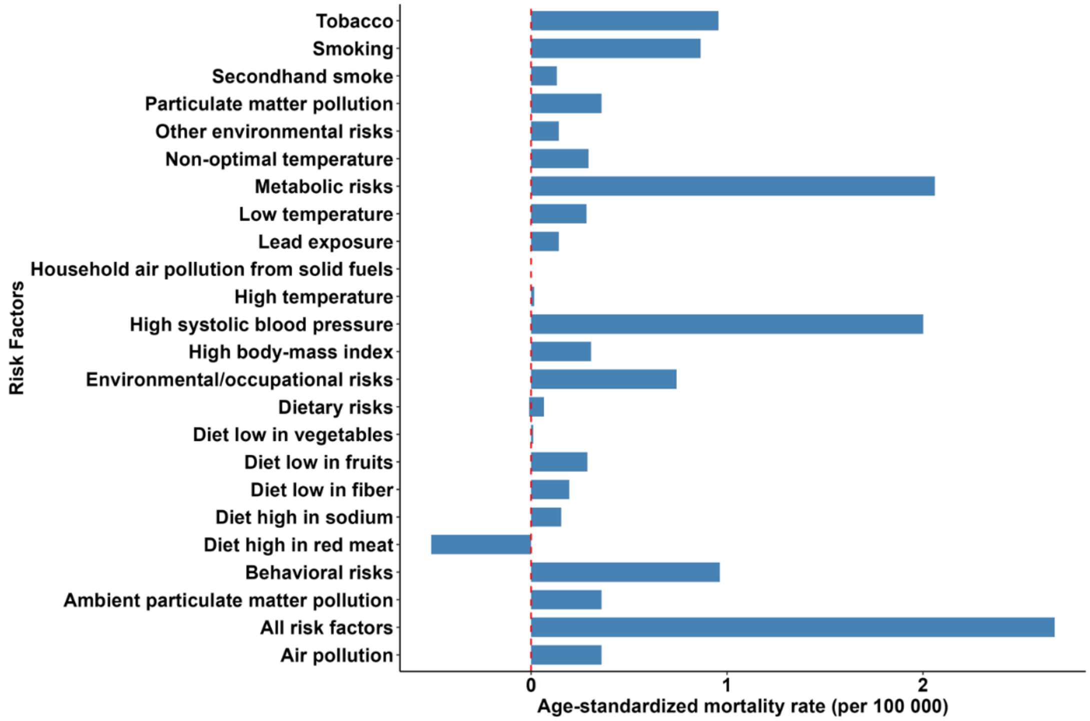

(B) DALYs

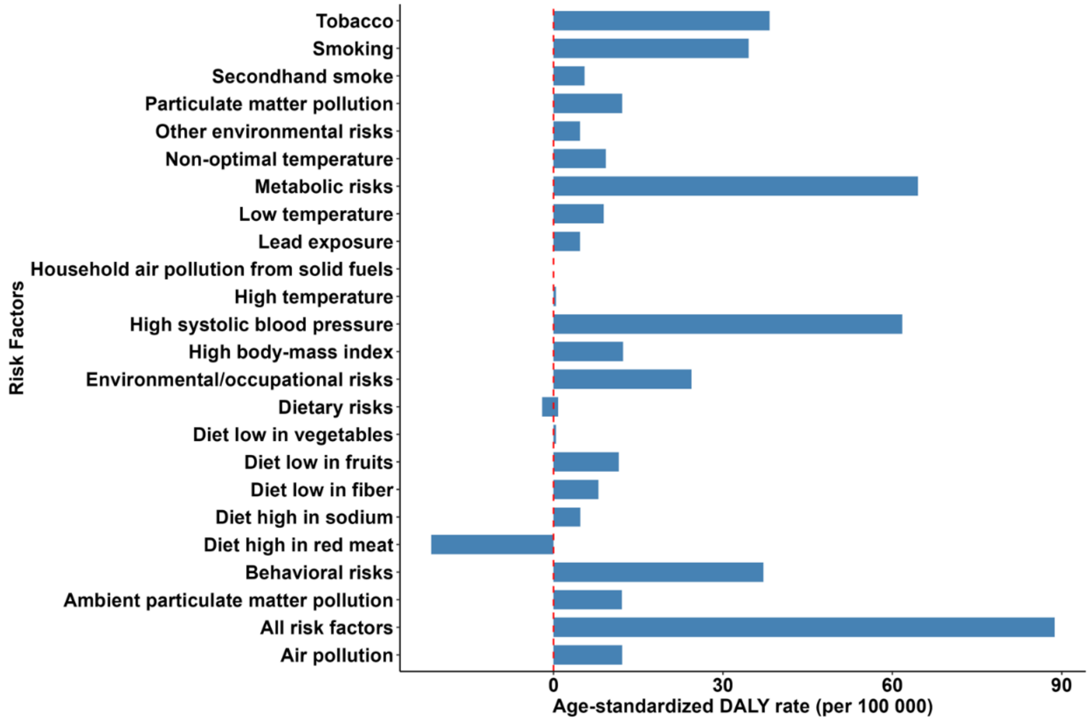

(C) YLDs

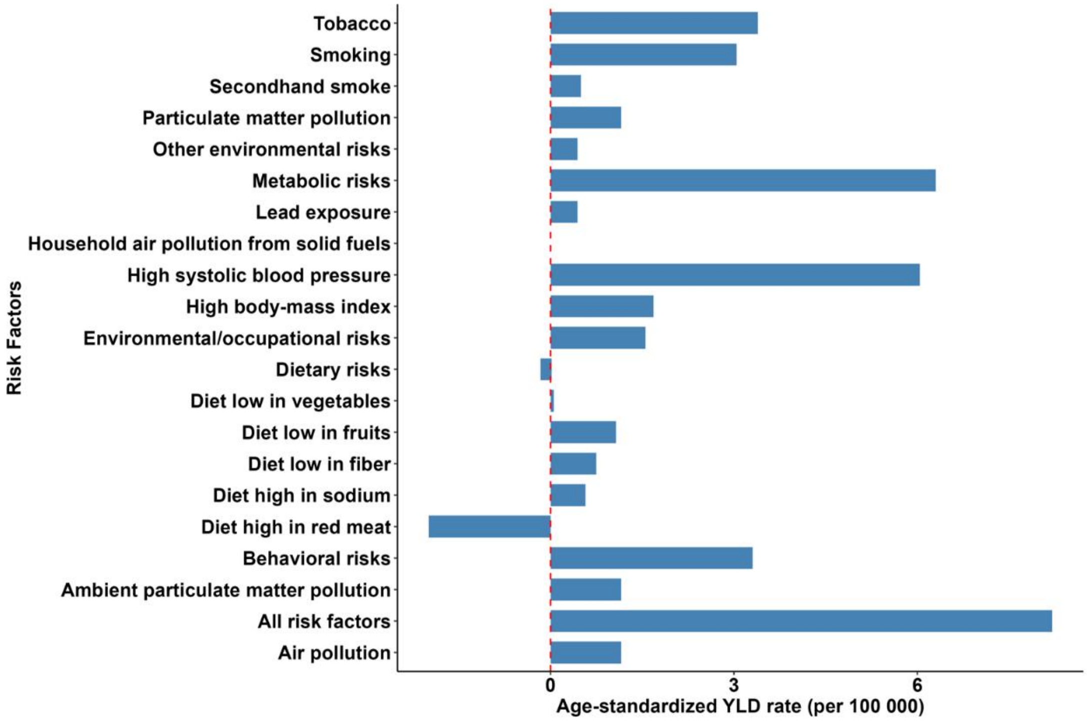

(D) YLLs

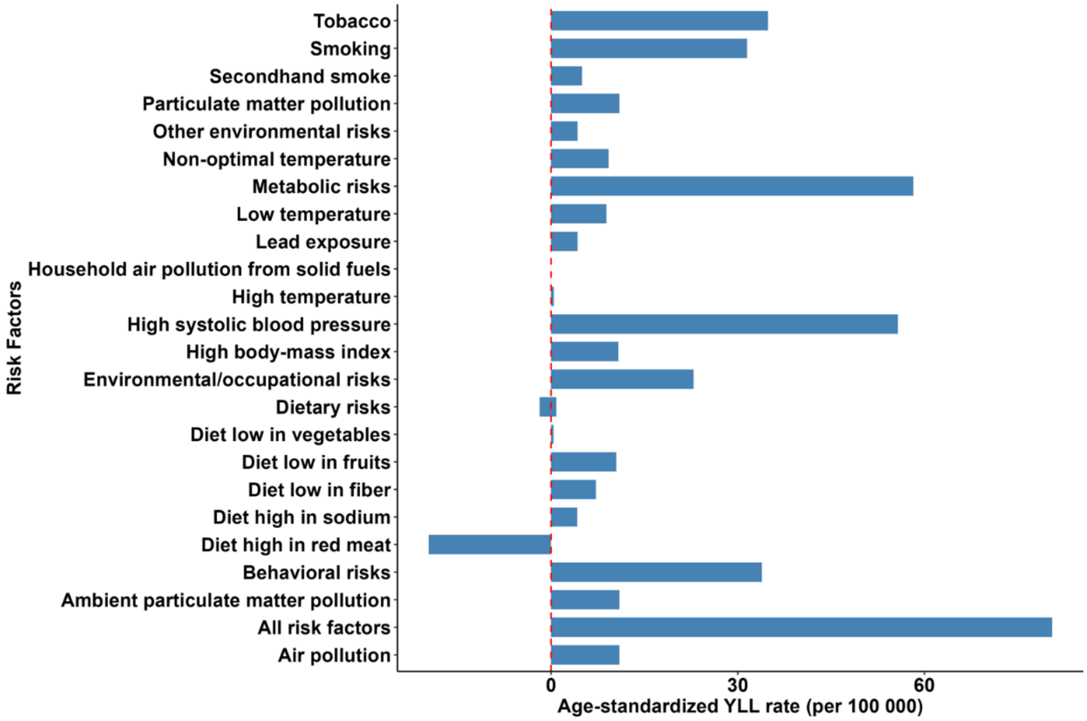

3.14 Figure S5d: Age-standardized (A) mortality, (B) DALY, (C) YLD, and (D) YLL rate for subarachnoid hemorrhage by risk factors, 2021, US
